# Perspectives on invasive amphibians in Brazil

**DOI:** 10.1371/journal.pone.0184703

**Published:** 2017-09-22

**Authors:** Lucas Rodriguez Forti, C. Guilherme Becker, Leandro Tacioli, Vânia Rosa Pereira, André Cid F. A. Santos, Igor Oliveira, Célio F. B. Haddad, Luís Felipe Toledo

**Affiliations:** 1 Laboratório Multiusuário de Bioacústica (LMBio) and Laboratório de História Natural de Anfíbios Brasileiros (LaHNAB), Departamento de Biologia Animal, Instituto de Biologia, Universidade Estadual de Campinas, Campinas, São Paulo, Brazil; 2 Department of Biological Sciences, The University of Alabama, Tuscaloosa, Alabama, United States of America; 3 Centro de Pesquisas Meteorológicas e Climáticas Aplicadas à Agricultura (CEPAGRI), Universidade Estadual de Campinas, Cidade Universitária Zeferino Vaz, Campinas, São Paulo, Brazil; 4 Pontifícia Universidade Católica de São Paulo, Praça Dr. Ermírio de Morais, Sorocaba, São Paulo, Brazil; 5 Departamento de Zoologia, Instituto de Biociências, Universidade Estadual de Paulista, Rio Claro, São Paulo, Brazil; Universitat Trier, GERMANY

## Abstract

Introduced species have the potential to become invasive and jeopardize entire ecosystems. The success of species establishing viable populations outside their original extent depends primarily on favorable climatic conditions in the invasive ranges. Species distribution modeling (SDM) can thus be used to estimate potential habitat suitability for populations of invasive species. Here we review the status of six amphibian species with invasive populations in Brazil (four domestic species and two imported species). We (i) modeled the current habitat suitability and future potential distribution of these six focal species, (ii) reported on the disease status of *Eleutherodactylus johnstonei* and *Phyllodytes luteolus*, and (iii) quantified the acoustic overlap of *P*. *luteolus* and *Leptodactylus labyrinthicus* with three co-occurring native species. Our models indicated that all six invasive species could potentially expand their ranges in Brazil within the next few decades. In addition, our SDMs predicted important expansions in available habitat for 2 out of 6 invasive species under future (2100) climatic conditions. We detected high acoustic niche overlap between invasive and native amphibian species, underscoring that acoustic interference might reduce mating success in local frogs. Despite the American bullfrog *Lithobates catesbeianus* being recognized as a potential reservoir for the frog-killing fungus *Batrachochytrium dendrobatidis* (*Bd*) in Brazil, we did not detect *Bd* in the recently introduced population of *E*. *johnstonei* and *P*. *luteolus* in the State of São Paulo. We emphasize that the number of invasive amphibian species in Brazil is increasing exponentially, highlighting the urgent need to monitor and control these populations and decrease potential impacts on the locally biodiverse wildlife.

## Introduction

Invasive species represent a new element within a long-term naturally established biological community [1, 2]. This often causes ecological imbalances, as native predators, competitors, or endemic pathogens and parasites may not have a strong negative impact on invasive species [3]. Therefore, biological invasions have the potential to cause changes in the distribution of pathogens [4], promote gene invasion [5], and modify the interactions between native species and the structure of natural landscapes [6, 7, 8, 9, 10]. As a consequence, there may be loss of genetic variability and high mortality among native populations. Beyond damaging the environment, populations of invasive species may also pose a threat to human economy and health [11, 12, 13]. As a result, invasion of alien species can cause multiple hazards, which are usually difficult to manage.

The success of invasive species in a new environment depends on a variety of factors, such as their ability to establish and complete their life cycle in the new habitat without their original higher genetic variability [14, 15, 16, 17]. Invasive species may also displace native species out of their original niches, occupy niches that are not used by native species, or sometimes create their own niches [2]. Although a myriad of abiotic and biotic factors dictate the likelihood of biological invasions [18, 19, 20], macro-environmental conditions are usually the main filter for establishment [21, 22]. Environmental modeling is thus a useful tool to predict the likelihood of invasions and also predict current and future invasive population trends [23, 24, 25].

Several amphibians are known for their success as invasive species [2]. Examples include *Eleutherodactylus coqui* and *E*. *planirostris*, originally from the Caribbean and accidentally introduced to Hawaii with the horticultural trade [26, 27]; the cane toad (*Rhinella marina*), from Central and South America, introduced to Australia and in some of the Pacific and Atlantic Islands for pest control [28, 29, 30, 31]; the American bullfrog (*Lithobates catesbeianus*), introduced to Europe, Asia, and South America, mostly for frog farming [32, 31]; and *Xenopus laevis*, an African species introduced in many countries as an animal model for lab research [33, 34]. These species are linked to evolutionary changes in morphology and behavior of native species [35], disease spread [36, 37, 38], and above all, to a variety of ecosystem imbalances and biodiversity loss [2, 39].

In Brazil, the only invasive amphibian species with confirmed impacts on biodiversity is the American bullfrog, *L*. *catesbeianus* [40, 41]. Originally from North America, this species was brought to South America for farming both to supply local restaurants and for the international trade [42, 36, 32]. Among the main impacts observed in the local fauna, *L*. *catesbeianus* negatively impacts native populations [43, 44, 45, 39], causes acoustic interference [46], and competes for resources [47]. In addition, this species is linked to disease spread in several continents, including South America [36, 37].

Three other frog species were recently added to the list of introduced species in Brazil (translocated by human action): *E*. *johnstonei* in the city of São Paulo [48], *Phyllodytes luteolus* in the city of Rio de Janeiro [49], and *Leptodactylus labyrinthicus* in central Amazonia [50]. There are also records of two invasive amphibian species in the archipelago of Fernando de Noronha [51] as well as unconfirmed records of introductions of *Xenopus laevis* and *Pipa carvalhoi* [52, 53, 2, 54]. The potential impacts of these species are yet to be revealed.

Here we review the status of the six amphibian species with confirmed invasive populations in Brazil. To assess potential negative impacts of these invasive species we (1) modeled current and potential future habitat suitability using species distribution modeling (SDM), identifying suitable habitats for population establishment and potential invasion routes. We also (2) analyzed acoustic niche overlap between local and invasive species to infer about call interference that likely reduces mating success. Finally, we (3) screened individuals of *Eleutherodactylus johnstonei* and *Phyllodytes luteolus* for the frog-killing fungus *Batrachochytrium dendrobatidis* (*Bd*) to identify potential *Bd* reservoirs. Combined, these goals provide additional ecological information to guide conservation efforts and manage biological invasions that may aid in mitigation programs to safeguard the local fauna.

## Materials and methods

### Data gathering

We used a combination of field surveys, literature review, personal communications, free database assessment (Sistema de Informação Distribuído para Coleções Biológicas <http://splink.cria.org.br/> and Global Biodiversity Information Facility <http://www.gbif.org/>) to gather spatial and biological information of invasive species in Brazil (further details in S1 and S2 Tables). Although analyses were focused on potential environmental niches in Brazil for discussions on invasion processes, we gathered available geographical information of the whole distributional range for each species, including localities beyond Brazil’s borders (*e*.*g*., occurrence records of *Scinax x-signatus* in Colombia and Guyana, the original distribution of *Eleutherodactylus johnstonei* in the Caribbean region, and current available records for *Lithobates catesbeianus* in North America, Europe and Asia). This procedure was done to capture the species’ full climatic niche, and then project it to the Brazilian territory for interpretation.

To reduce potential taxonomic bias or misidentification we only selected species clearly named in the original database accessed, excluding vague identifications, such as Genus plus sp., cf., or aff. We considered ‘introduced species’ those translocated by human action (accidental or intentional) to areas outside the species’ original distribution [15]. We recognized three categories of introduced species: (1) imported invader–a species that occurs originally abroad with stable population(s) in Brazil; (2) domestic invader–a species that is originally from Brazil with known stable populations outside its original occurrence area; and (3) domestic alien–a species with the same condition of domestic invader, but with unknown population status (*i*.*e*., demography).

We sampled individuals of the only known invasive population of *Eleutherodactylus johnstonei* in Brazil: Brooklin, São Paulo city (23.633° S, 46.682° W). We collected 80 individuals of *E*. *johnstonei* from three locations ~80m apart in January 2015. We sampled individuals of *Phyllodytes luteolus* in Serra do Guararu, municipality of Guarujá (a coastal island), state of São Paulo, southeastern Brazil (23.883° S, 46.166°W), between December 2013 and February 2014. We collected individuals of *P*. *luteolus* in a garden with 320 bromeliads at a private neighborhood. All collected tadpoles and post-metamorphs were deposited at Museu de Zoologia “Prof. Adão José Cardoso”, Universidade Estadual de Campinas (Unicamp), Campinas, São Paulo, Brazil (ZUEC 20931–57, 21245). The following permits were approved for sample collection (Ministério do Meio Ambiente–ICMBio—SISBIO permit number: 42817–4, and Comissão de Ética no Uso do Animal (CEUA)–UNESP #39/2015).

Although southern Brazil has been pointed to as a suitable environment for the establishment of *Xenopus laevis* [25], there are no official records of established populations of this species in the wild [54]. Due to lack of scientific information supporting the introduction of *X*. *laevis* and *Pipa carvalhoi* in the wild (as speculated by [52, 53, 2]), we did not consider these species as invasive in Brazil.

### Species distribution modeling

Occurrence records were inspected and processed in the ArcGIS platform (ESRI, 2010) to avoid data clustering (*e*.*g*., many points in the same locality). To build the SDMs we used all available occurrence records of both native and introduced populations, as well as records of species’ original distribution to capture the climatic niche closest to the species’ realized niche (*e*.*g*. [55]). Determination of native and introduced populations was done by crossing occurrence records and distributional maps available in AmphibiaWeb <http://amphibiaweb.org/> and IUCN Red List of Threatened Species <http://www.iucnredlist.org/>. Models were generated for the complete geographic extent of species occurrences (native and introduced) and then projected within Brazil’s political boundaries.

We downloaded interpolated bioclimatic rasters for the current years (1950 to 2000) from WorldClim <http://www.worldclim.org> at a grid cell resolution of 10 arc-minutes. We also downloaded rasters of projected future climatic conditions downscaled from global climate model (GCM) for representative concentration pathways (RCPs) to the year 2100 from WorldClim, obtained from CMIP5 (IPCC fifth assessment; [56]). We used the Hadley Global Environment Model 2 –Atmosphere Ocean (HADGEM2 –AO), RCP4.5 scenario, which predicts a global surface temperature likely increasing by more than 1.5°C [56]. We chose RCP4.5 scenario because it is parsimonious and likely occurring at the end of the 21^st^ century. Also, we avoided extreme scenarios as RCP8.5, which predicts an increase of global mean surface temperature by the order of almost 5°C [56]. To avoid bias due to multi-collinearity among environmental rasters, we used principal component analysis (PCA) to consolidate cross-correlated bioclimatic variables (we recovered at least 80% of variation with 4 principal components for all species, using all 19 bioclimatic variables). These analyses were performed in R [57] by the use of the following packages: alphashape3d, version 1.2 [58], raster version 2.5–8 [59], and rgdal version 1.1–10 [60].

Models were run with a calibration test including 10% of total occurrence records for each species by random sub-sampling, with 15 replicates and maximum interactions of 5000 in attempt to maximize model performance. Furthermore, SDMs were statistically evaluated by the inspection of Area Under the Receiver Operating Characteristic (ROC) Curve value (AUC). AUC values close to 0.5 indicate model performance no better than random, while AUC values close to 1.0 indicate good model performance [61].

We built species distribution models (SDMs) [62] for six species, namely the domestic invaders: *Leptodactylus labyrinthicus*, *Phyllodytes luteolus*, *Rhinella jimi*, and *Scinax x-signatus*; and also for the imported invaders: *Eleutherodactylus johnstonei* and *Lithobates catesbeianus*. To assess the potential habitat suitability at both current and future climatic conditions, and to investigate potential habitats for population establishment and/or potential invasion routes, we built SDMs using the low-memory multinomial logistic regression, also known as maximum entropy modeling method [61], implemented in the software MaxEnt (version 3.3.3.k). MaxEnt is a largely used machine learning algorithm that builds models of environmental suitability by the use of presence-only species records to project probability densities in covariate space [63]. All models were calibrated with the native range of the species (*e*.*g*.[55]). Then, the common resulting output, a continuous map provided by MaxEnt, was transformed into binary maps (0/1) to better discriminate suitable from non-suitable habitats and also to diminish uncertainty and underestimation of suitable extents (*e*.*g*.[64, 55, 65]). We used max SSS approach (which is based on sensitivity-sensibility sum maximization) as a threshold selection method [66, 67, 68]. In addition, using the binary categorization of the study grid for both models (present and future), we quantified percentage retraction or expansion for each species occurrence range using the reclass function in the Spatial Analyst tool available in Esri/ArcMap platform.

### Bioacoustics

The advertisement calls (*sensu* [69]) of three adult males of *Phyllodytes luteolus* from Guarujá were recorded to confirm species identity and to assess the acoustical overlap with a sympatric and supposedly competitive native species (*Ischnocnema* sp.). Both species were recorded calling at the same time and location. We obtained vocalization data of two males of *P*. *luteolus* (with an *Ischnocnema* male calling in the background) using a Marantz PMD660 digital recorder at a sampling rate of 48 kHz and 16 bit resolution, and using a microphone YOGA 9600EM positioned about 50 cm from the calling males. We recorded a third male of *P*. *luteolus* using a Tascam DR-680 recorder with sampling rate of 48 kHz and 24 bit resolution, using a Sennheiser ME67 microphone positioned about 50 cm from the calling male. Vocalizations obtained in the field were archived at Fonoteca Neotropical Jacques Vielliard (FNJV), Unicamp, Campinas, São Paulo (FNJV 30962–64). Additional recordings of *Leptodactylus pentadactylus* (FNJV 11042) and *L*. *labyrinthicus* (FNJV 13048) were obtained from the FNJV archives. We also downloaded the recordings of *Leptodactylus knudseni* (CENBAM 0645) from the website CENBAM database (http://ppbio.inpa.gov.br). We compared the bioacoustic profile of these three *Leptodactylus* species. With the invasive population of *L*. *labyrinthicus*, these three species are now sympatric in Manaus, state of Amazonas. We generated sonograms in Raven 1.4 (Bioacoustics Research Program, 2011), using settings of FFT (Fast Fourier Transformation) of 512 samples and a window overlap of 50 percent.

To obtain spectral similarity among calls of native and invasive species we assessed the power spectra and the energy distribution among frequencies transformed with a FFT of 512 samples using the software WASIS β [70]. We conducted a spectral filtering by selecting the power bands within the species frequency range. Then, we evaluated the spectral overlap among species by means of Pearson correlations using WASIS β. The correlation coefficient was considered as a proxy of spectral overlap (SO), from 0 (no correlation) to 1 (identical call). Additionally, we obtained dominant frequency and call duration with the software Raven 1.4 (Bioacoustics Research Program, 2011).

### Disease assessment

We swabbed 80 individuals of *Eleutherodactylus johnstonei* and 26 individuals of *Phyllodytes luteolus* immediately after capture for later detection of the frog-killing fungus *Batrachochytrium dendrobatidis* [71]. We tested swabs for *Bd* in singlicate using Taqman qPCR [72, 71] with standards of 0.1, 1, 10, 100, and 1000 zoospore genomic equivalents (GE) to detect *Bd* in each individual frog. We used strain CLFT 023 as a quantitative standard for all qPCR reactions [73]. We considered samples positive for *Bd* when GE was >1 [74].

## Results

**Fig 1:**
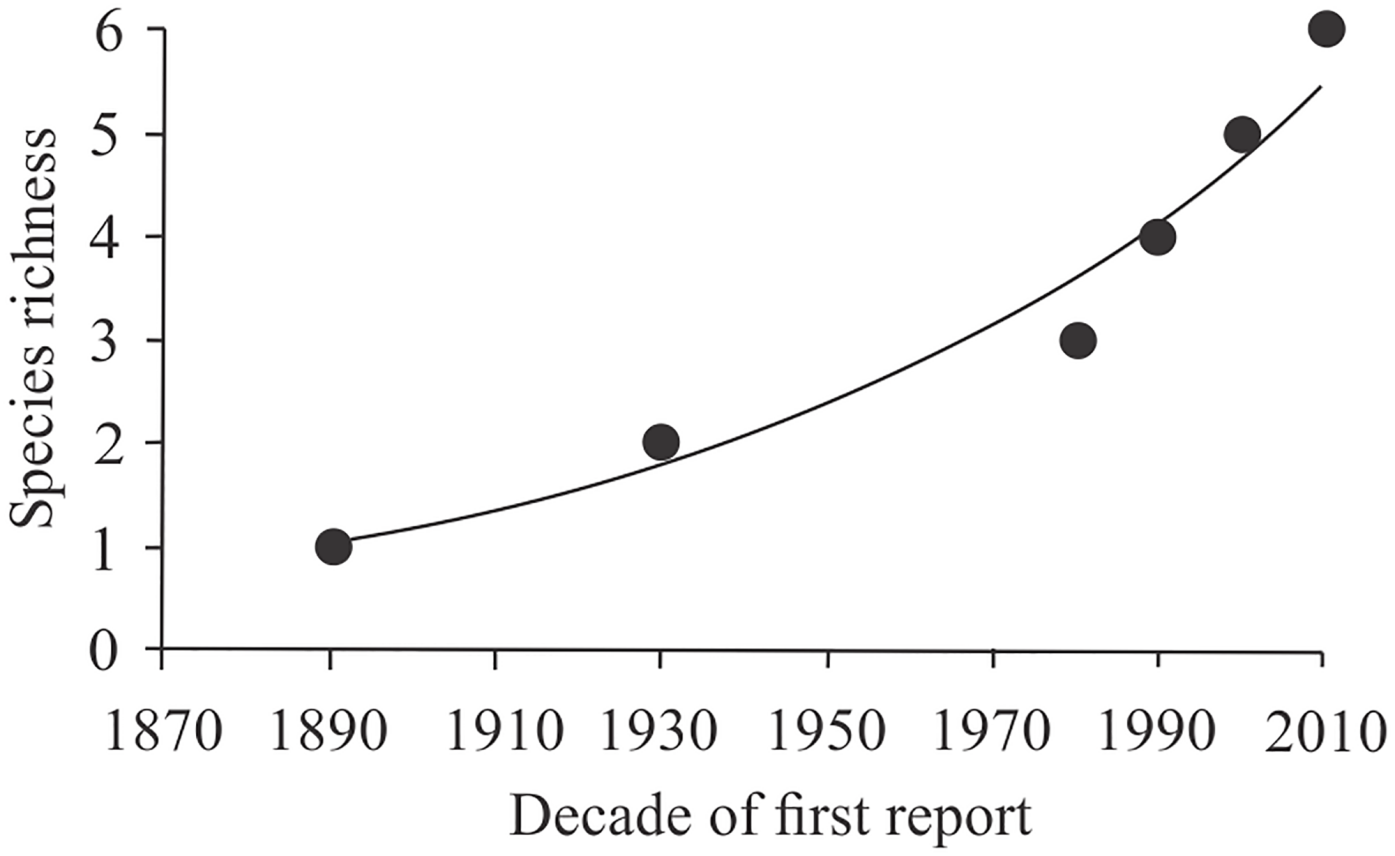
Cumulative invasive species richness in Brazil. Quadratic fit is shown (r^2^ = 0.973).

We synthesized information of invasive amphibian species in Brazil, providing new records for expanding populations for five of the six invasive species (Table 1). Distribution records for all species are in S2 Table and S1 Fig. We found that the reported number of frog species with invasive populations in Brazil is increasing exponentially (Fig 1).

**Table 1:** Invasive anuran species in Brazil. Asterisks denote novel data from the present study.

Invasive species	Category	Original distribution (Frost 2016)	Localities with invasive populations	Estimated decade of introduction	Introduction reason	Source
*Eleutherodactylus johnstonei*	Imported invasive	Lesser Antilles	São Paulo-SP	Not registered before the 2010s	Unintentional, probably with ornamental plants	Melo et al., 2014 [48]
*Leptodactylus labyrinthicus*	Domestic invasive	Cerrados and Caatingas of central and southeastern Brazil and eastern Paraguay	Manaus, AM; Canutama, AM*; Belém, PA; Alter do Chão, PA; Santarém, PA*; Presidente Médici, RO*	Unknown, but reports date back to the 1990s	Commercial production	Carvalho et al., 2013 [50]; Neckel-Oliveira et al., 2000 [75]
*Lithobates catesbeianus*	Imported invasive	North America	130 Brazilian municipalities, mainly in the Atlantic forest	First official shipment in the1930s	Commercial production	Giovanelli et al., 2008 [40]; Both et al., 2011 [41]
*Phyllodytes luteolus*	Domestic invasive and Domestic alien	Coastal region of eastern Brazil from Paraíba to northern Rio de Janeiro state, northeastern Minas Gerais, and southern Bahia	Rio de Janeiro, RJ; Guarujá, SP*; Rio Claro, SP (only few individuals)*	First records in the 2000s for RJ and 2010s for SP	Unintentional, probably with ornamental plants	Salles and Silva-Soares, 2010 [49]; present study
*Rhinella jimi*	Domestic invasive	Northeastern Brazil from Maranhão to Bahia and Piauí at elevations of 400–500 m	Fernando de Noronha, PE	Between 1888 and 1973	Insect pest control	Olson, 1981[76]; Oren, 1984 [51]; Vicente et al., 1990 [77]; Toledo and Ribeiro, 2009 [78]; Tolledo et al., 2014 [79]
*Scinax x-signatus*	Domestic invasive	Non-forest habitats of northern Colombia and Venezuela to Surinam; eastern, southern, and southeastern Brazil	Fernando de Noronha, PE	First records published date from the 1980s	Unintentional, probably with ornamental plants or food supply to the island	Oren, 1984 [51]; Toledo and Ribeiro,2009 [78]

### Species distribution modeling

**Fig 2:**
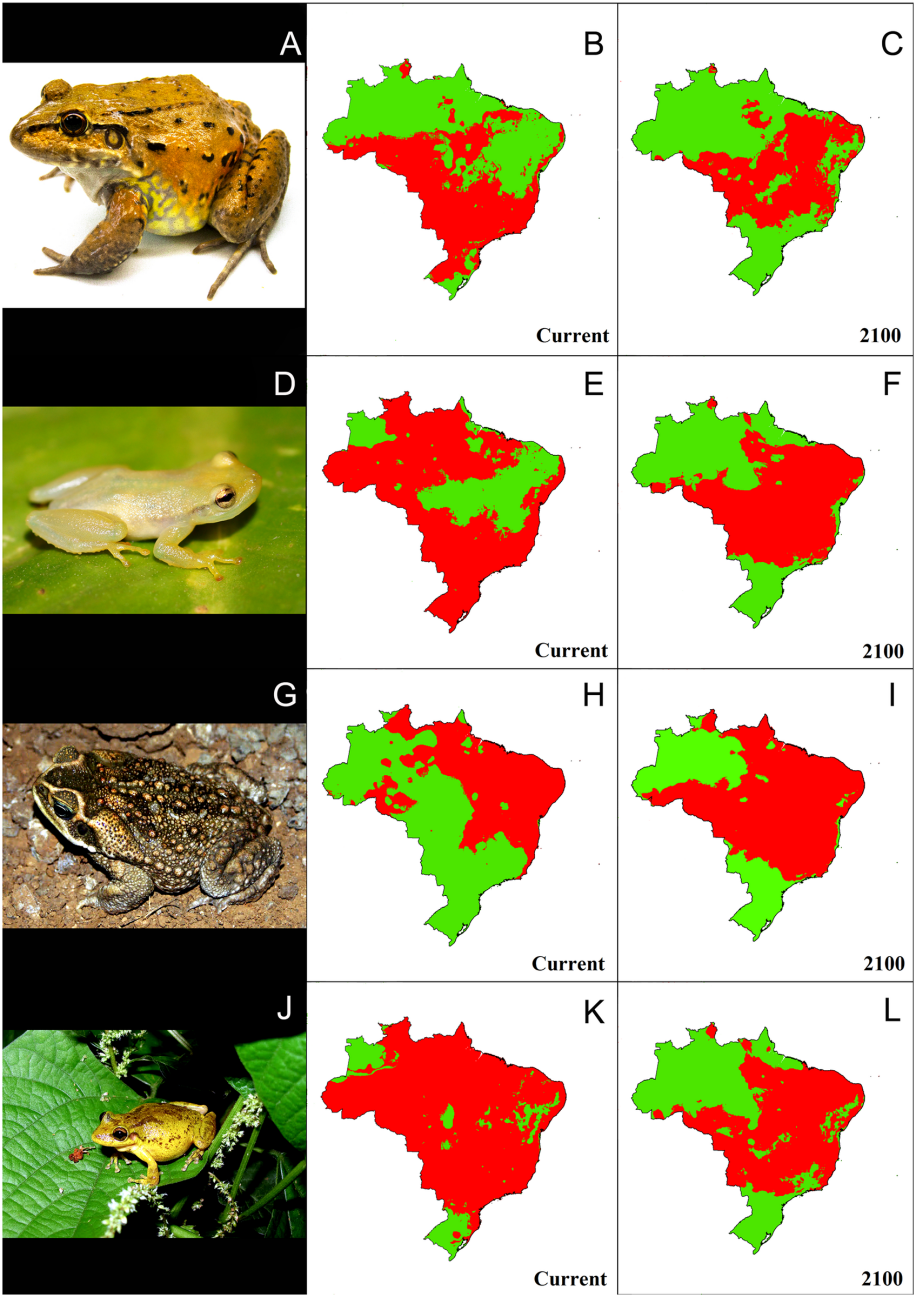
Current distribution models of domestic invasive anuran species in Brazil. *Leptodactylus labyrinthicus* (A), *Phyllodytes luteolus* (D), *Rhinella jimi* (G), and *Scinax x-signatus* (J). Species distribution models projected to 2100 as binary maps showing current (B, E, H, and K) and future (C, F, I, and L) potential suitable habitats (red spots) (RCP4.5 scenario provided by IPCC fifth assessment; [56]).

**Fig 3:**
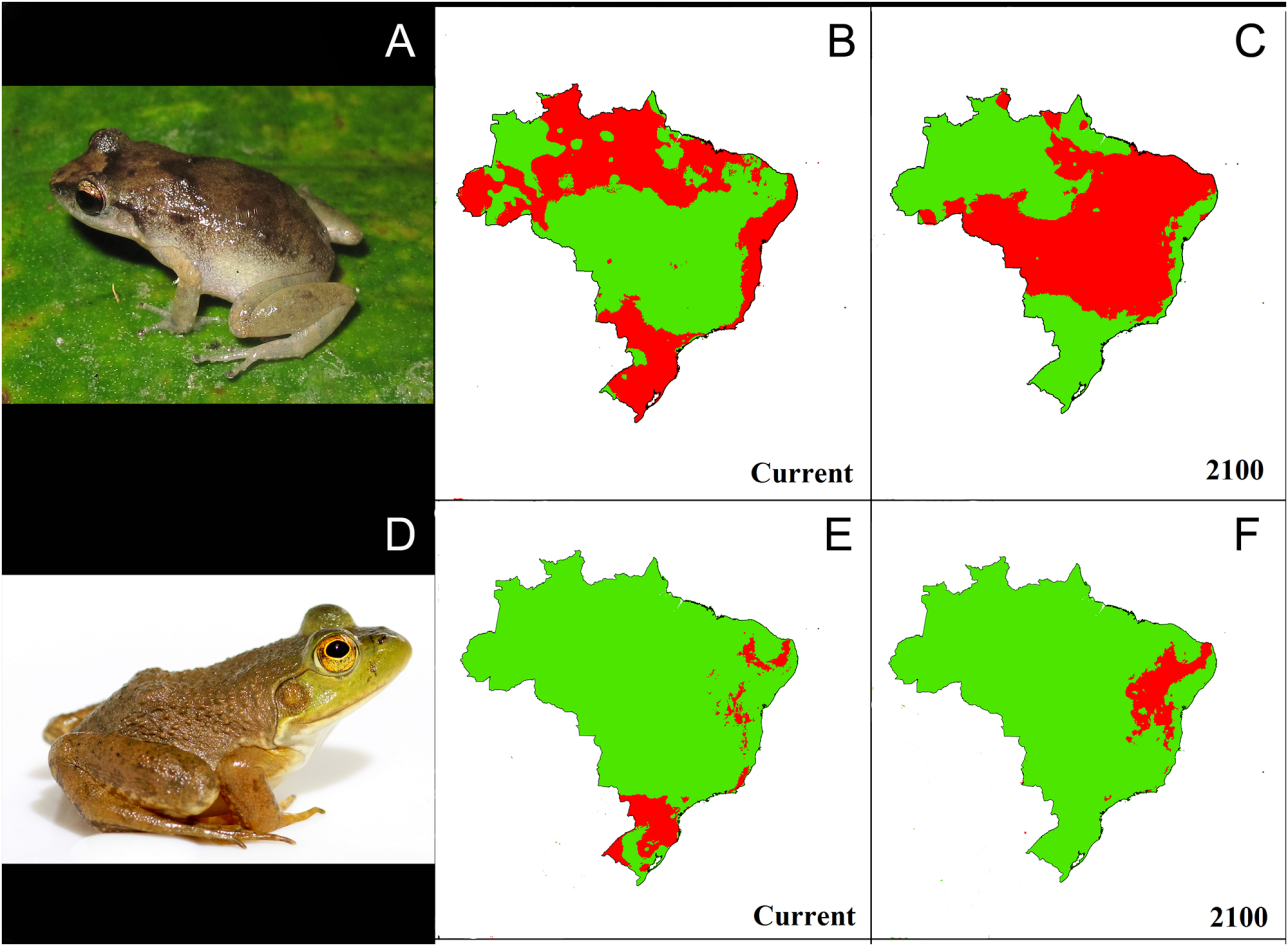
Current distribution modes of the following imported invasive anuran species in Brazil. *Eleutherodactylus johnstonei* (A), and *Lithobates catesbeianus* (D). Species Distribution Models projected to 2100 as binary maps showing current (B and E) and future (C and F) potential suitable habitats (red spots) (RCP4.5 scenario provided by IPCC fifth assessment; [56]).

All SDMs showed good discrimination capacity for training with averaged AUC values of 0.81 (*Lithobates catesbeianus*), 0.93 (*Scinax x-signatus*), 0.96 (*Leptodactylus labyrinthicus*), and 0.97 (*Phyllodytes luteolus*, *Rhinella jimi*, and *Eleutherodactylus johnstonei*). AUC equally weights omission and commission errors [80], and can be considered as a good measure of model performance [81], providing added reliability to the models. Therefore, none of the models were discarded during habitat suitability analyses. Overall, SDMs for future climatic habitat suitability showed contrasting patterns across species (Figs 2 and 3).

### Domestic invaders

Current SDM projections for *L*. *labyrinthicus* indicated high habitat suitability in the southern and eastern Brazilian Amazon, most of Brazil’s Atlantic forest, and a fair amount of the Brazilian Cerrado. However, future predictions showed a trend of decreasing habitat suitability in small portions of southern Amazonia and eastern Atlantic forest, with remaining available habitats concentrated in Cerrado and Caatinga. It is important to notice that our prediction also suggested high habitat unsuitability in the southern section of the Atlantic forest. Our models indicated that *L*. *labyrinthicus* might suffer a retraction of 8% in its total geographical range until 2100. Models built for *P*. *luteolus* indicated widespread suitable habitats throughout Amazonia, the Atlantic forest, and part of the Cerrado, while future predictions showed an increase in potential suitable habitats in the Cerrado. However, we found that the total geographic range for *P*. *luteolus* is projected to reduce by 16% in 2100. SDMs for the toad *R*. *jimi* predicted large habitat suitability in the Amazon, Cerrado, Atlantic forest, and in the archipelago of Fernando de Noronha, where this species was introduced. Nevertheless, SDMs indicated expansions in habitat suitability toward southwestern Amazon through the Cerrado and the Atlantic forest. In the future (2100), our models indicate that this species may expand its geographic range by 22%. Finally, models pointed to current suitable habitats for *S*. *x-signatus* through the whole country, matching its available occurrence points. However, our models predicted that one third of its suitable habitats will be gone by 2100, with most habitat retraction predicted for the Amazon and southern Atlantic forest.

### Imported invaders

Our models for current suitable habitats available for *E*. *johnstonei* showed a fragmented pattern for potential distribution. Suitable areas for this species were concentrated in the southern and coastal Atlantic forest and portions of the Amazon forest. Nevertheless, future potential distribution indicated a dramatic range shift, with a 94% increase in distributional range. Under this scenario, we found that the southern Atlantic forest may become climatically unsuitable for *E*. *johnstonei* while the Cerrado at the same time that some areas of the Amazon forest may become suitable for this species. SDMs for *L*. *catesbeianus* showed current habitat suitability mostly in the southern Atlantic forest, with some pockets of highly suitable habitats in northeastern Brazil. Under future climatic conditions, our models pointed to an increase in habitat suitability for *L*. *catesbeianus* in the northeastern Atlantic forest, very small areas of suitable habitats in the southeast, and unsuitable habitats throughout the south. Our spatial analysis projected a slight range retraction (0.5%) for *L*. *catesbeianus* in 2100.

### Bioacoustics and disease assessment

**Fig 4:**
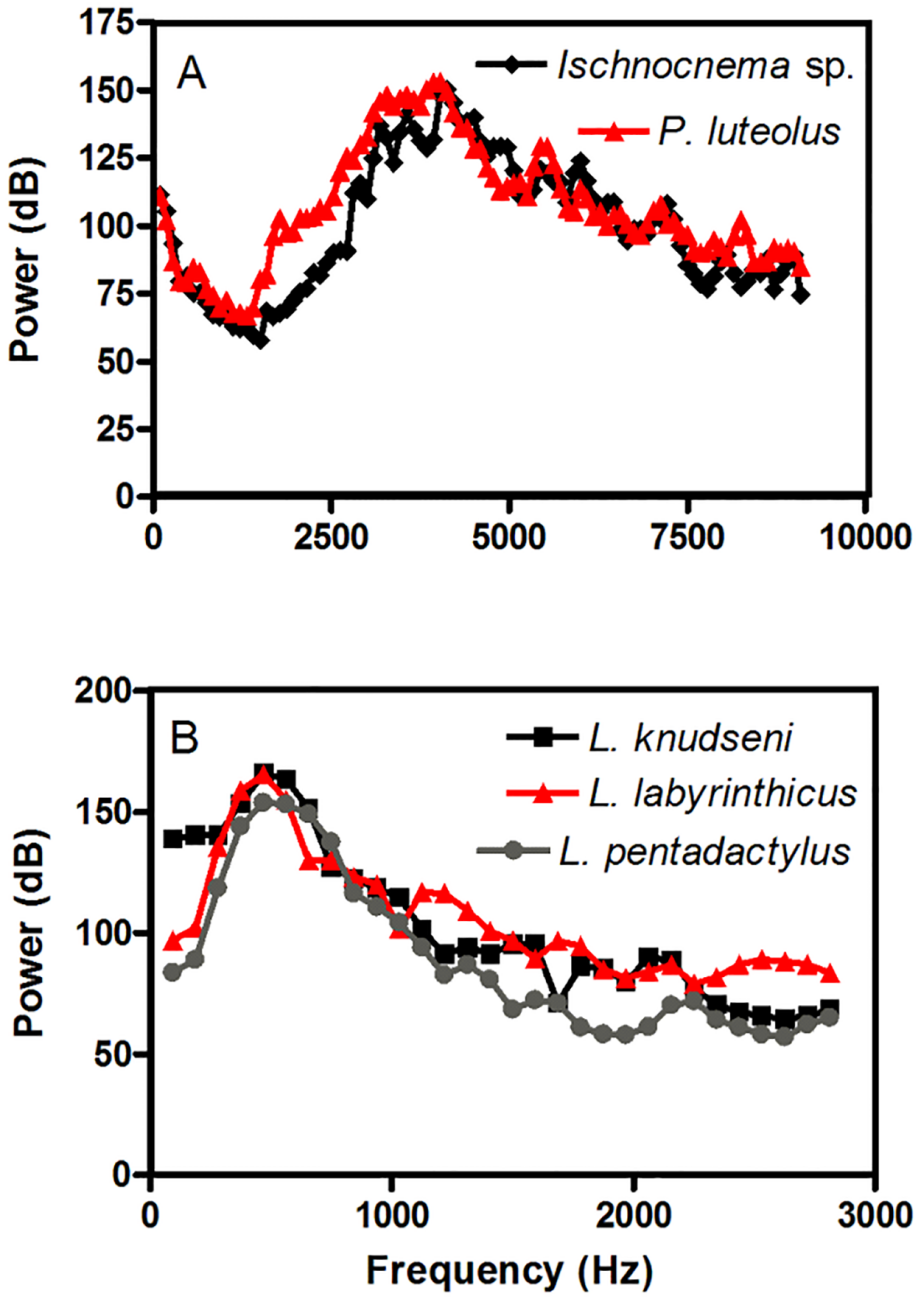
Power spectra of the advertisement calls of *Ischnocnema* sp. and *Phyllodytes luteolus* (A), *Leptodactylus knudseni*, *L*. *labyrinthicus*, and *L*. *pentadactylus* (B).

**Fig 5:**
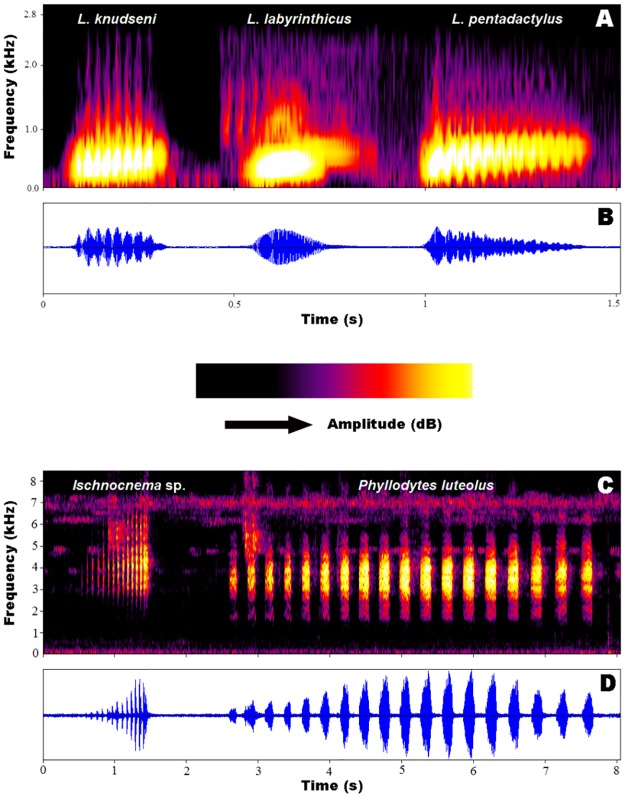
**Audiospectogram (A) and oscillogram (B) of the advertisement calls of three congeneric *Leptodactylus* spp. that are synchronopatric in Manaus, Amazonas, northern Brazil**. Audiospectogram (C) and oscillogram (D) of the advertisement calls of sympatric *Ischnocnema* sp. and *Phyllodytes luteolus* synchronopatric at Serra do Guararu, Guarujá, São Paulo, in southeastern Brazil.

We found a high spectral overlap between the calls of the invasive species *Phyllodytes luteolus* and the native *Ischnocnema* sp. (SO = 0.88;Fig 4a). Despite this spectral overlap, the calls of these species showed distinct temporal structures. *Phyllodytes luteolus* showed more spaced pulses and longer call duration than *Ischnocnema* sp. (Fig 5c and 5d). The spectral overlap index between the calls of *Leptodactylus labyrinthicus* (invasive) and *L*. *knudseni* (native) was 0.88 and between those of *L*. *labyrinthicus* and *L*. *pentadactylus* was even higher (SO = 0.93; Fig 4b). The advertisement call of *L*. *knudseni* had the same duration and dominant frequency when compared to the call of the invasive species (about 0.2 s and 375 Hz). However, the dominant frequency of the call of *L*. *pentadactylus* was higher than that of the invasive species (about 470 Hz) and also differed in call duration (about 0.5 s) (Fig 5a). Despite the high spectral overlap, the calls of these three species had substantial structural differences. Calls of *Leptodactylus knudseni* and *L*. *pentadactylus* are trills, while calls of *L*. *labyrinthicus* have a tonal structure. Pulses in the calls of *L*. *pentadactylus* were also more fused than those of *L*. *knudseni* (Fig 5b).

None of the sampled individuals of *Eleutherodactylus johnstonei* and *Phyllodytes luteolus* tested positive for the chytrid fungus *Batrachochytrium dendrobatidis*.

## Discussion

Our study indicates that the number of invasive amphibian species in Brazil is increasing exponentially, with at least two of these species potentially expanding their ranges in the near future. We also found that invasive populations of two species did not test positive for the lethal *Bd* fungus, which reduces the likelihood of introductions of novel pathogen strains. Our combined results highlight the need for further monitoring of invasive amphibians in Brazil and indicate that stricter regulations regarding the international plant trade are needed to prevent further introductions such as the recent case of *Eleutherodactylus johnstonei* in the city of São Paulo.

### Habitat suitability and potential distribution

*Leptodactylus labyrinthicus* was the only of our focal invasive species showing an isolated occurrence point outside our model predictions. Specifically, some individuals of *L*. *labyrinthicus* were recorded in Manaus, Brazilian Amazon, though our SDMs did not indicate suitable habitats for the species in that particular region. Conversely, our SDMs indicated large suitable habitats for *L*. *labyrinthicus* in the southern Amazon basin, which did not match its likely current distribution (see The IUCN Red List of Threatened Species for further details), but may represent a potential invasion route (Fig 2). Records of *L*. *labyrinthicus* from Manaus may characterize recently introduced populations and, therefore, monitoring the area where these individuals were captured is needed to gather detailed information about the status of this potential invasion. Also, when building SDMs we assumed that occurrence localities represent source habitats, not sink ones, which may harbor species without the minimum conditions to maintain populations without immigration flux [61]. Such assumption lacks ecological realism when dealing with highly vagile species [82] such as *L*. *labyrinthicus* [83]. Under current climate conditions, southern parts of the Amazon may be particularly suitable for this species not only due to favorable climate, but also due to recent deforestation practices [84]. Conversely, future climate itself may require species’ distributional shifts and also may interfere in the persistence of *L*. *labyrinthicus* within its original distribution, potentially driving much of its range towards northeastern Brazil (Fig 2).

The second species we modeled, *Phyllodytes luteolus*, is originally restricted to a narrow strip along the coastal Atlantic forest from the states of Espírito Santo to Pernambuco [85]. Conversely, SDM outputs for this species provide evidence of wider suitable habitats under current climates throughout the southern Atlantic forest, Cerrado, and the Amazon forest (Fig 2), which may explain punctual and recent success for this species in the states of Rio de Janeiro [49] and São Paulo (current study). This pattern may suggest that other factors such as interspecific competition and predation may constrain the expansion of this species for such distributional range. Additionally, populations of *P*. *luteolus* may be declining according to expert opinion [85]. Avoiding dispersal of *P*. *luteolus* individuals by the local and the international bromeliad trade may also contribute to the preservation of its native populations.

*Rhinella jimi* is another non-threatened domestic invader species according to IUCN. *R*. *jimi* is endemic to inland Brazil; it was introduced in the main island of the archipelago of Fernando de Noronha between the decades of 1890 and 1970 [51]. Amerigo Vespucci visited the island in 1503 and in a letter to Piero Soderini he mentioned the presence of skinks (*Trachylepis atlantica*) and worm lizards (*Amphisbaena ridleyi*), but did not refer to amphibians [86]. Charles Darwin sampled terrestrial vertebrates there in 1832, but did not report any amphibian [87]. Likewise, Henry Nicholas Ridley did not report any frog or toad living in the archipelago towards the end of the 1880’s [88], nor Alfredo Tito dos Santos, which only reported invasive tegu lizards (*Salvator merianae*) and tortoises (probably *Chelonoidis* sp., but currently absent in the island) [89]. Only in the 1980’s other authors reported the presence of amphibians in Fernando de Noronha [76, 51, 90]. Besides that, some specimens housed at the Smithsonian National Museum of Natural History (USNM 196575; 346812–17) were collected in 1973. During this 85-year time period (between 1888 –last trustable animal-oriented study and 1973 –first collected individual), the native island inhabitants speculate that the priest Francisco Adelino de Brito Dantas, who lived there during the end of the 19th century [91], might have brought some toads (*R*. *jimi*) from the mainland to control insect herbivores that were attacking his crops. Alternatively, during World War II (1939–1945), the American army established a military base in Fernando de Noronha, and there are also speculations that they brought some toads as a way of mosquito control [13]; that was a common practice of the North American government at that time (*e*.*g*., [92]). Both possibilities fall within our confirmed temporal window (1888–1973). Thus, measurements of the impacts caused by *R*. *jimi* on native organisms of Fernando de Noronha are needed before implementation of any management plan. Moreover, our SDMs suggest suitable current habitats for *R*. *jimi* in the Cerrado, and future suitability, with a large range expansion, in southern Amazonia, which may translate to future range shifts westward.

Similarly to *R*. *jimi* and in the same period of time, the treefrog *Scinax x-signatus* was also introduced to Fernando de Noronha, though likely unintentionally. The same recommendations made for *R*. *jimi* are applicable in this case. *S*. *x-signatus* has very wide inland distribution in Brazil, virtually occurring in all of the existing biomes within the country [85]. Our SDMs suggest current suitable habitats that are even larger than the known distribution of this species, probably due to some occurrence records in Colombia, which may have enlarged the interpretation of the niche range by the algorithm we used. A significant decrease of habitat suitability can be expected in future climate (Fig 2), with large areas of Amazonia becoming unsuitable. Although it is unlikely that climate change or habitat alteration could threaten this wide-ranging species, our models indicate that localized population declines of *S*. *x-signatus* may happen in the foreseeable future.

The imported invader *Eleutherodactylus johnstonei* originally occurs in the Lesser Antilles, but it has been introduced to Jamaica and Venezuela and it is currently being considered as a species in the process of expansion [85]. The SDMs we built for this species show a fragmented mosaic of suitable habitats under current conditions. The established population in the Brooklin neighborhood in São Paulo may represent a source population capable of spreading to southern areas of the Atlantic forest (Fig 3). The fact that there are no records of *E*. *johnstonei* outside urban centers in Brazil (the same is observed in other invaded areas outside its native range; [93,94, 95]) definitely bodes well for the local fauna. Because this species shows extremely high population densities in Brooklin, mitigation actions may be needed to halt the spread of *E*. *johnstonei* to other nearby locations [48]. Furthermore, similar inspection protocols to those suggested for *P*. *luteolus* could be implemented to prevent the introduction of *E*. *johnstonei* to other locations in Brazil [96, 48].

Finally, SDMs built for *Lithobates catesbeianus* showed a restricted distributional pattern of suitable habitats when compared to other studies [24, 40, 41, 55]. Aside from using distinct analytical tools, data sources, and the choice of climatic variables used, our study agrees in terms of prediction to the current SDMs and the occurrence records of *L*. *catesbeianus* [see 40]. Nevertheless, Both and colleagues [41] reported some individuals outside their optimum climatic conditions, as indicated by our models for current time. These individuals could be in the process of invading new sites, either by range expansions or due to recent translocations, not necessarily corresponding to established populations. To investigate this case, local monitoring or molecular analyses are necessary. Furthermore, our models for the current bullfrog distribution corroborate most of the expected future distribution for this invasive species in Brazil [85]. Our models mostly match those provided by Ficetola *et al*. [24] and partially match the models provided by Nori *et al*. [55]. However, we cannot discard the possibility that our models were influenced by the high number of occurrence records available for bullfrogs in North America, and also by the threshold method we used (*e*.*g*., [68]), which seems to be slightly high. To investigate this potential bias, further studies focused on multiple models based on forecast techniques (*e*.*g*., [97]) would be fruitful. Nonetheless, all cited studies (including ours) investigating potential bullfrog distribution show high statistical confidence and indicate matching patterns. In our current study, only habitats highly favorable for bullfrog occurrence in terms of suitable climates were projected into binary maps, ignoring low suitable habitats that could be used as routes for population establishments or migrations. Additionally, when dealing with highly vagile species such as *L*. *catesbeianus*, models might not be able to properly interpret potential sink habitats in a realistic fashion [82, 61]. Moreover, as previously discussed, SDM assumes only environmental variables, excluding biotic interactions [22]. Nonetheless, our SDMs suggest suitable habitats mainly in restricted areas of the southern Atlantic forest and the Pampas under current climates, and future favorable habitats in areas of the Cerrado and Caatinga (Fig 3). Although these results do not sound overly pessimistic, we suggest caution while using predicted bullfrog distributions.

### Bioacoustic interference

When an invasive amphibian species joins native species in any reproductive habitat, its vocalization may generate enough noise to disrupt the communication between native conspecifics that use similar frequency channels [46, 98]. For instance, Both *et al*. [46] reported a spectral displacement on calls of *Hypsiboas albomarginatus* when that species shared a breeding site with the invasive *Lithobates catesbeianus* in southern Brazil. Likewise, it is possible that such interference is also occurring between *Ischnocnema* sp. and *Phyllodytes luteolus*, because these species are synchronopatric in Guarujá and have advertisement calls with spectral overlap. Furthermore, invasions can also lead to mistakes in acoustic recognition between highly related species (see [99]) due to the likely similarity of calls between sister species [100, 101, 102]. The establishment of heterospecific pairs could be deleterious for those species of the genus *Leptodactylus* in Manaus because the invasive *L*. *labyrinthicus* is synchronopatric with the two other congeners [103]. Besides morphological similarities and the high phylogenetic relatedness, the high spectral overlap coupled with similar ascending modulation observed in their advertisement calls could lead to hybridization (*e*.*g*., [99]). Whether or not slight differences in temporal structure among the calls of these species are enough for species-specific recognition is a matter for further investigation (see [104, 105, 106]).

The calls of species such as *E*. *johnstonei* and *E*. *coqui* are often very loud, especially when males are in chorus [27, 48]. Therefore, invasive species may lead to a sharp drop in real estate assets; property value is expected to depreciate in areas surrounded by calling sites such as those from Brooklin neighborhood in the city of São Paulo [48].

### Trophic networks

Invasive species could pose negative impacts on local food webs by competition with and predation upon native species. *Leptodactylus labyrinthicus*, *Rhinella jimi*, and *Lithobates catesbeianus* have the potential to alter food web dynamics because they show large ontogenetic variation, feeding on algae (as tadpoles), invertebrates, and small vertebrates, including other native amphibians, as post metamorphs (*e*.*g*., [107, 108, 109, 110, 111, 112, 43, 113, 44, 114, 45]). Species with a relatively small body size could also impact ecosystem functioning. For instance, two amphibian species introduced in Hawaii (*Eleutherodactylus coqui* and *E*. *planirostris* from Puerto Rico and Cuba, respectively) live in dense populations without native predators and feed on a massive amount of invertebrate biomass [26, 2]. These two species are in turn dramatically altering the local biogeochemical cycles [2, 96]. These findings highlight the need for detailed research on how *Scinax x-signatus* and *Phyllodytes luteolus* change ecosystem functioning. Shift in trophic networks could be an additional environmental impact caused by *Eleutherodactylus johnstonei* in case it reaches undisturbed natural habitats.

Island ecosystems are generally more vulnerable and respond more rapidly to the impacts of invasive species, because islands tend to harbor less complex communities than the mainland [115]. Thus, *R*. *jimi* and *S*. *x-signatus* may be also affecting the terrestrial ecosystem of Fernando de Noronha. Islands usually show high levels of endemicity [116, 117], which makes the invasion of both *R*. *jimi* and *S*. *x-signatus* even more problematic in terms of conservation. *Rhinella jimi* is closely related to the cane-toad, *Rhinella marina* [118]; the latter is one of the top 100 most successful invasive species globally [119]. Cane-toads were introduced to Australia as a biological control agent against crop pests and disease vector insects [120, 28, 2]; the same likely reasons for the introduction of *R*. *jimi* in Fernando de Noronha. *R*. *marina* is able to avoid several types of predators [121] and is promoting the evolution of morphological and behavioral traits in some native species in Australia [35]. Thus far, we have no data on the potential impacts of *R*. *jimi* on the fauna and flora of Fernando de Noronha, but these impacts are likely similar to those observed in Australia.

### Interspecific competition

Invasive populations of *Phyllodytes luteolus* in the states of Rio de Janeiro and São Paulo are possibly affecting native species by competing for limited resources for breeding (*i*.*e*., bromeliads) [122, 123]. In the southeast, invasive *P*. *luteolus* coexists with other native species that are bromeligenous, such as *Scinax perpusillus* in Guarujá. Bromeliads can represent a limited and fundamental resource for the reproduction of these species [122, 123, 124]. Consequently, the coexistence of these frogs may be a source of spatial conflict. *P*. *luteolus* has the ability to reproduce using different species of bromeliads [122, 123] and this trait may facilitate its spread and success over other species. Moreover, the larger body size of *P*. *luteolus* [123, 125] may contribute to its success while defending its breeding site against species with smaller body size such as *S*. *perpusillus*, *Dendrophryniscus brevipollicatus*, and other bromeligenous anuran species. Furthermore, because different types of stressors could reduce amphibian immunity [126, 127], it is possible that stress caused by invasive species increases the susceptibility of native species to parasites and diseases.

### Diseases and parasites

In addition to competing and displacing local species, invasive amphibians may also disseminate parasites and diseases. For instance, the American bullfrog was recently identified as a competent carrier of the frog-killing *Bd* fungus in Brazil [128, 36, 37]. Another example is Ranavirus, which may be also spread with the international trade of bullfrogs [129]. Furthermore, some authors [77] found the parasite *Rhabdias fuelleborni* on in individuals of *R*. *jimi* from Fernando de Noronha. This parasite is not amphibian exclusive and, therefore, could infect other vertebrates from the archipelago. Biological invasions facilitating the spread of these and other pathogenic agents may explain a lot of amphibian populations declines globally [130, 131]. Although we did not screen invasive frogs for Ranavirus, the absence of *Bd* in the invasive populations of *Phyllodytes luteolus* and *Eleutherodactylus johnstonei* bodes well for the Brazilian herpetofauna. *Eleutherodactylus johnstonei*, in particular, could have brought a novel hypervirulent *Bd* strain from Central America or the Caribbean, potentially leading to epizootics and population declines such as the ones observed in Panama and Costa Rica [132].

### Biological invasions in Brazilian islands

The introduction of *Rhinella jimi* to the archipelago of Fernando de Noronha is a peculiar case of biological invasion. This population presents high rates of morphological anomalies, both in tadpoles [79] and post-metamorphics [78]. *Rhinella jimi* may be suffering either from sub-lethal environmental condition (*e*.*g*., chemical pollutants) or intrinsic handicaps such as inbreeding depression (see [78, 79]). *Rhinella jimi* has been established in Fernando de Noronha for about 100 years and these toads are thriving despite showing high prevalence of multiple types of abnormalities [78, 133]. This is another example of how invasive species can benefit from the lack of local competitors and predators in oceanic islands [78, 79].

## Conclusions

Our SDMs suggest that at least two invasive amphibians may expand their ranges in Brazil in the near future, following the accelerated pace of deforestation and global warming [134, 135, 136, 137]. These results underscore the need to further monitor populations at the edge of their invasive ranges in eastern Amazonia and around several urban areas of the Atlantic forest. To date, only the American bullfrog has been included in action plans by the Brazilian government to control expanding populations [138]. Further studies focused on acoustic interference are needed to evaluate the direct impact of the invasive species *Eleutherodactylus johnstonei* and *Phyllodytes luteolus* on native anurans breeding in southeastern Brazil, and *Leptodactylus labyrinthicus* on native species from northern Brazil. Our work underscores that biological invasions of amphibians are becoming more complex and widespread, requiring immediate attention from the government to safeguard Brazil’s biodiversity.

## Supporting information

S1 Table Invasive species analyzed and sources of occurrence records. (DOCX)

S2 Table Geographical locations used for the species distribution models. (DOCX)

S1 Fig Maps with the invasive species distribution in Brazil; black dots as natural populations and red dots as invasive populations. (A) *Eleutherodactylus johnstonei*; (B) *Lithobates catesbeianus*; (C) *Scinax x-signatus*; (D) *Rhinella jimi*; (E) *Leptodactylus labyrinthicus*; (F) *Phyllodytes luteolus*.
(DOCX)
