# Supplementary material for: Perspectives on invasive amphibians in Brazil
Source: PLoS One. 2017 Sep 22;12(9):e0184703. doi: 10.1371/journal.pone.0184703 (PMC5609743; doi:10.1371/journal.pone.0184703)
Supplement: S1 Table — (DOCX) [file pone.0184703.s001.docx]

| Table S1. Invasive species analyzed and sources of occurrence records. | |
| --- | --- |
| Taxon | Source |
| *Leptodactylus labyrinthicus* | Personal observation; Carvalho et al. 2003; Coleção Célio F. B. Haddad (CFBH), Coleção de Anfíbios DZSJRP (DZSJRP-Amphibia-adults), Coleção de Girinos DZSJRP (DZSJRP-Amphibia-tadpoles), Fonoteca Neotropical Jacques Vielliard (FNJV), Coleção de Anfíbios MBML (MBML-Anfibios), Coleção de Anfíbios do Museu de Ciências e Tecnologia da PUCRS, MCP, Coleção de Herpetofauna do Museu de Zoologia (MZUEL-Herpeto), Sistema de Informação do Programa Biota/Fapesp (SinBiota), Coleção de Anfíbios do Centro de Coleções Taxonômicas da UFMG (UFMG-AMP), Coleção de Girinos do Centro de Coleções Taxonômicas da UFMG (UFMG-GIR), Coleção Zoológica da UFMT- Setor Herpetologia-Amphibia (UFMT-A), Coleção de Anfíbios do Museu de Zoologia da UNICAMP (ZUEC-AMP) disponível na rede speciesLink (http://www.splink.org.br) em 26 de Agosto de 2016 às 16:10; GBIF.org (29th August 2016) GBIF Occurrence Download http://doi.org/10.15468/dl.10hojw |
|  |  |
| *Phyllodytes luteolus* | Personal observation; Salles and Silva-Soares, 2010; Coleção Célio F. B. Haddad (CFBH), Coleção de Anfíbios DZSJRP (DZSJRP-Amphibia-adults), Fonoteca Neotropical Jacques Vielliard (FNJV), Coleção de Anfíbios MBML (MBML-Anfibios), Coleção Zoológica da UFMT- Setor Herpetologia-Amphibia (UFMT-A), Coleção de Anfíbios do Museu de Zoologia da UNICAMP (ZUEC-AMP) disponível na rede speciesLink (http://www.splink.org.br) em 26 de Agosto de 2016 às 16:10.; GBIF.org (29th August 2016) GBIF Occurrence Download http://doi.org/10.15468/dl.553dxr |
|  |  |
| *Rhinella jimi* | Toledo & Ribeiro 2009; Coleção Célio F. B. Haddad (CFBH), Fonoteca Neotropical Jacques Vielliard (FNJV), Coleção de Anfíbios MBML (MBML-Anfibios), Coleção de Anfíbios do Museu de Ciências e Tecnologia da PUCRS, MCP, Coleção de Anfíbios do Centro de Coleções Taxonômicas da UFMG (UFMG-AMP), Coleção de Anfíbios do Museu de Zoologia da UNICAMP (ZUEC-AMP) disponível na rede speciesLink (http://www.splink.org.br) em 26 de Agosto de 2016 às 16:10.; GBIF.org (29th August 2016) GBIF Occurrence Download http://doi.org/10.15468/dl.qt6pnt |
|  |  |
| *Scinax x-signatus* | Toledo & Ribeiro 2009; Coleção Célio F. B. Haddad (CFBH), Coleção de Anfíbios DZSJRP (DZSJRP-Amphibia-adults), Fonoteca Neotropical Jacques Vielliard (FNJV), Coleção de Anfíbios MBML (MBML-Anfibios), Coleção de Anfíbios do Museu de Ciências e Tecnologia da PUCRS, MCP, Sistema de Informação do Programa Biota/Fapesp (SinBiota), Coleção de Anfíbios do Centro de Coleções Taxonômicas da UFMG (UFMG-AMP), Coleção de Girinos do Centro de Coleções Taxonômicas da UFMG (UFMG-GIR), Coleção de Anfíbios do Museu de Zoologia da UNICAMP (ZUEC-AMP) disponível na rede speciesLink (http://www.splink.org.br) em 26 de Agosto de 2016 às 16:10.; GBIF.org (29th August 2016) GBIF Occurrence Download http://doi.org/10.15468/dl.ymwncr |
|  |  |
| *Eleutherodactylus johnstonei* | Melo et al., 2014; Fonoteca Neotropical Jacques Vielliard (FNJV); Coleção de Anfíbios do Museu de Zoologia “prof. Adão José Cardoso” da UNICAMP (ZUEC-AMP) disponível na rede speciesLink (http://www.splink.org.br) em 26 de Agosto de 2016 às 16:10; GBIF.org (29th August 2016) GBIF Occurrence Download http://doi.org/10.15468/dl.u9hgvs |
|  |  |
| *Lithobates catesbeianus* | Coleção Célio F. B. Haddad (CFBH), Coleção de Anfíbios DZSJRP (DZSJRP-Amphibia-adults), Coleção de Girinos DZSJRP (DZSJRP-Amphibia-tadpoles), Coleção de Anfíbios MBML (MBML-Anfibios), Coleção de Anfíbios do Museu de Ciências e Tecnologia da PUCRS, MCP, Sistema de Informação do Programa Biota/Fapesp (SinBiota), Coleção de Anfíbios do Centro de Coleções Taxonômicas da UFMG (UFMG-AMP), Coleção de Anfíbios do Museu de Zoologia da UNICAMP (ZUEC-AMP) disponível na rede speciesLink (http://www.splink.org.br) em 26 de Agosto de 2016 às 16:00; GBIF.org (29th August 2016) GBIF Occurrence Download http://doi.org/10.15468/dl.idbljh |
