# Supplementary material for: Perspectives on invasive amphibians in Brazil
Source: PLoS One. 2017 Sep 22;12(9):e0184703. doi: 10.1371/journal.pone.0184703 (PMC5609743; doi:10.1371/journal.pone.0184703)
Supplement: S2 Table — (DOCX) [file pone.0184703.s002.docx]

Table S2. Geographical locations used for the species distribution models.

| **Taxon** | **Longitude** | **Latitude** |
| --- | --- | --- |
| *Leptodactylus labyrinthicus* | -49.5392 | -21.0786 |
| *Leptodactylus labyrinthicus* | -49.5192 | -21.0811 |
| *Leptodactylus labyrinthicus* | -49.5203 | -21.0744 |
| *Leptodactylus labyrinthicus* | -49.5206 | -21.0744 |
| *Leptodactylus labyrinthicus* | -49.5189 | -21.0736 |
| *Leptodactylus labyrinthicus* | -48 | -22.25 |
| *Leptodactylus labyrinthicus* | -45.625 | -23.0264 |
| *Leptodactylus labyrinthicus* | -45.5225 | -22.8708 |
| *Leptodactylus labyrinthicus* | -45.0497 | -22.5558 |
| *Leptodactylus labyrinthicus* | -45.7478 | -23.0094 |
| *Leptodactylus labyrinthicus* | -49.3794 | -20.8197 |
| *Leptodactylus labyrinthicus* | -49.3872 | -20.6569 |
| *Leptodactylus labyrinthicus* | -50.4492 | -21.5597 |
| *Leptodactylus labyrinthicus* | -49.9728 | -20.4228 |
| *Leptodactylus labyrinthicus* | -51.3889 | -22.1256 |
| *Leptodactylus labyrinthicus* | -51.5531 | -22.0061 |
| *Leptodactylus labyrinthicus* | -49.2047 | -20.355 |
| *Leptodactylus labyrinthicus* | -49.2019 | -20.3561 |
| *Leptodactylus labyrinthicus* | -49.1933 | -20.3633 |
| *Leptodactylus labyrinthicus* | -49.2019 | -20.3672 |
| *Leptodactylus labyrinthicus* | -49.5431 | -21.0761 |
| *Leptodactylus labyrinthicus* | -49.2019 | -21.3567 |
| *Leptodactylus labyrinthicus* | -49.2039 | -20.355 |
| *Leptodactylus labyrinthicus* | -49.2006 | -20.3686 |
| *Leptodactylus labyrinthicus* | -49.2756 | -20.355 |
| *Leptodactylus labyrinthicus* | -49.1939 | -20.3639 |
| *Leptodactylus labyrinthicus* | -49.1986 | -20.3633 |
| *Leptodactylus labyrinthicus* | -49.4997 | -21.0331 |
| *Leptodactylus labyrinthicus* | -50.4844 | -20.1967 |
| *Leptodactylus labyrinthicus* | -49.195 | -20.3417 |
| *Leptodactylus labyrinthicus* | -50.4339 | -20.0036 |
| *Leptodactylus labyrinthicus* | -48.7517 | -20.6372 |
| *Leptodactylus labyrinthicus* | -56.7233 | -18.731 |
| *Leptodactylus labyrinthicus* | -58.3104 | -14.4346 |
| *Leptodactylus labyrinthicus* | -55.5396 | -15.1071 |
| *Leptodactylus labyrinthicus* | -54.606 | -17.3359 |
| *Leptodactylus labyrinthicus* | -58.8619 | -15.3173 |
| *Leptodactylus labyrinthicus* | -57.6918 | -15.0433 |
| *Leptodactylus labyrinthicus* | -54.436 | -17.646 |
| *Leptodactylus labyrinthicus* | -57.8369 | -16.5165 |
| *Leptodactylus labyrinthicus* | -57.995 | -12.4251 |
| *Leptodactylus labyrinthicus* | -56.1657 | -13.0377 |
| *Leptodactylus labyrinthicus* | -58.4395 | -15.0853 |
| *Leptodactylus labyrinthicus* | -59.3525 | -15.2588 |
| *Leptodactylus labyrinthicus* | -38.901 | -4.4719 |
| *Leptodactylus labyrinthicus* | -57.6248 | -11.0586 |
| *Leptodactylus labyrinthicus* | -55.8894 | -15.48 |
| *Leptodactylus labyrinthicus* | -56.5281 | -15.8315 |
| *Leptodactylus labyrinthicus* | -58.4572 | -15.2558 |
| *Leptodactylus labyrinthicus* | -56.255 | -15.5709 |
| *Leptodactylus labyrinthicus* | -56.8171 | -14.0943 |
| *Leptodactylus labyrinthicus* | -59.983 | -15.1875 |
| *Leptodactylus labyrinthicus* | -55.4364 | -16.4825 |
| *Leptodactylus labyrinthicus* | -54.7664 | -12.9931 |
| *Leptodactylus labyrinthicus* | -59.4542 | -14.2059 |
| *Leptodactylus labyrinthicus* | -55.9402 | -13.0556 |
| *Leptodactylus labyrinthicus* | -58.58 | -15.1 |
| *Leptodactylus labyrinthicus* | -56.05 | -15.6 |
| *Leptodactylus labyrinthicus* | -56.0501 | -15.6001 |
| *Leptodactylus labyrinthicus* | -59.0268 | -14.9798 |
| *Leptodactylus labyrinthicus* | -56.0502 | -15.6002 |
| *Leptodactylus labyrinthicus* | -59.4517 | -13.971 |
| *Leptodactylus labyrinthicus* | -59.4545 | -14.2076 |
| *Leptodactylus labyrinthicus* | -43.7447 | -19.5142 |
| *Leptodactylus labyrinthicus* | -51.5328 | -21.4825 |
| *Leptodactylus labyrinthicus* | -51.4336 | -21.5147 |
| *Leptodactylus labyrinthicus* | -49.3872 | -20.6567 |
| *Leptodactylus labyrinthicus* | -50.4338 | -20.0036 |
| *Leptodactylus labyrinthicus* | -48.7516 | -20.6372 |
| *Leptodactylus labyrinthicus* | -56.0967 | -15.5961 |
| *Leptodactylus labyrinthicus* | -45.2417 | -18.2064 |
| *Leptodactylus labyrinthicus* | -43.8897 | -19.6272 |
| *Leptodactylus labyrinthicus* | -56.4819 | -21.1211 |
| *Leptodactylus labyrinthicus* | -50.8923 | -20.1782 |
| *Leptodactylus labyrinthicus* | -49.5 | -21.0331 |
| *Leptodactylus labyrinthicus* | -49.1853 | -20.3671 |
| *Leptodactylus labyrinthicus* | -49.75 | -23.1166 |
| *Leptodactylus labyrinthicus* | -49.5434 | -21.0767 |
| *Leptodactylus labyrinthicus* | -50.485 | -20.1972 |
| *Leptodactylus labyrinthicus* | -49.1977 | -20.3411 |
| *Leptodactylus labyrinthicus* | -45.5613 | -23.0311 |
| *Leptodactylus labyrinthicus* | -49.2526 | -20.3792 |
| *Leptodactylus labyrinthicus* | -49.2388 | -20.3641 |
| *Leptodactylus labyrinthicus* | -49.1982 | -20.3578 |
| *Leptodactylus labyrinthicus* | -43.7447 | -19.5136 |
| *Leptodactylus labyrinthicus* | -45.31 | -23.22 |
| *Leptodactylus labyrinthicus* | -48.1197 | -22.2803 |
| *Leptodactylus labyrinthicus* | -47.06 | -22.9 |
| *Leptodactylus labyrinthicus* | -46.3881 | -20.8636 |
| *Leptodactylus labyrinthicus* | -49.1178 | -5.36861 |
| *Leptodactylus labyrinthicus* | -46.5614 | -21.7881 |
| *Leptodactylus labyrinthicus* | -42.1392 | -19.7897 |
| *Leptodactylus labyrinthicus* | -36.9406 | -8.49694 |
| *Leptodactylus labyrinthicus* | -43.7142 | -19.1692 |
| *Leptodactylus labyrinthicus* | -42.6992 | -9.01556 |
| *Leptodactylus labyrinthicus* | -43.8467 | -19.9858 |
| *Leptodactylus labyrinthicus* | -49.5289 | -11.7969 |
| *Leptodactylus labyrinthicus* | -47.6024 | -6.94822 |
| *Leptodactylus labyrinthicus* | -60.0286 | -3.27167 |
| *Leptodactylus labyrinthicus* | -60.0333 | -3.12611 |
| *Leptodactylus labyrinthicus* | -60.0322 | -3.11389 |
| *Leptodactylus labyrinthicus* | -63.9581 | -7.31917 |
| *Leptodactylus labyrinthicus* | -54.95 | -2.51639 |
| *Leptodactylus labyrinthicus* | -62.0619 | -11.2625 |
| *Leptodactylus labyrinthicus* | -35.6333 | -5.63333 |
| *Leptodactylus labyrinthicus* | -49.2667 | -23.1833 |
| *Leptodactylus labyrinthicus* | -56.0583 | -22.6333 |
| *Leptodactylus labyrinthicus* | -56.0583 | -22.6333 |
| *Leptodactylus labyrinthicus* | -63.1579 | -17.7889 |
| *Leptodactylus labyrinthicus* | -57.6333 | -19.15 |
| *Leptodactylus labyrinthicus* | -56.779 | -26.074 |
| *Leptodactylus labyrinthicus* | -47.8 | -21.1667 |
| *Leptodactylus labyrinthicus* | -44.5667 | -17.9 |
| *Leptodactylus labyrinthicus* | -63.7406 | 10.2428 |
| *Leptodactylus labyrinthicus* | -54.9546 | -26.6642 |
| *Leptodactylus labyrinthicus* | -47.7 | -21.6 |
| *Leptodactylus labyrinthicus* | -43.9333 | -19.9167 |
| *Leptodactylus labyrinthicus* | -35.259 | -6.036 |
| *Leptodactylus labyrinthicus* | -56.51 | -23.43 |
| *Leptodactylus labyrinthicus* | -46.7658 | -12.9008 |
| *Leptodactylus labyrinthicus* | -58.8333 | -23.5 |
| *Leptodactylus labyrinthicus* | -55.78 | -15.42 |
| *Leptodactylus labyrinthicus* | -79.2667 | 0.1167 |
| *Leptodactylus labyrinthicus* | -50.15 | -25.0833 |
| *Leptodactylus labyrinthicus* | -60.6259 | -19.2822 |
| *Leptodactylus labyrinthicus* | -57.59 | -22.76 |
| *Leptodactylus labyrinthicus* | -54.985 | -19.412 |
| *Leptodactylus labyrinthicus* | -46.6167 | -23.5333 |
| *Leptodactylus labyrinthicus* | -46.199 | -23.766 |
| *Leptodactylus labyrinthicus* | -50.4167 | -22.6667 |
| *Leptodactylus labyrinthicus* | -54.84 | -27.1173 |
| *Leptodactylus labyrinthicus* | -57.05 | -26.02 |
| *Leptodactylus labyrinthicus* | -63.9167 | 10.25 |
| *Leptodactylus labyrinthicus* | -56.44 | -21.163 |
| *Leptodactylus labyrinthicus* | -75.9 | -9.55 |
| *Leptodactylus labyrinthicus* | -43.2333 | -22.9 |
| *Leptodactylus labyrinthicus* | -46.3882 | -20.8635 |
| *Leptodactylus labyrinthicus* | -46.5619 | -21.7854 |
| *Leptodactylus labyrinthicus* | -47.5651 | -22.4149 |
| *Leptodactylus labyrinthicus* | -49.8512 | -6.53209 |
| *Leptodactylus labyrinthicus* | -47.3336 | -22.7378 |
| *Leptodactylus labyrinthicus* | -50.4552 | -18.4476 |
| *Leptodactylus labyrinthicus* | -47.817 | -22.2567 |
| *Leptodactylus labyrinthicus* | -41.3907 | -12.5638 |
| *Leptodactylus labyrinthicus* | -42.9417 | -16.4391 |
| *Leptodactylus labyrinthicus* | -54.7192 | -28.1437 |
| *Leptodactylus labyrinthicus* | -47.4884 | -21.6867 |
| *Leptodactylus labyrinthicus* | -47.4779 | -20.2541 |
| *Leptodactylus labyrinthicus* | -50.4188 | -22.6604 |
| *Leptodactylus labyrinthicus* | -49.059 | -22.3145 |
| *Leptodactylus labyrinthicus* | -48.4138 | -23.4948 |
| *Leptodactylus labyrinthicus* | -46.9433 | -22.368 |
| *Leptodactylus labyrinthicus* | -44.9835 | -21.7222 |
| *Leptodactylus labyrinthicus* | -47.9606 | -6.85314 |
| *Leptodactylus labyrinthicus* | -47.7013 | -21.5511 |
| *Leptodactylus labyrinthicus* | -40.0838 | -13.8591 |
| *Leptodactylus labyrinthicus* | -47.0619 | -22.909 |
| *Leptodactylus labyrinthicus* | -47.4509 | -19.8653 |
| *Leptodactylus labyrinthicus* | -48.4467 | -22.2697 |
| *Leptodactylus labyrinthicus* | -48.5306 | -22.3027 |
| *Leptodactylus labyrinthicus* | -53.4153 | -15.3414 |
| *Leptodactylus labyrinthicus* | -55.5142 | -15.8231 |
| *Leptodactylus labyrinthicus* | -52.6646 | -27.1449 |
| *Leptodactylus labyrinthicus* | -47.4047 | -16.0106 |
| *Leptodactylus labyrinthicus* | -51.2472 | -23.4522 |
| *Leptodactylus labyrinthicus* | -49.4343 | -20.3915 |
| *Leptodactylus labyrinthicus* | -45.7478 | -23.0094 |
| *Leptodactylus labyrinthicus* | -45.0497 | -22.5558 |
| *Leptodactylus labyrinthicus* | -45.5225 | -22.8708 |
| *Leptodactylus labyrinthicus* | -49.3794 | -20.8197 |
| *Leptodactylus labyrinthicus* | -49.3872 | -20.6569 |
| *Leptodactylus labyrinthicus* | -51.4336 | -21.5147 |
| *Leptodactylus labyrinthicus* | -51.5328 | -21.4825 |
| *Leptodactylus labyrinthicus* | -51.3889 | -22.1256 |
| *Leptodactylus labyrinthicus* | -49.9728 | -20.4228 |
| *Leptodactylus labyrinthicus* | -51.5531 | -22.0061 |
| *Leptodactylus labyrinthicus* | -49.2047 | -20.355 |
| *Leptodactylus labyrinthicus* | -49.2019 | -20.3561 |
| *Leptodactylus labyrinthicus* | -49.1933 | -20.3633 |
| *Leptodactylus labyrinthicus* | -49.2019 | -20.3672 |
| *Leptodactylus labyrinthicus* | -49.5431 | -21.0761 |
| *Leptodactylus labyrinthicus* | -49.2039 | -20.355 |
| *Leptodactylus labyrinthicus* | -49.2019 | -21.3567 |
| *Leptodactylus labyrinthicus* | -49.2006 | -20.3686 |
| *Leptodactylus labyrinthicus* | -49.1939 | -20.3639 |
| *Leptodactylus labyrinthicus* | -49.1986 | -20.3633 |
| *Leptodactylus labyrinthicus* | -49.4997 | -21.0331 |
| *Leptodactylus labyrinthicus* | -50.4844 | -20.1967 |
| *Leptodactylus labyrinthicus* | -50.4339 | -20.0036 |
| *Leptodactylus labyrinthicus* | -48.7517 | -20.6372 |
| *Leptodactylus labyrinthicus* | -48.4628 | -23.3446 |
| *Leptodactylus labyrinthicus* | -50.8922 | -20.1783 |
| *Leptodactylus labyrinthicus* | -49.5192 | -21.0811 |
| *Leptodactylus labyrinthicus* | -49.5203 | -21.0744 |
| *Leptodactylus labyrinthicus* | -49.5206 | -21.0744 |
| *Leptodactylus labyrinthicus* | -49.5189 | -21.0736 |
| *Leptodactylus labyrinthicus* | -45.9333 | -23.0667 |
| *Leptodactylus labyrinthicus* | -47.0692 | -22.8211 |
| *Leptodactylus labyrinthicus* | -47.0697 | -22.8169 |
| *Leptodactylus labyrinthicus* | -63.9581 | -7.31917 |
| *Leptodactylus labyrinthicus* | -54.95 | -2.51639 |
| *Leptodactylus labyrinthicus* | -62.0619 | -11.2625 |
| *Leptodactylus labyrinthicus* | -60.0286 | -3.105 |
| *Leptodactylus labyrinthicus* | -60.0322 | -3.11389 |
| *Leptodactylus labyrinthicus* | -46.3866 | -20.8609 |
| *Leptodactylus labyrinthicus* | -46.5618 | -21.7849 |
| *Leptodactylus labyrinthicus* | -47.5649 | -22.4148 |
| *Leptodactylus labyrinthicus* | -47.3337 | -22.7369 |
| *Leptodactylus labyrinthicus* | -50.4551 | -18.4477 |
| *Leptodactylus labyrinthicus* | -41.3899 | -12.5618 |
| *Leptodactylus labyrinthicus* | -42.8895 | -16.5554 |
| *Leptodactylus labyrinthicus* | -54.7302 | -28.1471 |
| *Leptodactylus labyrinthicus* | -47.4887 | -21.6868 |
| *Leptodactylus labyrinthicus* | -47.481 | -20.2584 |
| *Leptodactylus labyrinthicus* | -49.0584 | -22.3141 |
| *Leptodactylus labyrinthicus* | -48.4138 | -23.4948 |
| *Leptodactylus labyrinthicus* | -46.9441 | -22.3686 |
| *Leptodactylus labyrinthicus* | -47.4477 | -19.8802 |
| *Leptodactylus labyrinthicus* | -47.0626 | -22.9099 |
| *Leptodactylus labyrinthicus* | -40.0838 | -13.8591 |
| *Leptodactylus labyrinthicus* | -47.6987 | -21.5528 |
| *Leptodactylus labyrinthicus* | -49.9676 | -9.31047 |
| *Leptodactylus labyrinthicus* | -38.5258 | -3.72817 |
| *Leptodactylus labyrinthicus* | -35.4241 | -5.64138 |
| *Leptodactylus labyrinthicus* | -46.7542 | -10.5463 |
| *Leptodactylus labyrinthicus* | -62.0619 | -11.2625 |
| *Leptodactylus labyrinthicus* | -54.5167 | -25.3 |
| *Leptodactylus labyrinthicus* | -49.9615 | -9.31058 |
| *Leptodactylus labyrinthicus* | -56.77 | -26.07 |
| *Leptodactylus labyrinthicus* | -57.3776 | -22.716 |
| *Leptodactylus labyrinthicus* | -56.05 | -22.63 |
| *Leptodactylus labyrinthicus* | -35.208 | -6.09154 |
| *Leptodactylus labyrinthicus* | -63.74 | 10.24 |
| *Leptodactylus labyrinthicus* | -46.15 | -23.85 |
| *Leptodactylus labyrinthicus* | -35.1859 | -6.11433 |
| *Leptodactylus labyrinthicus* | -46.7574 | -10.5565 |
| *Leptodactylus labyrinthicus* | -63.91 | 10.25 |
| *Leptodactylus labyrinthicus* | -53.4153 | -15.3414 |
| *Leptodactylus labyrinthicus* | -55.5142 | -15.8231 |
| *Leptodactylus labyrinthicus* | -47.4047 | -16.0106 |
| *Leptodactylus labyrinthicus* | -51.2472 | -23.4522 |
| *Leptodactylus labyrinthicus* | -35.1333 | -6.06667 |
| *Phyllodytes luteolus* | -39.2142 | -16.2307 |
| *Phyllodytes luteolus* | -40.4067 | -19.9325 |
| *Phyllodytes luteolus* | -39.8589 | -18.7161 |
| *Phyllodytes luteolus* | -40.493 | -20.657 |
| *Phyllodytes luteolus* | -40.0722 | -19.3911 |
| *Phyllodytes luteolus* | -39.7322 | -18.5933 |
| *Phyllodytes luteolus* | -40.3244 | -20.4228 |
| *Phyllodytes luteolus* | -40.8339 | -21.0111 |
| *Phyllodytes luteolus* | -40.6003 | -19.9364 |
| *Phyllodytes luteolus* | -40.4057 | -20.5821 |
| *Phyllodytes luteolus* | -40.503 | -20.65 |
| *Phyllodytes luteolus* | -40.2922 | -20.3297 |
| *Phyllodytes luteolus* | -34.891 | -7.994 |
| *Phyllodytes luteolus* | -35.3981 | -9.04528 |
| *Phyllodytes luteolus* | -40.0719 | -19.3911 |
| *Phyllodytes luteolus* | -40.0978 | -19.1969 |
| *Phyllodytes luteolus* | -43.458 | -22.964 |
| *Phyllodytes luteolus* | -46.247 | -23.89 |
| *Phyllodytes luteolus* | -46.237 | -23.814 |
| *Phyllodytes luteolus* | -47.5644 | -22.4147 |
| *Phyllodytes luteolus* | -40.35 | -20.407 |
| *Phyllodytes luteolus* | -40.521 | -20.655 |
| *Phyllodytes luteolus* | -40.2833 | -20.3333 |
| *Phyllodytes luteolus* | -43.2333 | -22.9 |
| *Phyllodytes luteolus* | -40.5223 | -20.6802 |
| *Phyllodytes luteolus* | -34.9017 | -7.82936 |
| *Phyllodytes luteolus* | -39.7366 | -18.5888 |
| *Phyllodytes luteolus* | -40.2958 | -20.2976 |
| *Phyllodytes luteolus* | -40.1862 | -19.0984 |
| *Phyllodytes luteolus* | -40.0647 | -19.3951 |
| *Phyllodytes luteolus* | -40.8557 | -21.0358 |
| *Phyllodytes luteolus* | -40.0838 | -13.8591 |
| *Phyllodytes luteolus* | -40.3195 | -20.3513 |
| *Phyllodytes luteolus* | -40.4975 | -20.6667 |
| *Phyllodytes luteolus* | -40.4975 | -20.6669 |
| *Phyllodytes luteolus* | -34.8808 | -8.05417 |
| *Phyllodytes luteolus* | -39.0753 | -15.2933 |
| *Phyllodytes luteolus* | -40.8244 | -21.0433 |
| *Phyllodytes luteolus* | -40.511 | -20.658 |
| *Phyllodytes luteolus* | -40.0978 | -19.1969 |
| *Phyllodytes luteolus* | -40.3378 | -20.3194 |
| *Phyllodytes luteolus* | -39.7322 | -18.5933 |
| *Phyllodytes luteolus* | -34.9064 | -7.83417 |
| *Phyllodytes luteolus* | -40.0722 | -19.3911 |
| *Phyllodytes luteolus* | -46.25 | -23.99 |
| *Phyllodytes luteolus* | -40.6003 | -19.9356 |
| *Phyllodytes luteolus* | -35.3983 | -9.045 |
| *Phyllodytes luteolus* | -43.471 | -23.0321 |
| *Phyllodytes luteolus* | -46.1717 | -23.8892 |
| *Phyllodytes luteolus* | -46.1656 | -23.8994 |
| *Phyllodytes luteolus* | -47.5644 | -22.4147 |
| *Phyllodytes luteolus* | -40.5147 | -20.6766 |
| *Phyllodytes luteolus* | -34.9016 | -7.82922 |
| *Phyllodytes luteolus* | -39.7506 | -18.6027 |
| *Phyllodytes luteolus* | -40.3061 | -20.3023 |
| *Phyllodytes luteolus* | -40.1862 | -19.0986 |
| *Phyllodytes luteolus* | -40.0653 | -19.3946 |
| *Phyllodytes luteolus* | -40.8373 | -21.0457 |
| *Phyllodytes luteolus* | -40.0838 | -13.8591 |
| *Phyllodytes luteolus* | -40.317 | -20.3532 |
| *Phyllodytes luteolus* | -40.5 | -20.6667 |
| *Rhinella jimi* | -32.405 | -3.84 |
| *Rhinella jimi* | -32.4092 | -3.845 |
| *Rhinella jimi* | -32.4314 | -3.84861 |
| *Rhinella jimi* | -32.4336 | -3.85111 |
| *Rhinella jimi* | -32.4203 | -3.84806 |
| *Rhinella jimi* | -36.8219 | -10.2147 |
| *Rhinella jimi* | -34.903 | -7.739 |
| *Rhinella jimi* | -40.9164 | -3.84583 |
| *Rhinella jimi* | -43.1957 | -13.3254 |
| *Rhinella jimi* | -44.4433 | -3.32944 |
| *Rhinella jimi* | -42.8008 | -2.74417 |
| *Rhinella jimi* | -32.4167 | -3.85 |
| *Rhinella jimi* | -34.987 | -8.064 |
| *Rhinella jimi* | -35.8833 | -7.21667 |
| *Rhinella jimi* | -35.4563 | -9.26152 |
| *Rhinella jimi* | -32.4281 | -3.85801 |
| *Rhinella jimi* | -38.9647 | -12.2597 |
| *Rhinella jimi* | -38.4409 | -12.9166 |
| *Rhinella jimi* | -41.0192 | -3.8692 |
| *Rhinella jimi* | -36.3508 | -9.78313 |
| *Rhinella jimi* | -40.4334 | -13.4378 |
| *Rhinella jimi* | -36.8372 | -10.2122 |
| *Rhinella jimi* | -34.897 | -7.739 |
| *Rhinella jimi* | -36.5695 | -8.36123 |
| *Rhinella jimi* | -42.4864 | -14.0688 |
| *Rhinella jimi* | -40.0838 | -13.8591 |
| *Rhinella jimi* | -39.8579 | -14.233 |
| *Rhinella jimi* | -35.198 | -6.251 |
| *Rhinella jimi* | -44.3517 | -3.39948 |
| *Rhinella jimi* | -42.8239 | -2.75911 |
| *Rhinella jimi* | -37.6812 | -11.8096 |
| *Rhinella jimi* | -39.4849 | -13.744 |
| *Rhinella jimi* | -38.2976 | -12.531 |
| *Rhinella jimi* | -38.9508 | -12.435 |
| *Rhinella jimi* | -41.3705 | -13.0057 |
| *Rhinella jimi* | -41.5612 | -12.5607 |
| *Rhinella jimi* | -41.999 | -16.9776 |
| *Rhinella jimi* | -41.2407 | -15.3041 |
| *Rhinella jimi* | -40.8779 | -14.5158 |
| *Rhinella jimi* | -40.9681 | -12.9836 |
| *Rhinella jimi* | -42.6991 | -9.01371 |
| *Rhinella jimi* | -43.0253 | -7.60033 |
| *Rhinella jimi* | -41.9054 | -7.23192 |
| *Rhinella jimi* | -42.4615 | -5.03927 |
| *Rhinella jimi* | -38.2164 | -9.40606 |
| *Rhinella jimi* | -43.136 | -15.5362 |
| *Rhinella jimi* | -43.713 | -14.9209 |
| *Rhinella jimi* | -43.4112 | -13.2514 |
| *Rhinella jimi* | -43.2134 | -11.959 |
| *Rhinella jimi* | -41.4693 | -11.6918 |
| *Rhinella jimi* | -40.4727 | -11.3216 |
| *Rhinella jimi* | -40.5534 | -11.4597 |
| *Rhinella jimi* | -39.382 | -11.8062 |
| *Rhinella jimi* | -39.4797 | -9.83473 |
| *Rhinella jimi* | -39.6102 | -8.39945 |
| *Rhinella jimi* | -40.2935 | -6.00769 |
| *Rhinella jimi* | -32.4092 | -3.845 |
| *Rhinella jimi* | -35.4563 | -9.26152 |
| *Rhinella jimi* | -32.4297 | -3.85763 |
| *Rhinella jimi* | -38.9642 | -12.2592 |
| *Rhinella jimi* | -38.472 | -12.9685 |
| *Rhinella jimi* | -40.9029 | -3.73688 |
| *Rhinella jimi* | -36.3509 | -9.78298 |
| *Rhinella jimi* | -40.4336 | -13.438 |
| *Rhinella jimi* | -36.8373 | -10.2123 |
| *Rhinella jimi* | -34.8373 | -7.75408 |
| *Rhinella jimi* | -36.5631 | -8.35989 |
| *Rhinella jimi* | -42.4859 | -14.0649 |
| *Rhinella jimi* | -40.0838 | -13.8591 |
| *Rhinella jimi* | -48.6386 | -11.0363 |
| *Rhinella jimi* | -39.8558 | -14.233 |
| *Rhinella jimi* | -35.1005 | -6.22562 |
| *Rhinella jimi* | -44.3518 | -3.39945 |
| *Rhinella jimi* | -42.8243 | -2.75929 |
| *Rhinella jimi* | -37.6814 | -11.8092 |
| *Rhinella jimi* | -39.4845 | -13.7437 |
| *Rhinella jimi* | -38.3018 | -12.5317 |
| *Rhinella jimi* | -38.9508 | -12.435 |
| *Rhinella jimi* | -41.3712 | -13.0088 |
| *Rhinella jimi* | -41.5612 | -12.5607 |
| *Rhinella jimi* | -42.0633 | -16.8488 |
| *Rhinella jimi* | -41.2353 | -15.5055 |
| *Rhinella jimi* | -41.1369 | -14.6161 |
| *Rhinella jimi* | -40.9681 | -12.9836 |
| *Rhinella jimi* | -42.692 | -9.01061 |
| *Rhinella jimi* | -43.0889 | -7.49434 |
| *Rhinella jimi* | -41.8799 | -7.23683 |
| *Rhinella jimi* | -42.4615 | -5.03927 |
| *Rhinella jimi* | -38.2164 | -9.40613 |
| *Rhinella jimi* | -43.7142 | -14.9196 |
| *Rhinella jimi* | -43.4142 | -13.2675 |
| *Rhinella jimi* | -41.4706 | -11.6921 |
| *Rhinella jimi* | -40.5528 | -11.4586 |
| *Rhinella jimi* | -39.3809 | -11.8074 |
| *Rhinella jimi* | -39.4797 | -9.83439 |
| *Rhinella jimi* | -39.603 | -8.61529 |
| *Rhinella jimi* | -40.2935 | -6.00769 |
| *Scinax x-signatus* | -49.4453 | -20.9181 |
| *Scinax x-signatus* | -49.5211 | -20.8192 |
| *Scinax x-signatus* | -49.6883 | -21.0528 |
| *Scinax x-signatus* | -49.2203 | -20.795 |
| *Scinax x-signatus* | -49.3794 | -20.8197 |
| *Scinax x-signatus* | -51.1092 | -20.6383 |
| *Scinax x-signatus* | -49.4961 | -21.0158 |
| *Scinax x-signatus* | -49.7142 | -20.7725 |
| *Scinax x-signatus* | -40.622 | -20.74 |
| *Scinax x-signatus* | -49.1994 | -20.2678 |
| *Scinax x-signatus* | -49.5431 | -21.0761 |
| *Scinax x-signatus* | -54.5464 | -21.8019 |
| *Scinax x-signatus* | -50.5547 | -20.6856 |
| *Scinax x-signatus* | -52.1686 | -21.7614 |
| *Scinax x-signatus* | -49.4853 | -21.3411 |
| *Scinax x-signatus* | -53.1292 | -18.5439 |
| *Scinax x-signatus* | -56.6228 | -16.2567 |
| *Scinax x-signatus* | -63.0208 | -7.5061 |
| *Scinax x-signatus* | -56.097 | -15.5961 |
| *Scinax x-signatus* | -45.2417 | -18.2064 |
| *Scinax x-signatus* | -40.4983 | -9.41194 |
| *Scinax x-signatus* | -39.9092 | -8.99056 |
| *Scinax x-signatus* | -32.4092 | -3.845 |
| *Scinax x-signatus* | -73.2009 | 6.09662 |
| *Scinax x-signatus* | -73.8393 | 9.10688 |
| *Scinax x-signatus* | -75.1937 | 4.4329 |
| *Scinax x-signatus* | -59.3787 | -26.3199 |
| *Scinax x-signatus* | -59.7914 | -3.11333 |
| *Scinax x-signatus* | -74.6453 | 4.2021 |
| *Scinax x-signatus* | -74.933 | 4.70381 |
| *Scinax x-signatus* | -74.8982 | 5.29346 |
| *Scinax x-signatus* | -75.4179 | 6.96399 |
| *Scinax x-signatus* | -40.45 | -13.4333 |
| *Scinax x-signatus* | -43.525 | -22.983 |
| *Scinax x-signatus* | -43.35 | -22.9167 |
| *Scinax x-signatus* | -49.2961 | -26.1936 |
| *Scinax x-signatus* | -75.5943 | 3.6455 |
| *Scinax x-signatus* | -48.4564 | -1.45 |
| *Scinax x-signatus* | -57.8589 | 5.6819 |
| *Scinax x-signatus* | -60.4833 | 7.3666 |
| *Scinax x-signatus* | -75.56 | 3.78 |
| *Scinax x-signatus* | -59.3211 | 2.4753 |
| *Scinax x-signatus* | -57.8589 | 5.68194 |
| *Scinax x-signatus* | -73.2034 | 6.0985 |
| *Scinax x-signatus* | -59.1833 | -23.2 |
| *Scinax x-signatus* | -75.4397 | 7.16312 |
| *Scinax x-signatus* | -74.8134 | 6.20858 |
| *Scinax x-signatus* | -48.9796 | -27.6833 |
| *Scinax x-signatus* | -74.608 | 5.98376 |
| *Scinax x-signatus* | -74.8473 | 6.22342 |
| *Scinax x-signatus* | -74.8171 | 6.21914 |
| *Scinax x-signatus* | -74.6724 | 4.20209 |
| *Scinax x-signatus* | -74.3242 | 5.8562 |
| *Scinax x-signatus* | -74.8391 | 6.21861 |
| *Scinax x-signatus* | -74.6475 | 4.207 |
| *Scinax x-signatus* | -48.9542 | -27.7417 |
| *Scinax x-signatus* | -59.524 | 2.825 |
| *Scinax x-signatus* | -75.4724 | 5.1925 |
| *Scinax x-signatus* | -73.1817 | 7.52461 |
| *Scinax x-signatus* | -75.2833 | 8.4 |
| *Scinax x-signatus* | -73.1173 | 6.04807 |
| *Scinax x-signatus* | -58.9442 | -26.1814 |
| *Scinax x-signatus* | -74.9833 | 8.3 |
| *Scinax x-signatus* | -75.8833 | 8.4 |
| *Scinax x-signatus* | -73.2817 | 5.12778 |
| *Scinax x-signatus* | -73.48 | 4.26 |
| *Scinax x-signatus* | -71.9666 | 6.69287 |
| *Scinax x-signatus* | -73.595 | 7.3761 |
| *Scinax x-signatus* | -73.8393 | 9.106875 |
| *Scinax x-signatus* | -59.3787 | -26.3199 |
| *Scinax x-signatus* | -59.7914 | -3.11333 |
| *Scinax x-signatus* | -75.4179 | 6.963991 |
| *Scinax x-signatus* | -40.45 | -13.4333 |
| *Scinax x-signatus* | -43.48 | -23.03 |
| *Scinax x-signatus* | -48.4564 | -1.45 |
| *Scinax x-signatus* | -57.8589 | 5.681944 |
| *Scinax x-signatus* | -75.4397 | 7.163124 |
| *Scinax x-signatus* | -74.8134 | 6.208583 |
| *Scinax x-signatus* | -74.8473 | 6.223417 |
| *Scinax x-signatus* | -74.8171 | 6.219139 |
| *Scinax x-signatus* | -74.8391 | 6.218611 |
| *Scinax x-signatus* | -48.475 | -8.05917 |
| *Scinax x-signatus* | -44.4147 | -2.40889 |
| *Scinax x-signatus* | -40.6592 | -20.3633 |
| *Scinax x-signatus* | -42.9444 | -8.11 |
| *Scinax x-signatus* | -42.475 | -14.0694 |
| *Scinax x-signatus* | -42.8256 | -8.21389 |
| *Scinax x-signatus* | -40.6003 | -19.9356 |
| *Scinax x-signatus* | -36.6011 | -5.66556 |
| *Scinax x-signatus* | -49.0686 | -11.7292 |
| *Scinax x-signatus* | -47.5047 | -4.94667 |
| *Scinax x-signatus* | -34.8256 | -7.74778 |
| *Scinax x-signatus* | -40.9211 | -3.85444 |
| *Scinax x-signatus* | -40.0836 | -13.8575 |
| *Scinax x-signatus* | -40.8244 | -21.0433 |
| *Scinax x-signatus* | -48.2072 | -7.19111 |
| *Scinax x-signatus* | -49.1486 | -13.4408 |
| *Scinax x-signatus* | -37.4253 | -10.685 |
| *Scinax x-signatus* | -32.4108 | -3.84028 |
| *Scinax x-signatus* | -43.425 | -19.0372 |
| *Scinax x-signatus* | -43.3089 | -17.0647 |
| *Scinax x-signatus* | -42.1433 | -18.3244 |
| *Scinax x-signatus* | -43.6003 | -18.2494 |
| *Scinax x-signatus* | -47.51 | -14.1325 |
| *Scinax x-signatus* | -43.4075 | -20.0747 |
| *Scinax x-signatus* | -44.3028 | -2.52972 |
| *Scinax x-signatus* | -46.0094 | -17.0097 |
| *Scinax x-signatus* | -44.18 | -17.8733 |
| *Scinax x-signatus* | -43.8578 | -20.4997 |
| *Scinax x-signatus* | -42.8897 | -16.5594 |
| *Scinax x-signatus* | -55.8661 | -1.76556 |
| *Scinax x-signatus* | -43.745 | -19.5136 |
| *Scinax x-signatus* | -43.3211 | -19.4144 |
| *Scinax x-signatus* | -46.42 | -23.89 |
| *Scinax x-signatus* | -44.9419 | -17.345 |
| *Scinax x-signatus* | -48.9158 | -14.9708 |
| *Scinax x-signatus* | -44.4308 | -18.7564 |
| *Scinax x-signatus* | -43.7894 | -20.0875 |
| *Scinax x-signatus* | -44.0797 | -21.0575 |
| *Scinax x-signatus* | -43.4153 | -19.9594 |
| *Scinax x-signatus* | -48.0783 | -14.1514 |
| *Scinax x-signatus* | -43.9378 | -19.9208 |
| *Scinax x-signatus* | -43.7144 | -19.1689 |
| *Scinax x-signatus* | -35.0922 | -8.11861 |
| *Scinax x-signatus* | -43.4161 | -20.3778 |
| *Scinax x-signatus* | -41.8114 | -13.5789 |
| *Scinax x-signatus* | -42.4333 | -14.8497 |
| *Scinax x-signatus* | -42.9403 | -14.5731 |
| *Scinax x-signatus* | -43.1619 | -14.2672 |
| *Scinax x-signatus* | -43.2269 | -19.6192 |
| *Scinax x-signatus* | -51.3967 | -16.0078 |
| *Scinax x-signatus* | -42.5397 | -15.6097 |
| *Scinax x-signatus* | -40.3608 | -10.7417 |
| *Scinax x-signatus* | -43.8014 | -20.2533 |
| *Scinax x-signatus* | -43.4872 | -19.9458 |
| *Scinax x-signatus* | -44.3617 | -15.4881 |
| *Scinax x-signatus* | -36.6611 | -9.7525 |
| *Scinax x-signatus* | -36.5864 | -10.2903 |
| *Scinax x-signatus* | -44.1997 | -20.1433 |
| *Scinax x-signatus* | -41.0639 | -19.4958 |
| *Scinax x-signatus* | -44.4211 | -20.1967 |
| *Scinax x-signatus* | -43.3622 | -19.8261 |
| *Scinax x-signatus* | -40.1494 | -16.2489 |
| *Scinax x-signatus* | -46.39 | -23.96 |
| *Scinax x-signatus* | -56.0861 | -9.87556 |
| *Scinax x-signatus* | -47.8831 | -12.6153 |
| *Scinax x-signatus* | -37.3089 | -7.1625 |
| *Scinax x-signatus* | -39.4958 | -15.4192 |
| *Scinax x-signatus* | -41.0069 | -20.6714 |
| *Scinax x-signatus* | -48.31 | -20.31 |
| *Scinax x-signatus* | -48.5044 | -1.45583 |
| *Scinax x-signatus* | -49.87 | -22.97 |
| *Scinax x-signatus* | -38.5108 | -12.9711 |
| *Scinax x-signatus* | -40.0978 | -19.1969 |
| *Eleutherodactylus johnstonei* | -66.8983 | 10.49605 |
| *Eleutherodactylus johnstonei* | -64.703 | 32.338 |
| *Eleutherodactylus johnstonei* | -61.783 | 17.608 |
| *Eleutherodactylus johnstonei* | -62.216 | 16.755 |
| *Eleutherodactylus johnstonei* | -61.645 | 12.081 |
| *Eleutherodactylus johnstonei* | -61.556 | 10.713 |
| *Eleutherodactylus johnstonei* | -66.9167 | 10.5 |
| *Eleutherodactylus johnstonei* | -61.217 | 13.233 |
| *Eleutherodactylus johnstonei* | -62.571 | 17.1113 |
| *Eleutherodactylus johnstonei* | -64.168 | 10.463 |
| *Eleutherodactylus johnstonei* | -61.672 | 12.136 |
| *Eleutherodactylus johnstonei* | -62.595 | 17.152 |
| *Eleutherodactylus johnstonei* | -61.0167 | 13.81667 |
| *Eleutherodactylus johnstonei* | -61.167 | 13.333 |
| *Eleutherodactylus johnstonei* | -61.217 | 13.25 |
| *Eleutherodactylus johnstonei* | -74.8003 | 10.98361 |
| *Eleutherodactylus johnstonei* | -61.233 | 13.2 |
| *Eleutherodactylus johnstonei* | -61.757 | 17.623 |
| *Eleutherodactylus johnstonei* | -61.683 | 12.033 |
| *Eleutherodactylus johnstonei* | -61.662 | 12.169 |
| *Eleutherodactylus johnstonei* | -62.2 | 16.73333 |
| *Eleutherodactylus johnstonei* | -63.261 | 17.628 |
| *Eleutherodactylus johnstonei* | -61.733 | 12.067 |
| *Eleutherodactylus johnstonei* | -61.636 | 12.214 |
| *Eleutherodactylus johnstonei* | -68.929 | 10.916 |
| *Eleutherodactylus johnstonei* | -46.63 | -23.54 |
| *Eleutherodactylus johnstonei* | -64.7 | 32.36667 |
| *Eleutherodactylus johnstonei* | -61.775 | 17.6592 |
| *Eleutherodactylus johnstonei* | -62.2194 | 16.78194 |
| *Eleutherodactylus johnstonei* | -61 | 12 |
| *Eleutherodactylus johnstonei* | -61.5554 | 10.68781 |
| *Eleutherodactylus johnstonei* | -62.5514 | 17.1111 |
| *Eleutherodactylus johnstonei* | -64.1833 | 10.46667 |
| *Eleutherodactylus johnstonei* | -61.617 | 12.117 |
| *Eleutherodactylus johnstonei* | -62.57 | 17.13 |
| *Eleutherodactylus johnstonei* | -61.75 | 17.65 |
| *Eleutherodactylus johnstonei* | -61.617 | 12.15 |
| *Eleutherodactylus johnstonei* | -63.2381 | 17.62861 |
| *Eleutherodactylus johnstonei* | -61.633 | 12.233 |
| *Eleutherodactylus johnstonei* | -73.1112 | 7.10617 |
| *Eleutherodactylus johnstonei* | -73.1088 | 7.11866 |
| *Eleutherodactylus johnstonei* | -73.0968 | 7.10423 |
| *Eleutherodactylus johnstonei* | -76.5361 | 3.41804 |
| *Eleutherodactylus johnstonei* | -63.2302 | 17.63055 |
| *Eleutherodactylus johnstonei* | -63.2396 | 17.63389 |
| *Eleutherodactylus johnstonei* | -59.43 | 13.11 |
| *Eleutherodactylus johnstonei* | -76.5251 | 3.41744 |
| *Eleutherodactylus johnstonei* | -62.2164 | 16.78397 |
| *Eleutherodactylus johnstonei* | -61.638 | 12.22433 |
| *Eleutherodactylus johnstonei* | -59.51 | 13.21 |
| *Eleutherodactylus johnstonei* | -73.1093 | 7.12821 |
| *Eleutherodactylus johnstonei* | -63.05 | 18.21 |
| *Eleutherodactylus johnstonei* | -63.0317 | 18.04193 |
| *Eleutherodactylus johnstonei* | -73.0966 | 7.1042 |
| *Eleutherodactylus johnstonei* | -61.2167 | 13.2333 |
| *Eleutherodactylus johnstonei* | -73.0963 | 7.10333 |
| *Eleutherodactylus johnstonei* | -61.1403 | 13.2445 |
| *Eleutherodactylus johnstonei* | -64.7583 | 32.3 |
| *Eleutherodactylus johnstonei* | -59.59 | 13.07 |
| *Eleutherodactylus johnstonei* | -73.1038 | 7.11459 |
| *Eleutherodactylus johnstonei* | -58.15 | 6.8 |
| *Eleutherodactylus johnstonei* | -73.069 | 6.99246 |
| *Eleutherodactylus johnstonei* | -78.3066 | 18.36914 |
| *Eleutherodactylus johnstonei* | -77.2729 | 18.14744 |
| *Eleutherodactylus johnstonei* | -64.6725 | 32.34056 |
| *Eleutherodactylus johnstonei* | -76.6869 | 18.08834 |
| *Eleutherodactylus johnstonei* | -46.6828 | 23.63325 |
| *Lithobates catesbeianus* | -77.7044 | 38.6897 |
| *Lithobates catesbeianus* | -82.2845 | 31.1922 |
| *Lithobates catesbeianus* | 129 | 35 |
| *Lithobates catesbeianus* | -97.644 | 30.68342 |
| *Lithobates catesbeianus* | 5.10529 | 51.32721 |
| *Lithobates catesbeianus* | -85.2143 | 30.8712 |
| *Lithobates catesbeianus* | -82.37 | 29.63656 |
| *Lithobates catesbeianus* | -82.3391 | 29.6809 |
| *Lithobates catesbeianus* | -84.384 | 30.5538 |
| *Lithobates catesbeianus* | -87.2932 | 30.9381 |
| *Lithobates catesbeianus* | -84.1518 | 30.28435 |
| *Lithobates catesbeianus* | -81.7471 | 29.4209 |
| *Lithobates catesbeianus* | 141 | 38.366 |
| *Lithobates catesbeianus* | -72.4645 | 41.30277 |
| *Lithobates catesbeianus* | -72.7968 | 41.279 |
| *Lithobates catesbeianus* | -73.1168 | 41.3634 |
| *Lithobates catesbeianus* | -92.2338 | 37.60105 |
| *Lithobates catesbeianus* | -72.6229 | 41.33129 |
| *Lithobates catesbeianus* | -72.8575 | 41.38178 |
| *Lithobates catesbeianus* | -72.6929 | 41.32137 |
| *Lithobates catesbeianus* | -94.8006 | 36.3834 |
| *Lithobates catesbeianus* | -72.5313 | 41.29543 |
| *Lithobates catesbeianus* | -76.3925 | 3.77568 |
| *Lithobates catesbeianus* | -82.9942 | 41.46 |
| *Lithobates catesbeianus* | -81.6956 | 41.4994 |
| *Lithobates catesbeianus* | -73.2817 | 5.12778 |
| *Lithobates catesbeianus* | -73.0492 | 6.86586 |
| *Lithobates catesbeianus* | -76.162 | 4.78256 |
| *Lithobates catesbeianus* | -74.4231 | 4.89697 |
| *Lithobates catesbeianus* | -76.1789 | 4.78786 |
| *Lithobates catesbeianus* | -74.7838 | 5.68833 |
| *Lithobates catesbeianus* | -112.07 | 41.50818 |
| *Lithobates catesbeianus* | -79.9332 | 44.99417 |
| *Lithobates catesbeianus* | -78.5071 | 44.5923 |
| *Lithobates catesbeianus* | -77.6 | 46.1 |
| *Lithobates catesbeianus* | -84.1333 | 46.68333 |
| *Lithobates catesbeianus* | -72.9208 | 46.01694 |
| *Lithobates catesbeianus* | -84.7328 | 47.70278 |
| *Lithobates catesbeianus* | -78.9333 | 45.6666 |
| *Lithobates catesbeianus* | -77.5847 | 45.88583 |
| *Lithobates catesbeianus* | -81.5132 | 43.96082 |
| *Lithobates catesbeianus* | -76.1661 | 44.48326 |
| *Lithobates catesbeianus* | -78.3667 | 45.63333 |
| *Lithobates catesbeianus* | -78.6804 | 45.51635 |
| *Lithobates catesbeianus* | -78.6833 | 45.8666 |
| *Lithobates catesbeianus* | -79.7857 | 45.21055 |
| *Lithobates catesbeianus* | -78.3925 | 45.81601 |
| *Lithobates catesbeianus* | -80.5167 | 45.55 |
| *Lithobates catesbeianus* | -79.4483 | 43.93469 |
| *Lithobates catesbeianus* | -79.2719 | 44.0306 |
| *Lithobates catesbeianus* | -97.3906 | 35.21137 |
| *Lithobates catesbeianus* | -95.3175 | 34.99551 |
| *Lithobates catesbeianus* | -97.4712 | 35.51554 |
| *Lithobates catesbeianus* | -94.7637 | 33.78341 |
| *Lithobates catesbeianus* | 4.74992 | 51.47043 |
| *Lithobates catesbeianus* | 4.75142 | 51.47456 |
| *Lithobates catesbeianus* | 4.7482 | 51.46951 |
| *Lithobates catesbeianus* | 5.10743 | 51.32634 |
| *Lithobates catesbeianus* | 5.12888 | 51.14383 |
| *Lithobates catesbeianus* | 5.12909 | 51.14483 |
| *Lithobates catesbeianus* | 5.12878 | 51.14356 |
| *Lithobates catesbeianus* | 5.13015 | 51.14471 |
| *Lithobates catesbeianus* | 5.1301 | 51.14438 |
| *Lithobates catesbeianus* | 5.12892 | 51.14412 |
| *Lithobates catesbeianus* | 5.12893 | 51.14444 |
| *Lithobates catesbeianus* | 5.1102 | 51.32551 |
| *Lithobates catesbeianus* | 5.10159 | 51.32636 |
| *Lithobates catesbeianus* | 5.1302 | 51.1451 |
| *Lithobates catesbeianus* | 5.12993 | 51.14503 |
| *Lithobates catesbeianus* | 5.01567 | 51.26937 |
| *Lithobates catesbeianus* | 5.01483 | 51.2686 |
| *Lithobates catesbeianus* | 5.01565 | 51.26731 |
| *Lithobates catesbeianus* | -95.104 | 31.40955 |
| *Lithobates catesbeianus* | -95.1573 | 31.40774 |
| *Lithobates catesbeianus* | -87.8081 | 42.4758 |
| *Lithobates catesbeianus* | -87.0927 | 41.644 |
| *Lithobates catesbeianus* | -83.4206 | 42.9044 |
| *Lithobates catesbeianus* | -83.6489 | 45.9589 |
| *Lithobates catesbeianus* | -82.6142 | 42.578 |
| *Lithobates catesbeianus* | -95.6042 | 47.0371 |
| *Lithobates catesbeianus* | -78.8525 | 42.8463 |
| *Lithobates catesbeianus* | -76.7582 | 42.977 |
| *Lithobates catesbeianus* | -78.4008 | 42.4429 |
| *Lithobates catesbeianus* | -75.8369 | 44.2766 |
| *Lithobates catesbeianus* | -75.8485 | 44.0143 |
| *Lithobates catesbeianus* | -81.617 | 40.6775 |
| *Lithobates catesbeianus* | -81.5916 | 40.621 |
| *Lithobates catesbeianus* | -81.9832 | 40.7116 |
| *Lithobates catesbeianus* | -81.3502 | 41.7138 |
| *Lithobates catesbeianus* | -83.2338 | 41.639 |
| *Lithobates catesbeianus* | -81.3147 | 41.0671 |
| *Lithobates catesbeianus* | -83.1997 | 41.6223 |
| *Lithobates catesbeianus* | -81.7768 | 41.2945 |
| *Lithobates catesbeianus* | -81.8296 | 41.3072 |
| *Lithobates catesbeianus* | -82.9918 | 41.5321 |
| *Lithobates catesbeianus* | -82.9956 | 41.5286 |
| *Lithobates catesbeianus* | -83.3058 | 41.6752 |
| *Lithobates catesbeianus* | -83.3157 | 41.682 |
| *Lithobates catesbeianus* | -83.2103 | 41.6253 |
| *Lithobates catesbeianus* | -74.5131 | 45.1164 |
| *Lithobates catesbeianus* | -82.4015 | 42.3691 |
| *Lithobates catesbeianus* | -77.3468 | 44.165 |
| *Lithobates catesbeianus* | -75.6377 | 45.1519 |
| *Lithobates catesbeianus* | -81.7273 | 46.1721 |
| *Lithobates catesbeianus* | -81.875 | 42.2697 |
| *Lithobates catesbeianus* | -76.2578 | 44.7908 |
| *Lithobates catesbeianus* | -81.2167 | 44.7833 |
| *Lithobates catesbeianus* | -81.25 | 44.8 |
| *Lithobates catesbeianus* | -76.4419 | 45.03 |
| *Lithobates catesbeianus* | -77.22 | 44.1165 |
| *Lithobates catesbeianus* | -77.7273 | 44.0008 |
| *Lithobates catesbeianus* | -80.303 | 43.1498 |
| *Lithobates catesbeianus* | -80.4062 | 43.0522 |
| *Lithobates catesbeianus* | -80.3667 | 45.4333 |
| *Lithobates catesbeianus* | -79.1914 | 45.116 |
| *Lithobates catesbeianus* | -77.6688 | 45.3604 |
| *Lithobates catesbeianus* | -83.0334 | 42.0333 |
| *Lithobates catesbeianus* | -80.2268 | 43.5107 |
| *Lithobates catesbeianus* | -83.0783 | 42.2644 |
| *Lithobates catesbeianus* | -78.9869 | 43.83 |
| *Lithobates catesbeianus* | -78.8393 | 43.8598 |
| *Lithobates catesbeianus* | -80.1051 | 42.1548 |
| *Lithobates catesbeianus* | -82.2506 | 35.4325 |
| *Lithobates catesbeianus* | -82.4611 | 35.31861 |
| *Lithobates catesbeianus* | -82.9486 | 38.3325 |
| *Lithobates catesbeianus* | -82.3842 | 39.66792 |
| *Lithobates catesbeianus* | -84.0722 | 39.40814 |
| *Lithobates catesbeianus* | -120.381 | 37.84455 |
| *Lithobates catesbeianus* | -99.108 | 18.453 |
| *Lithobates catesbeianus* | -74.1414 | 41.72278 |
| *Lithobates catesbeianus* | -75.6433 | 45.01694 |
| *Lithobates catesbeianus* | -82.42 | 29.6 |
| *Lithobates catesbeianus* | -95.952 | 34.45893 |
| *Lithobates catesbeianus* | -95.8838 | 34.42647 |
| *Lithobates catesbeianus* | -94.7441 | 34.68206 |
| *Lithobates catesbeianus* | -94.6979 | 34.76459 |
| *Lithobates catesbeianus* | -94.8318 | 35.67467 |
| *Lithobates catesbeianus* | -106.315 | 31.70593 |
| *Lithobates catesbeianus* | -106.675 | 32.04972 |
| *Lithobates catesbeianus* | -98.7409 | 30.22368 |
| *Lithobates catesbeianus* | -84.9609 | 30.87845 |
| *Lithobates catesbeianus* | -83.2087 | 41.60867 |
| *Lithobates catesbeianus* | -78.4666 | 45.78333 |
| *Lithobates catesbeianus* | -78.5516 | 45.93487 |
| *Lithobates catesbeianus* | -79.2592 | 44.85363 |
| *Lithobates catesbeianus* | -76.7221 | 44.65731 |
| *Lithobates catesbeianus* | -78.6762 | 44.78902 |
| *Lithobates catesbeianus* | -77.2789 | 43.96983 |
| *Lithobates catesbeianus* | -78.4833 | 46.08333 |
| *Lithobates catesbeianus* | -119.167 | 36.36671 |
| *Lithobates catesbeianus* | -95.8903 | 41.1723 |
| *Lithobates catesbeianus* | -86.9055 | 36.06078 |
| *Lithobates catesbeianus* | -88.2349 | 41.95919 |
| *Lithobates catesbeianus* | -116.901 | 33.34021 |
| *Lithobates catesbeianus* | -95.9635 | 35.6253 |
| *Lithobates catesbeianus* | -97.05 | 34.48926 |
| *Lithobates catesbeianus* | -95.4287 | 34.367 |
| *Lithobates catesbeianus* | -98.4445 | 36.41544 |
| *Lithobates catesbeianus* | -95.0925 | 35.5498 |
| *Lithobates catesbeianus* | -95.3072 | 34.948 |
| *Lithobates catesbeianus* | -95.2445 | 34.9254 |
| *Lithobates catesbeianus* | -98.4861 | 34.66583 |
| *Lithobates catesbeianus* | -96.9222 | 35.34037 |
| *Lithobates catesbeianus* | -98.3695 | 34.81157 |
| *Lithobates catesbeianus* | -96.7781 | 34.78238 |
| *Lithobates catesbeianus* | -100.287 | 36.85573 |
| *Lithobates catesbeianus* | -97.0276 | 35.96406 |
| *Lithobates catesbeianus* | -96.6274 | 34.45579 |
| *Lithobates catesbeianus* | -94.6412 | 36.32818 |
| *Lithobates catesbeianus* | -95.0257 | 33.85921 |
| *Lithobates catesbeianus* | -94.7003 | 35.92049 |
| *Lithobates catesbeianus* | -94.7832 | 36.10404 |
| *Lithobates catesbeianus* | -94.9721 | 34.65165 |
| *Lithobates catesbeianus* | -76.2505 | 35.7718 |
| *Lithobates catesbeianus* | -78.5219 | 34.8867 |
| *Lithobates catesbeianus* | -81.7134 | 36.1379 |
| *Lithobates catesbeianus* | -78.717 | 35.7982 |
| *Lithobates catesbeianus* | -77.9333 | 34.7352 |
| *Lithobates catesbeianus* | -80.5303 | 34.5865 |
| *Lithobates catesbeianus* | -78.101 | 33.9491 |
| *Lithobates catesbeianus* | -79.3564 | 34.8137 |
| *Lithobates catesbeianus* | -78.0238 | 34.2578 |
| *Lithobates catesbeianus* | -82.2018 | 35.6436 |
| *Lithobates catesbeianus* | -78.3625 | 34.1625 |
| *Lithobates catesbeianus* | -81.3564 | 35.1266 |
| *Lithobates catesbeianus* | -80.7578 | 35.4957 |
| *Lithobates catesbeianus* | -81.1067 | 35.9648 |
| *Lithobates catesbeianus* | -79.1998 | 34.8628 |
| *Lithobates catesbeianus* | -79.3943 | 34.9291 |
| *Lithobates catesbeianus* | -75.6798 | 39.4539 |
| *Lithobates catesbeianus* | -75.4743 | 38.5241 |
| *Lithobates catesbeianus* | -77.6571 | 34.4126 |
| *Lithobates catesbeianus* | -77.3372 | 37.6089 |
| *Lithobates catesbeianus* | -76.9658 | 37.4236 |
| *Lithobates catesbeianus* | -77.4819 | 37.7381 |
| *Lithobates catesbeianus* | -76.4491 | 36.9008 |
| *Lithobates catesbeianus* | -77.9037 | 37.4699 |
| *Lithobates catesbeianus* | -77.2841 | 37.3672 |
| *Lithobates catesbeianus* | -76.9107 | 37.4433 |
| *Lithobates catesbeianus* | -158.005 | 21.3805 |
| *Lithobates catesbeianus* | -157.979 | 21.3798 |
| *Lithobates catesbeianus* | -156.13 | 20.85 |
| *Lithobates catesbeianus* | -157.801 | 21.3888 |
| *Lithobates catesbeianus* | -77.5358 | 35.8968 |
| *Lithobates catesbeianus* | -83.7656 | 35.98089 |
| *Lithobates catesbeianus* | -79.8626 | 32.7941 |
| *Lithobates catesbeianus* | -82.8208 | 41.71527 |
| *Lithobates catesbeianus* | -101.801 | 39.77216 |
| *Lithobates catesbeianus* | -83.0753 | 31.0411 |
| *Lithobates catesbeianus* | -96.8765 | 35.87703 |
| *Lithobates catesbeianus* | -77.25 | 38.99 |
| *Lithobates catesbeianus* | -96.5073 | 37.07057 |
| *Lithobates catesbeianus* | -120.8 | 38.36954 |
| *Lithobates catesbeianus* | -83.8068 | 36.23321 |
| *Lithobates catesbeianus* | -88.9448 | 32.92115 |
| *Lithobates catesbeianus* | -97.0791 | 37.16851 |
| *Lithobates catesbeianus* | -121.289 | 38.75196 |
| *Lithobates catesbeianus* | -119.272 | 39.29631 |
| *Lithobates catesbeianus* | -76.46 | 37.05 |
| *Lithobates catesbeianus* | -76.8137 | 39.05715 |
| *Lithobates catesbeianus* | -72.6111 | 44.5742 |
| *Lithobates catesbeianus* | -76.8483 | 39.0993 |
| *Lithobates catesbeianus* | -71.1406 | 44.0742 |
| *Lithobates catesbeianus* | -81.5433 | 37.1277 |
| *Lithobates catesbeianus* | -77.3079 | 37.81431 |
| *Lithobates catesbeianus* | -76.5075 | 38.1423 |
| *Lithobates catesbeianus* | -78.13 | 38.88 |
| *Lithobates catesbeianus* | 132.756 | 34.44361 |
| *Lithobates catesbeianus* | -75.7286 | 38.9522 |
| *Lithobates catesbeianus* | -70.6433 | 44.9664 |
| *Lithobates catesbeianus* | -77.08 | 34.93 |
| *Lithobates catesbeianus* | -79.3637 | 34.7877 |
| *Lithobates catesbeianus* | -82.3334 | 36.01385 |
| *Lithobates catesbeianus* | -92.6986 | 29.64639 |
| *Lithobates catesbeianus* | -76.44 | 35.53734 |
| *Lithobates catesbeianus* | 128.2029 | 26.73292 |
| *Lithobates catesbeianus* | -71.1283 | 42.2111 |
| *Lithobates catesbeianus* | -77.28 | 37.67 |
| *Lithobates catesbeianus* | -77.52 | 37.4 |
| *Lithobates catesbeianus* | -77.54 | 37.84 |
| *Lithobates catesbeianus* | -77.67 | 37.53 |
| *Lithobates catesbeianus* | -77.267 | 36.9796 |
| *Lithobates catesbeianus* | -76.14 | 36.65 |
| *Lithobates catesbeianus* | -77.58 | 36.58 |
| *Lithobates catesbeianus* | -101.783 | 43.6436 |
| *Lithobates catesbeianus* | -88.6115 | 33.50541 |
| *Lithobates catesbeianus* | -76.3689 | 39.5239 |
| *Lithobates catesbeianus* | -116.702 | 34.0082 |
| *Lithobates catesbeianus* | -116.314 | 33.84923 |
| *Lithobates catesbeianus* | -117.87 | 33.87226 |
| *Lithobates catesbeianus* | -117.593 | 33.38692 |
| *Lithobates catesbeianus* | -117.95 | 36.53919 |
| *Lithobates catesbeianus* | -119.433 | 41.15 |
| *Lithobates catesbeianus* | -114.667 | 36.683 |
| *Lithobates catesbeianus* | -116.766 | 36.8833 |
| *Lithobates catesbeianus* | -117.557 | 46.1768 |
| *Lithobates catesbeianus* | -120.968 | 37.9202 |
| *Lithobates catesbeianus* | -109.706 | 31.47384 |
| *Lithobates catesbeianus* | -109.279 | 31.3375 |
| *Lithobates catesbeianus* | -122.312 | 37.91576 |
| *Lithobates catesbeianus* | -122.042 | 39.1575 |
| *Lithobates catesbeianus* | -111.255 | 31.5333 |
| *Lithobates catesbeianus* | -114.286 | 34.4358 |
| *Lithobates catesbeianus* | -66.0054 | 18.32528 |
| *Lithobates catesbeianus* | -66.6949 | 18.47749 |
| *Lithobates catesbeianus* | -122.583 | 47.06171 |
| *Lithobates catesbeianus* | -121.745 | 39.49378 |
| *Lithobates catesbeianus* | -114.617 | 48.2044 |
| *Lithobates catesbeianus* | -105.102 | 39.84056 |
| *Lithobates catesbeianus* | -105.1 | 39.84275 |
| *Lithobates catesbeianus* | -91.5872 | 37.95125 |
| *Lithobates catesbeianus* | -95.6659 | 37.0416 |
| *Lithobates catesbeianus* | -101.763 | 40.04166 |
| *Lithobates catesbeianus* | -101.521 | 37.2563 |
| *Lithobates catesbeianus* | -94.7189 | 37.0223 |
| *Lithobates catesbeianus* | -94.8417 | 37.7984 |
| *Lithobates catesbeianus* | -94.8322 | 37.7979 |
| *Lithobates catesbeianus* | -82.2979 | 37.2884 |
| *Lithobates catesbeianus* | -70.6272 | 43.8414 |
| *Lithobates catesbeianus* | -122.241 | 38.48357 |
| *Lithobates catesbeianus* | -117.173 | 33.52845 |
| *Lithobates catesbeianus* | -121.034 | 36.49422 |
| *Lithobates catesbeianus* | -118.657 | 35.70503 |
| *Lithobates catesbeianus* | -121.456 | 37.07202 |
| *Lithobates catesbeianus* | -117.254 | 33.55634 |
| *Lithobates catesbeianus* | -116.994 | 33.67653 |
| *Lithobates catesbeianus* | -122.239 | 38.89029 |
| *Lithobates catesbeianus* | -121.754 | 37.1767 |
| *Lithobates catesbeianus* | -121.019 | 39.98451 |
| *Lithobates catesbeianus* | -121.985 | 37.81647 |
| *Lithobates catesbeianus* | -121.883 | 37.79892 |
| *Lithobates catesbeianus* | -122.577 | 39.28544 |
| *Lithobates catesbeianus* | -112.896 | 27.28232 |
| *Lithobates catesbeianus* | -119.149 | 36.87915 |
| *Lithobates catesbeianus* | -122.665 | 38.52988 |
| *Lithobates catesbeianus* | -122.886 | 38.50841 |
| *Lithobates catesbeianus* | -117.594 | 33.38596 |
| *Lithobates catesbeianus* | -121.193 | 40.0089 |
| *Lithobates catesbeianus* | -116.799 | 32.77206 |
| *Lithobates catesbeianus* | -121.736 | 37.09117 |
| *Lithobates catesbeianus* | -121.742 | 37.09904 |
| *Lithobates catesbeianus* | -120.924 | 39.39639 |
| *Lithobates catesbeianus* | -121.744 | 37.08876 |
| *Lithobates catesbeianus* | -114.402 | 34.48081 |
| *Lithobates catesbeianus* | -122.23 | 37.84778 |
| *Lithobates catesbeianus* | -122.175 | 37.42194 |
| *Lithobates catesbeianus* | -120.02 | 36.94523 |
| *Lithobates catesbeianus* | -75.6222 | 5.7306 |
| *Lithobates catesbeianus* | -75.45 | 45.367 |
| *Lithobates catesbeianus* | -76.417 | 45.517 |
| *Lithobates catesbeianus* | -77.747 | 45.504 |
| *Lithobates catesbeianus* | -65.15 | 44.267 |
| *Lithobates catesbeianus* | -122.533 | 49.133 |
| *Lithobates catesbeianus* | -76.183 | 45.5 |
| *Lithobates catesbeianus* | -75.3 | 45.217 |
| *Lithobates catesbeianus* | -75.95 | 45.467 |
| *Lithobates catesbeianus* | -73.974 | 46.42 |
| *Lithobates catesbeianus* | -75.668 | 44.988 |
| *Lithobates catesbeianus* | -76.055 | 45.601 |
| *Lithobates catesbeianus* | -76.213 | 45.666 |
| *Lithobates catesbeianus* | -76.107 | 45.606 |
| *Lithobates catesbeianus* | -75.856 | 45.482 |
| *Lithobates catesbeianus* | -76.083 | 45.598 |
| *Lithobates catesbeianus* | -76.917 | 44.717 |
| *Lithobates catesbeianus* | -65.206 | 44.134 |
| *Lithobates catesbeianus* | -76.306 | 46.244 |
| *Lithobates catesbeianus* | -75.3 | 45.567 |
| *Lithobates catesbeianus* | -75.682 | 45.054 |
| *Lithobates catesbeianus* | -76.17 | 45.887 |
| *Lithobates catesbeianus* | -75.167 | 45.583 |
| *Lithobates catesbeianus* | -76.692 | 45.325 |
| *Lithobates catesbeianus* | -75.053 | 45.792 |
| *Lithobates catesbeianus* | -75.655 | 44.874 |
| *Lithobates catesbeianus* | -75.983 | 46.117 |
| *Lithobates catesbeianus* | -76.753 | 45.354 |
| *Lithobates catesbeianus* | -75.567 | 45.467 |
| *Lithobates catesbeianus* | -75.683 | 45.417 |
| *Lithobates catesbeianus* | -81.917 | 46.167 |
| *Lithobates catesbeianus* | -81.683 | 46.25 |
| *Lithobates catesbeianus* | -76.033 | 46.233 |
| *Lithobates catesbeianus* | -80.1 | 40.35 |
| *Lithobates catesbeianus* | -77.53 | 40.08 |
| *Lithobates catesbeianus* | -87.2899 | 38.27001 |
| *Lithobates catesbeianus* | -78.8496 | 39.43 |
| *Lithobates catesbeianus* | -79.9697 | 38.71001 |
| *Lithobates catesbeianus* | -73.46 | 41.94 |
| *Lithobates catesbeianus* | -75.35 | 40.55 |
| *Lithobates catesbeianus* | -85.89 | 30.45 |
| *Lithobates catesbeianus* | -75.69 | 39.36 |
| *Lithobates catesbeianus* | -75.21 | 40.05 |
| *Lithobates catesbeianus* | -79.85 | 42.07 |
| *Lithobates catesbeianus* | -76.89 | 40.37 |
| *Lithobates catesbeianus* | -75.14 | 38.29 |
| *Lithobates catesbeianus* | -76.99 | 40.57 |
| *Lithobates catesbeianus* | -77.8 | 40.79 |
| *Lithobates catesbeianus* | -74.26 | 44.63 |
| *Lithobates catesbeianus* | -81.34 | 32.05 |
| *Lithobates catesbeianus* | -70.47 | 43.36 |
| *Lithobates catesbeianus* | -78.2996 | 39.53999 |
| *Lithobates catesbeianus* | -76.15 | 36.86 |
| *Lithobates catesbeianus* | -77.83 | 37.67 |
| *Lithobates catesbeianus* | -76.87 | 37.14 |
| *Lithobates catesbeianus* | -77.34 | 38.45 |
| *Lithobates catesbeianus* | -76.89 | 37.12 |
| *Lithobates catesbeianus* | -77.12 | 38.71 |
| *Lithobates catesbeianus* | -78.93 | 37.03 |
| *Lithobates catesbeianus* | -76.38 | 37.04 |
| *Lithobates catesbeianus* | -121.143 | 45.6126 |
| *Lithobates catesbeianus* | -122.583 | 47.4993 |
| *Lithobates catesbeianus* | -122.032 | 43.46749 |
| *Lithobates catesbeianus* | -116.386 | 35.03727 |
| *Lithobates catesbeianus* | -82.4724 | 39.9337 |
| *Lithobates catesbeianus* | -72.1542 | 43.1882 |
| *Lithobates catesbeianus* | -99.2506 | 31.2504 |
| *Lithobates catesbeianus* | -83.3044 | 40.8424 |
| *Lithobates catesbeianus* | -83.0193 | 40.1248 |
| *Lithobates catesbeianus* | -86.2503 | 35.7504 |
| *Lithobates catesbeianus* | -120.573 | 34.77036 |
| *Lithobates catesbeianus* | -118.757 | 34.52851 |
| *Lithobates catesbeianus* | -95.1351 | 38.55384 |
| *Lithobates catesbeianus* | -94.8771 | 38.95499 |
| *Lithobates catesbeianus* | -95.2348 | 38.97137 |
| *Lithobates catesbeianus* | -96.154 | 37.61944 |
| *Lithobates catesbeianus* | -95.4434 | 38.8497 |
| *Lithobates catesbeianus* | -95.2404 | 38.95998 |
| *Lithobates catesbeianus* | -95.5559 | 38.00555 |
| *Lithobates catesbeianus* | -108.93 | 25.8003 |
| *Lithobates catesbeianus* | -83.1078 | 39.55087 |
| *Lithobates catesbeianus* | -83.4822 | 40.53347 |
| *Lithobates catesbeianus* | -98.6993 | 37.6311 |
| *Lithobates catesbeianus* | -100.925 | 38.69135 |
| *Lithobates catesbeianus* | -94.7195 | 38.2761 |
| *Lithobates catesbeianus* | -95.8712 | 37.83194 |
| *Lithobates catesbeianus* | -94.642 | 37.03224 |
| *Lithobates catesbeianus* | -95.844 | 37.79667 |
| *Lithobates catesbeianus* | -96.1057 | 37.01004 |
| *Lithobates catesbeianus* | -101.689 | 38.90189 |
| *Lithobates catesbeianus* | 138.887 | 36.2769 |
| *Lithobates catesbeianus* | -95.1036 | 31.4263 |
| *Lithobates catesbeianus* | -95.1015 | 31.42754 |
| *Lithobates catesbeianus* | -97.5912 | 29.58759 |
| *Lithobates catesbeianus* | -96.3344 | 30.63472 |
| *Lithobates catesbeianus* | -96.3636 | 30.59056 |
| *Lithobates catesbeianus* | -95.9758 | 31.95691 |
| *Lithobates catesbeianus* | -97.2087 | 28.97849 |
| *Lithobates catesbeianus* | -97.3628 | 30.81472 |
| *Lithobates catesbeianus* | -72.2478 | 41.80694 |
| *Lithobates catesbeianus* | -96.5209 | 30.6412 |
| *Lithobates catesbeianus* | -98.9311 | 32.58246 |
| *Lithobates catesbeianus* | -98.4916 | 33.95192 |
| *Lithobates catesbeianus* | -98.5793 | 31.21953 |
| *Lithobates catesbeianus* | -94.0662 | 29.89371 |
| *Lithobates catesbeianus* | -120.575 | 35.37889 |
| *Lithobates catesbeianus* | -104.021 | 37.12739 |
| *Lithobates catesbeianus* | -94.5413 | 38.52558 |
| *Lithobates catesbeianus* | -95.2058 | 39.0381 |
| *Lithobates catesbeianus* | -111.476 | 31.56914 |
| *Lithobates catesbeianus* | -116.648 | 32.87988 |
| *Lithobates catesbeianus* | -116.662 | 32.8891 |
| *Lithobates catesbeianus* | -118.438 | 34.28194 |
| *Lithobates catesbeianus* | -119.285 | 35.19979 |
| *Lithobates catesbeianus* | -83.1968 | 35.0526 |
| *Lithobates catesbeianus* | -118.145 | 35.66854 |
| *Lithobates catesbeianus* | -89.7056 | 45.99572 |
| *Lithobates catesbeianus* | -111.085 | 31.40929 |
| *Lithobates catesbeianus* | -72.4427 | 41.79055 |
| *Lithobates catesbeianus* | -120.762 | 37.64078 |
| *Lithobates catesbeianus* | -84.4158 | 30.47831 |
| *Lithobates catesbeianus* | -122.34 | 38.48814 |
| *Lithobates catesbeianus* | -114.091 | 36.78993 |
| *Lithobates catesbeianus* | -121.441 | 36.82047 |
| *Lithobates catesbeianus* | -116.624 | 34.92154 |
| *Lithobates catesbeianus* | -122.795 | 39.09226 |
| *Lithobates catesbeianus* | -118.926 | 36.05355 |
| *Lithobates catesbeianus* | -116.759 | 36.87951 |
| *Lithobates catesbeianus* | -115.137 | 36.18943 |
| *Lithobates catesbeianus* | -116.521 | 34.07059 |
| *Lithobates catesbeianus* | -122.151 | 37.75428 |
| *Lithobates catesbeianus* | -116.291 | 36.3778 |
| *Lithobates catesbeianus* | -74.4258 | 40.63417 |
| *Lithobates catesbeianus* | -73.9886 | 40.86167 |
| *Lithobates catesbeianus* | -71.3308 | 42.41786 |
| *Lithobates catesbeianus* | -71.0663 | 42.29944 |
| *Lithobates catesbeianus* | -71.5332 | 43.74214 |
| *Lithobates catesbeianus* | -96.8548 | 32.65145 |
| *Lithobates catesbeianus* | -119.185 | 34.99432 |
| *Lithobates catesbeianus* | -96.9849 | 32.99483 |
| *Lithobates catesbeianus* | -88.5822 | 33.2344 |
| *Lithobates catesbeianus* | -71.1792 | 42.3958 |
| *Lithobates catesbeianus* | -68.1083 | 46.63 |
| *Lithobates catesbeianus* | -71.7322 | 41.44556 |
| *Lithobates catesbeianus* | -81.0756 | 35.7074 |
| *Lithobates catesbeianus* | -68.9683 | 46.36833 |
| *Lithobates catesbeianus* | -71.9835 | 42.45609 |
| *Lithobates catesbeianus* | -71.1172 | 43.6853 |
| *Lithobates catesbeianus* | -81.2022 | 28.56453 |
| *Lithobates catesbeianus* | -71.9985 | 42.21309 |
| *Lithobates catesbeianus* | -78.8587 | 35.05465 |
| *Lithobates catesbeianus* | -102 | 33.49721 |
| *Lithobates catesbeianus* | -100.388 | 34.13094 |
| *Lithobates catesbeianus* | -77.2829 | 45.89826 |
| *Lithobates catesbeianus* | -94.3901 | 29.57448 |
| *Lithobates catesbeianus* | -123.827 | 46.15144 |
| *Lithobates catesbeianus* | -76.724 | 39.06884 |
| *Lithobates catesbeianus* | -71.1492 | 42.38741 |
| *Lithobates catesbeianus* | -97.1598 | 30.37176 |
| *Lithobates catesbeianus* | -96.921 | 28.24309 |
| *Lithobates catesbeianus* | -94.5361 | 29.61381 |
| *Lithobates catesbeianus* | -117.038 | 32.84102 |
| *Lithobates catesbeianus* | -76.2294 | 43.82582 |
| *Lithobates catesbeianus* | -89.4399 | 37.57676 |
| *Lithobates catesbeianus* | -71.1464 | 42.38734 |
| *Lithobates catesbeianus* | -98.3951 | 30.55911 |
| *Lithobates catesbeianus* | -98.4047 | 33.63017 |
| *Lithobates catesbeianus* | -76.7169 | 39.17097 |
| *Lithobates catesbeianus* | -121.289 | 38.15161 |
| *Lithobates catesbeianus* | -108.577 | 32.9659 |
| *Lithobates catesbeianus* | -69.2561 | 44.20586 |
| *Lithobates catesbeianus* | -70.2089 | 41.2503 |
| *Lithobates catesbeianus* | -97.8824 | 30.58311 |
| *Lithobates catesbeianus* | -121.291 | 38.14987 |
| *Lithobates catesbeianus* | -118.201 | 34.09788 |
| *Lithobates catesbeianus* | 7.8307 | 44.90862 |
| *Lithobates catesbeianus* | -81.5859 | 41.31941 |
| *Lithobates catesbeianus* | -122.92 | 49.24708 |
| *Lithobates catesbeianus* | -73.1408 | 44.15682 |
| *Lithobates catesbeianus* | -104.719 | 40.44773 |
| *Lithobates catesbeianus* | -122.078 | 37.02895 |
| *Lithobates catesbeianus* | -76.0203 | 45.42226 |
| *Lithobates catesbeianus* | -88.3797 | 40.19972 |
| *Lithobates catesbeianus* | -71.5674 | 41.89749 |
| *Lithobates catesbeianus* | -122.954 | 49.24545 |
| *Lithobates catesbeianus* | -116.605 | 31.91751 |
| *Lithobates catesbeianus* | -121.642 | 37.30564 |
| *Lithobates catesbeianus* | 8.08246 | 45.1066 |
| *Lithobates catesbeianus* | -82.2837 | 38.38 |
| *Lithobates catesbeianus* | -95.2329 | 38.92082 |
| *Lithobates catesbeianus* | -96.5323 | 37.65833 |
| *Lithobates catesbeianus* | -98.738 | 37.6461 |
| *Lithobates catesbeianus* | -95.2498 | 38.92831 |
| *Lithobates catesbeianus* | -95.5615 | 37.19529 |
| *Lithobates catesbeianus* | -97.039 | 37.06362 |
| *Lithobates catesbeianus* | -98.7149 | 36.15776 |
| *Lithobates catesbeianus* | -95.2329 | 38.92109 |
| *Lithobates catesbeianus* | -95.202 | 38.94574 |
| *Lithobates catesbeianus* | -105.484 | 29.15019 |
| *Lithobates catesbeianus* | -107.3 | 30.28348 |
| *Lithobates catesbeianus* | -85.7343 | 36.18285 |
| *Lithobates catesbeianus* | -100.158 | 39.13752 |
| *Lithobates catesbeianus* | -96.4073 | 39.46969 |
| *Lithobates catesbeianus* | -94.8095 | 39.1947 |
| *Lithobates catesbeianus* | -95.2117 | 37.10251 |
| *Lithobates catesbeianus* | -94.8456 | 39.14331 |
| *Lithobates catesbeianus* | -72.2192 | 19.5467 |
| *Lithobates catesbeianus* | -75.683 | 45.35 |
| *Lithobates catesbeianus* | -65.117 | 44.433 |
| *Lithobates catesbeianus* | -75.917 | 45.633 |
| *Lithobates catesbeianus* | -75.767 | 45.467 |
| *Lithobates catesbeianus* | -67.083 | 45.15 |
| *Lithobates catesbeianus* | -124.45 | 49.3 |
| *Lithobates catesbeianus* | -74.056 | 46.578 |
| *Lithobates catesbeianus* | -81.984 | 43.207 |
| *Lithobates catesbeianus* | -75.911 | 45.51 |
| *Lithobates catesbeianus* | -76.103 | 45.613 |
| *Lithobates catesbeianus* | -75.938 | 45.611 |
| *Lithobates catesbeianus* | -76.021 | 45.601 |
| *Lithobates catesbeianus* | -76.013 | 45.596 |
| *Lithobates catesbeianus* | -76.07 | 45.598 |
| *Lithobates catesbeianus* | -76.015 | 45.604 |
| *Lithobates catesbeianus* | -75.583 | 45.633 |
| *Lithobates catesbeianus* | -80.15 | 43.467 |
| *Lithobates catesbeianus* | -65.355 | 44.551 |
| *Lithobates catesbeianus* | -75.8 | 45.38 |
| *Lithobates catesbeianus* | -114.417 | 47.333 |
| *Lithobates catesbeianus* | -75.679 | 44.861 |
| *Lithobates catesbeianus* | -76.067 | 46.133 |
| *Lithobates catesbeianus* | -75.838 | 46.171 |
| *Lithobates catesbeianus* | -75.8 | 45.533 |
| *Lithobates catesbeianus* | -75.617 | 44.883 |
| *Lithobates catesbeianus* | -74.302 | 45.09 |
| *Lithobates catesbeianus* | -74.433 | 45.55 |
| *Lithobates catesbeianus* | -76.05 | 45.783 |
| *Lithobates catesbeianus* | -75.867 | 45.5 |
| *Lithobates catesbeianus* | 139.487 | 36.2308 |
| *Lithobates catesbeianus* | -99.3365 | 38.84285 |
| *Lithobates catesbeianus* | -78.05 | 40.9 |
| *Lithobates catesbeianus* | -79.31 | 40.28 |
| *Lithobates catesbeianus* | -76.03 | 39.73 |
| *Lithobates catesbeianus* | -82.33 | 29.57 |
| *Lithobates catesbeianus* | -77.63 | 37.64 |
| *Lithobates catesbeianus* | -76.39 | 37.08 |
| *Lithobates catesbeianus* | -100.074 | 37.0488 |
| *Lithobates catesbeianus* | -100.962 | 38.6558 |
| *Lithobates catesbeianus* | -101.766 | 39.0464 |
| *Lithobates catesbeianus* | -98.6748 | 37.6457 |
| *Lithobates catesbeianus* | -101.829 | 37.1372 |
| *Lithobates catesbeianus* | -95.6923 | 37.1652 |
| *Lithobates catesbeianus* | -94.6985 | 37.05588 |
| *Lithobates catesbeianus* | -118.634 | 35.71038 |
| *Lithobates catesbeianus* | -119.946 | 37.83642 |
| *Lithobates catesbeianus* | -120.019 | 37.822 |
| *Lithobates catesbeianus* | -121.085 | 35.61088 |
| *Lithobates catesbeianus* | -120.857 | 39.93775 |
| *Lithobates catesbeianus* | -122.077 | 37.28436 |
| *Lithobates catesbeianus* | -117.268 | 33.5426 |
| *Lithobates catesbeianus* | -121.778 | 37.1688 |
| *Lithobates catesbeianus* | -121.691 | 37.31514 |
| *Lithobates catesbeianus* | -98.494 | 29.44661 |
| *Lithobates catesbeianus* | -116.2 | 43.6177 |
| *Lithobates catesbeianus* | -116.23 | 43.6195 |
| *Lithobates catesbeianus* | -70.9969 | 43.7611 |
| *Lithobates catesbeianus* | -68.6783 | 44.5667 |
| *Lithobates catesbeianus* | -82.3245 | 29.6514 |
| *Lithobates catesbeianus* | -86.4387 | 41.3315 |
| *Lithobates catesbeianus* | -88.0008 | 46.5666 |
| *Lithobates catesbeianus* | -77.388 | 43.0706 |
| *Lithobates catesbeianus* | -78.943 | 43.2883 |
| *Lithobates catesbeianus* | -78.4068 | 43.1354 |
| *Lithobates catesbeianus* | -78.9984 | 43.2732 |
| *Lithobates catesbeianus* | -78.9832 | 43.0584 |
| *Lithobates catesbeianus* | -76.8848 | 43.2809 |
| *Lithobates catesbeianus* | -76.1603 | 44.1158 |
| *Lithobates catesbeianus* | -77.7103 | 43.2975 |
| *Lithobates catesbeianus* | -81.5577 | 41.2201 |
| *Lithobates catesbeianus* | -82.1048 | 41.0941 |
| *Lithobates catesbeianus* | -81.4189 | 41.5575 |
| *Lithobates catesbeianus* | -81.323 | 41.7281 |
| *Lithobates catesbeianus* | -81.8366 | 41.3812 |
| *Lithobates catesbeianus* | -81.838 | 41.3822 |
| *Lithobates catesbeianus* | -83.2468 | 41.6451 |
| *Lithobates catesbeianus* | -83.2232 | 41.626 |
| *Lithobates catesbeianus* | -82.4408 | 41.395 |
| *Lithobates catesbeianus* | -82.3991 | 42.3814 |
| *Lithobates catesbeianus* | -80.4539 | 42.5894 |
| *Lithobates catesbeianus* | -80.2712 | 42.564 |
| *Lithobates catesbeianus* | -77.4622 | 46.0699 |
| *Lithobates catesbeianus* | -78.3714 | 44.0501 |
| *Lithobates catesbeianus* | -74.8259 | 45.3404 |
| *Lithobates catesbeianus* | -79.2588 | 43.1173 |
| *Lithobates catesbeianus* | -77.139 | 44.8904 |
| *Lithobates catesbeianus* | -79.8819 | 43.1097 |
| *Lithobates catesbeianus* | -76.0496 | 44.5667 |
| *Lithobates catesbeianus* | -81.8574 | 42.2819 |
| *Lithobates catesbeianus* | -80.3713 | 43.2536 |
| *Lithobates catesbeianus* | -80.3371 | 43.232 |
| *Lithobates catesbeianus* | -80.4044 | 45.5997 |
| *Lithobates catesbeianus* | -83.0747 | 42.2528 |
| *Lithobates catesbeianus* | -82.3954 | 42.3675 |
| *Lithobates catesbeianus* | -77.494 | 44.067 |
| *Lithobates catesbeianus* | -78.6032 | 43.8983 |
| *Lithobates catesbeianus* | -82.1975 | 35.43194 |
| *Lithobates catesbeianus* | -82.9959 | 41.41493 |
| *Lithobates catesbeianus* | -83.9864 | 39.17542 |
| *Lithobates catesbeianus* | -82.5318 | 38.95933 |
| *Lithobates catesbeianus* | -84.631 | 39.31286 |
| *Lithobates catesbeianus* | -83.3387 | 40.14034 |
| *Lithobates catesbeianus* | -97.0789 | 37.1685 |
| *Lithobates catesbeianus* | 140.13 | 39.74 |
| *Lithobates catesbeianus* | -84.1051 | 39.42925 |
| *Lithobates catesbeianus* | -84.2999 | 39.24714 |
| *Lithobates catesbeianus* | -82.9507 | 39.22325 |
| *Lithobates catesbeianus* | -84.4615 | 38.9645 |
| *Lithobates catesbeianus* | -117.86 | 34.31205 |
| *Lithobates catesbeianus* | -119.11 | 35.16587 |
| *Lithobates catesbeianus* | -116.781 | 33.80422 |
| *Lithobates catesbeianus* | -117.728 | 33.78643 |
| *Lithobates catesbeianus* | -122.312 | 39.10669 |
| *Lithobates catesbeianus* | -117.946 | 33.93198 |
| *Lithobates catesbeianus* | -116.355 | 35.02309 |
| *Lithobates catesbeianus* | -117.111 | 32.78496 |
| *Lithobates catesbeianus* | -121.966 | 39.01258 |
| *Lithobates catesbeianus* | -78.2833 | 45.38333 |
| *Lithobates catesbeianus* | -78.75 | 45.6 |
| *Lithobates catesbeianus* | -77.2833 | 45 |
| *Lithobates catesbeianus* | -79.6184 | 45.01184 |
| *Lithobates catesbeianus* | -79.0307 | 45.44274 |
| *Lithobates catesbeianus* | -94.8073 | 34.12761 |
| *Lithobates catesbeianus* | -98.7075 | 34.71677 |
| *Lithobates catesbeianus* | -94.7838 | 35.63469 |
| *Lithobates catesbeianus* | -99.2694 | 36.43734 |
| *Lithobates catesbeianus* | -99.2788 | 34.88536 |
| *Lithobates catesbeianus* | -98.7656 | 34.75676 |
| *Lithobates catesbeianus* | -95.3325 | 34.9251 |
| *Lithobates catesbeianus* | -95.2973 | 34.9272 |
| *Lithobates catesbeianus* | -94.5648 | 33.88731 |
| *Lithobates catesbeianus* | -94.4894 | 34.05571 |
| *Lithobates catesbeianus* | -96.7428 | 35.24728 |
| *Lithobates catesbeianus* | -95.2363 | 36.74548 |
| *Lithobates catesbeianus* | -98.363 | 34.66457 |
| *Lithobates catesbeianus* | -94.8871 | 35.88679 |
| *Lithobates catesbeianus* | -95.3216 | 34.03011 |
| *Lithobates catesbeianus* | -94.9136 | 35.92349 |
| *Lithobates catesbeianus* | -94.9312 | 35.92049 |
| *Lithobates catesbeianus* | -94.8957 | 36.05119 |
| *Lithobates catesbeianus* | -78.6388 | 35.7801 |
| *Lithobates catesbeianus* | -78.7283 | 35.8428 |
| *Lithobates catesbeianus* | -79.1799 | 35.48 |
| *Lithobates catesbeianus* | -76.9022 | 36.0746 |
| *Lithobates catesbeianus* | -78.3641 | 34.157 |
| *Lithobates catesbeianus* | -78.474 | 34.434 |
| *Lithobates catesbeianus* | -78.5962 | 34.141 |
| *Lithobates catesbeianus* | -79.3869 | 34.8652 |
| *Lithobates catesbeianus* | -78.9511 | 35.9901 |
| *Lithobates catesbeianus* | -81.5246 | 36.0867 |
| *Lithobates catesbeianus* | -76.4637 | 35.768 |
| *Lithobates catesbeianus* | -77.9008 | 34.5791 |
| *Lithobates catesbeianus* | -80.7592 | 35.4963 |
| *Lithobates catesbeianus* | -74.4937 | 39.5162 |
| *Lithobates catesbeianus* | -75.4796 | 38.5275 |
| *Lithobates catesbeianus* | -74.7169 | 40.0792 |
| *Lithobates catesbeianus* | -83.258 | 36.5142 |
| *Lithobates catesbeianus* | -80.4189 | 34.6711 |
| *Lithobates catesbeianus* | -81.243 | 34.8967 |
| *Lithobates catesbeianus* | -75.6488 | 39.4475 |
| *Lithobates catesbeianus* | -76.0551 | 36.6154 |
| *Lithobates catesbeianus* | -77.1663 | 38.6334 |
| *Lithobates catesbeianus* | -77.1889 | 37.3359 |
| *Lithobates catesbeianus* | -77.6122 | 37.7602 |
| *Lithobates catesbeianus* | -77.6798 | 37.3613 |
| *Lithobates catesbeianus* | -118.361 | 33.35204 |
| *Lithobates catesbeianus* | -81.6281 | 40.93326 |
| *Lithobates catesbeianus* | -91.7082 | 37.96701 |
| *Lithobates catesbeianus* | -76.7975 | 39.02705 |
| *Lithobates catesbeianus* | -96.3612 | 30.63911 |
| *Lithobates catesbeianus* | -101.701 | 33.53576 |
| *Lithobates catesbeianus* | -95.4055 | 32.75171 |
| *Lithobates catesbeianus* | -98.7998 | 31.21125 |
| *Lithobates catesbeianus* | -98.9066 | 34.03952 |
| *Lithobates catesbeianus* | -96.8074 | 33.63804 |
| *Lithobates catesbeianus* | -98.387 | 33.90852 |
| *Lithobates catesbeianus* | -98.5568 | 33.8295 |
| *Lithobates catesbeianus* | -98.515 | 33.85167 |
| *Lithobates catesbeianus* | -97.9232 | 33.65158 |
| *Lithobates catesbeianus* | -94.0364 | 29.87996 |
| *Lithobates catesbeianus* | -71.6008 | 43.02028 |
| *Lithobates catesbeianus* | -96.3699 | 30.67437 |
| *Lithobates catesbeianus* | -95.1573 | 31.40773 |
| *Lithobates catesbeianus* | -94.7678 | 31.49889 |
| *Lithobates catesbeianus* | -122.339 | 47.7234 |
| *Lithobates catesbeianus* | -121.905 | 45.6957 |
| *Lithobates catesbeianus* | -122.395 | 48.7915 |
| *Lithobates catesbeianus* | -122.492 | 47.2109 |
| *Lithobates catesbeianus* | -122.959 | 47.2654 |
| *Lithobates catesbeianus* | -122.76 | 47.0343 |
| *Lithobates catesbeianus* | -122.325 | 48.4757 |
| *Lithobates catesbeianus* | -157.804 | 21.3145 |
| *Lithobates catesbeianus* | -158.007 | 21.4347 |
| *Lithobates catesbeianus* | 11.07812 | 43.8056 |
| *Lithobates catesbeianus* | -109.795 | 23.44234 |
| *Lithobates catesbeianus* | -94.1778 | 30.34889 |
| *Lithobates catesbeianus* | -114.664 | 33.41603 |
| *Lithobates catesbeianus* | -117.228 | 34.34454 |
| *Lithobates catesbeianus* | -120.795 | 38.83473 |
| *Lithobates catesbeianus* | -121.282 | 38.0527 |
| *Lithobates catesbeianus* | -68.2 | 44.38 |
| *Lithobates catesbeianus* | -80.15 | 41.72 |
| *Lithobates catesbeianus* | -79.85 | 41.97 |
| *Lithobates catesbeianus* | -74.97 | 40.13 |
| *Lithobates catesbeianus* | -73.12 | 40.84 |
| *Lithobates catesbeianus* | -72.82 | 40.91 |
| *Lithobates catesbeianus* | -67.13 | 18.2 |
| *Lithobates catesbeianus* | -96.7701 | 33.74009 |
| *Lithobates catesbeianus* | -95.1101 | 38.93998 |
| *Lithobates catesbeianus* | -96.1801 | 38.74998 |
| *Lithobates catesbeianus* | -115.301 | 36.35993 |
| *Lithobates catesbeianus* | -71.4 | 43.1 |
| *Lithobates catesbeianus* | -96.2137 | 30.56111 |
| *Lithobates catesbeianus* | -78.67 | 48.5 |
| *Lithobates catesbeianus* | -80.1 | 40.32 |
| *Lithobates catesbeianus* | -79.2796 | 38.82001 |
| *Lithobates catesbeianus* | -79.1096 | 38.94 |
| *Lithobates catesbeianus* | -120.555 | 34.67772 |
| *Lithobates catesbeianus* | -121.455 | 39.14588 |
| *Lithobates catesbeianus* | -85.8271 | 30.77548 |
| *Lithobates catesbeianus* | -82.4609 | 29.04914 |
| *Lithobates catesbeianus* | -81.4546 | 27.9209 |
| *Lithobates catesbeianus* | -82.488 | 28.84449 |
| *Lithobates catesbeianus* | -84.1962 | 30.3719 |
| *Lithobates catesbeianus* | -87.5122 | 30.8856 |
| *Lithobates catesbeianus* | -82.3054 | 30.39198 |
| *Lithobates catesbeianus* | -84.2751 | 30.21027 |
| *Lithobates catesbeianus* | -82.2534 | 29.56551 |
| *Lithobates catesbeianus* | -76.4513 | 42.47935 |
| *Lithobates catesbeianus* | -76.4532 | 42.48045 |
| *Lithobates catesbeianus* | -120.434 | 39.69603 |
| *Lithobates catesbeianus* | -76.2283 | 4.97417 |
| *Lithobates catesbeianus* | -98.4083 | 33.62715 |
| *Lithobates catesbeianus* | -69.919 | 45.30798 |
| *Lithobates catesbeianus* | -80 | 37.3483 |
| *Lithobates catesbeianus* | -73.1 | 41.356 |
| *Lithobates catesbeianus* | -94.6416 | 33.74901 |
| *Lithobates catesbeianus* | -94.6063 | 34.72494 |
| *Lithobates catesbeianus* | -94.642 | 33.73319 |
| *Lithobates catesbeianus* | 5.13001 | 51.14375 |
| *Lithobates catesbeianus* | 5.13004 | 51.14476 |
| *Lithobates catesbeianus* | 5.12866 | 51.14358 |
| *Lithobates catesbeianus* | 5.13017 | 51.14442 |
| *Lithobates catesbeianus* | 5.12995 | 51.14516 |
| *Lithobates catesbeianus* | -94.2508 | 31.38478 |
| *Lithobates catesbeianus* | -90.1215 | 29.78478 |
| *Lithobates catesbeianus* | -122.184 | 37.42961 |
| *Lithobates catesbeianus* | -97.4713 | 35.51705 |
| *Lithobates catesbeianus* | -95.4002 | 34.50549 |
| *Lithobates catesbeianus* | -78.5333 | 45.59167 |
| *Lithobates catesbeianus* | -76.2 | 44.88333 |
| *Lithobates catesbeianus* | -78.15 | 44.1666 |
| *Lithobates catesbeianus* | -78.8167 | 45.63333 |
| *Lithobates catesbeianus* | -79.8337 | 43.15028 |
| *Lithobates catesbeianus* | -78.7167 | 45.01666 |
| *Lithobates catesbeianus* | -79.2833 | 45.21666 |
| *Lithobates catesbeianus* | -77.8666 | 44.4 |
| *Lithobates catesbeianus* | -75.9912 | 45.73448 |
| *Lithobates catesbeianus* | -78.2347 | 34.5486 |
| *Lithobates catesbeianus* | -122.596 | 38.34142 |
| *Lithobates catesbeianus* | 5.01395 | 51.27039 |
| *Lithobates catesbeianus* | -79.317 | 46.39993 |
| *Lithobates catesbeianus* | -123.204 | 39.26525 |
| *Lithobates catesbeianus* | -88.9192 | 37.35859 |
| *Lithobates catesbeianus* | -83 | 40 |
| *Lithobates catesbeianus* | -121.91 | 39.692 |
| *Lithobates catesbeianus* | 138.604 | 36.2136 |
| *Lithobates catesbeianus* | -155.654 | 20.00954 |
| *Lithobates catesbeianus* | -110.448 | 31.42345 |
| *Lithobates catesbeianus* | -119.8 | 39.533 |
| *Lithobates catesbeianus* | -115.217 | 37.6 |
| *Lithobates catesbeianus* | -114.583 | 36.6667 |
| *Lithobates catesbeianus* | -114.717 | 36.7166 |
| *Lithobates catesbeianus* | -119.279 | 39.6644 |
| *Lithobates catesbeianus* | -123.014 | 44.04812 |
| *Lithobates catesbeianus* | -114.177 | 36.02606 |
| *Lithobates catesbeianus* | -111.797 | 32.69487 |
| *Lithobates catesbeianus* | -108.079 | 33.34888 |
| *Lithobates catesbeianus* | -117.417 | 33.894 |
| *Lithobates catesbeianus* | -157 | 21.13511 |
| *Lithobates catesbeianus* | -122.489 | 37.73587 |
| *Lithobates catesbeianus* | -110.676 | 32.03562 |
| *Lithobates catesbeianus* | -109.171 | 32.41147 |
| *Lithobates catesbeianus* | -123.641 | 39.74988 |
| *Lithobates catesbeianus* | -121.126 | 37.72243 |
| *Lithobates catesbeianus* | -123.324 | 44.4097 |
| *Lithobates catesbeianus* | -114.489 | 32.8186 |
| *Lithobates catesbeianus* | -114.005 | 36.2581 |
| *Lithobates catesbeianus* | -114.488 | 34.745 |
| *Lithobates catesbeianus* | -116.314 | 36.42051 |
| *Lithobates catesbeianus* | -71.6719 | 41.915 |
| *Lithobates catesbeianus* | -85.255 | 36.48345 |
| *Lithobates catesbeianus* | -76.6091 | 36.0607 |
| *Lithobates catesbeianus* | -83.186 | 35.05256 |
| *Lithobates catesbeianus* | -98.1599 | 38.73969 |
| *Lithobates catesbeianus* | -78.3916 | 39.49118 |
| *Lithobates catesbeianus* | -78.38 | 34.2 |
| *Lithobates catesbeianus* | -79.021 | 33.81699 |
| *Lithobates catesbeianus* | -96.9893 | 37.24028 |
| *Lithobates catesbeianus* | -77.26 | 38.9 |
| *Lithobates catesbeianus* | -82.6766 | 34.70596 |
| *Lithobates catesbeianus* | -65.4256 | 44.62591 |
| *Lithobates catesbeianus* | -79.0456 | 36.41477 |
| *Lithobates catesbeianus* | -72.5891 | 41.5988 |
| *Lithobates catesbeianus* | -90.7236 | 38.4736 |
| *Lithobates catesbeianus* | -95.3123 | 39.56553 |
| *Lithobates catesbeianus* | -77.53 | 37.64 |
| *Lithobates catesbeianus* | -77.51 | 37.7 |
| *Lithobates catesbeianus* | -77.5 | 37.32 |
| *Lithobates catesbeianus* | -77.6 | 37.72 |
| *Lithobates catesbeianus* | -76.9402 | 37.742 |
| *Lithobates catesbeianus* | -77.53 | 37.7 |
| *Lithobates catesbeianus* | -83.1728 | 36.6332 |
| *Lithobates catesbeianus* | -80.4006 | 36.7745 |
| *Lithobates catesbeianus* | -89.2364 | 37.8462 |
| *Lithobates catesbeianus* | -76.985 | 37.6514 |
| *Lithobates catesbeianus* | -76.9347 | 37.6208 |
| *Lithobates catesbeianus* | -111.087 | 31.40485 |
| *Lithobates catesbeianus* | -76.518 | 37.66583 |
| *Lithobates catesbeianus* | -82.4932 | 38.6122 |
| *Lithobates catesbeianus* | -82.0911 | 39.3682 |
| *Lithobates catesbeianus* | -83.5002 | 32.7504 |
| *Lithobates catesbeianus* | -119.751 | 37.2502 |
| *Lithobates catesbeianus* | -120.359 | 38.20009 |
| *Lithobates catesbeianus* | -92.8484 | 40.46533 |
| *Lithobates catesbeianus* | -121.491 | 36.32264 |
| *Lithobates catesbeianus* | -123.039 | 43.79754 |
| *Lithobates catesbeianus* | -72.5565 | 41.79675 |
| *Lithobates catesbeianus* | -119.856 | 37.88029 |
| *Lithobates catesbeianus* | -116.272 | 36.4009 |
| *Lithobates catesbeianus* | -119.709 | 37.01769 |
| *Lithobates catesbeianus* | -70.5394 | 41.63995 |
| *Lithobates catesbeianus* | -118.852 | 39.47345 |
| *Lithobates catesbeianus* | -106.833 | 34.40414 |
| *Lithobates catesbeianus* | -121.069 | 36.46641 |
| *Lithobates catesbeianus* | -117.291 | 34.57961 |
| *Lithobates catesbeianus* | -120.31 | 37.96665 |
| *Lithobates catesbeianus* | -92.6662 | 32.37151 |
| *Lithobates catesbeianus* | -89.5607 | 40.87673 |
| *Lithobates catesbeianus* | -84.6973 | 45.58075 |
| *Lithobates catesbeianus* | -74.4571 | 45.85129 |
| *Lithobates catesbeianus* | -122.29 | 47.65 |
| *Lithobates catesbeianus* | -122.914 | 45.26772 |
| *Lithobates catesbeianus* | -121.719 | 37.34467 |
| *Lithobates catesbeianus* | -118.427 | 33.96581 |
| *Lithobates catesbeianus* | -118.409 | 34.13129 |
| *Lithobates catesbeianus* | -73.0607 | 43.61729 |
| *Lithobates catesbeianus* | -76.9446 | 38.91256 |
| *Lithobates catesbeianus* | -121.744 | 37.29729 |
| *Lithobates catesbeianus* | -70.7505 | 42.1791 |
| *Lithobates catesbeianus* | -77.0522 | 35.5466 |
| *Lithobates catesbeianus* | -71.5025 | 41.98694 |
| *Lithobates catesbeianus* | -71.6364 | 41.44083 |
| *Lithobates catesbeianus* | -75.551 | 41.38046 |
| *Lithobates catesbeianus* | -95.235 | 38.97141 |
| *Lithobates catesbeianus* | -122.963 | 49.25262 |
| *Lithobates catesbeianus* | -122.963 | 49.25254 |
| *Lithobates catesbeianus* | -71.2253 | 42.42968 |
| *Lithobates catesbeianus* | -96.8054 | 28.30471 |
| *Lithobates catesbeianus* | -80.2421 | 43.50084 |
| *Lithobates catesbeianus* | -118.88 | 34.23851 |
| *Lithobates catesbeianus* | -98.1514 | 29.62305 |
| *Lithobates catesbeianus* | -94.3897 | 29.57364 |
| *Lithobates catesbeianus* | -117.032 | 32.84091 |
| *Lithobates catesbeianus* | -74.4643 | 40.71353 |
| *Lithobates catesbeianus* | 102.7037 | 25.05159 |
| *Lithobates catesbeianus* | -120.506 | 46.60207 |
| *Lithobates catesbeianus* | -98.86 | 28.95465 |
| *Lithobates catesbeianus* | -96.0916 | 29.54749 |
| *Lithobates catesbeianus* | -81.0292 | 41.74932 |
| *Lithobates catesbeianus* | -92.5904 | 42.72194 |
| *Lithobates catesbeianus* | -122.924 | 45.36614 |
| *Lithobates catesbeianus* | -94.5551 | 29.6071 |
| *Lithobates catesbeianus* | -74.4532 | 40.41064 |
| *Lithobates catesbeianus* | -122.069 | 37.05518 |
| *Lithobates catesbeianus* | -122.378 | 48.32522 |
| *Lithobates catesbeianus* | -97.8658 | 30.5826 |
| *Lithobates catesbeianus* | -93.1708 | 36.04049 |
| *Lithobates catesbeianus* | -73.9531 | 41.49614 |
| *Lithobates catesbeianus* | -90.15 | 38.80585 |
| *Lithobates catesbeianus* | -76.9418 | 38.9127 |
| *Lithobates catesbeianus* | -75.2474 | 44.59171 |
| *Lithobates catesbeianus* | -95.9398 | 29.78949 |
| *Lithobates catesbeianus* | -123.444 | 48.51014 |
| *Lithobates catesbeianus* | -96.6951 | 32.60061 |
| *Lithobates catesbeianus* | -96.207 | 30.5607 |
| *Lithobates catesbeianus* | -121.469 | 37.0904 |
| *Lithobates catesbeianus* | -88.1871 | 42.34943 |
| *Lithobates catesbeianus* | -121.975 | 36.89418 |
| *Lithobates catesbeianus* | 126.4664 | 36.79609 |
| *Lithobates catesbeianus* | -106.306 | 31.64283 |
| *Lithobates catesbeianus* | -64.9521 | 45.594 |
| *Lithobates catesbeianus* | -70.7209 | 43.17174 |
| *Lithobates catesbeianus* | -76.7027 | 38.78466 |
| *Lithobates catesbeianus* | -71.151 | 42.38896 |
| *Lithobates catesbeianus* | -68.2081 | 44.36257 |
| *Lithobates catesbeianus* | -76.9258 | 40.4777 |
| *Lithobates catesbeianus* | -76.4779 | 42.32557 |
| *Lithobates catesbeianus* | -95.1703 | 43.42996 |
| *Lithobates catesbeianus* | -77.2597 | 42.08067 |
| *Lithobates catesbeianus* | -76.5461 | 42.396 |
| *Lithobates catesbeianus* | -97.46 | 35.18165 |
| *Lithobates catesbeianus* | -74.8781 | 40.29212 |
| *Lithobates catesbeianus* | -76.1262 | 41.05672 |
| *Lithobates catesbeianus* | -68.2502 | 44.3236 |
| *Lithobates catesbeianus* | -97.4266 | 35.24413 |
| *Lithobates catesbeianus* | -97.2226 | 35.0432 |
| *Lithobates catesbeianus* | -122.265 | 37.90979 |
| *Lithobates catesbeianus* | -75.4724 | 5.1925 |
| *Lithobates catesbeianus* | -74.7803 | 7.76833 |
| *Lithobates catesbeianus* | -74.7801 | 7.76833 |
| *Lithobates catesbeianus* | -82.261 | 29.65798 |
| *Lithobates catesbeianus* | -81.6609 | 29.45339 |
| *Lithobates catesbeianus* | -84.3457 | 30.48003 |
| *Lithobates catesbeianus* | -84.2969 | 30.41348 |
| *Lithobates catesbeianus* | -85.2198 | 30.8713 |
| *Lithobates catesbeianus* | -86.7559 | 33.10091 |
| *Lithobates catesbeianus* | -81.7502 | 29.25461 |
| *Lithobates catesbeianus* | -84.3362 | 30.2547 |
| *Lithobates catesbeianus* | -81.7627 | 29.43636 |
| *Lithobates catesbeianus* | -81.9773 | 29.69858 |
| *Lithobates catesbeianus* | -82.427 | 29.6404 |
| *Lithobates catesbeianus* | 5.13004 | 51.14407 |
| *Lithobates catesbeianus* | 5.1303 | 51.14504 |
| *Lithobates catesbeianus* | 5.12751 | 51.14395 |
| *Lithobates catesbeianus* | 5.0161 | 51.27201 |
| *Lithobates catesbeianus* | -95.1568 | 31.5487 |
| *Lithobates catesbeianus* | -77.4014 | 44.69556 |
| *Lithobates catesbeianus* | -79.1309 | 44.19029 |
| *Lithobates catesbeianus* | -61.1465 | 46.21002 |
| *Lithobates catesbeianus* | -67.0719 | 45.12658 |
| *Lithobates catesbeianus* | -76.6978 | 44.68347 |
| *Lithobates catesbeianus* | -79.8 | 46.2 |
| *Lithobates catesbeianus* | -79.8033 | 43.64571 |
| *Lithobates catesbeianus* | -79.5043 | 33.2188 |
| *Lithobates catesbeianus* | -82.95 | 40.1833 |
| *Lithobates catesbeianus* | -73.0089 | 41.3525 |
| *Lithobates catesbeianus* | -72.9286 | 41.3081 |
| *Lithobates catesbeianus* | -73.2946 | 41.20142 |
| *Lithobates catesbeianus* | -72.5058 | 41.50933 |
| *Lithobates catesbeianus* | -73.1172 | 41.4339 |
| *Lithobates catesbeianus* | -71.842 | 43.71738 |
| *Lithobates catesbeianus* | -72.86 | 41.3908 |
| *Lithobates catesbeianus* | -87.268 | 36.8222 |
| *Lithobates catesbeianus* | -82.4296 | 34.7771 |
| *Lithobates catesbeianus* | 138.888 | 36.2764 |
| *Lithobates catesbeianus* | -116.648 | 32.87888 |
| *Lithobates catesbeianus* | -116.95 | 33.04577 |
| *Lithobates catesbeianus* | -86.3408 | 41.542 |
| *Lithobates catesbeianus* | -87.09 | 41.6398 |
| *Lithobates catesbeianus* | -87.0372 | 41.6621 |
| *Lithobates catesbeianus* | -85.977 | 42.5881 |
| *Lithobates catesbeianus* | -85.2873 | 42.0708 |
| *Lithobates catesbeianus* | -85.5155 | 42.6843 |
| *Lithobates catesbeianus* | -85.4339 | 42.596 |
| *Lithobates catesbeianus* | -78.4946 | 43.1225 |
| *Lithobates catesbeianus* | -78.3621 | 42.4234 |
| *Lithobates catesbeianus* | -78.6076 | 42.9609 |
| *Lithobates catesbeianus* | -74.8723 | 44.0901 |
| *Lithobates catesbeianus* | -77.7323 | 43.2972 |
| *Lithobates catesbeianus* | -77.7617 | 43.3086 |
| *Lithobates catesbeianus* | -81.3233 | 41.7281 |
| *Lithobates catesbeianus* | -79.2508 | 42.9339 |
| *Lithobates catesbeianus* | -81.9174 | 42.2981 |
| *Lithobates catesbeianus* | -82.4035 | 42.3681 |
| *Lithobates catesbeianus* | -80.4617 | 42.593 |
| *Lithobates catesbeianus* | -80.4451 | 42.5773 |
| *Lithobates catesbeianus* | -80.3593 | 43.2972 |
| *Lithobates catesbeianus* | -79.3288 | 44.25 |
| *Lithobates catesbeianus* | -78.9986 | 44.1417 |
| *Lithobates catesbeianus* | -78.2622 | 44.529 |
| *Lithobates catesbeianus* | -79.7783 | 44.7413 |
| *Lithobates catesbeianus* | -77.259 | 43.9049 |
| *Lithobates catesbeianus* | -77.1821 | 43.9606 |
| *Lithobates catesbeianus* | -76.7011 | 44.8657 |
| *Lithobates catesbeianus* | -79.6493 | 44.7375 |
| *Lithobates catesbeianus* | -79.0988 | 43.0051 |
| *Lithobates catesbeianus* | -79.2904 | 43.0166 |
| *Lithobates catesbeianus* | -77.8928 | 44.6427 |
| *Lithobates catesbeianus* | -80.4569 | 42.6128 |
| *Lithobates catesbeianus* | -76.5276 | 44.2632 |
| *Lithobates catesbeianus* | -79.938 | 41.5675 |
| *Lithobates catesbeianus* | -83.5103 | 35.71417 |
| *Lithobates catesbeianus* | -80.746 | 41.5745 |
| *Lithobates catesbeianus* | -84.7888 | 39.4168 |
| *Lithobates catesbeianus* | -84.3807 | 40.15092 |
| *Lithobates catesbeianus* | -82.3584 | 38.92087 |
| *Lithobates catesbeianus* | -97.8463 | 42.8068 |
| *Lithobates catesbeianus* | -98.1266 | 42.3935 |
| *Lithobates catesbeianus* | -105.75 | 37.37 |
| *Lithobates catesbeianus* | -98.7 | 34.76667 |
| *Lithobates catesbeianus* | -94.7038 | 37.0458 |
| *Lithobates catesbeianus* | -98.2412 | 38.6434 |
| *Lithobates catesbeianus* | -82.751 | 27.96555 |
| *Lithobates catesbeianus* | -101.844 | 37.1407 |
| *Lithobates catesbeianus* | -101.764 | 39.18833 |
| *Lithobates catesbeianus* | -94.6635 | 37.22215 |
| *Lithobates catesbeianus* | -121.343 | 38.35784 |
| *Lithobates catesbeianus* | -121.345 | 37.42031 |
| *Lithobates catesbeianus* | -122.275 | 38.7445 |
| *Lithobates catesbeianus* | -109.058 | 31.79849 |
| *Lithobates catesbeianus* | -121.096 | 35.56075 |
| *Lithobates catesbeianus* | -120.552 | 37.63807 |
| *Lithobates catesbeianus* | -121.447 | 38.6911 |
| *Lithobates catesbeianus* | -121.609 | 38.44248 |
| *Lithobates catesbeianus* | -123.283 | 43.59845 |
| *Lithobates catesbeianus* | -118.765 | 36.19905 |
| *Lithobates catesbeianus* | -118.94 | 36.64967 |
| *Lithobates catesbeianus* | -120.586 | 37.90227 |
| *Lithobates catesbeianus* | -121.621 | 37.16204 |
| *Lithobates catesbeianus* | -78.6388 | 35.7801 |
| *Lithobates catesbeianus* | -116.245 | 43.61821 |
| *Lithobates catesbeianus* | -76.6811 | 39.314 |
| *Lithobates catesbeianus* | -122.89 | 40.69191 |
| *Lithobates catesbeianus* | -122.614 | 38.7525 |
| *Lithobates catesbeianus* | -121.078 | 40.25193 |
| *Lithobates catesbeianus* | -95.1396 | 31.25288 |
| *Lithobates catesbeianus* | -96.3847 | 30.62778 |
| *Lithobates catesbeianus* | -74.165 | 43.96944 |
| *Lithobates catesbeianus* | -95.1535 | 31.38715 |
| *Lithobates catesbeianus* | -98.0495 | 29.98708 |
| *Lithobates catesbeianus* | -96.1671 | 32.39424 |
| *Lithobates catesbeianus* | -99.4394 | 32.235 |
| *Lithobates catesbeianus* | -97.5892 | 33.53198 |
| *Lithobates catesbeianus* | -97.5226 | 33.69471 |
| *Lithobates catesbeianus* | -98.6937 | 33.97156 |
| *Lithobates catesbeianus* | -98.4916 | 33.90849 |
| *Lithobates catesbeianus* | -98.4547 | 33.93909 |
| *Lithobates catesbeianus* | -94.4875 | 29.67167 |
| *Lithobates catesbeianus* | -95.8208 | 31.44028 |
| *Lithobates catesbeianus* | -116.627 | 43.18953 |
| *Lithobates catesbeianus* | -121.973 | 37.23151 |
| *Lithobates catesbeianus* | -110.454 | 31.42617 |
| *Lithobates catesbeianus* | -156.294 | 20.75997 |
| *Lithobates catesbeianus* | -121.317 | 45.9583 |
| *Lithobates catesbeianus* | -122.077 | 45.6053 |
| *Lithobates catesbeianus* | -110.3 | 34.98333 |
| *Lithobates catesbeianus* | -114.464 | 32.8831 |
| *Lithobates catesbeianus* | -112.029 | 34.84167 |
| *Lithobates catesbeianus* | -66.6788 | 18.473 |
| *Lithobates catesbeianus* | -115.194 | 38.16911 |
| *Lithobates catesbeianus* | -117.603 | 33.52512 |
| *Lithobates catesbeianus* | -122.846 | 42.5197 |
| *Lithobates catesbeianus* | -108.5 | 45.78329 |
| *Lithobates catesbeianus* | -114.529 | 33.54975 |
| *Lithobates catesbeianus* | -95.1839 | 43.3822 |
| *Lithobates catesbeianus* | -105.154 | 40.03388 |
| *Lithobates catesbeianus* | -122.682 | 47.1573 |
| *Lithobates catesbeianus* | -116.786 | 47.4543 |
| *Lithobates catesbeianus* | -122.88 | 47.9398 |
| *Lithobates catesbeianus* | -117.38 | 47.4708 |
| *Lithobates catesbeianus* | -88.0431 | 35.62242 |
| *Lithobates catesbeianus* | -96.2823 | 37.53167 |
| *Lithobates catesbeianus* | -95.579 | 37.29362 |
| *Lithobates catesbeianus* | -95.6837 | 37.45372 |
| *Lithobates catesbeianus* | -97.4508 | 35.21491 |
| *Lithobates catesbeianus* | -77.2389 | 34.94199 |
| *Lithobates catesbeianus* | -94.6392 | 37.1789 |
| *Lithobates catesbeianus* | -95.3012 | 37.04807 |
| *Lithobates catesbeianus* | -101.927 | 37.10567 |
| *Lithobates catesbeianus* | -100.302 | 38.78544 |
| *Lithobates catesbeianus* | -95.1172 | 38.81942 |
| *Lithobates catesbeianus* | -96.1735 | 38.57899 |
| *Lithobates catesbeianus* | -96.2942 | 38.48144 |
| *Lithobates catesbeianus* | -100.69 | 19.6206 |
| *Lithobates catesbeianus* | -94.7088 | 36.90398 |
| *Lithobates catesbeianus* | -99.6963 | 38.43275 |
| *Lithobates catesbeianus* | -94.6428 | 37.04224 |
| *Lithobates catesbeianus* | -91.9418 | 32.48211 |
| *Lithobates catesbeianus* | -83.7688 | 33.0997 |
| *Lithobates catesbeianus* | -80.02 | 40.61 |
| *Lithobates catesbeianus* | -79.23 | 40.18 |
| *Lithobates catesbeianus* | -79.24 | 40.19 |
| *Lithobates catesbeianus* | -79.91 | 41.36 |
| *Lithobates catesbeianus* | -81.0897 | 38.80001 |
| *Lithobates catesbeianus* | -80.4397 | 37.71003 |
| *Lithobates catesbeianus* | -82.32 | 27.6 |
| *Lithobates catesbeianus* | -82.84 | 32.03 |
| *Lithobates catesbeianus* | -81.1397 | 39.43 |
| *Lithobates catesbeianus* | -73.97 | 40.85 |
| *Lithobates catesbeianus* | -91.17 | 30.4 |
| *Lithobates catesbeianus* | -80.4697 | 39.09 |
| *Lithobates catesbeianus* | -81.37 | 31.97 |
| *Lithobates catesbeianus* | -76.58 | 41.76 |
| *Lithobates catesbeianus* | -75.06 | 40.47 |
| *Lithobates catesbeianus* | -75.08 | 40.27 |
| *Lithobates catesbeianus* | -155.61 | 20.1 |
| *Lithobates catesbeianus* | -75.44 | 38.14 |
| *Lithobates catesbeianus* | -116.86 | 32.65 |
| *Lithobates catesbeianus* | -78.15 | 37 |
| *Lithobates catesbeianus* | -76.59 | 36.63 |
| *Lithobates catesbeianus* | -76.44 | 36.67 |
| *Lithobates catesbeianus* | -76.82 | 36.71 |
| *Lithobates catesbeianus* | -77.24 | 38.85 |
| *Lithobates catesbeianus* | -116.305 | 33.86487 |
| *Lithobates catesbeianus* | -118.022 | 34.47751 |
| *Lithobates catesbeianus* | -118.289 | 33.78563 |
| *Lithobates catesbeianus* | -118.382 | 34.26751 |
| *Lithobates catesbeianus* | -118.073 | 34.0809 |
| *Lithobates catesbeianus* | -114.626 | 32.73103 |
| *Lithobates catesbeianus* | -118.03 | 34.05374 |
| *Lithobates catesbeianus* | -114.733 | 33.43481 |
| *Lithobates catesbeianus* | -117.87 | 34.24133 |
| *Lithobates catesbeianus* | -117.697 | 33.6235 |
| *Lithobates catesbeianus* | -117.847 | 34.31875 |
| *Lithobates catesbeianus* | -122.237 | 37.40323 |
| *Lithobates catesbeianus* | -119.311 | 34.35278 |
| *Lithobates catesbeianus* | -98.1268 | 39.98848 |
| *Lithobates catesbeianus* | -51.2164 | -30.0364 |
| *Lithobates catesbeianus* | -86.8663 | 36.31411 |
| *Lithobates catesbeianus* | -116.982 | 33.48951 |
| *Lithobates catesbeianus* | -116.947 | 32.85696 |
| *Lithobates catesbeianus* | -75.0261 | 39.4861 |
| *Lithobates catesbeianus* | -76.2418 | 40.02529 |
| *Lithobates catesbeianus* | -75.0153 | 40.0114 |
| *Lithobates catesbeianus* | -83.1775 | 35.04576 |
| *Lithobates catesbeianus* | -78.5799 | 38.69178 |
| *Lithobates catesbeianus* | -77.8126 | 39.21669 |
| *Lithobates catesbeianus* | -97.5046 | 38.7108 |
| *Lithobates catesbeianus* | -97.0301 | 37.24028 |
| *Lithobates catesbeianus* | -79 | 37.92 |
| *Lithobates catesbeianus* | -116.651 | 47.6201 |
| *Lithobates catesbeianus* | -76.7207 | 37.09039 |
| *Lithobates catesbeianus* | -84.9239 | 42.44171 |
| *Lithobates catesbeianus* | -74.0633 | 41.15972 |
| *Lithobates catesbeianus* | -75.76 | 37.57 |
| *Lithobates catesbeianus* | -76.784 | 39.05826 |
| *Lithobates catesbeianus* | -80.3881 | 37.2231 |
| *Lithobates catesbeianus* | -79.74 | 37.89 |
| *Lithobates catesbeianus* | -77.18 | 37.33 |
| *Lithobates catesbeianus* | -79.7048 | 36.5951 |
| *Lithobates catesbeianus* | -76.9178 | 37.0892 |
| *Lithobates catesbeianus* | -78.11 | 37.68 |
| *Lithobates catesbeianus* | -77.51 | 37.64 |
| *Lithobates catesbeianus* | -78.8 | 36.99 |
| *Lithobates catesbeianus* | -78.3284 | 36.62831 |
| *Lithobates catesbeianus* | -77.59 | 37.79 |
| *Lithobates catesbeianus* | -117.744 | 33.63305 |
| *Lithobates catesbeianus* | -103.623 | 29.54846 |
| *Lithobates catesbeianus* | -76.6114 | 39.92835 |
| *Lithobates catesbeianus* | -79.3028 | 42.91834 |
| *Lithobates catesbeianus* | -79.6167 | 46.11666 |
| *Lithobates catesbeianus* | -84.8776 | 38.2004 |
| *Lithobates catesbeianus* | -121.455 | 39.13021 |
| *Lithobates catesbeianus* | -120.534 | 34.6789 |
| *Lithobates catesbeianus* | -120.506 | 34.77928 |
| *Lithobates catesbeianus* | -99.9619 | 28.31646 |
| *Lithobates catesbeianus* | -98.4853 | 29.0564 |
| *Lithobates catesbeianus* | -89.3324 | 30.31769 |
| *Lithobates catesbeianus* | -88.9054 | 31.13302 |
| *Lithobates catesbeianus* | -97.723 | 33.86752 |
| *Lithobates catesbeianus* | -99.775 | 25.56 |
| *Lithobates catesbeianus* | -88.9516 | 37.314 |
| *Lithobates catesbeianus* | -121.77 | 36.91333 |
| *Lithobates catesbeianus* | -77.037 | 39.05708 |
| *Lithobates catesbeianus* | -98.677 | 34.70177 |
| *Lithobates catesbeianus* | -98.6759 | 34.71187 |
| *Lithobates catesbeianus* | -98.7253 | 34.73077 |
| *Lithobates catesbeianus* | -95.3078 | 34.8548 |
| *Lithobates catesbeianus* | -95.2948 | 34.9293 |
| *Lithobates catesbeianus* | -95.3072 | 34.9262 |
| *Lithobates catesbeianus* | -99.3116 | 35.29155 |
| *Lithobates catesbeianus* | -94.6041 | 34.7723 |
| *Lithobates catesbeianus* | -94.5771 | 33.89751 |
| *Lithobates catesbeianus* | -100.002 | 36.85257 |
| *Lithobates catesbeianus* | -98.3173 | 34.55058 |
| *Lithobates catesbeianus* | -95.0218 | 35.86119 |
| *Lithobates catesbeianus* | -96.1209 | 34.90179 |
| *Lithobates catesbeianus* | -83.6569 | 36.56894 |
| *Lithobates catesbeianus* | -96.4104 | 36.31007 |
| *Lithobates catesbeianus* | -98.0071 | 34.18009 |
| *Lithobates catesbeianus* | -96.1101 | 36.12749 |
| *Lithobates catesbeianus* | -97.185 | 35.50097 |
| *Lithobates catesbeianus* | -94.9704 | 36.93607 |
| *Lithobates catesbeianus* | -93.665 | 34.39569 |
| *Lithobates catesbeianus* | -95.1886 | 35.67961 |
| *Lithobates catesbeianus* | -95.9881 | 34.299 |
| *Lithobates catesbeianus* | -81.6466 | 36.1667 |
| *Lithobates catesbeianus* | -80.2396 | 36.3496 |
| *Lithobates catesbeianus* | -80.24 | 36.3498 |
| *Lithobates catesbeianus* | -79.4146 | 34.8745 |
| *Lithobates catesbeianus* | -75.6665 | 35.9898 |
| *Lithobates catesbeianus* | -75.8514 | 42.3854 |
| *Lithobates catesbeianus* | -80.9814 | 35.2953 |
| *Lithobates catesbeianus* | -81.0628 | 35.3518 |
| *Lithobates catesbeianus* | -79.4033 | 34.8495 |
| *Lithobates catesbeianus* | -75.4617 | 39.5052 |
| *Lithobates catesbeianus* | -80.8765 | 36.5342 |
| *Lithobates catesbeianus* | -79.3722 | 35.2557 |
| *Lithobates catesbeianus* | -79.0269 | 36.0321 |
| *Lithobates catesbeianus* | -75.8513 | 42.3857 |
| *Lithobates catesbeianus* | -77.1325 | 37.4767 |
| *Lithobates catesbeianus* | -77.0244 | 37.4881 |
| *Lithobates catesbeianus* | -77.1311 | 37.4428 |
| *Lithobates catesbeianus* | -77.4842 | 37.6008 |
| *Lithobates catesbeianus* | -77.4303 | 37.6172 |
| *Lithobates catesbeianus* | -81.4734 | 31.2382 |
| *Lithobates catesbeianus* | -80.1501 | 37.1346 |
| *Lithobates catesbeianus* | -77.6095 | 34.6339 |
| *Lithobates catesbeianus* | -77.1888 | 37.3359 |
| *Lithobates catesbeianus* | -75.8722 | 5.97694 |
| *Lithobates catesbeianus* | -75.917 | 46.033 |
| *Lithobates catesbeianus* | -80.267 | 43.25 |
| *Lithobates catesbeianus* | -77.25 | 44.15 |
| *Lithobates catesbeianus* | -65.283 | 44.383 |
| *Lithobates catesbeianus* | -80.222 | 42.575 |
| *Lithobates catesbeianus* | -75.713 | 45.26 |
| *Lithobates catesbeianus* | -76.333 | 44.617 |
| *Lithobates catesbeianus* | -76.333 | 44.6 |
| *Lithobates catesbeianus* | -75.617 | 45.6 |
| *Lithobates catesbeianus* | -76.833 | 44.733 |
| *Lithobates catesbeianus* | -75.793 | 45.477 |
| *Lithobates catesbeianus* | -76.113 | 45.604 |
| *Lithobates catesbeianus* | -76.077 | 45.596 |
| *Lithobates catesbeianus* | -75.302 | 45.544 |
| *Lithobates catesbeianus* | -75.71 | 44.665 |
| *Lithobates catesbeianus* | -76.102 | 45.599 |
| *Lithobates catesbeianus* | -76.073 | 45.594 |
| *Lithobates catesbeianus* | -75.95 | 46.033 |
| *Lithobates catesbeianus* | -75.7 | 45.367 |
| *Lithobates catesbeianus* | -67.067 | 45.133 |
| *Lithobates catesbeianus* | -75.794 | 45.375 |
| *Lithobates catesbeianus* | -81.136 | 44.674 |
| *Lithobates catesbeianus* | -75.65 | 45.469 |
| *Lithobates catesbeianus* | -76.184 | 45.92 |
| *Lithobates catesbeianus* | -80.383 | 45.8 |
| *Lithobates catesbeianus* | -80.317 | 42.683 |
| *Lithobates catesbeianus* | -75.633 | 45 |
| *Lithobates catesbeianus* | -67.05 | 45.067 |
| *Lithobates catesbeianus* | -84.933 | 46.183 |
| *Lithobates catesbeianus* | -81.633 | 46.217 |
| *Lithobates catesbeianus* | -76.95 | 44.667 |
| *Lithobates catesbeianus* | -63.267 | 44.933 |
| *Lithobates catesbeianus* | -75.913 | 45.508 |
| *Lithobates catesbeianus* | -103.775 | 29.26183 |
| *Lithobates catesbeianus* | -81.5457 | 41.24788 |
| *Lithobates catesbeianus* | -72.3126 | 41.8235 |
| *Lithobates catesbeianus* | -78.7484 | 35.79734 |
| *Lithobates catesbeianus* | -76.2006 | 38.41932 |
| *Lithobates catesbeianus* | -121.686 | 37.28444 |
| *Lithobates catesbeianus* | -88.1742 | 41.86045 |
| *Lithobates catesbeianus* | -94.8449 | 37.25086 |
| *Lithobates catesbeianus* | -71.97 | 42.7833 |
| *Lithobates catesbeianus* | -70.7833 | 42.6319 |
| *Lithobates catesbeianus* | -71.6545 | 42.2961 |
| *Lithobates catesbeianus* | -71.7375 | 43.3087 |
| *Lithobates catesbeianus* | -77.0861 | 38.7079 |
| *Lithobates catesbeianus* | -73.2992 | 42.3828 |
| *Lithobates catesbeianus* | -73.0822 | 42.7406 |
| *Lithobates catesbeianus* | -83.806 | 42.27576 |
| *Lithobates catesbeianus* | -121.336 | 37.43331 |
| *Lithobates catesbeianus* | -121.371 | 37.75437 |
| *Lithobates catesbeianus* | -96.801 | 32.78 |
| *Lithobates catesbeianus* | -92.4642 | 30.18041 |
| *Lithobates catesbeianus* | -116.589 | 34.94606 |
| *Lithobates catesbeianus* | -122.346 | 40.84939 |
| *Lithobates catesbeianus* | -122.276 | 37.95698 |
| *Lithobates catesbeianus* | -116.383 | 35.03739 |
| *Lithobates catesbeianus* | -117.295 | 34.5489 |
| *Lithobates catesbeianus* | -123.305 | 39.30558 |
| *Lithobates catesbeianus* | -74.4078 | 40.65087 |
| *Lithobates catesbeianus* | -84.4119 | 42.04092 |
| *Lithobates catesbeianus* | -96.039 | 39.06719 |
| *Lithobates catesbeianus* | -74.7881 | 39.9928 |
| *Lithobates catesbeianus* | -92.7103 | 32.28839 |
| *Lithobates catesbeianus* | -119.57 | 37.31398 |
| *Lithobates catesbeianus* | -119.569 | 37.31476 |
| *Lithobates catesbeianus* | -97.9472 | 37.68007 |
| *Lithobates catesbeianus* | -118.411 | 34.11808 |
| *Lithobates catesbeianus* | -123.02 | 40.29351 |
| *Lithobates catesbeianus* | -118.829 | 34.12904 |
| *Lithobates catesbeianus* | -106.195 | 35.5496 |
| *Lithobates catesbeianus* | -73.2171 | 43.6912 |
| *Lithobates catesbeianus* | -77.0365 | 39.05933 |
| *Lithobates catesbeianus* | -81.3966 | 46.01878 |
| *Lithobates catesbeianus* | -104.989 | 40.17195 |
| *Lithobates catesbeianus* | -97.1173 | 32.79293 |
| *Lithobates catesbeianus* | -121.455 | 37.07205 |
| *Lithobates catesbeianus* | -75.7241 | 45.33491 |
| *Lithobates catesbeianus* | -98.6732 | 33.66649 |
| *Lithobates catesbeianus* | -115.71 | 33.0827 |
| *Lithobates catesbeianus* | -68.0239 | 44.60727 |
| *Lithobates catesbeianus* | -76.595 | 39.24337 |
| *Lithobates catesbeianus* | -78.3289 | 43.12147 |
| *Lithobates catesbeianus* | -77.247 | 38.99565 |
| *Lithobates catesbeianus* | -76.4997 | 44.36677 |
| *Lithobates catesbeianus* | -121.191 | 38.9876 |
| *Lithobates catesbeianus* | -97.1557 | 33.20064 |
| *Lithobates catesbeianus* | -122.712 | 47.07266 |
| *Lithobates catesbeianus* | -75.8212 | 45.06697 |
| *Lithobates catesbeianus* | -79.5057 | 40.5919 |
| *Lithobates catesbeianus* | -72.6341 | 41.70543 |
| *Lithobates catesbeianus* | -76.4504 | 40.05947 |
| *Lithobates catesbeianus* | -98.1333 | 34.03083 |
| *Lithobates catesbeianus* | -83.1039 | 40.12224 |
| *Lithobates catesbeianus* | -121.755 | 37.27095 |
| *Lithobates catesbeianus* | -95.9031 | 36.22345 |
| *Lithobates catesbeianus* | -97.1776 | 30.2776 |
| *Lithobates catesbeianus* | -115.021 | 36.10234 |
| *Lithobates catesbeianus* | -116.273 | 35.9797 |
| *Lithobates catesbeianus* | -97.654 | 30.21956 |
| *Lithobates catesbeianus* | -94.7028 | 31.33003 |
| *Lithobates catesbeianus* | -73.14 | 44.15855 |
| *Lithobates catesbeianus* | -95.4533 | 29.76405 |
| *Lithobates catesbeianus* | -97.4831 | 32.84638 |
| *Lithobates catesbeianus* | -122.654 | 38.45253 |
| *Lithobates catesbeianus* | -122.548 | 39.29861 |
| *Lithobates catesbeianus* | -118.262 | 34.11132 |
| *Lithobates catesbeianus* | -98.1139 | 30.19859 |
| *Lithobates catesbeianus* | -82.1438 | 39.95651 |
| *Lithobates catesbeianus* | -96.3795 | 29.68561 |
| *Lithobates catesbeianus* | -97.1176 | 33.11869 |
| *Lithobates catesbeianus* | -117.037 | 33.35233 |
| *Lithobates catesbeianus* | -92.8968 | 44.97133 |
| *Lithobates catesbeianus* | -97.7685 | 29.70442 |
| *Lithobates catesbeianus* | -96.0885 | 29.60609 |
| *Lithobates catesbeianus* | -114.714 | 36.71147 |
| *Lithobates catesbeianus* | -97.1222 | 32.78849 |
| *Lithobates catesbeianus* | -122.254 | 37.20102 |
| *Lithobates catesbeianus* | -97.089 | 37.65036 |
| *Lithobates catesbeianus* | -72.509 | 42.73644 |
| *Lithobates catesbeianus* | -98.5355 | 33.87461 |
| *Lithobates catesbeianus* | -98.5356 | 33.87473 |
| *Lithobates catesbeianus* | -99.2066 | 18.97413 |
| *Lithobates catesbeianus* | -76.4664 | 44.43977 |
| *Lithobates catesbeianus* | -73.8143 | 44.87455 |
| *Lithobates catesbeianus* | -76.2913 | 42.55219 |
| *Lithobates catesbeianus* | -80.5284 | 37.7083 |
| *Lithobates catesbeianus* | -70.9811 | 44.0164 |
| *Lithobates catesbeianus* | -122.6 | 37.95603 |
| *Lithobates catesbeianus* | -95.1223 | 34.14252 |
| *Lithobates catesbeianus* | -79.9785 | 32.7139 |
| *Lithobates catesbeianus* | -84.2433 | 30.3929 |
| *Lithobates catesbeianus* | -84.2415 | 30.18118 |
| *Lithobates catesbeianus* | -90.9563 | 30.46021 |
| *Lithobates catesbeianus* | -93.2402 | 30.76173 |
| *Lithobates catesbeianus* | -84.3031 | 30.08736 |
| *Lithobates catesbeianus* | -84.3757 | 30.27182 |
| *Lithobates catesbeianus* | -81.9587 | 29.69698 |
| *Lithobates catesbeianus* | -70.7573 | 43.42437 |
| *Lithobates catesbeianus* | -72.9851 | 41.3188 |
| *Lithobates catesbeianus* | -71.7979 | 41.5982 |
| *Lithobates catesbeianus* | -73.1128 | 41.35733 |
| *Lithobates catesbeianus* | -95.6156 | 29.1411 |
| *Lithobates catesbeianus* | -97.3904 | 35.21107 |
| *Lithobates catesbeianus* | -81.3127 | 41.3089 |
| *Lithobates catesbeianus* | 5.12997 | 51.14473 |
| *Lithobates catesbeianus* | -70.112 | 44.58482 |
| *Lithobates catesbeianus* | -75.7 | 45.26666 |
| *Lithobates catesbeianus* | -82.6347 | 41.82917 |
| *Lithobates catesbeianus* | -79.0833 | 45.88333 |
| *Lithobates catesbeianus* | -78.5714 | 44.99528 |
| *Lithobates catesbeianus* | -76.9219 | 44.7223 |
| *Lithobates catesbeianus* | -79.3734 | 44.92847 |
| *Lithobates catesbeianus* | -80.0833 | 46.95 |
| *Lithobates catesbeianus* | -79.7743 | 46.14091 |
| *Lithobates catesbeianus* | -79.2177 | 43.08381 |
| *Lithobates catesbeianus* | -76.48 | 37.18 |
| *Lithobates catesbeianus* | -68.7012 | 44.75241 |
| *Lithobates catesbeianus* | -76.7962 | 39.02811 |
| *Lithobates catesbeianus* | -76.1755 | 44.71318 |
| *Lithobates catesbeianus* | -123.953 | 41.20389 |
| *Lithobates catesbeianus* | -84.4035 | 40.16022 |
| *Lithobates catesbeianus* | -97.845 | 22.421 |
| *Lithobates catesbeianus* | -116.954 | 33.26308 |
| *Lithobates catesbeianus* | -155.278 | 19.4365 |
| *Lithobates catesbeianus* | -98.959 | 18.524 |
| *Lithobates catesbeianus* | -69.1022 | -31.2837 |
| *Lithobates catesbeianus* | -117.593 | 33.38682 |
| *Lithobates catesbeianus* | -92.5005 | 38.2503 |
| *Lithobates catesbeianus* | -75.783 | 45.25 |
| *Lithobates catesbeianus* | -75.283 | 45.567 |
| *Lithobates catesbeianus* | -76.203 | 45.592 |
| *Lithobates catesbeianus* | -75.866 | 45.512 |
| *Lithobates catesbeianus* | -75.783 | 45.454 |
| *Lithobates catesbeianus* | -75.825 | 45.503 |
| *Lithobates catesbeianus* | -76.071 | 45.599 |
| *Lithobates catesbeianus* | -78.732 | 46.353 |
| *Lithobates catesbeianus* | -67.1 | 45.15 |
| *Lithobates catesbeianus* | -76.184 | 45.854 |
| *Lithobates catesbeianus* | -76.161 | 45.946 |
| *Lithobates catesbeianus* | -81.6 | 45.2 |
| *Lithobates catesbeianus* | -71.417 | 42.383 |
| *Lithobates catesbeianus* | -76.25 | 45.333 |
| *Lithobates catesbeianus* | -75.667 | 45.817 |
| *Lithobates catesbeianus* | -76.22 | 45.485 |
| *Lithobates catesbeianus* | -63.933 | 46.717 |
| *Lithobates catesbeianus* | -75.936 | 45.118 |
| *Lithobates catesbeianus* | -75.367 | 45.517 |
| *Lithobates catesbeianus* | -94.7201 | 34.96006 |
| *Lithobates catesbeianus* | -77.32 | 40.03 |
| *Lithobates catesbeianus* | -79.8397 | 38.97 |
| *Lithobates catesbeianus* | -82.5597 | 38.15002 |
| *Lithobates catesbeianus* | -82.6197 | 38.14002 |
| *Lithobates catesbeianus* | -78.37 | 40.01 |
| *Lithobates catesbeianus* | -79.68 | 41.14 |
| *Lithobates catesbeianus* | -78.9596 | 39.06 |
| *Lithobates catesbeianus* | -82.34 | 29.62 |
| *Lithobates catesbeianus* | -96.4801 | 41.42994 |
| *Lithobates catesbeianus* | -75.15 | 39.6 |
| *Lithobates catesbeianus* | -92.45 | 29.64 |
| *Lithobates catesbeianus* | -106.651 | 31.9109 |
| *Lithobates catesbeianus* | -104.64 | 25.31034 |
| *Lithobates catesbeianus* | -120.567 | 34.67174 |
| *Lithobates catesbeianus* | -118.775 | 34.57209 |
| *Lithobates catesbeianus* | -94.857 | 38.40693 |
| *Lithobates catesbeianus* | -95.302 | 39.01304 |
| *Lithobates catesbeianus* | -101.936 | 38.87523 |
| *Lithobates catesbeianus* | -99.3246 | 37.06333 |
| *Lithobates catesbeianus* | -100.18 | 37.50193 |
| *Lithobates catesbeianus* | -94.9506 | 38.49888 |
| *Lithobates catesbeianus* | -95.2574 | 38.58568 |
| *Lithobates catesbeianus* | -123.171 | 44.51982 |
| *Lithobates catesbeianus* | -0.09147 | 50.98351 |
| *Lithobates catesbeianus* | -87.2763 | 41.6125 |
| *Lithobates catesbeianus* | -87.0882 | 41.642 |
| *Lithobates catesbeianus* | -85.3181 | 42.1003 |
| *Lithobates catesbeianus* | -83.7944 | 45.9936 |
| *Lithobates catesbeianus* | -78.4077 | 43.1436 |
| *Lithobates catesbeianus* | -78.3878 | 42.4606 |
| *Lithobates catesbeianus* | -82.1557 | 41.285 |
| *Lithobates catesbeianus* | -74.3956 | 45.1792 |
| *Lithobates catesbeianus* | -80.4225 | 42.5811 |
| *Lithobates catesbeianus* | -79.5306 | 45.4833 |
| *Lithobates catesbeianus* | -79.8181 | 43.9583 |
| *Lithobates catesbeianus* | -77.7289 | 44.0042 |
| *Lithobates catesbeianus* | -80.2667 | 43.1333 |
| *Lithobates catesbeianus* | -76.6367 | 44.379 |
| *Lithobates catesbeianus* | -79.4855 | 44.192 |
| *Lithobates catesbeianus* | -79.1171 | 43.0111 |
| *Lithobates catesbeianus* | -80.4813 | 42.6048 |
| *Lithobates catesbeianus* | -77.3543 | 44.2848 |
| *Lithobates catesbeianus* | -88.5025 | 42.9824 |
| *Lithobates catesbeianus* | -75.77 | 41.78 |
| *Lithobates catesbeianus* | -79.26 | 40.16 |
| *Lithobates catesbeianus* | -79.11 | 38.01 |
| *Lithobates catesbeianus* | -76.12 | 36.7 |
| *Lithobates catesbeianus* | -76.68 | 37.25 |
| *Lithobates catesbeianus* | -77.59 | 37.69 |
| *Lithobates catesbeianus* | -76.43 | 36.69 |
| *Lithobates catesbeianus* | -76.71 | 36.61 |
| *Lithobates catesbeianus* | -77.33 | 38.12 |
| *Lithobates catesbeianus* | -75.97 | 36.83 |
| *Lithobates catesbeianus* | -97.8764 | 42.6094 |
| *Lithobates catesbeianus* | -83.3644 | 42.8089 |
| *Lithobates catesbeianus* | -82.1992 | 39.7595 |
| *Lithobates catesbeianus* | -84.7706 | 39.53125 |
| *Lithobates catesbeianus* | -82.8564 | 38.69145 |
| *Lithobates catesbeianus* | -84.1351 | 39.56225 |
| *Lithobates catesbeianus* | -94.6279 | 37.1318 |
| *Lithobates catesbeianus* | -101.984 | 39.0436 |
| *Lithobates catesbeianus* | -85.0161 | 30.05542 |
| *Lithobates catesbeianus* | -98.5586 | 38.15837 |
| *Lithobates catesbeianus* | -80.019 | 33.12406 |
| *Lithobates catesbeianus* | -98.4253 | 35.45999 |
| *Lithobates catesbeianus* | -87.7611 | 38.41224 |
| *Lithobates catesbeianus* | -100.49 | 28.70888 |
| *Lithobates catesbeianus* | -91.2067 | 29.69921 |
| *Lithobates catesbeianus* | -88.0377 | 35.62158 |
| *Lithobates catesbeianus* | -106.8 | 32.27 |
| *Lithobates catesbeianus* | -82.4296 | 38.42052 |
| *Lithobates catesbeianus* | -77.13 | 35.03 |
| *Lithobates catesbeianus* | -72.685 | 44.4653 |
| *Lithobates catesbeianus* | -76.8144 | 39.0565 |
| *Lithobates catesbeianus* | -76.8003 | 39.05062 |
| *Lithobates catesbeianus* | -95.9896 | 42.27325 |
| *Lithobates catesbeianus* | -77.42 | 37.64 |
| *Lithobates catesbeianus* | -78.5395 | 35.82474 |
| *Lithobates catesbeianus* | -76.1 | 36.7 |
| *Lithobates catesbeianus* | -77.1503 | 38.239 |
| *Lithobates catesbeianus* | -77.0699 | 36.971 |
| *Lithobates catesbeianus* | -76.41 | 36.84 |
| *Lithobates catesbeianus* | -76.9469 | 37.6292 |
| *Lithobates catesbeianus* | -79.7913 | 47.06401 |
| *Lithobates catesbeianus* | -79.8167 | 46.2 |
| *Lithobates catesbeianus* | -78.05 | 45.96666 |
| *Lithobates catesbeianus* | -78.55 | 44.53333 |
| *Lithobates catesbeianus* | -82.3261 | 29.64509 |
| *Lithobates catesbeianus* | -97.401 | 35.17387 |
| *Lithobates catesbeianus* | -94.6221 | 36.14968 |
| *Lithobates catesbeianus* | -96.0494 | 34.3861 |
| *Lithobates catesbeianus* | -94.7387 | 35.49839 |
| *Lithobates catesbeianus* | -94.5459 | 34.7747 |
| *Lithobates catesbeianus* | -98.1952 | 34.67017 |
| *Lithobates catesbeianus* | -99.3319 | 36.48613 |
| *Lithobates catesbeianus* | -96.3697 | 35.02818 |
| *Lithobates catesbeianus* | -94.7267 | 34.02651 |
| *Lithobates catesbeianus* | -97.2136 | 35.22097 |
| *Lithobates catesbeianus* | -92.5326 | 37.91576 |
| *Lithobates catesbeianus* | -102.813 | 36.91492 |
| *Lithobates catesbeianus* | -99.4761 | 34.94996 |
| *Lithobates catesbeianus* | -98.2865 | 34.20909 |
| *Lithobates catesbeianus* | -97.2804 | 36.80583 |
| *Lithobates catesbeianus* | -78.749 | 35.83 |
| *Lithobates catesbeianus* | -76.774 | 35.0258 |
| *Lithobates catesbeianus* | -76.4509 | 35.4982 |
| *Lithobates catesbeianus* | -77.8802 | 34.5949 |
| *Lithobates catesbeianus* | -78.3576 | 35.9651 |
| *Lithobates catesbeianus* | -75.6687 | 35.9919 |
| *Lithobates catesbeianus* | -78.8575 | 35.8828 |
| *Lithobates catesbeianus* | -75.6525 | 35.9714 |
| *Lithobates catesbeianus* | -78.6008 | 34.2623 |
| *Lithobates catesbeianus* | -78.2501 | 36.145 |
| *Lithobates catesbeianus* | -78.8171 | 35.6211 |
| *Lithobates catesbeianus* | -78.3624 | 34.1626 |
| *Lithobates catesbeianus* | -78.3851 | 34.2845 |
| *Lithobates catesbeianus* | -81.0962 | 36.5084 |
| *Lithobates catesbeianus* | -75.1363 | 38.5692 |
| *Lithobates catesbeianus* | -77.3306 | 34.626 |
| *Lithobates catesbeianus* | -77.4181 | 37.5989 |
| *Lithobates catesbeianus* | -77.625 | 37.6658 |
| *Lithobates catesbeianus* | -77.1136 | 37.4803 |
| *Lithobates catesbeianus* | -76.4205 | 36.9002 |
| *Lithobates catesbeianus* | -81.9626 | 36.1868 |
| *Lithobates catesbeianus* | -79.7674 | 36.9399 |
| *Lithobates catesbeianus* | -80.2106 | 35.745 |
| *Lithobates catesbeianus* | -122.396 | 37.25065 |
| *Lithobates catesbeianus* | -121.193 | 39.51882 |
| *Lithobates catesbeianus* | -121.094 | 38.33852 |
| *Lithobates catesbeianus* | -121.89 | 37.24332 |
| *Lithobates catesbeianus* | -121.916 | 37.30376 |
| *Lithobates catesbeianus* | -121.365 | 39.72308 |
| *Lithobates catesbeianus* | -121.559 | 36.9449 |
| *Lithobates catesbeianus* | -119.307 | 36.92081 |
| *Lithobates catesbeianus* | -120.813 | 35.83033 |
| *Lithobates catesbeianus* | -121.559 | 36.95177 |
| *Lithobates catesbeianus* | -121.85 | 39.36204 |
| *Lithobates catesbeianus* | -72.4011 | 44.5589 |
| *Lithobates catesbeianus* | -118.225 | 34.28518 |
| *Lithobates catesbeianus* | -118.867 | 34.1372 |
| *Lithobates catesbeianus* | -116.309 | 33.83728 |
| *Lithobates catesbeianus* | -118.087 | 34.02334 |
| *Lithobates catesbeianus* | -118.707 | 34.08485 |
| *Lithobates catesbeianus* | -117.187 | 34.2671 |
| *Lithobates catesbeianus* | -106.778 | 32.31222 |
| *Lithobates catesbeianus* | -122.433 | 39.18981 |
| *Lithobates catesbeianus* | -117.959 | 33.92367 |
| *Lithobates catesbeianus* | -118.07 | 34.00501 |
| *Lithobates catesbeianus* | -118.68 | 35.69776 |
| *Lithobates catesbeianus* | -121.636 | 39.52812 |
| *Lithobates catesbeianus* | -121.519 | 39.7719 |
| *Lithobates catesbeianus* | -155.522 | 19.58049 |
| *Lithobates catesbeianus* | -123.709 | 39.57211 |
| *Lithobates catesbeianus* | -121.975 | 37.22661 |
| *Lithobates catesbeianus* | -113.394 | 39.861 |
| *Lithobates catesbeianus* | -109.537 | 31.6006 |
| *Lithobates catesbeianus* | -123.788 | 46.2062 |
| *Lithobates catesbeianus* | -82.5 | 42.1667 |
| *Lithobates catesbeianus* | -114.357 | 34.5681 |
| *Lithobates catesbeianus* | -110.87 | 31.4931 |
| *Lithobates catesbeianus* | -111.666 | 34.375 |
| *Lithobates catesbeianus* | -111.826 | 34.6328 |
| *Lithobates catesbeianus* | -65.6438 | 18.37051 |
| *Lithobates catesbeianus* | -123.077 | 44.7842 |
| *Lithobates catesbeianus* | -122.829 | 45.64099 |
| *Lithobates catesbeianus* | -114.5 | 37.2833 |
| *Lithobates catesbeianus* | -96.6266 | 28.54242 |
| *Lithobates catesbeianus* | -94.7761 | 31.50111 |
| *Lithobates catesbeianus* | -96.2989 | 30.73583 |
| *Lithobates catesbeianus* | -96.5017 | 30.7025 |
| *Lithobates catesbeianus* | -96.3607 | 30.64121 |
| *Lithobates catesbeianus* | -96.2577 | 29.95047 |
| *Lithobates catesbeianus* | -96.0447 | 28.96438 |
| *Lithobates catesbeianus* | -95.0561 | 33.76033 |
| *Lithobates catesbeianus* | -96.1964 | 33.75362 |
| *Lithobates catesbeianus* | -99.7312 | 34.10441 |
| *Lithobates catesbeianus* | -99.0242 | 31.47694 |
| *Lithobates catesbeianus* | -100.906 | 32.69335 |
| *Lithobates catesbeianus* | -116.304 | 43.61947 |
| *Lithobates catesbeianus* | -120.169 | 39.03985 |
| *Lithobates catesbeianus* | -120.055 | 38.93801 |
| *Lithobates catesbeianus* | -111.823 | 33.56735 |
| *Lithobates catesbeianus* | -81.3314 | 28.54586 |
| *Lithobates catesbeianus* | -118.44 | 33.35376 |
| *Lithobates catesbeianus* | -119.167 | 36.36679 |
| *Lithobates catesbeianus* | -121.798 | 39.31562 |
| *Lithobates catesbeianus* | -122.711 | 47.07191 |
| *Lithobates catesbeianus* | -81.4471 | 41.08107 |
| *Lithobates catesbeianus* | -76.6876 | 39.88137 |
| *Lithobates catesbeianus* | -79.2525 | 42.10517 |
| *Lithobates catesbeianus* | -75.9129 | 40.19138 |
| *Lithobates catesbeianus* | -75.452 | 39.25319 |
| *Lithobates catesbeianus* | -79.4607 | 40.90023 |
| *Lithobates catesbeianus* | -116.347 | 36.483 |
| *Lithobates catesbeianus* | -119.717 | 39.55 |
| *Lithobates catesbeianus* | -116 | 36.2166 |
| *Lithobates catesbeianus* | -115.217 | 37.6 |
| *Lithobates catesbeianus* | -79.0547 | 42.94141 |
| *Lithobates catesbeianus* | -77.5101 | 37.54613 |
| *Lithobates catesbeianus* | -90.2904 | 38.77482 |
| *Lithobates catesbeianus* | -97.5454 | 30.26966 |
| *Lithobates catesbeianus* | -72.4181 | 41.30016 |
| *Lithobates catesbeianus* | -116.335 | 36.4252 |
| *Lithobates catesbeianus* | -122.223 | 37.41518 |
| *Lithobates catesbeianus* | -91.1273 | 30.3191 |
| *Lithobates catesbeianus* | -121.292 | 38.74583 |
| *Lithobates catesbeianus* | -120.502 | 36.80348 |
| *Lithobates catesbeianus* | -122.646 | 38.44722 |
| *Lithobates catesbeianus* | -118.351 | 34.34996 |
| *Lithobates catesbeianus* | -120.185 | 37.29218 |
| *Lithobates catesbeianus* | -115.987 | 36.20521 |
| *Lithobates catesbeianus* | -86.2209 | 41.70376 |
| *Lithobates catesbeianus* | -117.291 | 34.59412 |
| *Lithobates catesbeianus* | -97.677 | 30.633 |
| *Lithobates catesbeianus* | -121.742 | 36.96245 |
| *Lithobates catesbeianus* | -84.7987 | 30.7745 |
| *Lithobates catesbeianus* | -105.228 | 40.1565 |
| *Lithobates catesbeianus* | -122.795 | 44.25162 |
| *Lithobates catesbeianus* | -122.9 | 46.99 |
| *Lithobates catesbeianus* | -108.183 | 42.83197 |
| *Lithobates catesbeianus* | -71.4172 | 43.40139 |
| *Lithobates catesbeianus* | -82.3014 | 35.91667 |
| *Lithobates catesbeianus* | -117.422 | 47.63814 |
| *Lithobates catesbeianus* | -96.8077 | 32.95455 |
| *Lithobates catesbeianus* | -122.817 | 38.08552 |
| *Lithobates catesbeianus* | -118.409 | 34.12002 |
| *Lithobates catesbeianus* | -117.936 | 33.9093 |
| *Lithobates catesbeianus* | -96.1998 | 30.81151 |
| *Lithobates catesbeianus* | -72.7609 | 41.56938 |
| *Lithobates catesbeianus* | -81.5282 | 41.01617 |
| *Lithobates catesbeianus* | -95.7757 | 30.56258 |
| *Lithobates catesbeianus* | -80.5534 | 45.94285 |
| *Lithobates catesbeianus* | -110.391 | 31.27215 |
| *Lithobates catesbeianus* | 126.7078 | 35.53313 |
| *Lithobates catesbeianus* | -118.167 | 34.2005 |
| *Lithobates catesbeianus* | -74.4535 | 40.57744 |
| *Lithobates catesbeianus* | -95.8852 | 31.93524 |
| *Lithobates catesbeianus* | -118.859 | 36.44702 |
| *Lithobates catesbeianus* | -96.2391 | 30.68617 |
| *Lithobates catesbeianus* | -72.5068 | 42.36972 |
| *Lithobates catesbeianus* | -76.5488 | 44.26831 |
| *Lithobates catesbeianus* | -122.135 | 48.0607 |
| *Lithobates catesbeianus* | -122.654 | 38.44978 |
| *Lithobates catesbeianus* | -96.9551 | 33.06684 |
| *Lithobates catesbeianus* | -121.364 | 38.05705 |
| *Lithobates catesbeianus* | -76.7464 | 39.22628 |
| *Lithobates catesbeianus* | -82.8302 | 42.17399 |
| *Lithobates catesbeianus* | -96.1022 | 30.64783 |
| *Lithobates catesbeianus* | -73.9908 | 40.65294 |
| *Lithobates catesbeianus* | -75.4848 | 45.49945 |
| *Lithobates catesbeianus* | -76.7028 | 38.78464 |
| *Lithobates catesbeianus* | -97.1288 | 33.22847 |
| *Lithobates catesbeianus* | -96.8908 | 33.18918 |
| *Lithobates catesbeianus* | -88.9031 | 43.09039 |
| *Lithobates catesbeianus* | -87.5534 | 41.64258 |
| *Lithobates catesbeianus* | -71.4455 | 41.23422 |
| *Lithobates catesbeianus* | -118.361 | 33.35287 |
| *Lithobates catesbeianus* | -77.2304 | 38.63898 |
| *Lithobates catesbeianus* | -94.3799 | 29.83648 |
| *Lithobates catesbeianus* | -121.296 | 38.14699 |
| *Lithobates catesbeianus* | -97.6496 | 33.66086 |
| *Lithobates catesbeianus* | -76.2749 | 36.81904 |
| *Lithobates catesbeianus* | -121.841 | 47.54466 |
| *Lithobates catesbeianus* | -98.0012 | 32.80013 |
| *Lithobates catesbeianus* | -76.0239 | 45.4378 |
| *Lithobates catesbeianus* | -95.8784 | 30.10522 |
| *Lithobates catesbeianus* | -95.6065 | 29.37433 |
| *Lithobates catesbeianus* | 126.453 | 36.70256 |
| *Lithobates catesbeianus* | -120.637 | 35.48331 |
| *Lithobates catesbeianus* | -80.5258 | 42.67687 |
| *Lithobates catesbeianus* | -96.2536 | 41.02298 |
| *Lithobates catesbeianus* | -95.9877 | 30.09327 |
| *Lithobates catesbeianus* | -121.291 | 37.9577 |
| *Lithobates catesbeianus* | -95.447 | 32.57258 |
| *Lithobates catesbeianus* | -77.4054 | 39.01743 |
| *Lithobates catesbeianus* | -84.6056 | 33.39348 |
| *Lithobates catesbeianus* | -97.5831 | 29.5881 |
| *Lithobates catesbeianus* | -83.2003 | 39.99452 |
| *Lithobates catesbeianus* | -76.1982 | 42.36249 |
| *Lithobates catesbeianus* | -76.4754 | 42.44419 |
| *Lithobates catesbeianus* | -73.7738 | 43.49541 |
| *Lithobates catesbeianus* | -82.0393 | 28.30101 |
| *Lithobates catesbeianus* | -77.7581 | 43.30045 |
| *Lithobates catesbeianus* | -78.8059 | 38.9533 |
| *Lithobates catesbeianus* | -79.6862 | 42.08154 |
| *Lithobates catesbeianus* | -79.5496 | 35.74358 |
| *Lithobates catesbeianus* | -77.5866 | 37.0779 |
| *Lithobates catesbeianus* | -96.8594 | 38.9014 |
| *Lithobates catesbeianus* | -94.739 | 38.01967 |
| *Lithobates catesbeianus* | -99.3655 | 37.19305 |
| *Lithobates catesbeianus* | -95.3585 | 39.93551 |
| *Lithobates catesbeianus* | -95.086 | 34.91804 |
| *Lithobates catesbeianus* | -117.001 | 33.69292 |
| *Lithobates catesbeianus* | -121.474 | 38.7116 |
| *Lithobates catesbeianus* | -120.419 | 39.8168 |
| *Lithobates catesbeianus* | -122.653 | 38.40485 |
| *Lithobates catesbeianus* | -119.175 | 36.79817 |
| *Lithobates catesbeianus* | -118.762 | 36.19908 |
| *Lithobates catesbeianus* | -122.071 | 40.73879 |
| *Lithobates catesbeianus* | -116.707 | 33.0031 |
| *Lithobates catesbeianus* | -121.74 | 37.09373 |
| *Lithobates catesbeianus* | -116.223 | 43.6135 |
| *Lithobates catesbeianus* | -97.1464 | 31.54917 |
| *Lithobates catesbeianus* | -96.4476 | 30.54001 |
| *Lithobates catesbeianus* | -120.057 | 38.94056 |
| *Lithobates catesbeianus* | -121.32 | 37.43811 |
| *Lithobates catesbeianus* | -119.531 | 36.49698 |
| *Lithobates catesbeianus* | -100.519 | 36.82231 |
| *Lithobates catesbeianus* | -99.225 | 18.564 |
| *Lithobates catesbeianus* | -106.64 | 32.31215 |
| *Lithobates catesbeianus* | -119.289 | 35.1918 |
| *Lithobates catesbeianus* | -84.3325 | 33.8746 |
| *Lithobates catesbeianus* | -83.1904 | 35.054 |
| *Lithobates catesbeianus* | -118.463 | 36.07819 |
| *Lithobates catesbeianus* | -66.0592 | 18.38477 |
| *Lithobates catesbeianus* | -114.75 | 33.4315 |
| *Lithobates catesbeianus* | -117.626 | 33.71238 |
| *Lithobates catesbeianus* | -114.575 | 34.80531 |
| *Lithobates catesbeianus* | -118.197 | 33.85929 |
| *Lithobates catesbeianus* | -118.193 | 33.86533 |
| *Lithobates catesbeianus* | -106.778 | 32.31221 |
| *Lithobates catesbeianus* | -121.754 | 37.16327 |
| *Lithobates catesbeianus* | -121.737 | 37.13024 |
| *Lithobates catesbeianus* | -117.598 | 33.92333 |
| *Lithobates catesbeianus* | -117.578 | 33.38482 |
| *Lithobates catesbeianus* | -117.977 | 36.53344 |
| *Lithobates catesbeianus* | -95.2003 | 31.39589 |
| *Lithobates catesbeianus* | -96.4356 | 30.62778 |
| *Lithobates catesbeianus* | -96.2275 | 30.53556 |
| *Lithobates catesbeianus* | -98.4236 | 29.42361 |
| *Lithobates catesbeianus* | -97.8235 | 32.91878 |
| *Lithobates catesbeianus* | -97.4255 | 31.16392 |
| *Lithobates catesbeianus* | -95.908 | 29.78614 |
| *Lithobates catesbeianus* | -95.3973 | 32.74301 |
| *Lithobates catesbeianus* | -97.4289 | 30.05319 |
| *Lithobates catesbeianus* | -100.127 | 32.73994 |
| *Lithobates catesbeianus* | -98.5507 | 33.84792 |
| *Lithobates catesbeianus* | -98.6133 | 33.60571 |
| *Lithobates catesbeianus* | -94.735 | 33.23881 |
| *Lithobates catesbeianus* | -99.575 | 29.84225 |
| *Lithobates catesbeianus* | -93.7139 | 30.11861 |
| *Lithobates catesbeianus* | -95.2423 | 39.05609 |
| *Lithobates catesbeianus* | -94.7215 | 37.41695 |
| *Lithobates catesbeianus* | -83.7421 | 42.26955 |
| *Lithobates catesbeianus* | -101.8 | 39.79272 |
| *Lithobates catesbeianus* | -96.6474 | 39.76246 |
| *Lithobates catesbeianus* | -108.97 | 25.8303 |
| *Lithobates catesbeianus* | -88.716 | 36.43468 |
| *Lithobates catesbeianus* | -83.0482 | 40.43526 |
| *Lithobates catesbeianus* | -100.452 | 37.17277 |
| *Lithobates catesbeianus* | -95.2412 | 38.18166 |
| *Lithobates catesbeianus* | -99.9977 | 37.2386 |
| *Lithobates catesbeianus* | -98.6366 | 38.47803 |
| *Lithobates catesbeianus* | -91.9632 | 40.97609 |
| *Lithobates catesbeianus* | -83.9753 | 39.58056 |
| *Lithobates catesbeianus* | -84.3804 | 40.15185 |
| *Lithobates catesbeianus* | -84.7791 | 39.1933 |
| *Lithobates catesbeianus* | -83.9723 | 39.44512 |
| *Lithobates catesbeianus* | -82.7974 | 39.10429 |
| *Lithobates catesbeianus* | -107.184 | 33.14861 |
| *Lithobates catesbeianus* | -105.688 | 31.12991 |
| *Lithobates catesbeianus* | -120.558 | 34.67604 |
| *Lithobates catesbeianus* | -120.54 | 34.68067 |
| *Lithobates catesbeianus* | -98.6987 | 35.53215 |
| *Lithobates catesbeianus* | -98.9181 | 36.57924 |
| *Lithobates catesbeianus* | -95.3072 | 34.919 |
| *Lithobates catesbeianus* | -98.9799 | 34.52328 |
| *Lithobates catesbeianus* | -94.712 | 36.45248 |
| *Lithobates catesbeianus* | -97.9622 | 34.11319 |
| *Lithobates catesbeianus* | -97.897 | 22.38647 |
| *Lithobates catesbeianus* | -82.0918 | 27.7223 |
| *Lithobates catesbeianus* | -94.6314 | 34.5774 |
| *Lithobates catesbeianus* | -98.4621 | 35.83385 |
| *Lithobates catesbeianus* | -97.8558 | 35.00756 |
| *Lithobates catesbeianus* | -94.7125 | 36.54568 |
| *Lithobates catesbeianus* | -97.5909 | 34.81927 |
| *Lithobates catesbeianus* | -78.2342 | 34.9837 |
| *Lithobates catesbeianus* | -82.2045 | 35.4283 |
| *Lithobates catesbeianus* | -81.6481 | 36.1533 |
| *Lithobates catesbeianus* | -81.6482 | 36.1533 |
| *Lithobates catesbeianus* | -80.2543 | 36.4093 |
| *Lithobates catesbeianus* | -82.1565 | 35.2825 |
| *Lithobates catesbeianus* | -77.9855 | 34.0059 |
| *Lithobates catesbeianus* | -78.3889 | 34.2052 |
| *Lithobates catesbeianus* | -81.1614 | 35.913 |
| *Lithobates catesbeianus* | -82.9275 | 34.9138 |
| *Lithobates catesbeianus* | -78.7843 | 35.7265 |
| *Lithobates catesbeianus* | -78.3646 | 34.165 |
| *Lithobates catesbeianus* | -76.158 | 36.1316 |
| *Lithobates catesbeianus* | -82.4878 | 35.1517 |
| *Lithobates catesbeianus* | -75.2219 | 38.7985 |
| *Lithobates catesbeianus* | -81.3008 | 34.9697 |
| *Lithobates catesbeianus* | -82.6122 | 35.7945 |
| *Lithobates catesbeianus* | -77.1883 | 37.5081 |
| *Lithobates catesbeianus* | -82.4569 | 35.8397 |
| *Lithobates catesbeianus* | -78.5412 | 36.6578 |
| *Lithobates catesbeianus* | -77.426 | 37.6194 |
| *Lithobates catesbeianus* | -79.835 | 36.1345 |
| *Lithobates catesbeianus* | -122.677 | 38.45031 |
| *Lithobates catesbeianus* | -122.659 | 38.4543 |
| *Lithobates catesbeianus* | -72.9018 | 41.65034 |
| *Lithobates catesbeianus* | -86.4732 | 36.95267 |
| *Lithobates catesbeianus* | -122.785 | 38.61307 |
| *Lithobates catesbeianus* | -84.1961 | 39.49817 |
| *Lithobates catesbeianus* | -83.9044 | 39.91194 |
| *Lithobates catesbeianus* | -75.867 | 45.35 |
| *Lithobates catesbeianus* | -73.027 | 46.713 |
| *Lithobates catesbeianus* | -80.367 | 45.55 |
| *Lithobates catesbeianus* | -79.662 | 43.548 |
| *Lithobates catesbeianus* | -76.517 | 46.55 |
| *Lithobates catesbeianus* | -75.667 | 45.45 |
| *Lithobates catesbeianus* | 0 | 45.767 |
| *Lithobates catesbeianus* | -74.523 | 45.747 |
| *Lithobates catesbeianus* | -75.117 | 45.333 |
| *Lithobates catesbeianus* | -75.668 | 44.992 |
| *Lithobates catesbeianus* | -82 | 42.4 |
| *Lithobates catesbeianus* | -75.874 | 45.501 |
| *Lithobates catesbeianus* | -76.452 | 44.611 |
| *Lithobates catesbeianus* | -75.617 | 45.45 |
| *Lithobates catesbeianus* | -76.178 | 45.621 |
| *Lithobates catesbeianus* | -76.001 | 45.636 |
| *Lithobates catesbeianus* | -76.096 | 45.599 |
| *Lithobates catesbeianus* | -75.873 | 45.957 |
| *Lithobates catesbeianus* | -75.653 | 45.596 |
| *Lithobates catesbeianus* | -65.869 | 43.923 |
| *Lithobates catesbeianus* | -75.678 | 44.965 |
| *Lithobates catesbeianus* | -75.687 | 44.86 |
| *Lithobates catesbeianus* | -76.95 | 45.217 |
| *Lithobates catesbeianus* | -72.383 | 45.2 |
| *Lithobates catesbeianus* | -75.827 | 46.192 |
| *Lithobates catesbeianus* | -76.903 | 44.741 |
| *Lithobates catesbeianus* | -79.385 | 46.303 |
| *Lithobates catesbeianus* | -82.15 | 46.867 |
| *Lithobates catesbeianus* | -76.333 | 44.95 |
| *Lithobates catesbeianus* | -81.867 | 46.133 |
| *Lithobates catesbeianus* | -73.95 | 45.417 |
| *Lithobates catesbeianus* | -82.27 | 29.65 |
| *Lithobates catesbeianus* | -81.4 | 31.79 |
| *Lithobates catesbeianus* | -86.4 | 34.21 |
| *Lithobates catesbeianus* | -78.06 | 40.89 |
| *Lithobates catesbeianus* | -77.9 | 39.91 |
| *Lithobates catesbeianus* | -76.69 | 37.58 |
| *Lithobates catesbeianus* | -76.69 | 37.27 |
| *Lithobates catesbeianus* | -75.31 | 37.94 |
| *Lithobates catesbeianus* | -81.8 | 36.96 |
| *Lithobates catesbeianus* | -76.49 | 37.17 |
| *Lithobates catesbeianus* | -78.99 | 37.97 |
| *Lithobates catesbeianus* | -76.39 | 37.04 |
| *Lithobates catesbeianus* | -76.78 | 37.22 |
| *Lithobates catesbeianus* | -76.74 | 37.23 |
| *Lithobates catesbeianus* | -119.809 | 34.425 |
| *Lithobates catesbeianus* | -87.8024 | 42.4517 |
| *Lithobates catesbeianus* | -85.3105 | 42.6166 |
| *Lithobates catesbeianus* | -86.0209 | 41.8261 |
| *Lithobates catesbeianus* | -77.6799 | 43.283 |
| *Lithobates catesbeianus* | -78.7198 | 43.3327 |
| *Lithobates catesbeianus* | -78.995 | 43.0595 |
| *Lithobates catesbeianus* | -76.2247 | 43.8343 |
| *Lithobates catesbeianus* | -83.1846 | 41.6209 |
| *Lithobates catesbeianus* | -81.3199 | 41.1773 |
| *Lithobates catesbeianus* | -81.5513 | 41.2433 |
| *Lithobates catesbeianus* | -81.4652 | 43.6302 |
| *Lithobates catesbeianus* | -77.3665 | 44.1591 |
| *Lithobates catesbeianus* | -74.6818 | 45.2667 |
| *Lithobates catesbeianus* | -82.3968 | 42.3796 |
| *Lithobates catesbeianus* | -78.4141 | 44.5154 |
| *Lithobates catesbeianus* | -80.3364 | 42.6656 |
| *Lithobates catesbeianus* | -79.9333 | 44.8 |
| *Lithobates catesbeianus* | -76.2917 | 44.4389 |
| *Lithobates catesbeianus* | -79.3041 | 43.1021 |
| *Lithobates catesbeianus* | -78.6707 | 43.8912 |
| *Lithobates catesbeianus* | -88.4067 | 43.0456 |
| *Lithobates catesbeianus* | -91.5931 | 46.6231 |
| *Lithobates catesbeianus* | -157.728 | 21.3327 |
| *Lithobates catesbeianus* | -122.773 | 47.0182 |
| *Lithobates catesbeianus* | -122.662 | 45.6387 |
| *Lithobates catesbeianus* | -122.964 | 46.662 |
| *Lithobates catesbeianus* | -120.506 | 46.4863 |
| *Lithobates catesbeianus* | -79.9556 | 32.7277 |
| *Lithobates catesbeianus* | -84.6486 | 39.6289 |
| *Lithobates catesbeianus* | -83.0304 | 38.9932 |
| *Lithobates catesbeianus* | -84.2311 | 39.1089 |
| *Lithobates catesbeianus* | -92.0004 | 31.0005 |
| *Lithobates catesbeianus* | -78.53 | 44.64 |
| *Lithobates catesbeianus* | -78.26 | 44.68 |
| *Lithobates catesbeianus* | -82.1004 | 29.51164 |
| *Lithobates catesbeianus* | -81.9042 | 29.37225 |
| *Lithobates catesbeianus* | -85.1194 | 30.3172 |
| *Lithobates catesbeianus* | -81.7999 | 27.5067 |
| *Lithobates catesbeianus* | -85.9594 | 30.97991 |
| *Lithobates catesbeianus* | -83.3768 | 30.12747 |
| *Lithobates catesbeianus* | -84.2566 | 30.44996 |
| *Lithobates catesbeianus* | -87.3441 | 30.93531 |
| *Lithobates catesbeianus* | -85.2193 | 30.8712 |
| *Lithobates catesbeianus* | -82.2044 | 29.5287 |
| *Lithobates catesbeianus* | -81.6854 | 30.2342 |
| *Lithobates catesbeianus* | -73.7167 | 4.6 |
| *Lithobates catesbeianus* | -73.1281 | 6.12111 |
| *Lithobates catesbeianus* | -75.7738 | 5.01942 |
| *Lithobates catesbeianus* | -97.3908 | 35.21148 |
| *Lithobates catesbeianus* | 5.13022 | 51.14521 |
| *Lithobates catesbeianus* | 5.13016 | 51.14513 |
| *Lithobates catesbeianus* | -95.4439 | 35.01926 |
| *Lithobates catesbeianus* | 2.0423 | 41.365 |
| *Lithobates catesbeianus* | -121.821 | 37.50364 |
| *Lithobates catesbeianus* | -82.1733 | 43.04 |
| *Lithobates catesbeianus* | -78.8666 | 45.83333 |
| *Lithobates catesbeianus* | -78.7936 | 45.63972 |
| *Lithobates catesbeianus* | -76.8483 | 44.73887 |
| *Lithobates catesbeianus* | -78.5167 | 45.05 |
| *Lithobates catesbeianus* | -80.5 | 45.56666 |
| *Lithobates catesbeianus* | -82.5 | 39.9167 |
| *Lithobates catesbeianus* | -110.167 | 31.7333 |
| *Lithobates catesbeianus* | -76.3239 | 3.46966 |
| *Lithobates catesbeianus* | -76.3918 | 3.7882 |
| *Lithobates catesbeianus* | -95.6 | 30.08333 |
| *Lithobates catesbeianus* | -102.011 | 37.52421 |
| *Lithobates catesbeianus* | 138.628 | 36.2103 |
| *Lithobates catesbeianus* | 138.885 | 36.2792 |
| *Lithobates catesbeianus* | -74.1317 | 44.32979 |
| *Lithobates catesbeianus* | -88.0728 | 38.75828 |
| *Lithobates catesbeianus* | -83.0642 | 36.69511 |
| *Lithobates catesbeianus* | -69.9775 | 44.305 |
| *Lithobates catesbeianus* | -78.48 | 34.68 |
| *Lithobates catesbeianus* | -73.9664 | 41.36823 |
| *Lithobates catesbeianus* | -79.62 | 37.99 |
| *Lithobates catesbeianus* | -91.1398 | 43.69071 |
| *Lithobates catesbeianus* | -76.5347 | 38.6862 |
| *Lithobates catesbeianus* | -78.3053 | 39.51448 |
| *Lithobates catesbeianus* | -77.53 | 37.41 |
| *Lithobates catesbeianus* | -65.1607 | 44.40679 |
| *Lithobates catesbeianus* | -77.22 | 37.62 |
| *Lithobates catesbeianus* | -77.49 | 37.29 |
| *Lithobates catesbeianus* | -77.56 | 37.8 |
| *Lithobates catesbeianus* | -78.18 | 37.67 |
| *Lithobates catesbeianus* | -76.32 | 36.6 |
| *Lithobates catesbeianus* | -74.1844 | 41.28205 |
| *Lithobates catesbeianus* | -119.983 | 39.516 |
| *Lithobates catesbeianus* | -119.9 | 41.7166 |
| *Lithobates catesbeianus* | -114.683 | 35.1833 |
| *Lithobates catesbeianus* | -122.238 | 37.40426 |
| *Lithobates catesbeianus* | -121.71 | 39.41354 |
| *Lithobates catesbeianus* | -123.259 | 44.9678 |
| *Lithobates catesbeianus* | -123.41 | 46.2308 |
| *Lithobates catesbeianus* | -66.9184 | 18.38059 |
| *Lithobates catesbeianus* | -105.01 | 39.6087 |
| *Lithobates catesbeianus* | -80.4583 | 42.85 |
| *Lithobates catesbeianus* | -79.55 | 45.6333 |
| *Lithobates catesbeianus* | -79.7747 | 46.14098 |
| *Lithobates catesbeianus* | -79.3 | 45.68333 |
| *Lithobates catesbeianus* | -122.782 | 38.61222 |
| *Lithobates catesbeianus* | -115.527 | 33.20856 |
| *Lithobates catesbeianus* | -94.9795 | 36.8984 |
| *Lithobates catesbeianus* | -95.3754 | 39.84385 |
| *Lithobates catesbeianus* | -121.462 | 39.69803 |
| *Lithobates catesbeianus* | -78.4601 | 34.5919 |
| *Lithobates catesbeianus* | -115.709 | 33.07919 |
| *Lithobates catesbeianus* | -86.6454 | 30.74448 |
| *Lithobates catesbeianus* | -119.426 | 37.07886 |
| *Lithobates catesbeianus* | -116.286 | 35.04356 |
| *Lithobates catesbeianus* | -122.019 | 37.92806 |
| *Lithobates catesbeianus* | -123.035 | 40.69963 |
| *Lithobates catesbeianus* | -123.722 | 39.85883 |
| *Lithobates catesbeianus* | -96.3364 | 30.59256 |
| *Lithobates catesbeianus* | -90.1177 | 29.78354 |
| *Lithobates catesbeianus* | -84.6876 | 39.30033 |
| *Lithobates catesbeianus* | -76.725 | 42.46262 |
| *Lithobates catesbeianus* | -97.2807 | 32.88363 |
| *Lithobates catesbeianus* | -75.9395 | 38.95544 |
| *Lithobates catesbeianus* | -122.183 | 37.00397 |
| *Lithobates catesbeianus* | -118.41 | 34.12015 |
| *Lithobates catesbeianus* | -76.2287 | 38.94729 |
| *Lithobates catesbeianus* | -75.9338 | 38.95336 |
| *Lithobates catesbeianus* | -70.7315 | 42.1491 |
| *Lithobates catesbeianus* | -71.7145 | 42.1291 |
| *Lithobates catesbeianus* | -71.233 | 42.603 |
| *Lithobates catesbeianus* | -72.9361 | 42.3972 |
| *Lithobates catesbeianus* | -72.3292 | 42.1583 |
| *Lithobates catesbeianus* | -87.217 | 30.42 |
| *Lithobates catesbeianus* | -103.097 | 41.66717 |
| *Lithobates catesbeianus* | -106.685 | 35.12792 |
| *Lithobates catesbeianus* | -91.427 | 39.98742 |
| *Lithobates catesbeianus* | -88.069 | 41.63893 |
| *Lithobates catesbeianus* | -117.036 | 33.35273 |
| *Lithobates catesbeianus* | -102.121 | 31.96424 |
| *Lithobates catesbeianus* | -68.2075 | 44.36483 |
| *Lithobates catesbeianus* | -75.4985 | 45.50127 |
| *Lithobates catesbeianus* | -111.046 | 31.57181 |
| *Lithobates catesbeianus* | -81.4583 | 41.08143 |
| *Lithobates catesbeianus* | -97.0096 | 33.03087 |
| *Lithobates catesbeianus* | -75.4986 | 45.50113 |
| *Lithobates catesbeianus* | -75.5017 | 45.4991 |
| *Lithobates catesbeianus* | -73.1776 | 44.01925 |
| *Lithobates catesbeianus* | 126.2307 | 36.85858 |
| *Lithobates catesbeianus* | -96.7923 | 28.2501 |
| *Lithobates catesbeianus* | 127.2232 | 36.12606 |
| *Lithobates catesbeianus* | -95.9628 | 31.89859 |
| *Lithobates catesbeianus* | -82.5567 | 36.97804 |
| *Lithobates catesbeianus* | -123.439 | 48.53127 |
| *Lithobates catesbeianus* | -96.8061 | 28.30153 |
| *Lithobates catesbeianus* | -64.982 | 45.58241 |
| *Lithobates catesbeianus* | -121.411 | 38.10813 |
| *Lithobates catesbeianus* | -75.4846 | 45.49915 |
| *Lithobates catesbeianus* | -74.2349 | 41.12174 |
| *Lithobates catesbeianus* | -76.4824 | 44.36252 |
| *Lithobates catesbeianus* | -122.162 | 37.92714 |
| *Lithobates catesbeianus* | -76.9416 | 38.91268 |
| *Lithobates catesbeianus* | -72.575 | 44.28454 |
| *Lithobates catesbeianus* | -92.6804 | 42.78682 |
| *Lithobates catesbeianus* | -94.7279 | 31.37517 |
| *Lithobates catesbeianus* | -118.728 | 34.09774 |
| *Lithobates catesbeianus* | -74.4892 | 40.7125 |
| *Lithobates catesbeianus* | -122.4 | 48.78971 |
| *Lithobates catesbeianus* | -118.729 | 34.09678 |
| *Lithobates catesbeianus* | -116.953 | 32.75196 |
| *Lithobates catesbeianus* | -73.965 | 40.66549 |
| *Lithobates catesbeianus* | -122.713 | 47.07447 |
| *Lithobates catesbeianus* | -82.83 | 42.17341 |
| *Lithobates catesbeianus* | -97.6257 | 30.18455 |
| *Lithobates catesbeianus* | -76.5056 | 42.44599 |
| *Lithobates catesbeianus* | -73.9142 | 42.36139 |
| *Lithobates catesbeianus* | -74.7277 | 44.54198 |
| *Lithobates catesbeianus* | -76.4743 | 42.45101 |
| *Lithobates catesbeianus* | -73.9208 | 44.66507 |
| *Lithobates catesbeianus* | -72.9173 | 41.45292 |
| *Lithobates catesbeianus* | -75.7061 | 36.0644 |
| *Lithobates catesbeianus* | -85.9072 | 35.0286 |
| *Lithobates catesbeianus* | 117 | 23.4667 |
| *Lithobates catesbeianus* | -73.8697 | 41.4416 |
| *Lithobates catesbeianus* | -116.662 | 32.88907 |
| *Lithobates catesbeianus* | -79.0655 | 41.44887 |
| *Lithobates catesbeianus* | -81.0873 | 35.3441 |
| *Lithobates catesbeianus* | -77.1316 | 37.5011 |
| *Lithobates catesbeianus* | -80.35 | 42.725 |
| *Lithobates catesbeianus* | -80.6 | 47.1 |
| *Lithobates catesbeianus* | -77.35 | 45.2 |
| *Lithobates catesbeianus* | -78.4223 | 45.18518 |
| *Lithobates catesbeianus* | -63.5864 | 44.90727 |
| *Lithobates catesbeianus* | -67.1166 | 45.515 |
| *Lithobates catesbeianus* | -78.7 | 45.78333 |
| *Lithobates catesbeianus* | -78.3833 | 45.7 |
| *Lithobates catesbeianus* | -78.1364 | 39.432 |
| *Lithobates catesbeianus* | -120.447 | 39.69566 |
| *Lithobates catesbeianus* | -81.8151 | 32.46191 |
| *Lithobates catesbeianus* | -97.4718 | 35.51694 |
| *Lithobates catesbeianus* | -82.8333 | 37.6667 |
| *Lithobates catesbeianus* | -76.2976 | 3.97735 |
| *Lithobates catesbeianus* | 5.12698 | 51.14296 |
| *Lithobates catesbeianus* | 5.12893 | 51.14414 |
| *Lithobates catesbeianus* | 5.12982 | 51.14378 |
| *Lithobates catesbeianus* | -76.47 | 37.18 |
| *Lithobates catesbeianus* | 139.559 | 36.2456 |
| *Lithobates catesbeianus* | -82.1311 | 35.7366 |
| *Lithobates catesbeianus* | -80.3533 | 33.2222 |
| *Lithobates catesbeianus* | -97.7619 | 30.46087 |
| *Lithobates catesbeianus* | -96.2111 | 30.71278 |
| *Lithobates catesbeianus* | -6.62 | 40.18 |
| *Lithobates catesbeianus* | -88.313 | 47.1531 |
| *Lithobates catesbeianus* | -78.8416 | 43.3117 |
| *Lithobates catesbeianus* | -78.837 | 43.3113 |
| *Lithobates catesbeianus* | -76.7691 | 42.9797 |
| *Lithobates catesbeianus* | -80.2252 | 43.5459 |
| *Lithobates catesbeianus* | -79.5318 | 44.1733 |
| *Lithobates catesbeianus* | -75.9333 | 44.8333 |
| *Lithobates catesbeianus* | -78.8739 | 46.2909 |
| *Lithobates catesbeianus* | -78.1911 | 44.6058 |
| *Lithobates catesbeianus* | -75.7778 | 45.0892 |
| *Lithobates catesbeianus* | -77.3477 | 44.1647 |
| *Lithobates catesbeianus* | -82.653 | 46.3979 |
| *Lithobates catesbeianus* | -77.336 | 44.0863 |
| *Lithobates catesbeianus* | -77.181 | 43.9249 |
| *Lithobates catesbeianus* | -78.9562 | 43.8496 |
| *Lithobates catesbeianus* | -99.184 | 18.839 |
| *Lithobates catesbeianus* | -121.811 | 39.18628 |
| *Lithobates catesbeianus* | -84.6502 | 39.69067 |
| *Lithobates catesbeianus* | -84.4118 | 40.62633 |
| *Lithobates catesbeianus* | -83.8499 | 39.29007 |
| *Lithobates catesbeianus* | -83.4963 | 40.17347 |
| *Lithobates catesbeianus* | -81.7407 | 40.01942 |
| *Lithobates catesbeianus* | -82.861 | 38.89683 |
| *Lithobates catesbeianus* | -83.8676 | 38.91078 |
| *Lithobates catesbeianus* | -83.6488 | 38.68065 |
| *Lithobates catesbeianus* | -82.914 | 39.37845 |
| *Lithobates catesbeianus* | -84.7006 | 39.30517 |
| *Lithobates catesbeianus* | -83.453 | 39.28963 |
| *Lithobates catesbeianus* | -83.8505 | 39.93461 |
| *Lithobates catesbeianus* | -84.2821 | 41.18007 |
| *Lithobates catesbeianus* | -99.3261 | 38.7539 |
| *Lithobates catesbeianus* | -99.7819 | 37.3825 |
| *Lithobates catesbeianus* | -95.6863 | 37.1617 |
| *Lithobates catesbeianus* | -98.1217 | 38.5073 |
| *Lithobates catesbeianus* | -98.7438 | 37.2911 |
| *Lithobates catesbeianus* | -95.1979 | 37.4194 |
| *Lithobates catesbeianus* | -99.1706 | 37.1209 |
| *Lithobates catesbeianus* | -95.2534 | 39.94196 |
| *Lithobates catesbeianus* | -97.083 | 37.07761 |
| *Lithobates catesbeianus* | -94.8232 | 37.83196 |
| *Lithobates catesbeianus* | -122.784 | 46.2957 |
| *Lithobates catesbeianus* | -121.926 | 45.6957 |
| *Lithobates catesbeianus* | -122.284 | 40.37755 |
| *Lithobates catesbeianus* | -121.336 | 37.43128 |
| *Lithobates catesbeianus* | -121.371 | 37.42055 |
| *Lithobates catesbeianus* | -121.244 | 38.48287 |
| *Lithobates catesbeianus* | -123.234 | 39.18105 |
| *Lithobates catesbeianus* | -120.787 | 35.54847 |
| *Lithobates catesbeianus* | -121.784 | 37.70056 |
| *Lithobates catesbeianus* | -122.39 | 38.88386 |
| *Lithobates catesbeianus* | -120.99 | 39.65976 |
| *Lithobates catesbeianus* | -120.947 | 39.93672 |
| *Lithobates catesbeianus* | -123.024 | 43.78983 |
| *Lithobates catesbeianus* | -121.857 | 40.98789 |
| *Lithobates catesbeianus* | -117.232 | 32.90925 |
| *Lithobates catesbeianus* | -122.6 | 38.40854 |
| *Lithobates catesbeianus* | -86.5647 | 39.16389 |
| *Lithobates catesbeianus* | -119.816 | 39.05813 |
| *Lithobates catesbeianus* | -123.184 | 38.62187 |
| *Lithobates catesbeianus* | -120.983 | 39.60339 |
| *Lithobates catesbeianus* | -99.8569 | 32.42 |
| *Lithobates catesbeianus* | -96.6129 | 39.73079 |
| *Lithobates catesbeianus* | -95.677 | 38.21151 |
| *Lithobates catesbeianus* | -95.2395 | 39.02831 |
| *Lithobates catesbeianus* | -95.2312 | 38.93915 |
| *Lithobates catesbeianus* | -98.0263 | 37.28667 |
| *Lithobates catesbeianus* | -101.99 | 38.0358 |
| *Lithobates catesbeianus* | -96.6335 | 39.34004 |
| *Lithobates catesbeianus* | -84.6192 | 39.58913 |
| *Lithobates catesbeianus* | -83.5454 | 39.35398 |
| *Lithobates catesbeianus* | -95.299 | 37.95917 |
| *Lithobates catesbeianus* | -95.849 | 38.09777 |
| *Lithobates catesbeianus* | -97.4063 | 39.84218 |
| *Lithobates catesbeianus* | -95.1704 | 39.63636 |
| *Lithobates catesbeianus* | -98.8093 | 37.97858 |
| *Lithobates catesbeianus* | -97.4676 | 38.47637 |
| *Lithobates catesbeianus* | -99.1591 | 39.02386 |
| *Lithobates catesbeianus* | -98.9259 | 39.66145 |
| *Lithobates catesbeianus* | -119.663 | 34.41944 |
| *Lithobates catesbeianus* | -120.569 | 35.345 |
| *Lithobates catesbeianus* | -120.389 | 35.29417 |
| *Lithobates catesbeianus* | -82.2484 | 29.7293 |
| *Lithobates catesbeianus* | -82.3351 | 29.5774 |
| *Lithobates catesbeianus* | -83.2232 | 30.11354 |
| *Lithobates catesbeianus* | -81.7796 | 27.9718 |
| *Lithobates catesbeianus* | -85.1183 | 30.0991 |
| *Lithobates catesbeianus* | -81.8374 | 29.54256 |
| *Lithobates catesbeianus* | -84.6658 | 30.16683 |
| *Lithobates catesbeianus* | -82.9479 | 30.44578 |
| *Lithobates catesbeianus* | -118.386 | 34.25968 |
| *Lithobates catesbeianus* | -119.299 | 35.28358 |
| *Lithobates catesbeianus* | -75.3754 | 39.5458 |
| *Lithobates catesbeianus* | -82.2745 | 28.1006 |
| *Lithobates catesbeianus* | -117.909 | 33.60843 |
| *Lithobates catesbeianus* | -117.351 | 33.70168 |
| *Lithobates catesbeianus* | -115.979 | 33.53394 |
| *Lithobates catesbeianus* | -118.287 | 34.01465 |
| *Lithobates catesbeianus* | 132.836 | 33.08514 |
| *Lithobates catesbeianus* | -116.35 | 36.5 |
| *Lithobates catesbeianus* | -122.176 | 37.42246 |
| *Lithobates catesbeianus* | -111.949 | 33.4511 |
| *Lithobates catesbeianus* | -109.279 | 31.337 |
| *Lithobates catesbeianus* | -96.0316 | 41.52651 |
| *Lithobates catesbeianus* | -109.85 | 32.8139 |
| *Lithobates catesbeianus* | -65.9158 | 18.40368 |
| *Lithobates catesbeianus* | -65.8188 | 18.12436 |
| *Lithobates catesbeianus* | -67.1704 | 18.0109 |
| *Lithobates catesbeianus* | -122.398 | 45.56551 |
| *Lithobates catesbeianus* | -123.065 | 40.74959 |
| *Lithobates catesbeianus* | -105.202 | 39.97071 |
| *Lithobates catesbeianus* | -95.1176 | 31.38592 |
| *Lithobates catesbeianus* | -96.4372 | 30.67472 |
| *Lithobates catesbeianus* | -96.37 | 30.67944 |
| *Lithobates catesbeianus* | -95.3444 | 30.93565 |
| *Lithobates catesbeianus* | -73.0519 | 41.55806 |
| *Lithobates catesbeianus* | -97.3264 | 32.17926 |
| *Lithobates catesbeianus* | -97.2907 | 30.11036 |
| *Lithobates catesbeianus* | -99.3412 | 31.31236 |
| *Lithobates catesbeianus* | -94.1535 | 32.67487 |
| *Lithobates catesbeianus* | -93.7136 | 30.11278 |
| *Lithobates catesbeianus* | -83.8829 | 43.65876 |
| *Lithobates catesbeianus* | -80.8688 | 36.8708 |
| *Lithobates catesbeianus* | -116.757 | 37.03134 |
| *Lithobates catesbeianus* | -70.445 | 19.01902 |
| *Lithobates catesbeianus* | -72.2995 | 42.4765 |
| *Lithobates catesbeianus* | -91.1287 | 43.05165 |
| *Lithobates catesbeianus* | -89.0603 | 37.20044 |
| *Lithobates catesbeianus* | -91.3802 | 40.39048 |
| *Lithobates catesbeianus* | -77.14 | 38.84 |
| *Lithobates catesbeianus* | -77.52 | 37.38 |
| *Lithobates catesbeianus* | -76.5936 | 38.49398 |
| *Lithobates catesbeianus* | -76.7632 | 37.3516 |
| *Lithobates catesbeianus* | -82.7504 | 36.7001 |
| *Lithobates catesbeianus* | -77.52 | 37.63 |
| *Lithobates catesbeianus* | -77.0164 | 34.93283 |
| *Lithobates catesbeianus* | -77.58 | 37.78 |
| *Lithobates catesbeianus* | -76.42 | 36.86 |
| *Lithobates catesbeianus* | -78.15 | 37.47 |
| *Lithobates catesbeianus* | -77.61 | 36.6 |
| *Lithobates catesbeianus* | -77.54 | 37.64 |
| *Lithobates catesbeianus* | -78.19 | 37.71 |
| *Lithobates catesbeianus* | -118.288 | 34.25222 |
| *Lithobates catesbeianus* | -103.154 | 43.9364 |
| *Lithobates catesbeianus* | -75.6523 | 35.9821 |
| *Lithobates catesbeianus* | -75.6613 | 35.9829 |
| *Lithobates catesbeianus* | -75.5684 | 35.2472 |
| *Lithobates catesbeianus* | -79.0136 | 36.0676 |
| *Lithobates catesbeianus* | -74.1475 | 39.9455 |
| *Lithobates catesbeianus* | -78.6143 | 34.8711 |
| *Lithobates catesbeianus* | -79.0713 | 36.0604 |
| *Lithobates catesbeianus* | -76.4328 | 36.8964 |
| *Lithobates catesbeianus* | -77.4786 | 37.7164 |
| *Lithobates catesbeianus* | -77.4889 | 37.6908 |
| *Lithobates catesbeianus* | -77.3612 | 37.5092 |
| *Lithobates catesbeianus* | -77.887 | 37.2308 |
| *Lithobates catesbeianus* | -77.4799 | 37.759 |
| *Lithobates catesbeianus* | -82.5143 | 35.8113 |
| *Lithobates catesbeianus* | -75.7993 | 35.8559 |
| *Lithobates catesbeianus* | -73.9597 | 40.9837 |
| *Lithobates catesbeianus* | -78.9761 | 42.95472 |
| *Lithobates catesbeianus* | -78.0743 | 44.26782 |
| *Lithobates catesbeianus* | -78.1333 | 45.66 |
| *Lithobates catesbeianus* | -79.2137 | 43.08907 |
| *Lithobates catesbeianus* | -121.769 | 38.5589 |
| *Lithobates catesbeianus* | -73 | 46.65 |
| *Lithobates catesbeianus* | -79.833 | 45.65 |
| *Lithobates catesbeianus* | -74.533 | 45.75 |
| *Lithobates catesbeianus* | -76.25 | 45.45 |
| *Lithobates catesbeianus* | -75.55 | 45.65 |
| *Lithobates catesbeianus* | -77.011 | 44.01 |
| *Lithobates catesbeianus* | -77.054 | 44.022 |
| *Lithobates catesbeianus* | -73.433 | 46.017 |
| *Lithobates catesbeianus* | -82.45 | 42.3 |
| *Lithobates catesbeianus* | -75.833 | 45.471 |
| *Lithobates catesbeianus* | -76.203 | 45.641 |
| *Lithobates catesbeianus* | -75.854 | 45.508 |
| *Lithobates catesbeianus* | -75.881 | 45.506 |
| *Lithobates catesbeianus* | -76.056 | 45.601 |
| *Lithobates catesbeianus* | -73.317 | 45.55 |
| *Lithobates catesbeianus* | -76.013 | 45.608 |
| *Lithobates catesbeianus* | -76.12 | 45.61 |
| *Lithobates catesbeianus* | -75.917 | 45.983 |
| *Lithobates catesbeianus* | -76.105 | 45.628 |
| *Lithobates catesbeianus* | -67.083 | 46.267 |
| *Lithobates catesbeianus* | -75.69 | 45.385 |
| *Lithobates catesbeianus* | -75.788 | 46.197 |
| *Lithobates catesbeianus* | -81.731 | 46.201 |
| *Lithobates catesbeianus* | -76.512 | 45.66 |
| *Lithobates catesbeianus* | -76.423 | 45.521 |
| *Lithobates catesbeianus* | -80.417 | 42.583 |
| *Lithobates catesbeianus* | -76.193 | 45.897 |
| *Lithobates catesbeianus* | -76.467 | 44.6 |
| *Lithobates catesbeianus* | -81.7 | 46.25 |
| *Lithobates catesbeianus* | -64.4 | 44.933 |
| *Lithobates catesbeianus* | -79.21 | 45.32 |
| *Lithobates catesbeianus* | -78.9796 | 39.43 |
| *Lithobates catesbeianus* | -75.59 | 39.87 |
| *Lithobates catesbeianus* | -72.42 | 44.27 |
| *Lithobates catesbeianus* | -82.35 | 29.63 |
| *Lithobates catesbeianus* | -75.22 | 38.29 |
| *Lithobates catesbeianus* | -75.2 | 40.02 |
| *Lithobates catesbeianus* | -80.4597 | 39.09 |
| *Lithobates catesbeianus* | -79.67 | 41.1 |
| *Lithobates catesbeianus* | -79.44 | 41.7 |
| *Lithobates catesbeianus* | -108.55 | 39.07 |
| *Lithobates catesbeianus* | -79.13 | 41.61 |
| *Lithobates catesbeianus* | -76.13 | 36.75 |
| *Lithobates catesbeianus* | -76.01 | 36.9 |
| *Lithobates catesbeianus* | -83.0002 | 40.2503 |
| *Lithobates catesbeianus* | -82.8912 | 40.0806 |
| *Lithobates catesbeianus* | -75.4999 | 43.0004 |
| *Lithobates catesbeianus* | -106.212 | 31.47456 |
| *Lithobates catesbeianus* | -80.1112 | 40.33879 |
| *Lithobates catesbeianus* | -79.6986 | 40.73568 |
| *Lithobates catesbeianus* | -94.8301 | 36.65977 |
| *Lithobates catesbeianus* | -98.6783 | 34.74757 |
| *Lithobates catesbeianus* | -98.6794 | 34.71197 |
| *Lithobates catesbeianus* | -97.1017 | 33.7583 |
| *Lithobates catesbeianus* | -97.1513 | 33.66281 |
| *Lithobates catesbeianus* | -98.233 | 34.52538 |
| *Lithobates catesbeianus* | -98.4136 | 34.57978 |
| *Lithobates catesbeianus* | -96.002 | 36.24499 |
| *Lithobates catesbeianus* | -100.616 | 36.90703 |
| *Lithobates catesbeianus* | -98.3623 | 34.65757 |
| *Lithobates catesbeianus* | -97.4347 | 35.20937 |
| *Lithobates catesbeianus* | -122.304 | 48.7003 |
| *Lithobates catesbeianus* | -97.0492 | 34.92038 |
| *Lithobates catesbeianus* | -94.8246 | 36.09479 |
| *Lithobates catesbeianus* | -97.4346 | 35.92035 |
| *Lithobates catesbeianus* | -94.8602 | 36.08029 |
| *Lithobates catesbeianus* | -99.7212 | 35.88815 |
| *Lithobates catesbeianus* | -99.639 | 35.89752 |
| *Lithobates catesbeianus* | -76.9783 | 37.6194 |
| *Lithobates catesbeianus* | -76.9628 | 37.6017 |
| *Lithobates catesbeianus* | -120.051 | 37.63409 |
| *Lithobates catesbeianus* | -122.058 | 41.76686 |
| *Lithobates catesbeianus* | -96.9971 | 32.61337 |
| *Lithobates catesbeianus* | -118.411 | 34.11978 |
| *Lithobates catesbeianus* | -71.0541 | 44.02976 |
| *Lithobates catesbeianus* | -120.562 | 35.37826 |
| *Lithobates catesbeianus* | -122.052 | 39.15321 |
| *Lithobates catesbeianus* | -88.8426 | 37.54775 |
| *Lithobates catesbeianus* | -95.6869 | 31.654 |
| *Lithobates catesbeianus* | -87.2691 | 41.59816 |
| *Lithobates catesbeianus* | -122.75 | 45.8 |
| *Lithobates catesbeianus* | -116.225 | 35.8393 |
| *Lithobates catesbeianus* | -118.113 | 36.7404 |
| *Lithobates catesbeianus* | -111.033 | 31.39805 |
| *Lithobates catesbeianus* | -87.4138 | 39.46879 |
| *Lithobates catesbeianus* | -121.539 | 39.64404 |
| *Lithobates catesbeianus* | -90.0539 | 29.95492 |
| *Lithobates catesbeianus* | -84.3055 | 33.7673 |
| *Lithobates catesbeianus* | -71.2931 | 42.2964 |
| *Lithobates catesbeianus* | -71.2135 | 42.74009 |
| *Lithobates catesbeianus* | -122.487 | 47.44723 |
| *Lithobates catesbeianus* | -122.713 | 47.07262 |
| *Lithobates catesbeianus* | -94.3913 | 29.5729 |
| *Lithobates catesbeianus* | -75.9149 | 44.20945 |
| *Lithobates catesbeianus* | -94.3985 | 30.3213 |
| *Lithobates catesbeianus* | -76.5926 | 39.24896 |
| *Lithobates catesbeianus* | -76.7193 | 38.75404 |
| *Lithobates catesbeianus* | -97.6781 | 37.5369 |
| *Lithobates catesbeianus* | -69.1757 | 44.336 |
| *Lithobates catesbeianus* | -74.5799 | 40.27108 |
| *Lithobates catesbeianus* | -122.664 | 38.18939 |
| *Lithobates catesbeianus* | -97.7368 | 30.16933 |
| *Lithobates catesbeianus* | -96.8049 | 28.30614 |
| *Lithobates catesbeianus* | -122.009 | 37.10363 |
| *Lithobates catesbeianus* | -77.1049 | 38.75449 |
| *Lithobates catesbeianus* | -121.671 | 37.29144 |
| *Lithobates catesbeianus* | -92.4903 | 34.8387 |
| *Lithobates catesbeianus* | -71.5589 | 41.86026 |
| *Lithobates catesbeianus* | -70.815 | 42.66733 |
| *Lithobates catesbeianus* | -97.8049 | 30.24345 |
| *Lithobates catesbeianus* | -68.2452 | 44.39995 |
| *Lithobates catesbeianus* | -121.494 | 38.371 |
| *Lithobates catesbeianus* | -118.211 | 34.20099 |
| *Lithobates catesbeianus* | -95.7054 | 29.34489 |
| *Lithobates catesbeianus* | -122.326 | 48.709 |
| *Lithobates catesbeianus* | -91.1167 | 30.37745 |
| *Lithobates catesbeianus* | -98.5118 | 33.88108 |
| *Lithobates catesbeianus* | -124.016 | 49.20518 |
| *Lithobates catesbeianus* | -94.5296 | 29.60512 |
| *Lithobates catesbeianus* | -97.2497 | 30.52412 |
| *Lithobates catesbeianus* | -76.138 | 41.09547 |
| *Lithobates catesbeianus* | -77.5679 | 45.65916 |
| *Lithobates catesbeianus* | -120.539 | 38.06778 |
| *Lithobates catesbeianus* | 126.346 | 36.71783 |
| *Lithobates catesbeianus* | 126.6432 | 36.66173 |
| *Lithobates catesbeianus* | -73.4653 | 44.69164 |
| *Lithobates catesbeianus* | -95.9817 | 30.10185 |
| *Lithobates catesbeianus* | -121.723 | 37.29137 |
| *Lithobates catesbeianus* | -123.179 | 49.21771 |
| *Lithobates catesbeianus* | -79.1481 | 33.4732 |
| *Lithobates catesbeianus* | -89.4317 | 36.37041 |
| *Lithobates catesbeianus* | -76.3367 | 42.22829 |
| *Lithobates catesbeianus* | -77.9368 | 34.20973 |
| *Lithobates catesbeianus* | -70.8883 | 44.26437 |
| *Lithobates catesbeianus* | -82.3942 | 34.8525 |
| *Lithobates catesbeianus* | -85.4186 | 35.21399 |
| *Lithobates catesbeianus* | -73.1666 | 41.7094 |
| *Lithobates catesbeianus* | 117.05 | 23.5 |
| *Lithobates catesbeianus* | -95.8783 | 34.42796 |
| *Lithobates catesbeianus* | -95.4839 | 34.19843 |
| *Lithobates catesbeianus* | -78.2589 | 34.7404 |
| *Lithobates catesbeianus* | -81.8948 | 28.77759 |
| *Lithobates catesbeianus* | -84.117 | 30.4366 |
| *Lithobates catesbeianus* | -84.3166 | 30.43353 |
| *Lithobates catesbeianus* | -85.8379 | 30.5605 |
| *Lithobates catesbeianus* | -81.9886 | 29.689 |
| *Lithobates catesbeianus* | -82.6487 | 29.8258 |
| *Lithobates catesbeianus* | -76.4375 | 42.5035 |
| *Lithobates catesbeianus* | -76.2291 | 39.0332 |
| *Lithobates catesbeianus* | -122.471 | 37.78792 |
| *Lithobates catesbeianus* | -97.4327 | 35.52229 |
| *Lithobates catesbeianus* | 5.12754 | 51.14333 |
| *Lithobates catesbeianus* | 5.12895 | 51.14441 |
| *Lithobates catesbeianus* | 5.12895 | 51.14386 |
| *Lithobates catesbeianus* | 5.12753 | 51.14336 |
| *Lithobates catesbeianus* | -78.1833 | 44.68333 |
| *Lithobates catesbeianus* | -81.5135 | 43.96088 |
| *Lithobates catesbeianus* | -77.6739 | 46.17995 |
| *Lithobates catesbeianus* | -67.2652 | 44.74809 |
| *Lithobates catesbeianus* | -95.05 | 29.57 |
| *Lithobates catesbeianus* | -97.1698 | 30.08747 |
| *Lithobates catesbeianus* | -79.3392 | 37.43177 |
| *Lithobates catesbeianus* | -97.6272 | 30.54003 |
| *Lithobates catesbeianus* | -74.777 | 40.32146 |
| *Lithobates catesbeianus* | -111.004 | 32.13386 |
| *Lithobates catesbeianus* | -100.39 | 19.42 |
| *Lithobates catesbeianus* | -76.933 | 44.717 |
| *Lithobates catesbeianus* | -76.45 | 44.85 |
| *Lithobates catesbeianus* | -75.65 | 45.617 |
| *Lithobates catesbeianus* | -76.65 | 45.05 |
| *Lithobates catesbeianus* | -75.767 | 45.233 |
| *Lithobates catesbeianus* | -75.3 | 45.533 |
| *Lithobates catesbeianus* | -77.047 | 44.907 |
| *Lithobates catesbeianus* | -75.669 | 44.986 |
| *Lithobates catesbeianus* | -76.206 | 45.592 |
| *Lithobates catesbeianus* | -75.869 | 45.539 |
| *Lithobates catesbeianus* | -75.833 | 45.522 |
| *Lithobates catesbeianus* | -76.163 | 45.656 |
| *Lithobates catesbeianus* | -76.044 | 45.603 |
| *Lithobates catesbeianus* | -75.851 | 45.519 |
| *Lithobates catesbeianus* | -75.701 | 45.442 |
| *Lithobates catesbeianus* | -76.128 | 43.568 |
| *Lithobates catesbeianus* | -77.276 | 45.903 |
| *Lithobates catesbeianus* | -76.757 | 45.565 |
| *Lithobates catesbeianus* | -76.633 | 46.367 |
| *Lithobates catesbeianus* | -76.367 | 45.683 |
| *Lithobates catesbeianus* | -75.698 | 44.869 |
| *Lithobates catesbeianus* | -76.129 | 45.531 |
| *Lithobates catesbeianus* | -75.838 | 46.173 |
| *Lithobates catesbeianus* | -73.316 | 45.551 |
| *Lithobates catesbeianus* | -75.96 | 45.393 |
| *Lithobates catesbeianus* | -76.167 | 45.917 |
| *Lithobates catesbeianus* | -75.6 | 45.4 |
| *Lithobates catesbeianus* | -76.283 | 45.317 |
| *Lithobates catesbeianus* | -82.05 | 46.117 |
| *Lithobates catesbeianus* | -78.67 | 40.89 |
| *Lithobates catesbeianus* | -80.21 | 40.9 |
| *Lithobates catesbeianus* | -82.88 | 35.13 |
| *Lithobates catesbeianus* | -78.68 | 35.96 |
| *Lithobates catesbeianus* | -77.9 | 40.03 |
| *Lithobates catesbeianus* | -75.62 | 38.32 |
| *Lithobates catesbeianus* | -78.36 | 42.08 |
| *Lithobates catesbeianus* | -75.09 | 40.43 |
| *Lithobates catesbeianus* | -76.97 | 40.36 |
| *Lithobates catesbeianus* | -78.52 | 41.75 |
| *Lithobates catesbeianus* | -79.46 | 40.52 |
| *Lithobates catesbeianus* | -82.24 | 33.34 |
| *Lithobates catesbeianus* | -76.48 | 42.43 |
| *Lithobates catesbeianus* | -95.9001 | 36.06003 |
| *Lithobates catesbeianus* | -104.99 | 39.56 |
| *Lithobates catesbeianus* | -91.74 | 30.53 |
| *Lithobates catesbeianus* | -94.8081 | 37.64784 |
| *Lithobates catesbeianus* | -75.97 | 46.56 |
| *Lithobates catesbeianus* | -78.05 | 40.81 |
| *Lithobates catesbeianus* | -76.84 | 40.94 |
| *Lithobates catesbeianus* | -78.86 | 41.39 |
| *Lithobates catesbeianus* | -76.9 | 37.42 |
| *Lithobates catesbeianus* | -76.6 | 37.27 |
| *Lithobates catesbeianus* | -96.0532 | 37.71631 |
| *Lithobates catesbeianus* | -97.0406 | 37.28129 |
| *Lithobates catesbeianus* | -94.7475 | 38.35406 |
| *Lithobates catesbeianus* | -100.91 | 43.23 |
| *Lithobates catesbeianus* | -99.8163 | 38.79357 |
| *Lithobates catesbeianus* | -95.299 | 37.82806 |
| *Lithobates catesbeianus* | -95.1365 | 38.02361 |
| *Lithobates catesbeianus* | -96.0093 | 38.52137 |
| *Lithobates catesbeianus* | -100.917 | 38.66385 |
| *Lithobates catesbeianus* | -114.408 | 34.1118 |
| *Lithobates catesbeianus* | -114.027 | 37.02448 |
| *Lithobates catesbeianus* | -108.737 | 32.02508 |
| *Lithobates catesbeianus* | -118.251 | 35.68801 |
| *Lithobates catesbeianus* | -109.649 | 32.83141 |
| *Lithobates catesbeianus* | -158.238 | 21.56582 |
| *Lithobates catesbeianus* | -116.873 | 32.72375 |
| *Lithobates catesbeianus* | -114.71 | 32.73285 |
| *Lithobates catesbeianus* | -66.4921 | 18.42745 |
| *Lithobates catesbeianus* | -114.742 | 36.57002 |
| *Lithobates catesbeianus* | -114.754 | 36.5943 |
| *Lithobates catesbeianus* | -114.127 | 45.93992 |
| *Lithobates catesbeianus* | -105.672 | 37.5661 |
| *Lithobates catesbeianus* | -118.022 | 34.48559 |
| *Lithobates catesbeianus* | -109.04 | 25.63031 |
| *Lithobates catesbeianus* | -81.7094 | 36.14328 |
| *Lithobates catesbeianus* | -95.1018 | 31.42772 |
| *Lithobates catesbeianus* | -72.2342 | 41.76583 |
| *Lithobates catesbeianus* | -73.3831 | 41.70639 |
| *Lithobates catesbeianus* | -97.3241 | 31.09183 |
| *Lithobates catesbeianus* | -97.3877 | 28.70292 |
| *Lithobates catesbeianus* | -96.3781 | 30.64556 |
| *Lithobates catesbeianus* | -98.3211 | 33.96278 |
| *Lithobates catesbeianus* | -98.4547 | 33.87783 |
| *Lithobates catesbeianus* | -94.8811 | 30.26415 |
| *Lithobates catesbeianus* | -98.7269 | 38.44914 |
| *Lithobates catesbeianus* | -72.2 | 19.75 |
| *Lithobates catesbeianus* | -85.3197 | 42.5471 |
| *Lithobates catesbeianus* | -82.6033 | 42.597 |
| *Lithobates catesbeianus* | -95.6602 | 46.9262 |
| *Lithobates catesbeianus* | -76.2677 | 43.2428 |
| *Lithobates catesbeianus* | -79.6044 | 43.5183 |
| *Lithobates catesbeianus* | -78.3251 | 44.1077 |
| *Lithobates catesbeianus* | -79.1217 | 42.9981 |
| *Lithobates catesbeianus* | -78.3363 | 44.2182 |
| *Lithobates catesbeianus* | -99.283 | 18.469 |
| *Lithobates catesbeianus* | -82.9205 | 39.35394 |
| *Lithobates catesbeianus* | -98.4623 | 40.79217 |
| *Lithobates catesbeianus* | -83.991 | 39.23717 |
| *Lithobates catesbeianus* | -84.4514 | 41.427 |
| *Lithobates catesbeianus* | -84.1853 | 40.115 |
| *Lithobates catesbeianus* | -83.7352 | 39.3171 |
| *Lithobates catesbeianus* | -82.0896 | 39.16884 |
| *Lithobates catesbeianus* | -97.2336 | 33.76736 |
| *Lithobates catesbeianus* | -96.0437 | 28.69029 |
| *Lithobates catesbeianus* | -88.9605 | 34.79285 |
| *Lithobates catesbeianus* | -86.2503 | 40.0003 |
| *Lithobates catesbeianus* | -82.5723 | 39.477 |
| *Lithobates catesbeianus* | -83.4755 | 39.3018 |
| *Lithobates catesbeianus* | -82.8536 | 38.867 |
| *Lithobates catesbeianus* | -120.548 | 34.67943 |
| *Lithobates catesbeianus* | -120.555 | 34.67691 |
| *Lithobates catesbeianus* | -99.9886 | 28.10115 |
| *Lithobates catesbeianus* | -81.4383 | 31.7221 |
| *Lithobates catesbeianus* | -84.9854 | 36.18287 |
| *Lithobates catesbeianus* | -97.0663 | 37.3018 |
| *Lithobates catesbeianus* | -76.9241 | 38.999 |
| *Lithobates catesbeianus* | -104.457 | 32.10743 |
| *Lithobates catesbeianus* | -95.6779 | 36.57147 |
| *Lithobates catesbeianus* | -73.0624 | 41.3099 |
| *Lithobates catesbeianus* | -82.4976 | 33.789 |
| *Lithobates catesbeianus* | -71.3494 | 42.46754 |
| *Lithobates catesbeianus* | -98.3531 | 35.44642 |
| *Lithobates catesbeianus* | -76.9176 | 38.99698 |
| *Lithobates catesbeianus* | -78.7497 | 38.8619 |
| *Lithobates catesbeianus* | -77.3779 | 39.10644 |
| *Lithobates catesbeianus* | -80.3548 | 37.24729 |
| *Lithobates catesbeianus* | -77.57 | 37.67 |
| *Lithobates catesbeianus* | -83.3149 | 35.4743 |
| *Lithobates catesbeianus* | -78.18 | 37.66 |
| *Lithobates catesbeianus* | -78.86 | 37.19 |
| *Lithobates catesbeianus* | -82.9544 | 36.773 |
| *Lithobates catesbeianus* | -75.77 | 37.68 |
| *Lithobates catesbeianus* | -94.7373 | 34.03091 |
| *Lithobates catesbeianus* | -94.7258 | 35.73299 |
| *Lithobates catesbeianus* | -95.3072 | 34.9335 |
| *Lithobates catesbeianus* | -94.7386 | 34.8622 |
| *Lithobates catesbeianus* | -97.2972 | 34.70114 |
| *Lithobates catesbeianus* | -98.3736 | 34.47798 |
| *Lithobates catesbeianus* | -97.4689 | 35.14457 |
| *Lithobates catesbeianus* | -96.2407 | 34.55589 |
| *Lithobates catesbeianus* | -95.9551 | 35.5684 |
| *Lithobates catesbeianus* | -96.7864 | 31.0183 |
| *Lithobates catesbeianus* | -108.993 | 25.79353 |
| *Lithobates catesbeianus* | -95.0734 | 35.9641 |
| *Lithobates catesbeianus* | -94.8809 | 36.77687 |
| *Lithobates catesbeianus* | -97.3457 | 34.81927 |
| *Lithobates catesbeianus* | -96.2376 | 34.97899 |
| *Lithobates catesbeianus* | -77.9865 | 34.1945 |
| *Lithobates catesbeianus* | -81.7791 | 35.8584 |
| *Lithobates catesbeianus* | -78.6641 | 35.3758 |
| *Lithobates catesbeianus* | -76.6266 | 34.9373 |
| *Lithobates catesbeianus* | -83.6996 | 35.1657 |
| *Lithobates catesbeianus* | -78.5968 | 34.148 |
| *Lithobates catesbeianus* | -79.0149 | 35.4027 |
| *Lithobates catesbeianus* | -79.4633 | 35.798 |
| *Lithobates catesbeianus* | -74.5853 | 41.01 |
| *Lithobates catesbeianus* | -80.5699 | 32.9315 |
| *Lithobates catesbeianus* | -77.2906 | 37.5761 |
| *Lithobates catesbeianus* | -77.5994 | 37.7147 |
| *Lithobates catesbeianus* | -77.4469 | 37.6864 |
| *Lithobates catesbeianus* | -77.4419 | 37.6942 |
| *Lithobates catesbeianus* | -77.4608 | 37.6922 |
| *Lithobates catesbeianus* | -79.562 | 35.0992 |
| *Lithobates catesbeianus* | -80.7747 | 35.4575 |
| *Lithobates catesbeianus* | -77.6811 | 37.4694 |
| *Lithobates catesbeianus* | -77.7317 | 37.5785 |
| *Lithobates catesbeianus* | -79.9553 | 34.8843 |
| *Lithobates catesbeianus* | -79.9506 | 32.7936 |
| *Lithobates catesbeianus* | -77.7132 | 43.9938 |
| *Lithobates catesbeianus* | -77.9 | 45.51667 |
| *Lithobates catesbeianus* | -76.8833 | 44.08333 |
| *Lithobates catesbeianus* | -80.0333 | 45.35 |
| *Lithobates catesbeianus* | -80.2016 | 46.23751 |
| *Lithobates catesbeianus* | -78.5833 | 44.65 |
| *Lithobates catesbeianus* | -82.4471 | 42.512 |
| *Lithobates catesbeianus* | -76.6875 | 39.88125 |
| *Lithobates catesbeianus* | -88.3459 | 41.73059 |
| *Lithobates catesbeianus* | -96.1178 | 30.5425 |
| *Lithobates catesbeianus* | -119.707 | 37.36458 |
| *Lithobates catesbeianus* | -117.257 | 34.50009 |
| *Lithobates catesbeianus* | -117.295 | 34.54922 |
| *Lithobates catesbeianus* | -122.13 | 37.31941 |
| *Lithobates catesbeianus* | -122.045 | 40.10741 |
| *Lithobates catesbeianus* | -121.702 | 36.96302 |
| *Lithobates catesbeianus* | -119.739 | 37.09946 |
| *Lithobates catesbeianus* | -121.288 | 38.75208 |
| *Lithobates catesbeianus* | -121.238 | 37.97208 |
| *Lithobates catesbeianus* | -97.8674 | 38.77497 |
| *Lithobates catesbeianus* | -122.825 | 38.48573 |
| *Lithobates catesbeianus* | -121.766 | 36.2303 |
| *Lithobates catesbeianus* | -121.356 | 39.0961 |
| *Lithobates catesbeianus* | -121.782 | 37.18343 |
| *Lithobates catesbeianus* | -71.2016 | 44.6104 |
| *Lithobates catesbeianus* | -72.29 | 43.7022 |
| *Lithobates catesbeianus* | -122.722 | 38.85633 |
| *Lithobates catesbeianus* | -99.3907 | 38.8581 |
| *Lithobates catesbeianus* | -100.912 | 38.6841 |
| *Lithobates catesbeianus* | -100.908 | 38.6827 |
| *Lithobates catesbeianus* | -95.197 | 37.4164 |
| *Lithobates catesbeianus* | -157.82 | 21.3462 |
| *Lithobates catesbeianus* | -118.242 | 34.10255 |
| *Lithobates catesbeianus* | -120.511 | 35.65847 |
| *Lithobates catesbeianus* | -118.675 | 35.73026 |
| *Lithobates catesbeianus* | -97.5339 | 30.24662 |
| *Lithobates catesbeianus* | -88.5424 | 44.02463 |
| *Lithobates catesbeianus* | -90.7995 | 30.16181 |
| *Lithobates catesbeianus* | -70.6294 | 41.54995 |
| *Lithobates catesbeianus* | -122.897 | 46.03742 |
| *Lithobates catesbeianus* | -122.724 | 38.84672 |
| *Lithobates catesbeianus* | -122.191 | 39.23429 |
| *Lithobates catesbeianus* | -122.494 | 38.32254 |
| *Lithobates catesbeianus* | -119.312 | 36.90809 |
| *Lithobates catesbeianus* | -72.5755 | 41.78615 |
| *Lithobates catesbeianus* | -72.3024 | 41.79643 |
| *Lithobates catesbeianus* | -76.9444 | 38.91369 |
| *Lithobates catesbeianus* | -93.6759 | 30.1371 |
| *Lithobates catesbeianus* | -72.7555 | 44.41916 |
| *Lithobates catesbeianus* | -97.9338 | 29.59008 |
| *Lithobates catesbeianus* | -96.3366 | 30.59264 |
| *Lithobates catesbeianus* | -97.7937 | 30.24806 |
| *Lithobates catesbeianus* | -122.174 | 37.36318 |
| *Lithobates catesbeianus* | -95.738 | 29.30705 |
| *Lithobates catesbeianus* | -98.4843 | 39.0119 |
| *Lithobates catesbeianus* | -122.817 | 38.08493 |
| *Lithobates catesbeianus* | -122.645 | 48.39301 |
| *Lithobates catesbeianus* | -71.6584 | 43.09941 |
| *Lithobates catesbeianus* | -75.495 | 45.50356 |
| *Lithobates catesbeianus* | -71.763 | 42.78522 |
| *Lithobates catesbeianus* | -71.3368 | 42.2543 |
| *Lithobates catesbeianus* | -69.9958 | 41.88171 |
| *Lithobates catesbeianus* | -97.4479 | 30.32956 |
| *Lithobates catesbeianus* | -116.191 | 32.61986 |
| *Lithobates catesbeianus* | -122.487 | 47.44727 |
| *Lithobates catesbeianus* | -75.4848 | 45.49934 |
| *Lithobates catesbeianus* | -85.3543 | 33.92394 |
| *Lithobates catesbeianus* | -121.849 | 36.8365 |
| *Lithobates catesbeianus* | -122.893 | 45.22069 |
| *Lithobates catesbeianus* | -71.1811 | 42.43329 |
| *Lithobates catesbeianus* | -75.4994 | 45.50076 |
| *Lithobates catesbeianus* | -75.7424 | 45.73644 |
| *Lithobates catesbeianus* | -68.2079 | 44.36264 |
| *Lithobates catesbeianus* | -75.6354 | 45.46358 |
| *Lithobates catesbeianus* | -75.6254 | 45.46302 |
| *Lithobates catesbeianus* | -96.8059 | 28.3014 |
| *Lithobates catesbeianus* | -93.386 | 41.75677 |
| *Lithobates catesbeianus* | -94.1789 | 30.25058 |
| *Lithobates catesbeianus* | -96.5717 | 39.18361 |
| *Lithobates catesbeianus* | -77.1054 | 38.75339 |
| *Lithobates catesbeianus* | -77.0985 | 38.75754 |
| *Lithobates catesbeianus* | -96.2083 | 29.77978 |
| *Lithobates catesbeianus* | -95.6014 | 29.37667 |
| *Lithobates catesbeianus* | -96.2145 | 30.56956 |
| *Lithobates catesbeianus* | -102.12 | 31.967 |
| *Lithobates catesbeianus* | -72.6433 | 41.76545 |
| *Lithobates catesbeianus* | -71.4426 | 43.81166 |
| *Lithobates catesbeianus* | -81.2732 | 44.65288 |
| *Lithobates catesbeianus* | -111.735 | 33.36122 |
| *Lithobates catesbeianus* | -95.8908 | 29.88885 |
| *Lithobates catesbeianus* | -96.8025 | 28.30873 |
| *Lithobates catesbeianus* | -94.3895 | 29.57349 |
| *Lithobates catesbeianus* | -81.8853 | 41.41149 |
| *Lithobates catesbeianus* | -95.8892 | 31.9303 |
| *Lithobates catesbeianus* | -95.8533 | 31.86481 |
| *Lithobates catesbeianus* | 126.4248 | 36.70779 |
| *Lithobates catesbeianus* | -123.135 | 49.29415 |
| *Lithobates catesbeianus* | -94.0383 | 34.53225 |
| *Lithobates catesbeianus* | -97.4762 | 35.59612 |
| *Lithobates catesbeianus* | -94.6605 | 33.73669 |
| *Lithobates catesbeianus* | -81.3044 | 41.60685 |
| *Lithobates catesbeianus* | -122.224 | 37.37166 |
| *Lithobates catesbeianus* | -106.351 | 35.66765 |
| *Lithobates catesbeianus* | -122.264 | 37.91028 |
| *Lithobates catesbeianus* | -71.1497 | 42.38856 |
| *Lithobates catesbeianus* | -121.718 | 37.34466 |
| *Lithobates catesbeianus* | -98.535 | 33.8612 |
| *Lithobates catesbeianus* | -71.3947 | 41.93209 |
| *Lithobates catesbeianus* | -71.4503 | 41.63833 |
| *Lithobates catesbeianus* | -71.1165 | 42.37809 |
| *Lithobates catesbeianus* | -71.5667 | 42.3917 |
| *Lithobates catesbeianus* | -71.8985 | 42.1881 |
| *Lithobates catesbeianus* | -70.0225 | 41.7539 |
| *Lithobates catesbeianus* | -74.1377 | 42.5162 |
| *Lithobates catesbeianus* | -76.4853 | 42.44229 |
| *Lithobates catesbeianus* | -73.1958 | 42.2833 |
| *Lithobates catesbeianus* | -73.8178 | 40.76531 |
| *Lithobates catesbeianus* | -95.6008 | 33.1383 |
| *Lithobates catesbeianus* | -79.436 | 34.6726 |
| *Lithobates catesbeianus* | -81.7423 | 28.00417 |
| *Lithobates catesbeianus* | -83.3099 | 29.72461 |
| *Lithobates catesbeianus* | -81.7468 | 29.4187 |
| *Lithobates catesbeianus* | 5.12784 | 51.14489 |
| *Lithobates catesbeianus* | 5.12995 | 51.14407 |
| *Lithobates catesbeianus* | 5.12876 | 51.14355 |
| *Lithobates catesbeianus* | 5.12976 | 51.14373 |
| *Lithobates catesbeianus* | -96.805 | 31.02133 |
| *Lithobates catesbeianus* | -95.0071 | 31.11384 |
| *Lithobates catesbeianus* | -81.4202 | 35.7927 |
| *Lithobates catesbeianus* | -96.2149 | 30.52508 |
| *Lithobates catesbeianus* | -96.1953 | 38.85026 |
| *Lithobates catesbeianus* | -79.2671 | 40.16307 |
| *Lithobates catesbeianus* | -110.355 | 31.31629 |
| *Lithobates catesbeianus* | -97.9333 | 29.88333 |
| *Lithobates catesbeianus* | -76.5836 | 39.09928 |
| *Lithobates catesbeianus* | -95.3324 | 35.0226 |
| *Lithobates catesbeianus* | -82.6502 | 41.77131 |
| *Lithobates catesbeianus* | -79.9167 | 45.61667 |
| *Lithobates catesbeianus* | -79.6 | 46.9333 |
| *Lithobates catesbeianus* | -78.8 | 44.71666 |
| *Lithobates catesbeianus* | -116.663 | 32.88881 |
| *Lithobates catesbeianus* | -111.247 | 31.45965 |
| *Lithobates catesbeianus* | -116.903 | 32.6332 |
| *Lithobates catesbeianus* | -75.7069 | 5.75972 |
| *Lithobates catesbeianus* | -76.183 | 45.517 |
| *Lithobates catesbeianus* | -79.1 | 45.317 |
| *Lithobates catesbeianus* | -65.133 | 44.367 |
| *Lithobates catesbeianus* | -72.333 | 45.667 |
| *Lithobates catesbeianus* | -81.55 | 46.7 |
| *Lithobates catesbeianus* | -75.25 | 45.367 |
| *Lithobates catesbeianus* | -75.417 | 45.583 |
| *Lithobates catesbeianus* | -75.31 | 45.547 |
| *Lithobates catesbeianus* | -76.917 | 44.75 |
| *Lithobates catesbeianus* | -75.797 | 45.372 |
| *Lithobates catesbeianus* | -76.255 | 45.592 |
| *Lithobates catesbeianus* | -72.333 | 45.117 |
| *Lithobates catesbeianus* | -75.864 | 45.492 |
| *Lithobates catesbeianus* | -76.183 | 45.683 |
| *Lithobates catesbeianus* | -76.196 | 45.632 |
| *Lithobates catesbeianus* | -76.349 | 45.341 |
| *Lithobates catesbeianus* | -75.9 | 41.033 |
| *Lithobates catesbeianus* | -75.664 | 44.982 |
| *Lithobates catesbeianus* | -75.833 | 46.179 |
| *Lithobates catesbeianus* | -75.829 | 46.1 |
| *Lithobates catesbeianus* | -71.3 | 42.417 |
| *Lithobates catesbeianus* | -80.317 | 43.367 |
| *Lithobates catesbeianus* | -76.9 | 45 |
| *Lithobates catesbeianus* | -94.7674 | 38.92728 |
| *Lithobates catesbeianus* | -97.7129 | 37.25611 |
| *Lithobates catesbeianus* | -96.2248 | 37.43944 |
| *Lithobates catesbeianus* | -94.6615 | 37.10419 |
| *Lithobates catesbeianus* | -95.9376 | 37.56861 |
| *Lithobates catesbeianus* | -98.6517 | 36.00393 |
| *Lithobates catesbeianus* | -106.825 | 32.24837 |
| *Lithobates catesbeianus* | -95.119 | 39.10942 |
| *Lithobates catesbeianus* | -96.1451 | 37.0214 |
| *Lithobates catesbeianus* | -94.7876 | 36.93618 |
| *Lithobates catesbeianus* | -80.16 | 40.51 |
| *Lithobates catesbeianus* | -78.0196 | 39.55999 |
| *Lithobates catesbeianus* | -70.6294 | 41.90995 |
| *Lithobates catesbeianus* | -82.01 | 29.59 |
| *Lithobates catesbeianus* | -79.38 | 40.2 |
| *Lithobates catesbeianus* | -75.28 | 39.87 |
| *Lithobates catesbeianus* | -77.36 | 40.03 |
| *Lithobates catesbeianus* | -78.22 | 42.23 |
| *Lithobates catesbeianus* | -75.97 | 40.32 |
| *Lithobates catesbeianus* | -75.22 | 41.71 |
| *Lithobates catesbeianus* | -81.43 | 32.11 |
| *Lithobates catesbeianus* | -78.5 | 39.05 |
| *Lithobates catesbeianus* | -76.55 | 36.61 |
| *Lithobates catesbeianus* | -95.1753 | 38.6086 |
| *Lithobates catesbeianus* | -94.7006 | 37.0491 |
| *Lithobates catesbeianus* | -95.6986 | 37.1646 |
| *Lithobates catesbeianus* | -95.6769 | 37.697 |
| *Lithobates catesbeianus* | -97.8914 | 38.72041 |
| *Lithobates catesbeianus* | -99.3658 | 37.19304 |
| *Lithobates catesbeianus* | -94.659 | 37.48406 |
| *Lithobates catesbeianus* | -122.408 | 37.26885 |
| *Lithobates catesbeianus* | -120.448 | 37.53618 |
| *Lithobates catesbeianus* | -120.919 | 40.08288 |
| *Lithobates catesbeianus* | -122.079 | 37.55417 |
| *Lithobates catesbeianus* | -121.691 | 40.08104 |
| *Lithobates catesbeianus* | -119.922 | 36.37151 |
| *Lithobates catesbeianus* | -121.667 | 38.18333 |
| *Lithobates catesbeianus* | -73.0836 | 44.8108 |
| *Lithobates catesbeianus* | -100.76 | 41.10964 |
| *Lithobates catesbeianus* | -117.695 | 33.62504 |
| *Lithobates catesbeianus* | -107.758 | 32.13945 |
| *Lithobates catesbeianus* | -117.646 | 33.89777 |
| *Lithobates catesbeianus* | -117.594 | 33.72228 |
| *Lithobates catesbeianus* | -117.285 | 33.36233 |
| *Lithobates catesbeianus* | -107.132 | 32.66528 |
| *Lithobates catesbeianus* | -111.086 | 31.40536 |
| *Lithobates catesbeianus* | -118.722 | 34.65786 |
| *Lithobates catesbeianus* | -117.318 | 33.3658 |
| *Lithobates catesbeianus* | -114.69 | 33.08001 |
| *Lithobates catesbeianus* | -118.26 | 34.07273 |
| *Lithobates catesbeianus* | -71.2769 | 43.6764 |
| *Lithobates catesbeianus* | -119.917 | 39.4333 |
| *Lithobates catesbeianus* | -124.003 | 44.96887 |
| *Lithobates catesbeianus* | -159.538 | 22.02455 |
| *Lithobates catesbeianus* | -155.09 | 19.6969 |
| *Lithobates catesbeianus* | -120.623 | 35.0056 |
| *Lithobates catesbeianus* | -109.279 | 31.33725 |
| *Lithobates catesbeianus* | -65.8074 | 18.14051 |
| *Lithobates catesbeianus* | -122.446 | 47.11038 |
| *Lithobates catesbeianus* | -95.1647 | 31.53428 |
| *Lithobates catesbeianus* | -96.503 | 30.62963 |
| *Lithobates catesbeianus* | -96.2169 | 30.62778 |
| *Lithobates catesbeianus* | -97.2706 | 31.03111 |
| *Lithobates catesbeianus* | -73.26 | 41.08917 |
| *Lithobates catesbeianus* | -97.4196 | 31.16498 |
| *Lithobates catesbeianus* | -95.7421 | 33.29633 |
| *Lithobates catesbeianus* | -102.198 | 35.48862 |
| *Lithobates catesbeianus* | -94.8435 | 33.33296 |
| *Lithobates catesbeianus* | -75.8333 | 45.25 |
| *Lithobates catesbeianus* | -81.8578 | 42.2635 |
| *Lithobates catesbeianus* | -78.6685 | 44.9796 |
| *Lithobates catesbeianus* | -77.3285 | 43.5921 |
| *Lithobates catesbeianus* | -80.9324 | 41.38955 |
| *Lithobates catesbeianus* | -81.9778 | 40.03181 |
| *Lithobates catesbeianus* | -80.5299 | 41.45566 |
| *Lithobates catesbeianus* | -81.6478 | 40.17517 |
| *Lithobates catesbeianus* | -83.9028 | 39.91153 |
| *Lithobates catesbeianus* | -83.9489 | 39.56585 |
| *Lithobates catesbeianus* | -83.0044 | 39.77285 |
| *Lithobates catesbeianus* | -122.892 | 46.1926 |
| *Lithobates catesbeianus* | -122.655 | 48.3943 |
| *Lithobates catesbeianus* | -116.786 | 47.4833 |
| *Lithobates catesbeianus* | -83.1865 | 39.0921 |
| *Lithobates catesbeianus* | -82.8468 | 40.0921 |
| *Lithobates catesbeianus* | -97.5033 | 35.4921 |
| *Lithobates catesbeianus* | -81.3061 | 31.997 |
| *Lithobates catesbeianus* | -89.4449 | 37.21912 |
| *Lithobates catesbeianus* | -122.7 | 38.58432 |
| *Lithobates catesbeianus* | -99.1595 | 33.61701 |
| *Lithobates catesbeianus* | -111.879 | 34.58677 |
| *Lithobates catesbeianus* | -119.782 | 36.96572 |
| *Lithobates catesbeianus* | -96.1263 | 36.38998 |
| *Lithobates catesbeianus* | -99.3049 | 36.45364 |
| *Lithobates catesbeianus* | -95.3411 | 34.9376 |
| *Lithobates catesbeianus* | -94.7173 | 36.83577 |
| *Lithobates catesbeianus* | -96.8672 | 33.9983 |
| *Lithobates catesbeianus* | -96.3433 | 36.65836 |
| *Lithobates catesbeianus* | -94.5634 | 34.00461 |
| *Lithobates catesbeianus* | -97.2098 | 34.44959 |
| *Lithobates catesbeianus* | -94.7109 | 34.03281 |
| *Lithobates catesbeianus* | -94.666 | 36.84927 |
| *Lithobates catesbeianus* | -99.7541 | 35.89746 |
| *Lithobates catesbeianus* | -157.788 | 21.3671 |
| *Lithobates catesbeianus* | -69.8238 | 19.34873 |
| *Lithobates catesbeianus* | -77.2789 | 39.3419 |
| *Lithobates catesbeianus* | -76.168 | 4.39938 |
| *Lithobates catesbeianus* | -86.6506 | 36.4353 |
| *Lithobates catesbeianus* | -79.6774 | 41.14649 |
| *Lithobates catesbeianus* | -92.1568 | 29.94831 |
| *Lithobates catesbeianus* | -79.2041 | 35.46619 |
| *Lithobates catesbeianus* | -81.5608 | 36.8217 |
| *Lithobates catesbeianus* | -96.5857 | 39.18997 |
| *Lithobates catesbeianus* | -78 | 38.7 |
| *Lithobates catesbeianus* | -91.23 | 43.42206 |
| *Lithobates catesbeianus* | -76.8117 | 39.0569 |
| *Lithobates catesbeianus* | -95.124 | 37.2739 |
| *Lithobates catesbeianus* | -78.4041 | 34.76553 |
| *Lithobates catesbeianus* | -80.9972 | 27.0917 |
| *Lithobates catesbeianus* | -82.3689 | 35.89508 |
| *Lithobates catesbeianus* | -78.1115 | 39.68523 |
| *Lithobates catesbeianus* | -76.3794 | 36.6419 |
| *Lithobates catesbeianus* | -83.2241 | 31.38278 |
| *Lithobates catesbeianus* | -76.41 | 36.85 |
| *Lithobates catesbeianus* | -81.2504 | 36.761 |
| *Lithobates catesbeianus* | -77.192 | 35.5636 |
| *Lithobates catesbeianus* | -79.9063 | 34.8325 |
| *Lithobates catesbeianus* | -77.0897 | 35.0517 |
| *Lithobates catesbeianus* | -78.4936 | 35.243 |
| *Lithobates catesbeianus* | -78.2675 | 34.007 |
| *Lithobates catesbeianus* | -76.9332 | 35.107 |
| *Lithobates catesbeianus* | -78.0204 | 33.8929 |
| *Lithobates catesbeianus* | -77.7385 | 35.6858 |
| *Lithobates catesbeianus* | -81.0217 | 36.5456 |
| *Lithobates catesbeianus* | -79.0086 | 35.9892 |
| *Lithobates catesbeianus* | -82.3333 | 36.0131 |
| *Lithobates catesbeianus* | -82.836 | 35.8804 |
| *Lithobates catesbeianus* | -75.4731 | 39.468 |
| *Lithobates catesbeianus* | -75.6881 | 39.4625 |
| *Lithobates catesbeianus* | -76.4206 | 36.9003 |
| *Lithobates catesbeianus* | -77.3228 | 34.6257 |
| *Lithobates catesbeianus* | -77.4833 | 37.6592 |
| *Lithobates catesbeianus* | -75.9523 | 36.7107 |
| *Lithobates catesbeianus* | -76.3908 | 37.73 |
| *Lithobates catesbeianus* | -77.4264 | 37.6191 |
| *Lithobates catesbeianus* | -74.4735 | 40.7118 |
| *Lithobates catesbeianus* | -83.9936 | 39.8831 |
| *Lithobates catesbeianus* | -78.2167 | 45.2 |
| *Lithobates catesbeianus* | -78.2833 | 45.5 |
| *Lithobates catesbeianus* | -74.775 | 45.11 |
| *Lithobates catesbeianus* | -77.2883 | 45.9 |
| *Lithobates catesbeianus* | -79.7649 | 43.48132 |
| *Lithobates catesbeianus* | -92.7543 | 32.92397 |
| *Lithobates catesbeianus* | -105.239 | 40.16792 |
| *Lithobates catesbeianus* | -117.897 | 34.15855 |
| *Lithobates catesbeianus* | -121.32 | 45.72 |
| *Lithobates catesbeianus* | -73.5398 | 40.79191 |
| *Lithobates catesbeianus* | -116.303 | 43.6134 |
| *Lithobates catesbeianus* | -97.9411 | 29.8832 |
| *Lithobates catesbeianus* | -73.1238 | 40.9117 |
| *Lithobates catesbeianus* | -73.2367 | 43.4879 |
| *Lithobates catesbeianus* | -71.5508 | 41.98083 |
| *Lithobates catesbeianus* | -71.3455 | 42.2341 |
| *Lithobates catesbeianus* | -95.8901 | 31.92505 |
| *Lithobates catesbeianus* | -72.2763 | 44.30354 |
| *Lithobates catesbeianus* | -94.1169 | 32.68523 |
| *Lithobates catesbeianus* | -89.7527 | 38.77195 |
| *Lithobates catesbeianus* | -118.745 | 34.61561 |
| *Lithobates catesbeianus* | 126.516 | 36.61553 |
| *Lithobates catesbeianus* | -122.485 | 47.44724 |
| *Lithobates catesbeianus* | -96.4709 | 30.62818 |
| *Lithobates catesbeianus* | -122.045 | 47.71632 |
| *Lithobates catesbeianus* | 7.8314 | 44.9081 |
| *Lithobates catesbeianus* | -122.487 | 38.30204 |
| *Lithobates catesbeianus* | -90.1186 | 29.78658 |
| *Lithobates catesbeianus* | -96.8668 | 33.03615 |
| *Lithobates catesbeianus* | -100.719 | 43.3848 |
| *Lithobates catesbeianus* | -96.1716 | 30.7226 |
| *Lithobates catesbeianus* | -121.893 | 37.23665 |
| *Lithobates catesbeianus* | -96.092 | 30.74524 |
| *Lithobates catesbeianus* | -75.2168 | 39.97999 |
| *Lithobates catesbeianus* | -117.046 | 32.83782 |
| *Lithobates catesbeianus* | -117.042 | 32.83967 |
| *Lithobates catesbeianus* | -110.514 | 31.54249 |
| *Lithobates catesbeianus* | -94.7284 | 31.37261 |
| *Lithobates catesbeianus* | -78.8583 | 35.05417 |
| *Lithobates catesbeianus* | -117.906 | 33.915 |
| *Lithobates catesbeianus* | -76.5971 | 39.24367 |
| *Lithobates catesbeianus* | -71.6423 | 42.82201 |
| *Lithobates catesbeianus* | -97.6436 | 30.68346 |
| *Lithobates catesbeianus* | -94.6649 | 29.8219 |
| *Lithobates catesbeianus* | -104.934 | 39.77585 |
| *Lithobates catesbeianus* | -89.7305 | 41.33142 |
| *Lithobates catesbeianus* | -78.521 | 45.59028 |
| *Lithobates catesbeianus* | -98.18 | 33.9075 |
| *Lithobates catesbeianus* | -64.7878 | 46.08002 |
| *Lithobates catesbeianus* | -81.8867 | 41.41114 |
| *Lithobates catesbeianus* | -76.4199 | 44.32955 |
| *Lithobates catesbeianus* | -68.7847 | 44.99041 |
| *Lithobates catesbeianus* | -99.2162 | 28.27874 |
| *Lithobates catesbeianus* | -73.9696 | 40.65644 |
| *Lithobates catesbeianus* | -121.365 | 38.05662 |
| *Lithobates catesbeianus* | -74.8301 | 44.16752 |
| *Lithobates catesbeianus* | -122.224 | 37.3735 |
| *Lithobates catesbeianus* | -122.298 | 47.65385 |
| *Lithobates catesbeianus* | -118.41 | 34.11984 |
| *Lithobates catesbeianus* | -71.1849 | 42.4047 |
| *Lithobates catesbeianus* | -96.9972 | 32.61315 |
| *Lithobates catesbeianus* | -97.2806 | 28.2963 |
| *Lithobates catesbeianus* | -96.8058 | 28.30145 |
| *Lithobates catesbeianus* | -69.9912 | 41.88104 |
| *Lithobates catesbeianus* | -75.7918 | 45.37324 |
| *Lithobates catesbeianus* | -72.6069 | 43.99727 |
| *Lithobates catesbeianus* | -123.029 | 43.84147 |
| *Lithobates catesbeianus* | 126.44 | 36.74933 |
| *Lithobates catesbeianus* | -97.2223 | 32.66139 |
| *Lithobates catesbeianus* | -97.4864 | 35.63752 |
| *Lithobates catesbeianus* | -81.4271 | 35.39237 |
| *Lithobates catesbeianus* | -95.1611 | 29.8588 |
| *Lithobates catesbeianus* | -74.3117 | 40.0144 |
| *Lithobates catesbeianus* | -82.3229 | 29.65227 |
| *Lithobates catesbeianus* | -80.4545 | 32.7702 |
| *Lithobates catesbeianus* | -76.8738 | 42.09551 |
| *Lithobates catesbeianus* | -74.1494 | 44.32979 |
| *Lithobates catesbeianus* | -80.9863 | 32.91279 |
| *Lithobates catesbeianus* | -81.782 | 38.4595 |
| *Lithobates catesbeianus* | -76.3349 | 42.22884 |
| *Lithobates catesbeianus* | -74.0288 | 41.3098 |
| *Lithobates catesbeianus* | -78.7665 | 42.01068 |
| *Lithobates catesbeianus* | -67.1717 | 44.8719 |
| *Lithobates catesbeianus* | -72.53 | 41.9156 |
| *Lithobates catesbeianus* | -71.7966 | 43.69444 |
| *Lithobates catesbeianus* | -93.0557 | 37.26835 |
| *Lithobates catesbeianus* | -72.9911 | 41.3238 |
| *Lithobates catesbeianus* | -97.55 | 35.466 |
| *Lithobates catesbeianus* | -79.4655 | 34.7028 |
| *Lithobates catesbeianus* | -65.0782 | 45.62465 |
| *Lithobates catesbeianus* | 5.13146 | 51.14376 |
| *Lithobates catesbeianus* | 5.12761 | 51.14329 |
| *Lithobates catesbeianus* | 5.12887 | 51.14446 |
| *Lithobates catesbeianus* | -110.359 | 31.32256 |
| *Lithobates catesbeianus* | -81.5222 | 33.8254 |
| *Lithobates catesbeianus* | -80.3333 | 45.9 |
| *Lithobates catesbeianus* | -80.36 | 43.303 |
| *Lithobates catesbeianus* | -78.1111 | 45.64583 |
| *Lithobates catesbeianus* | -79.8949 | 46.37146 |
| *Lithobates catesbeianus* | -80.3483 | 47.295 |
| *Lithobates catesbeianus* | -79.4356 | 43.94957 |
| *Lithobates catesbeianus* | -75.931 | 45.6831 |
| *Lithobates catesbeianus* | -116.902 | 33.34005 |
| *Lithobates catesbeianus* | -122.591 | 38.59298 |
| *Lithobates catesbeianus* | -96.0238 | 30.73051 |
| *Lithobates catesbeianus* | 140.3617 | 35.98595 |
| *Lithobates catesbeianus* | -81.4501 | 32.2979 |
| *Lithobates catesbeianus* | -96.1819 | 36.74756 |
| *Lithobates catesbeianus* | -85.2547 | 42.1085 |
| *Lithobates catesbeianus* | -82.5798 | 42.5773 |
| *Lithobates catesbeianus* | -86.3876 | 43.3851 |
| *Lithobates catesbeianus* | -83.17 | 42.7125 |
| *Lithobates catesbeianus* | -83.3105 | 41.6839 |
| *Lithobates catesbeianus* | -77.2414 | 44.1 |
| *Lithobates catesbeianus* | -80.2665 | 43.1334 |
| *Lithobates catesbeianus* | -79.1869 | 43.2318 |
| *Lithobates catesbeianus* | -96.7248 | 39.66812 |
| *Lithobates catesbeianus* | -87.7922 | 34.7294 |
| *Lithobates catesbeianus* | -75.667 | 45.65 |
| *Lithobates catesbeianus* | -66.133 | 45.467 |
| *Lithobates catesbeianus* | -75.985 | 45.583 |
| *Lithobates catesbeianus* | -71.567 | 47.017 |
| *Lithobates catesbeianus* | -79.783 | 43.067 |
| *Lithobates catesbeianus* | -75.35 | 45.5 |
| *Lithobates catesbeianus* | -76.717 | 44.651 |
| *Lithobates catesbeianus* | -77.028 | 44.033 |
| *Lithobates catesbeianus* | -77.217 | 44.55 |
| *Lithobates catesbeianus* | -75.65 | 44.967 |
| *Lithobates catesbeianus* | -75.88 | 45.563 |
| *Lithobates catesbeianus* | -76.214 | 45.657 |
| *Lithobates catesbeianus* | -76.144 | 45.655 |
| *Lithobates catesbeianus* | -76.694 | 46.299 |
| *Lithobates catesbeianus* | -66.083 | 45.517 |
| *Lithobates catesbeianus* | -80.75 | 46.667 |
| *Lithobates catesbeianus* | -75.913 | 45.381 |
| *Lithobates catesbeianus* | -81.367 | 46.367 |
| *Lithobates catesbeianus* | -75.907 | 46.242 |
| *Lithobates catesbeianus* | -76.199 | 45.902 |
| *Lithobates catesbeianus* | -81.717 | 46.1 |
| *Lithobates catesbeianus* | -76.1 | 45.6 |
| *Lithobates catesbeianus* | -64.55 | 44.533 |
| *Lithobates catesbeianus* | -75.967 | 45.633 |
| *Lithobates catesbeianus* | -77.35 | 46 |
| *Lithobates catesbeianus* | -75.95 | 45.567 |
| *Lithobates catesbeianus* | -87.3399 | 38.04001 |
| *Lithobates catesbeianus* | -78.9396 | 39.13 |
| *Lithobates catesbeianus* | -82.5 | 35.42 |
| *Lithobates catesbeianus* | -79.83 | 40.54 |
| *Lithobates catesbeianus* | -78.63 | 35.78 |
| *Lithobates catesbeianus* | -76.79 | 39.04 |
| *Lithobates catesbeianus* | -80.9397 | 38.93 |
| *Lithobates catesbeianus* | -78.06 | 40.91 |
| *Lithobates catesbeianus* | -79.36 | 40.71 |
| *Lithobates catesbeianus* | -77.24 | 38.98 |
| *Lithobates catesbeianus* | -79.69 | 40.7 |
| *Lithobates catesbeianus* | -76.39 | 37.47 |
| *Lithobates catesbeianus* | -79.9568 | 32.85254 |
| *Lithobates catesbeianus* | -117.373 | 33.85349 |
| *Lithobates catesbeianus* | -118.699 | 34.05919 |
| *Lithobates catesbeianus* | -118.166 | 34.1453 |
| *Lithobates catesbeianus* | -114.469 | 32.88299 |
| *Lithobates catesbeianus* | -117.73 | 33.86893 |
| *Lithobates catesbeianus* | -117.624 | 33.71446 |
| *Lithobates catesbeianus* | -117.317 | 34.57001 |
| *Lithobates catesbeianus* | -118.475 | 34.03245 |
| *Lithobates catesbeianus* | -121.657 | 37.01215 |
| *Lithobates catesbeianus* | -117.694 | 33.62587 |
| *Lithobates catesbeianus* | -121.557 | 39.51359 |
| *Lithobates catesbeianus* | -121.607 | 39.77008 |
| *Lithobates catesbeianus* | -117.114 | 32.94317 |
| *Lithobates catesbeianus* | -94.2453 | 31.38729 |
| *Lithobates catesbeianus* | -73.9658 | 40.78222 |
| *Lithobates catesbeianus* | -98.6724 | 32.96903 |
| *Lithobates catesbeianus* | -100.269 | 35.91469 |
| *Lithobates catesbeianus* | -97.9275 | 29.86813 |
| *Lithobates catesbeianus* | -98.0996 | 32.51821 |
| *Lithobates catesbeianus* | -96.2276 | 29.94484 |
| *Lithobates catesbeianus* | -96.9601 | 29.90577 |
| *Lithobates catesbeianus* | -99.2768 | 32.75498 |
| *Lithobates catesbeianus* | -98.6731 | 33.79512 |
| *Lithobates catesbeianus* | -109.358 | 31.8648 |
| *Lithobates catesbeianus* | -122.01 | 45.6264 |
| *Lithobates catesbeianus* | -120.117 | 46.2833 |
| *Lithobates catesbeianus* | -112.388 | 34.5217 |
| *Lithobates catesbeianus* | -111.839 | 34.61096 |
| *Lithobates catesbeianus* | -118.602 | 34.19528 |
| *Lithobates catesbeianus* | -94.3115 | 38.91453 |
| *Lithobates catesbeianus* | -95.0006 | 38.57009 |
| *Lithobates catesbeianus* | -99.9023 | 37.85254 |
| *Lithobates catesbeianus* | -84.5085 | 39.59418 |
| *Lithobates catesbeianus* | -95.1145 | 39.27914 |
| *Lithobates catesbeianus* | -84.2669 | 39.2626 |
| *Lithobates catesbeianus* | -99.743 | 38.90635 |
| *Lithobates catesbeianus* | -100.918 | 38.6694 |
| *Lithobates catesbeianus* | -122.06 | 37.57495 |
| *Lithobates catesbeianus* | -121.252 | 37.46313 |
| *Lithobates catesbeianus* | -120.328 | 37.38505 |
| *Lithobates catesbeianus* | -120.32 | 38.00361 |
| *Lithobates catesbeianus* | -121.24 | 37.47324 |
| *Lithobates catesbeianus* | -121.319 | 37.1186 |
| *Lithobates catesbeianus* | -118.899 | 36.79678 |
| *Lithobates catesbeianus* | -116.383 | 35.0376 |
| *Lithobates catesbeianus* | -98.25 | 32.7729 |
| *Lithobates catesbeianus* | -89.6964 | 35.75995 |
| *Lithobates catesbeianus* | -84.3043 | 33.8748 |
| *Lithobates catesbeianus* | -78.5 | 34.5 |
| *Lithobates catesbeianus* | -82.2367 | 33.78627 |
| *Lithobates catesbeianus* | -81.8948 | 33.50054 |
| *Lithobates catesbeianus* | -78.32 | 39.33 |
| *Lithobates catesbeianus* | -80.4667 | 22.4667 |
| *Lithobates catesbeianus* | -77.25 | 38.93 |
| *Lithobates catesbeianus* | -68.7049 | 18.44878 |
| *Lithobates catesbeianus* | -92.381 | 32.88869 |
| *Lithobates catesbeianus* | -70.9347 | 41.6361 |
| *Lithobates catesbeianus* | -76.13 | 36.76 |
| *Lithobates catesbeianus* | -75.82 | 37.58 |
| *Lithobates catesbeianus* | -120.24 | 44.75114 |
| *Lithobates catesbeianus* | -117.273 | 40.96114 |
| *Lithobates catesbeianus* | -117.392 | 33.23768 |
| *Lithobates catesbeianus* | -73.6815 | 41.18103 |
| *Lithobates catesbeianus* | -76.9964 | 37.6614 |
| *Lithobates catesbeianus* | -84.0154 | 30.3585 |
| *Lithobates catesbeianus* | -84.9817 | 30.4317 |
| *Lithobates catesbeianus* | -81.8461 | 29.50843 |
| *Lithobates catesbeianus* | -86.42 | 30.79931 |
| *Lithobates catesbeianus* | -84.2811 | 30.47694 |
| *Lithobates catesbeianus* | -84.3592 | 30.5854 |
| *Lithobates catesbeianus* | -82.0021 | 29.6882 |
| *Lithobates catesbeianus* | -81.9888 | 29.72055 |
| *Lithobates catesbeianus* | -97.451 | 35.21497 |
| *Lithobates catesbeianus* | -94.7609 | 35.68269 |
| *Lithobates catesbeianus* | -98.2126 | 34.71047 |
| *Lithobates catesbeianus* | -97.3233 | 35.24667 |
| *Lithobates catesbeianus* | -97.1244 | 34.45379 |
| *Lithobates catesbeianus* | -97.5544 | 35.50046 |
| *Lithobates catesbeianus* | -94.9135 | 36.03669 |
| *Lithobates catesbeianus* | -94.7536 | 36.13828 |
| *Lithobates catesbeianus* | -102.85 | 36.92992 |
| *Lithobates catesbeianus* | -95.4456 | 35.1818 |
| *Lithobates catesbeianus* | -94.9204 | 35.28069 |
| *Lithobates catesbeianus* | -77.5023 | 34.4966 |
| *Lithobates catesbeianus* | -75.6415 | 35.9705 |
| *Lithobates catesbeianus* | -76.0643 | 35.9447 |
| *Lithobates catesbeianus* | -80.5004 | 33.8717 |
| *Lithobates catesbeianus* | -78.9871 | 35.9168 |
| *Lithobates catesbeianus* | -81.1402 | 35.3335 |
| *Lithobates catesbeianus* | -78.3879 | 34.2051 |
| *Lithobates catesbeianus* | -75.7566 | 39.3384 |
| *Lithobates catesbeianus* | -80.1725 | 34.8063 |
| *Lithobates catesbeianus* | -77.9881 | 34.3064 |
| *Lithobates catesbeianus* | -91.6357 | 35.9039 |
| *Lithobates catesbeianus* | -80.6922 | 33.1492 |
| *Lithobates catesbeianus* | -81.3489 | 34.9917 |
| *Lithobates catesbeianus* | -75.2734 | 39.4731 |
| *Lithobates catesbeianus* | -77.0603 | 37.5003 |
| *Lithobates catesbeianus* | -77.3226 | 34.6245 |
| *Lithobates catesbeianus* | -77.1075 | 37.4347 |
| *Lithobates catesbeianus* | -78.3625 | 34.1667 |
| *Lithobates catesbeianus* | -82.0829 | 36.7773 |
| *Lithobates catesbeianus* | -90.7649 | 38.11511 |
| *Lithobates catesbeianus* | -79.9278 | 42.82083 |
| *Lithobates catesbeianus* | -78.8442 | 42.03639 |
| *Lithobates catesbeianus* | -78.25 | 45.41667 |
| *Lithobates catesbeianus* | -79.8833 | 46.23333 |
| *Lithobates catesbeianus* | -122.782 | 38.612 |
| *Lithobates catesbeianus* | -91.4835 | 37.96799 |
| *Lithobates catesbeianus* | -108.079 | 33.35028 |
| *Lithobates catesbeianus* | -99.4547 | 38.7531 |
| *Lithobates catesbeianus* | -100.088 | 39.3048 |
| *Lithobates catesbeianus* | 140.721 | 36.7237 |
| *Lithobates catesbeianus* | -95.1953 | 37.34 |
| *Lithobates catesbeianus* | -86.4023 | 39.44295 |
| *Lithobates catesbeianus* | -83.1482 | 39.75431 |
| *Lithobates catesbeianus* | -84.3778 | 41.38277 |
| *Lithobates catesbeianus* | -83.2564 | 39.63219 |
| *Lithobates catesbeianus* | -83.8601 | 39.28057 |
| *Lithobates catesbeianus* | -84.3199 | 39.0076 |
| *Lithobates catesbeianus* | -82.8242 | 38.81648 |
| *Lithobates catesbeianus* | -116.19 | 32.61766 |
| *Lithobates catesbeianus* | -83.9876 | 41.0128 |
| *Lithobates catesbeianus* | -83.1947 | 39.12456 |
| *Lithobates catesbeianus* | -82.5306 | 38.95756 |
| *Lithobates catesbeianus* | -84.6329 | 41.24487 |
| *Lithobates catesbeianus* | -106.58 | 31.78444 |
| *Lithobates catesbeianus* | -106.921 | 32.48039 |
| *Lithobates catesbeianus* | -89.4978 | 34.34947 |
| *Lithobates catesbeianus* | -89.3913 | 34.4276 |
| *Lithobates catesbeianus* | -82.9725 | 39.6206 |
| *Lithobates catesbeianus* | -84.5827 | 37.7592 |
| *Lithobates catesbeianus* | -93.0083 | 35.6951 |
| *Lithobates catesbeianus* | -120.532 | 34.67891 |
| *Lithobates catesbeianus* | -72.2005 | 42.65009 |
| *Lithobates catesbeianus* | -71.5444 | 41.9875 |
| *Lithobates catesbeianus* | -71.6994 | 41.90125 |
| *Lithobates catesbeianus* | -71.5167 | 41.64917 |
| *Lithobates catesbeianus* | -71.5483 | 41.98361 |
| *Lithobates catesbeianus* | -73.9143 | 41.69483 |
| *Lithobates catesbeianus* | -104.929 | 40.42262 |
| *Lithobates catesbeianus* | -122.77 | 46.99 |
| *Lithobates catesbeianus* | -119.05 | 43.59 |
| *Lithobates catesbeianus* | -123.218 | 42.27965 |
| *Lithobates catesbeianus* | -78.0202 | 33.92152 |
| *Lithobates catesbeianus* | -118.404 | 36.30714 |
| *Lithobates catesbeianus* | -97.5342 | 30.24644 |
| *Lithobates catesbeianus* | -122.748 | 38.90091 |
| *Lithobates catesbeianus* | -79.5839 | 45.17096 |
| *Lithobates catesbeianus* | -112.897 | 27.29708 |
| *Lithobates catesbeianus* | -122.817 | 38.69193 |
| *Lithobates catesbeianus* | -106.799 | 34.41738 |
| *Lithobates catesbeianus* | -94.6888 | 38.8035 |
| *Lithobates catesbeianus* | -97.8093 | 30.1626 |
| *Lithobates catesbeianus* | -96.8225 | 33.07889 |
| *Lithobates catesbeianus* | -96.8548 | 33.13734 |
| *Lithobates catesbeianus* | -96.144 | 30.82214 |
| *Lithobates catesbeianus* | -71.8636 | 41.73145 |
| *Lithobates catesbeianus* | -118.41 | 34.12008 |
| *Lithobates catesbeianus* | -79.0339 | 35.8993 |
| *Lithobates catesbeianus* | 135.1022 | 34.66417 |
| *Lithobates catesbeianus* | -122.84 | 45.38336 |
| *Lithobates catesbeianus* | -96.8045 | 28.3059 |
| *Lithobates catesbeianus* | -95.5696 | 29.77158 |
| *Lithobates catesbeianus* | -97.1021 | 32.58738 |
| *Lithobates catesbeianus* | -122.176 | 37.36389 |
| *Lithobates catesbeianus* | -77.1055 | 38.75444 |
| *Lithobates catesbeianus* | 126.3457 | 36.73118 |
| *Lithobates catesbeianus* | -76.9416 | 38.91266 |
| *Lithobates catesbeianus* | -72.2685 | 41.8163 |
| *Lithobates catesbeianus* | -94.1574 | 36.06258 |
| *Lithobates catesbeianus* | -72.3182 | 44.29541 |
| *Lithobates catesbeianus* | -72.308 | 41.82002 |
| *Lithobates catesbeianus* | -82.8948 | 40.1015 |
| *Lithobates catesbeianus* | -96.0116 | 28.91922 |
| *Lithobates catesbeianus* | -116.167 | 46.4797 |
| *Lithobates catesbeianus* | -107.804 | 36.74519 |
| *Lithobates catesbeianus* | -87.9349 | 43.03439 |
| *Lithobates catesbeianus* | -123.631 | 39.75594 |
| *Lithobates catesbeianus* | -90.058 | 38.65388 |
| *Lithobates catesbeianus* | -94.7033 | 31.32986 |
| *Lithobates catesbeianus* | 11.65822 | 44.88892 |
| *Lithobates catesbeianus* | -75.3947 | 40.0206 |
| *Lithobates catesbeianus* | -94.0263 | 34.51038 |
| *Lithobates catesbeianus* | -75.8356 | 39.70125 |
| *Lithobates catesbeianus* | -83.1884 | 40.15558 |
| *Lithobates catesbeianus* | -95.4521 | 29.76477 |
| *Lithobates catesbeianus* | -122.513 | 38.35831 |
| *Lithobates catesbeianus* | -97.6441 | 30.68341 |
| *Lithobates catesbeianus* | -78.2014 | 37.18765 |
| *Lithobates catesbeianus* | -97.7592 | 30.46533 |
| *Lithobates catesbeianus* | -121.713 | 37.34271 |
| *Lithobates catesbeianus* | -77.0476 | 38.92949 |
| *Lithobates catesbeianus* | -82.0482 | 41.40031 |
| *Lithobates catesbeianus* | -98.1511 | 29.6228 |
| *Lithobates catesbeianus* | -77.1213 | 38.91909 |
| *Lithobates catesbeianus* | -97.4863 | 35.63745 |
| *Lithobates catesbeianus* | -105.142 | 40.16838 |
| *Lithobates catesbeianus* | -83.8473 | 42.30765 |
| *Lithobates catesbeianus* | -92.842 | 37.7201 |
| *Lithobates catesbeianus* | -74.1106 | 44.50766 |
| *Lithobates catesbeianus* | -92.211 | 35.9936 |
| *Lithobates catesbeianus* | -76.5095 | 42.4381 |
| *Lithobates catesbeianus* | -94.8239 | 35.70894 |
| *Lithobates catesbeianus* | -75.3333 | 4.53667 |
| *Lithobates catesbeianus* | -82.3525 | 29.6818 |
| *Lithobates catesbeianus* | -82.3733 | 29.6414 |
| *Lithobates catesbeianus* | -84.2985 | 30.36504 |
| *Lithobates catesbeianus* | -84.1721 | 30.4807 |
| *Lithobates catesbeianus* | -81.336 | 30.022 |
| *Lithobates catesbeianus* | -95.26 | 40.062 |
| *Lithobates catesbeianus* | -97.5728 | 32.49858 |
| *Lithobates catesbeianus* | -80.2977 | 36.874 |
| *Lithobates catesbeianus* | -67.8593 | 44.91978 |
| *Lithobates catesbeianus* | 4.7498 | 51.47226 |
| *Lithobates catesbeianus* | 5.12909 | 51.14475 |
| *Lithobates catesbeianus* | 5.13004 | 51.14517 |
| *Lithobates catesbeianus* | -97.3904 | 35.21153 |
| *Lithobates catesbeianus* | -73.1311 | 41.2528 |
| *Lithobates catesbeianus* | -97.834 | 27.51717 |
| *Lithobates catesbeianus* | -79.9358 | 33.0488 |
| *Lithobates catesbeianus* | -78.2682 | 34.6636 |
| *Lithobates catesbeianus* | -119.517 | 37.2447 |
| *Lithobates catesbeianus* | -74.68 | 45.05 |
| *Lithobates catesbeianus* | -77.8 | 45.73333 |
| *Lithobates catesbeianus* | -78.45 | 45.5666 |
| *Lithobates catesbeianus* | -80.0167 | 45.33333 |
| *Lithobates catesbeianus* | -116.948 | 33.0396 |
| *Lithobates catesbeianus* | -111.091 | 31.40067 |
| *Lithobates catesbeianus* | -117.266 | 34.23972 |
| *Lithobates catesbeianus* | -95.2154 | 39.02804 |
| *Lithobates catesbeianus* | -120.159 | 39.07947 |
| *Lithobates catesbeianus* | -122.773 | 38.01711 |
| *Lithobates catesbeianus* | -111.691 | 33.51578 |
| *Lithobates catesbeianus* | -122.483 | 38.30419 |
| *Lithobates catesbeianus* | -117.104 | 32.59224 |
| *Lithobates catesbeianus* | -121.913 | 37.30651 |
| *Lithobates catesbeianus* | -74.9273 | 38.95928 |
| *Lithobates catesbeianus* | -105.102 | 39.84071 |
| *Lithobates catesbeianus* | -114.289 | 34.15 |
| *Lithobates catesbeianus* | -65.8026 | 18.33595 |
| *Lithobates catesbeianus* | -117.524 | 33.58248 |
| *Lithobates catesbeianus* | -113.821 | 33.70836 |
| *Lithobates catesbeianus* | -124.426 | 42.81715 |
| *Lithobates catesbeianus* | -115.337 | 32.97501 |
| *Lithobates catesbeianus* | -96.0273 | 39.76663 |
| *Lithobates catesbeianus* | -99.231 | 38.06054 |
| *Lithobates catesbeianus* | -106.756 | 34.60013 |
| *Lithobates catesbeianus* | -95.9546 | 29.98183 |
| *Lithobates catesbeianus* | -102.953 | 29.17925 |
| *Lithobates catesbeianus* | -96.37 | 30.64583 |
| *Lithobates catesbeianus* | -95.3752 | 30.94514 |
| *Lithobates catesbeianus* | -96.4722 | 30.63972 |
| *Lithobates catesbeianus* | -97.2412 | 31.96274 |
| *Lithobates catesbeianus* | -96.9438 | 29.44679 |
| *Lithobates catesbeianus* | -101.014 | 35.97138 |
| *Lithobates catesbeianus* | -99.1219 | 29.73598 |
| *Lithobates catesbeianus* | -96.0825 | 31.97611 |
| *Lithobates catesbeianus* | -100.365 | 34.12056 |
| *Lithobates catesbeianus* | -119.958 | 36.86745 |
| *Lithobates catesbeianus* | -121.265 | 37.68843 |
| *Lithobates catesbeianus* | -122.445 | 38.6014 |
| *Lithobates catesbeianus* | -122.396 | 37.25027 |
| *Lithobates catesbeianus* | -123.793 | 39.48889 |
| *Lithobates catesbeianus* | -120.465 | 37.36303 |
| *Lithobates catesbeianus* | -122.772 | 38.38879 |
| *Lithobates catesbeianus* | -119.931 | 37.81241 |
| *Lithobates catesbeianus* | -114.217 | 33.66504 |
| *Lithobates catesbeianus* | -110.214 | 31.87518 |
| *Lithobates catesbeianus* | -121.871 | 37.34219 |
| *Lithobates catesbeianus* | -122.113 | 37.41994 |
| *Lithobates catesbeianus* | -119.134 | 36.8693 |
| *Lithobates catesbeianus* | -122.043 | 37.25355 |
| *Lithobates catesbeianus* | -121.556 | 36.94614 |
| *Lithobates catesbeianus* | -116.461 | 33.215 |
| *Lithobates catesbeianus* | -121.28 | 36.72111 |
| *Lithobates catesbeianus* | -116.137 | 43.56362 |
| *Lithobates catesbeianus* | -81.0519 | 41.53472 |
| *Lithobates catesbeianus* | -95.6472 | 37.0637 |
| *Lithobates catesbeianus* | -100.978 | 37.2459 |
| *Lithobates catesbeianus* | -95.0727 | 37.7845 |
| *Lithobates catesbeianus* | -97.1278 | 39.92991 |
| *Lithobates catesbeianus* | -115.233 | 37.533 |
| *Lithobates catesbeianus* | -82.3504 | 29.6451 |
| *Lithobates catesbeianus* | -81.6434 | 29.24637 |
| *Lithobates catesbeianus* | -82.38 | 29.57 |
| *Lithobates catesbeianus* | -70.66 | 41.95 |
| *Lithobates catesbeianus* | -84.84 | 34.78 |
| *Lithobates catesbeianus* | -75.42 | 38.76 |
| *Lithobates catesbeianus* | -81.16 | 32.14 |
| *Lithobates catesbeianus* | -75.74 | 38.37 |
| *Lithobates catesbeianus* | -94.6396 | 37.03473 |
| *Lithobates catesbeianus* | -99.1508 | 38.89261 |
| *Lithobates catesbeianus* | -74.76 | 40.15 |
| *Lithobates catesbeianus* | -75.01 | 41.07 |
| *Lithobates catesbeianus* | -78.85 | 38.62 |
| *Lithobates catesbeianus* | -77.83 | 36.67 |
| *Lithobates catesbeianus* | -76.1 | 36.64 |
| *Lithobates catesbeianus* | -98.1 | 39.8 |
| *Lithobates catesbeianus* | -82.8054 | 41.68945 |
| *Lithobates catesbeianus* | -81.6845 | 40.06044 |
| *Lithobates catesbeianus* | -84.3833 | 39.79833 |
| *Lithobates catesbeianus* | -83.1744 | 40.07727 |
| *Lithobates catesbeianus* | -81.6696 | 40.09664 |
| *Lithobates catesbeianus* | -84.0152 | 38.85178 |
| *Lithobates catesbeianus* | -84.5664 | 39.8146 |
| *Lithobates catesbeianus* | -81.8844 | 39.36114 |
| *Lithobates catesbeianus* | -121.91 | 39.01271 |
| *Lithobates catesbeianus* | -87.2002 | 41.6109 |
| *Lithobates catesbeianus* | -74.3819 | 45.1748 |
| *Lithobates catesbeianus* | -75.9163 | 45.0834 |
| *Lithobates catesbeianus* | -77.3473 | 44.1087 |
| *Lithobates catesbeianus* | -79.1446 | 42.8749 |
| *Lithobates catesbeianus* | -81.0356 | 44.7153 |
| *Lithobates catesbeianus* | -74.7128 | 45.0389 |
| *Lithobates catesbeianus* | -79.2855 | 43.1852 |
| *Lithobates catesbeianus* | -78.6799 | 43.8871 |
| *Lithobates catesbeianus* | -92.4102 | 45.9921 |
| *Lithobates catesbeianus* | -119.812 | 34.42444 |
| *Lithobates catesbeianus* | -117.093 | 32.8984 |
| *Lithobates catesbeianus* | -117.033 | 32.84655 |
| *Lithobates catesbeianus* | -2.22354 | 53.0018 |
| *Lithobates catesbeianus* | -122.236 | 48.5039 |
| *Lithobates catesbeianus* | -122.255 | 47.8743 |
| *Lithobates catesbeianus* | -98.2803 | 39.69664 |
| *Lithobates catesbeianus* | -98.5874 | 34.77137 |
| *Lithobates catesbeianus* | -95.8446 | 34.50019 |
| *Lithobates catesbeianus* | -94.5402 | 33.86671 |
| *Lithobates catesbeianus* | -95.2607 | 36.2977 |
| *Lithobates catesbeianus* | -98.6326 | 34.65257 |
| *Lithobates catesbeianus* | -94.7149 | 34.03111 |
| *Lithobates catesbeianus* | -95.5122 | 35.467 |
| *Lithobates catesbeianus* | -95.2355 | 35.1551 |
| *Lithobates catesbeianus* | -97.0995 | 35.26117 |
| *Lithobates catesbeianus* | -102.976 | 36.97342 |
| *Lithobates catesbeianus* | -102.957 | 36.92942 |
| *Lithobates catesbeianus* | -97.9683 | 35.55855 |
| *Lithobates catesbeianus* | -95.8906 | 35.9496 |
| *Lithobates catesbeianus* | -98.0504 | 36.70453 |
| *Lithobates catesbeianus* | -95.1835 | 35.67281 |
| *Lithobates catesbeianus* | -102.968 | 36.9659 |
| *Lithobates catesbeianus* | -82.1355 | 35.7378 |
| *Lithobates catesbeianus* | -80.5438 | 35.0038 |
| *Lithobates catesbeianus* | -79.0505 | 35.7669 |
| *Lithobates catesbeianus* | -79.0408 | 35.7501 |
| *Lithobates catesbeianus* | -78.4875 | 35.6487 |
| *Lithobates catesbeianus* | -79.009 | 35.9886 |
| *Lithobates catesbeianus* | -78.6179 | 36.5363 |
| *Lithobates catesbeianus* | -79.1812 | 34.8856 |
| *Lithobates catesbeianus* | -79.3315 | 34.7725 |
| *Lithobates catesbeianus* | -75.3595 | 38.6684 |
| *Lithobates catesbeianus* | -78.6386 | 35.6763 |
| *Lithobates catesbeianus* | -74.8129 | 39.0995 |
| *Lithobates catesbeianus* | -75.9641 | 36.5175 |
| *Lithobates catesbeianus* | -74.5297 | 39.4969 |
| *Lithobates catesbeianus* | -79.8224 | 36.8963 |
| *Lithobates catesbeianus* | -77.1312 | 37.2703 |
| *Lithobates catesbeianus* | -74.4732 | 40.712 |
| *Lithobates catesbeianus* | -78.614 | 34.7723 |
| *Lithobates catesbeianus* | -106.594 | 31.84406 |
| *Lithobates catesbeianus* | -106.796 | 32.23611 |
| *Lithobates catesbeianus* | -98.5085 | 30.01628 |
| *Lithobates catesbeianus* | -106.227 | 31.5883 |
| *Lithobates catesbeianus* | -97.9351 | 22.39549 |
| *Lithobates catesbeianus* | -118.438 | 33.35328 |
| *Lithobates catesbeianus* | -83.8449 | 38.74563 |
| *Lithobates catesbeianus* | -90.5853 | 41.75614 |
| *Lithobates catesbeianus* | -81.3265 | 40.2825 |
| *Lithobates catesbeianus* | -82.0028 | 39.6028 |
| *Lithobates catesbeianus* | -82.2928 | 40.1656 |
| *Lithobates catesbeianus* | -89.7504 | 32.7504 |
| *Lithobates catesbeianus* | -77.4006 | 44.69611 |
| *Lithobates catesbeianus* | -81.8392 | 42.34129 |
| *Lithobates catesbeianus* | -82.3648 | 30.82192 |
| *Lithobates catesbeianus* | -88.2866 | 41.93972 |
| *Lithobates catesbeianus* | -120.556 | 34.67678 |
| *Lithobates catesbeianus* | -120.556 | 34.6768 |
| *Lithobates catesbeianus* | -76.5266 | 36.66732 |
| *Lithobates catesbeianus* | -157.977 | 21.3957 |
| *Lithobates catesbeianus* | -155.079 | 19.5908 |
| *Lithobates catesbeianus* | -77.119 | 36.75 |
| *Lithobates catesbeianus* | -82.1028 | 41.39721 |
| *Lithobates catesbeianus* | -95.5669 | 34.02641 |
| *Lithobates catesbeianus* | -77.1816 | 39.55986 |
| *Lithobates catesbeianus* | -78.2271 | 43.00553 |
| *Lithobates catesbeianus* | -78.4441 | 38.5158 |
| *Lithobates catesbeianus* | -83.8982 | 31.9479 |
| *Lithobates catesbeianus* | -83.1908 | 35.05398 |
| *Lithobates catesbeianus* | -85.2664 | 34.8833 |
| *Lithobates catesbeianus* | -78.4548 | 34.71217 |
| *Lithobates catesbeianus* | -79.7908 | 33.14435 |
| *Lithobates catesbeianus* | -76.7793 | 36.8664 |
| *Lithobates catesbeianus* | -77.24 | 38.91 |
| *Lithobates catesbeianus* | -78.27 | 37.42 |
| *Lithobates catesbeianus* | -82.9424 | 36.7965 |
| *Lithobates catesbeianus* | -82.9223 | 36.802 |
| *Lithobates catesbeianus* | -119.705 | 39.14292 |
| *Lithobates catesbeianus* | -76.9781 | 37.6492 |
| *Lithobates catesbeianus* | -75.6553 | 5.90278 |
| *Lithobates catesbeianus* | -75.6167 | 5.72667 |
| *Lithobates catesbeianus* | 0 | 45.917 |
| *Lithobates catesbeianus* | -83.217 | 46.75 |
| *Lithobates catesbeianus* | -76.138 | 45.658 |
| *Lithobates catesbeianus* | -75.807 | 45.468 |
| *Lithobates catesbeianus* | -76.044 | 45.602 |
| *Lithobates catesbeianus* | -76.083 | 45.597 |
| *Lithobates catesbeianus* | -75.814 | 45.468 |
| *Lithobates catesbeianus* | -64.583 | 45.067 |
| *Lithobates catesbeianus* | -76.283 | 45.3 |
| *Lithobates catesbeianus* | -64.283 | 44.833 |
| *Lithobates catesbeianus* | -76.409 | 46.319 |
| *Lithobates catesbeianus* | -75.843 | 46.18 |
| *Lithobates catesbeianus* | -77.55 | 45.867 |
| *Lithobates catesbeianus* | -76.904 | 44.741 |
| *Lithobates catesbeianus* | -75.867 | 45.509 |
| *Lithobates catesbeianus* | -81.283 | 45.233 |
| *Lithobates catesbeianus* | -64.667 | 44.833 |
| *Lithobates catesbeianus* | -95.888 | 31.9292 |
| *Lithobates catesbeianus* | -118.427 | 33.96675 |
| *Lithobates catesbeianus* | -97.2806 | 32.88388 |
| *Lithobates catesbeianus* | -70.5307 | 44.42413 |
| *Lithobates catesbeianus* | -76.5635 | 42.50693 |
| *Lithobates catesbeianus* | -80.2025 | 40.80364 |
| *Lithobates catesbeianus* | -72.3465 | 43.81609 |
| *Lithobates catesbeianus* | -79.0289 | 35.96236 |
| *Lithobates catesbeianus* | -118.911 | 46.05763 |
| *Lithobates catesbeianus* | -71.64 | 41.58417 |
| *Lithobates catesbeianus* | -71.4136 | 41.68778 |
| *Lithobates catesbeianus* | -84.4018 | 33.82639 |
| *Lithobates catesbeianus* | -92.1018 | 38.58193 |
| *Lithobates catesbeianus* | -123.515 | 42.91594 |
| *Lithobates catesbeianus* | -122.283 | 37.92904 |
| *Lithobates catesbeianus* | -122.651 | 49.09971 |
| *Lithobates catesbeianus* | -95.2088 | 38.98378 |
| *Lithobates catesbeianus* | -119.579 | 37.74615 |
| *Lithobates catesbeianus* | -114.667 | 36.85175 |
| *Lithobates catesbeianus* | -99.2757 | 33.55249 |
| *Lithobates catesbeianus* | -94.7032 | 31.32982 |
| *Lithobates catesbeianus* | -97.1168 | 32.79278 |
| *Lithobates catesbeianus* | -71.5471 | 41.86813 |
| *Lithobates catesbeianus* | -81.4487 | 41.07256 |
| *Lithobates catesbeianus* | -76.3239 | 44.56699 |
| *Lithobates catesbeianus* | -122.489 | 47.44707 |
| *Lithobates catesbeianus* | -75.4987 | 45.50107 |
| *Lithobates catesbeianus* | -95.2688 | 29.47168 |
| *Lithobates catesbeianus* | 126.3676 | 36.81871 |
| *Lithobates catesbeianus* | -96.3632 | 30.69476 |
| *Lithobates catesbeianus* | -75.4902 | 39.27103 |
| *Lithobates catesbeianus* | -71.1505 | 42.38906 |
| *Lithobates catesbeianus* | -87.9053 | 41.64681 |
| *Lithobates catesbeianus* | -84.6145 | 33.38301 |
| *Lithobates catesbeianus* | -88.1726 | 41.86288 |
| *Lithobates catesbeianus* | -77.7525 | 45.86623 |
| *Lithobates catesbeianus* | -79.5436 | 37.62702 |
| *Lithobates catesbeianus* | -77.1047 | 38.75414 |
| *Lithobates catesbeianus* | -75.6709 | 39.87262 |
| *Lithobates catesbeianus* | -121.93 | 37.21548 |
| *Lithobates catesbeianus* | -96.3351 | 30.59159 |
| *Lithobates catesbeianus* | -102.993 | 21.14121 |
| *Lithobates catesbeianus* | -97.8091 | 30.23957 |
| *Lithobates catesbeianus* | -122.494 | 38.37361 |
| *Lithobates catesbeianus* | -122.783 | 38.61373 |
| *Lithobates catesbeianus* | -97.2829 | 32.88671 |
| *Lithobates catesbeianus* | -121.917 | 36.91198 |
| *Lithobates catesbeianus* | -105.171 | 40.10387 |
| *Lithobates catesbeianus* | -93.7373 | 30.14003 |
| *Lithobates catesbeianus* | -75.6684 | 35.98988 |
| *Lithobates catesbeianus* | -122.293 | 47.65584 |
| *Lithobates catesbeianus* | -71.1507 | 42.38884 |
| *Lithobates catesbeianus* | -75.5025 | 45.49878 |
| *Lithobates catesbeianus* | -94.6937 | 38.79599 |
| *Lithobates catesbeianus* | -71.3394 | 42.25328 |
| *Lithobates catesbeianus* | -71.1471 | 42.38712 |
| *Lithobates catesbeianus* | -68.2465 | 44.39655 |
| *Lithobates catesbeianus* | -75.4852 | 45.49955 |
| *Lithobates catesbeianus* | -109.003 | 31.92704 |
| *Lithobates catesbeianus* | -76.4763 | 42.45127 |
| *Lithobates catesbeianus* | -76.5049 | 42.45997 |
| *Lithobates catesbeianus* | -79.3369 | 38.6369 |
| *Lithobates catesbeianus* | -97.4268 | 35.2437 |
| *Lithobates catesbeianus* | -82.7182 | 29.00122 |
| *Lithobates catesbeianus* | -84.4392 | 30.1978 |
| *Lithobates catesbeianus* | -85.2372 | 30.68771 |
| *Lithobates catesbeianus* | -83.2347 | 29.96235 |
| *Lithobates catesbeianus* | -84.2795 | 30.21284 |
| *Lithobates catesbeianus* | -76.4693 | 42.45056 |
| *Lithobates catesbeianus* | -94.5555 | 29.60715 |
| *Lithobates catesbeianus* | -72.6307 | 41.33665 |
| *Lithobates catesbeianus* | -72.1232 | 41.351 |
| *Lithobates catesbeianus* | -74.8333 | 3.75 |
| *Lithobates catesbeianus* | -97.8531 | 29.99678 |
| *Lithobates catesbeianus* | 5.12883 | 51.1445 |
| *Lithobates catesbeianus* | 5.12872 | 51.14413 |
| *Lithobates catesbeianus* | 5.12879 | 51.14382 |
| *Lithobates catesbeianus* | -76.3823 | 3.83582 |
| *Lithobates catesbeianus* | -80.9949 | 43.05867 |
| *Lithobates catesbeianus* | -79.4504 | 43.86325 |
| *Lithobates catesbeianus* | 0.55821 | 47.14953 |
| *Lithobates catesbeianus* | -94.3881 | 36.0906 |
| *Lithobates catesbeianus* | 2.1 | 41.41 |
| *Lithobates catesbeianus* | -120.455 | 37.35578 |
| *Lithobates catesbeianus* | -119.503 | 37.14737 |
| *Lithobates catesbeianus* | -122.182 | 38.82916 |
| *Lithobates catesbeianus* | -120.413 | 35.33861 |
| *Lithobates catesbeianus* | -123.1 | 43.79762 |
| *Lithobates catesbeianus* | -121.902 | 37.40957 |
| *Lithobates catesbeianus* | -121.769 | 36.23022 |
| *Lithobates catesbeianus* | -76.7319 | 39.2721 |
| *Lithobates catesbeianus* | -157.954 | 21.4666 |
| *Lithobates catesbeianus* | -115.891 | 36.15441 |
| *Lithobates catesbeianus* | -98.4908 | 29.29444 |
| *Lithobates catesbeianus* | -97.215 | 29.23306 |
| *Lithobates catesbeianus* | -73.3694 | 41.84111 |
| *Lithobates catesbeianus* | -73.0242 | 41.77583 |
| *Lithobates catesbeianus* | -97.8384 | 32.29358 |
| *Lithobates catesbeianus* | -95.239 | 33.27342 |
| *Lithobates catesbeianus* | -98.695 | 33.72915 |
| *Lithobates catesbeianus* | -98.5028 | 30.27194 |
| *Lithobates catesbeianus* | -119.309 | 34.2825 |
| *Lithobates catesbeianus* | -95.2743 | 38.95692 |
| *Lithobates catesbeianus* | -96.2896 | 38.87665 |
| *Lithobates catesbeianus* | -96.082 | 37.37806 |
| *Lithobates catesbeianus* | -94.7924 | 37.12788 |
| *Lithobates catesbeianus* | -95.2345 | 38.97137 |
| *Lithobates catesbeianus* | -71.4549 | 42.29495 |
| *Lithobates catesbeianus* | -94.7954 | 38.98831 |
| *Lithobates catesbeianus* | -100.375 | 37.25821 |
| *Lithobates catesbeianus* | -97.947 | 39.0869 |
| *Lithobates catesbeianus* | -98.281 | 39.69746 |
| *Lithobates catesbeianus* | -94.6984 | 37.47584 |
| *Lithobates catesbeianus* | -95.5148 | 39.67136 |
| *Lithobates catesbeianus* | -82.5578 | 39.6514 |
| *Lithobates catesbeianus* | -90.0004 | 44.5002 |
| *Lithobates catesbeianus* | -85.8147 | 33.4821 |
| *Lithobates catesbeianus* | -87.2192 | 41.6128 |
| *Lithobates catesbeianus* | -86.3246 | 43.4252 |
| *Lithobates catesbeianus* | -80.6058 | 43.1474 |
| *Lithobates catesbeianus* | -80.1 | 42.55 |
| *Lithobates catesbeianus* | -76.683 | 44.767 |
| *Lithobates catesbeianus* | -76.15 | 45.05 |
| *Lithobates catesbeianus* | -75.833 | 45.633 |
| *Lithobates catesbeianus* | -75.783 | 45.633 |
| *Lithobates catesbeianus* | -77.119 | 44.104 |
| *Lithobates catesbeianus* | -77.372 | 46.031 |
| *Lithobates catesbeianus* | -75.359 | 45.521 |
| *Lithobates catesbeianus* | -76.025 | 45.604 |
| *Lithobates catesbeianus* | -75.817 | 45.35 |
| *Lithobates catesbeianus* | -75.683 | 44.967 |
| *Lithobates catesbeianus* | -75.835 | 45.054 |
| *Lithobates catesbeianus* | -81.224 | 44.846 |
| *Lithobates catesbeianus* | -75.656 | 44.882 |
| *Lithobates catesbeianus* | -76.667 | 45.563 |
| *Lithobates catesbeianus* | -81.543 | 46.677 |
| *Lithobates catesbeianus* | -77.138 | 44.835 |
| *Lithobates catesbeianus* | -76.763 | 45.939 |
| *Lithobates catesbeianus* | -76.85 | 45 |
| *Lithobates catesbeianus* | -77.233 | 44.95 |
| *Lithobates catesbeianus* | -83.261 | 39.10025 |
| *Lithobates catesbeianus* | -83.6238 | 40.1195 |
| *Lithobates catesbeianus* | -81.7425 | 40.03961 |
| *Lithobates catesbeianus* | -84.0189 | 39.56382 |
| *Lithobates catesbeianus* | -83.9702 | 39.54673 |
| *Lithobates catesbeianus* | -83.2578 | 39.71552 |
| *Lithobates catesbeianus* | -85.7919 | 40.4544 |
| *Lithobates catesbeianus* | -83.8799 | 40.89955 |
| *Lithobates catesbeianus* | -84.575 | 41.66623 |
| *Lithobates catesbeianus* | -117.09 | 33.05442 |
| *Lithobates catesbeianus* | -117.218 | 32.94024 |
| *Lithobates catesbeianus* | -122.606 | 46.9131 |
| *Lithobates catesbeianus* | -78.69 | 41.41 |
| *Lithobates catesbeianus* | -78.06 | 40.86 |
| *Lithobates catesbeianus* | -76.52 | 38.64 |
| *Lithobates catesbeianus* | -76.56 | 36.64 |
| *Lithobates catesbeianus* | -77.11 | 40 |
| *Lithobates catesbeianus* | -82.1456 | 27.01285 |
| *Lithobates catesbeianus* | -118.026 | 34.03244 |
| *Lithobates catesbeianus* | -118.068 | 34.02106 |
| *Lithobates catesbeianus* | -120.503 | 34.71427 |
| *Lithobates catesbeianus* | -94.5783 | 39.09972 |
| *Lithobates catesbeianus* | -99.1286 | 37.118 |
| *Lithobates catesbeianus* | -95.0646 | 39.81277 |
| *Lithobates catesbeianus* | -94.6943 | 37.05672 |
| *Lithobates catesbeianus* | -120.006 | 39.09596 |
| *Lithobates catesbeianus* | -117.902 | 35.94772 |
| *Lithobates catesbeianus* | -108.078 | 33.3487 |
| *Lithobates catesbeianus* | -157.956 | 21.69 |
| *Lithobates catesbeianus* | -118.954 | 46.1972 |
| *Lithobates catesbeianus* | -119.6 | 45.9 |
| *Lithobates catesbeianus* | -114.474 | 32.9797 |
| *Lithobates catesbeianus* | -111.489 | 32.85111 |
| *Lithobates catesbeianus* | -111.233 | 34.7939 |
| *Lithobates catesbeianus* | -106.51 | 31.76174 |
| *Lithobates catesbeianus* | -105.267 | 40.16039 |
| *Lithobates catesbeianus* | -104.798 | 39.81228 |
| *Lithobates catesbeianus* | -155.087 | 19.7004 |
| *Lithobates catesbeianus* | -75.12 | 40.96 |
| *Lithobates catesbeianus* | -75.22 | 41.06 |
| *Lithobates catesbeianus* | -80.1 | 40.95 |
| *Lithobates catesbeianus* | -84.34 | 32.89 |
| *Lithobates catesbeianus* | -92.8078 | 34.8866 |
| *Lithobates catesbeianus* | -98.8711 | 39.24951 |
| *Lithobates catesbeianus* | -88.0395 | 36.89706 |
| *Lithobates catesbeianus* | -120.548 | 34.679 |
| *Lithobates catesbeianus* | -120.54 | 34.68066 |
| *Lithobates catesbeianus* | -70.6736 | 41.5264 |
| *Lithobates catesbeianus* | -116.31 | 36.42916 |
| *Lithobates catesbeianus* | -83.3848 | 34.9633 |
| *Lithobates catesbeianus* | -89.4444 | 36.38016 |
| *Lithobates catesbeianus* | -75.6194 | 38.344 |
| *Lithobates catesbeianus* | -68.7238 | 18.51654 |
| *Lithobates catesbeianus* | -76.7504 | 39.23074 |
| *Lithobates catesbeianus* | -78.554 | 38.2413 |
| *Lithobates catesbeianus* | -79.1603 | 36.853 |
| *Lithobates catesbeianus* | -82.6666 | 36.8928 |
| *Lithobates catesbeianus* | -79.73 | 37.85 |
| *Lithobates catesbeianus* | -76.8523 | 36.8085 |
| *Lithobates catesbeianus* | -98.9226 | 36.57924 |
| *Lithobates catesbeianus* | -98.9262 | 36.58963 |
| *Lithobates catesbeianus* | -95.3331 | 34.6595 |
| *Lithobates catesbeianus* | -95.4915 | 34.90339 |
| *Lithobates catesbeianus* | -98.2295 | 34.63748 |
| *Lithobates catesbeianus* | -98.3173 | 34.59478 |
| *Lithobates catesbeianus* | -97.492 | 35.16967 |
| *Lithobates catesbeianus* | -97.9584 | 34.50768 |
| *Lithobates catesbeianus* | -98.3846 | 35.56407 |
| *Lithobates catesbeianus* | -98.2621 | 35.07246 |
| *Lithobates catesbeianus* | -96.2564 | 33.99746 |
| *Lithobates catesbeianus* | -94.7734 | 36.87827 |
| *Lithobates catesbeianus* | -94.7303 | 36.54568 |
| *Lithobates catesbeianus* | -102.992 | 36.90082 |
| *Lithobates catesbeianus* | -94.6943 | 33.69151 |
| *Lithobates catesbeianus* | -94.6422 | 33.7487 |
| *Lithobates catesbeianus* | -76.4569 | 35.7726 |
| *Lithobates catesbeianus* | -116.194 | 43.6106 |
| *Lithobates catesbeianus* | -78.6581 | 33.991 |
| *Lithobates catesbeianus* | -75.5341 | 39.3297 |
| *Lithobates catesbeianus* | -78.2443 | 34.852 |
| *Lithobates catesbeianus* | -81.8051 | 36.2347 |
| *Lithobates catesbeianus* | -75.8515 | 42.3854 |
| *Lithobates catesbeianus* | -77.3147 | 37.4908 |
| *Lithobates catesbeianus* | -77.4739 | 37.5928 |
| *Lithobates catesbeianus* | -77.7972 | 37.6368 |
| *Lithobates catesbeianus* | -76.8933 | 37.4302 |
| *Lithobates catesbeianus* | -81.8195 | 35.1411 |
| *Lithobates catesbeianus* | -77.6524 | 35.8624 |
| *Lithobates catesbeianus* | -78.7167 | 45.58333 |
| *Lithobates catesbeianus* | -123.098 | 38.52859 |
| *Lithobates catesbeianus* | -81.3 | 43.88333 |
| *Lithobates catesbeianus* | -106.655 | 31.89448 |
| *Lithobates catesbeianus* | -82.7457 | 29.8521 |
| *Lithobates catesbeianus* | -89.1732 | 34.28976 |
| *Lithobates catesbeianus* | -94.7388 | 32.90593 |
| *Lithobates catesbeianus* | -90.1858 | 38.15683 |
| *Lithobates catesbeianus* | -122.067 | 37.19757 |
| *Lithobates catesbeianus* | -73.3539 | 43.71701 |
| *Lithobates catesbeianus* | -116.328 | 43.65153 |
| *Lithobates catesbeianus* | -122.98 | 47.44 |
| *Lithobates catesbeianus* | -119.03 | 36.0686 |
| *Lithobates catesbeianus* | -119.722 | 37.09052 |
| *Lithobates catesbeianus* | -117.415 | 33.89375 |
| *Lithobates catesbeianus* | -122.036 | 40.10757 |
| *Lithobates catesbeianus* | -114.667 | 36.88075 |
| *Lithobates catesbeianus* | -122.163 | 47.75412 |
| *Lithobates catesbeianus* | -122.707 | 38.06134 |
| *Lithobates catesbeianus* | -93.6256 | 32.34764 |
| *Lithobates catesbeianus* | -71.2755 | 42.21011 |
| *Lithobates catesbeianus* | -77.7567 | 45.86794 |
| *Lithobates catesbeianus* | -108.594 | 32.84521 |
| *Lithobates catesbeianus* | -70.4981 | 43.951 |
| *Lithobates catesbeianus* | -121.731 | 37.20192 |
| *Lithobates catesbeianus* | -122.785 | 38.61205 |
| *Lithobates catesbeianus* | -69.9958 | 41.88152 |
| *Lithobates catesbeianus* | -120.667 | 35.30931 |
| *Lithobates catesbeianus* | -94.3912 | 29.57322 |
| *Lithobates catesbeianus* | -102.123 | 31.96676 |
| *Lithobates catesbeianus* | -120.054 | 38.93571 |
| *Lithobates catesbeianus* | -122.508 | 38.36747 |
| *Lithobates catesbeianus* | -71.3304 | 42.41706 |
| *Lithobates catesbeianus* | -117.936 | 33.90806 |
| *Lithobates catesbeianus* | -65.949 | 43.89621 |
| *Lithobates catesbeianus* | -88.3212 | 41.82068 |
| *Lithobates catesbeianus* | -121.672 | 38.55676 |
| *Lithobates catesbeianus* | -122.283 | 37.57266 |
| *Lithobates catesbeianus* | -77.1042 | 38.75474 |
| *Lithobates catesbeianus* | -91.0269 | 30.4267 |
| *Lithobates catesbeianus* | -123.948 | 46.15023 |
| *Lithobates catesbeianus* | -96.8484 | 33.05121 |
| *Lithobates catesbeianus* | -88.9517 | 40.38264 |
| *Lithobates catesbeianus* | -117.217 | 32.8413 |
| *Lithobates catesbeianus* | -122.312 | 47.6417 |
| *Lithobates catesbeianus* | -122.4 | 37.26155 |
| *Lithobates catesbeianus* | -116.384 | 35.0372 |
| *Lithobates catesbeianus* | -105.088 | 39.49483 |
| *Lithobates catesbeianus* | -76.8149 | 44.95877 |
| *Lithobates catesbeianus* | -99.2615 | 33.59857 |
| *Lithobates catesbeianus* | -117.217 | 32.8413 |
| *Lithobates catesbeianus* | -70.6465 | 41.8871 |
| *Lithobates catesbeianus* | -71.7825 | 42.21009 |
| *Lithobates catesbeianus* | -75.4969 | 45.50224 |
| *Lithobates catesbeianus* | -121.739 | 37.25817 |
| *Lithobates catesbeianus* | -75.4847 | 45.49944 |
| *Lithobates catesbeianus* | -96.8052 | 28.30459 |
| *Lithobates catesbeianus* | -97.2827 | 30.10523 |
| *Lithobates catesbeianus* | 126.4223 | 36.71027 |
| *Lithobates catesbeianus* | -97.9059 | 29.65357 |
| *Lithobates catesbeianus* | -79.8384 | 36.08973 |
| *Lithobates catesbeianus* | -95.4129 | 30.10827 |
| *Lithobates catesbeianus* | -74.4549 | 40.40906 |
| *Lithobates catesbeianus* | -97.7401 | 30.1683 |
| *Lithobates catesbeianus* | -89.8697 | 30.05738 |
| *Lithobates catesbeianus* | -78.1486 | 38.89942 |
| *Lithobates catesbeianus* | -74.2954 | 39.94685 |
| *Lithobates catesbeianus* | -88.9061 | 37.23483 |
| *Lithobates catesbeianus* | -96.2368 | 29.63689 |
| *Lithobates catesbeianus* | -72.7737 | 41.54648 |
| *Lithobates catesbeianus* | -82.6085 | 35.49766 |
| *Lithobates catesbeianus* | -118.526 | 34.24094 |
| *Lithobates catesbeianus* | -96.9539 | 33.06687 |
| *Lithobates catesbeianus* | -121.701 | 37.22478 |
| *Lithobates catesbeianus* | -75.8232 | 45.29328 |
| *Lithobates catesbeianus* | -96.5481 | 33.33772 |
| *Lithobates catesbeianus* | -75.5023 | 45.49893 |
| *Lithobates catesbeianus* | -97.0923 | 32.62018 |
| *Lithobates catesbeianus* | -119.304 | 34.35029 |
| *Lithobates catesbeianus* | -74.2181 | 40.0978 |
| *Lithobates catesbeianus* | -76.5422 | 42.70354 |
| *Lithobates catesbeianus* | -94.6996 | 34.77816 |
| *Lithobates catesbeianus* | 5.12889 | 51.14443 |
| *Lithobates catesbeianus* | 5.12997 | 51.14441 |
| *Lithobates catesbeianus* | 5.12886 | 51.14382 |
| *Lithobates catesbeianus* | -72.9931 | 41.31525 |
| *Lithobates catesbeianus* | -81.6803 | 36.2144 |
| *Lithobates catesbeianus* | -79.9717 | 33.305 |
| *Lithobates catesbeianus* | -75.7936 | 36.2656 |
| *Lithobates catesbeianus* | -77.44 | 37.62 |
| *Lithobates catesbeianus* | -98.335 | 21.054 |
| *Lithobates catesbeianus* | -99.158 | 18.5 |
| *Lithobates catesbeianus* | -116.86 | 32.67869 |
| *Lithobates catesbeianus* | -79.0022 | 45.39813 |
| *Lithobates catesbeianus* | -79.5832 | 42.8709 |
| *Lithobates catesbeianus* | -76.5437 | 44.2289 |
| *Lithobates catesbeianus* | -79.2151 | 43.081 |
| *Lithobates catesbeianus* | -88.669 | 44.0725 |
| *Lithobates catesbeianus* | -86.5596 | 30.925 |
| *Lithobates catesbeianus* | -74.7702 | 5.38505 |
| *Lithobates catesbeianus* | -75.795 | 36.0786 |
| *Lithobates catesbeianus* | -90.972 | 37.236 |
| *Lithobates catesbeianus* | -73.1138 | 41.56718 |
| *Lithobates catesbeianus* | -95.244 | 38.3011 |
| *Lithobates catesbeianus* | -99.7016 | 38.78968 |
| *Lithobates catesbeianus* | -95.1245 | 37.48807 |
| *Lithobates catesbeianus* | -75.5 | 45.25 |
| *Lithobates catesbeianus* | -65.017 | 44.367 |
| *Lithobates catesbeianus* | -75.817 | 45.633 |
| *Lithobates catesbeianus* | -76.355 | 44.611 |
| *Lithobates catesbeianus* | -76.015 | 45.643 |
| *Lithobates catesbeianus* | -76.074 | 45.599 |
| *Lithobates catesbeianus* | -76.101 | 45.621 |
| *Lithobates catesbeianus* | -76.104 | 45.628 |
| *Lithobates catesbeianus* | -76.008 | 45.606 |
| *Lithobates catesbeianus* | -65.308 | 44.717 |
| *Lithobates catesbeianus* | -76.425 | 45.275 |
| *Lithobates catesbeianus* | -75.683 | 44.97 |
| *Lithobates catesbeianus* | -81.554 | 45.2 |
| *Lithobates catesbeianus* | -75.817 | 45.542 |
| *Lithobates catesbeianus* | -76.017 | 45.417 |
| *Lithobates catesbeianus* | -75.79 | 46.198 |
| *Lithobates catesbeianus* | -76.746 | 44.771 |
| *Lithobates catesbeianus* | -75.798 | 45.372 |
| *Lithobates catesbeianus* | 0 | 46.883 |
| *Lithobates catesbeianus* | -76.906 | 44.743 |
| *Lithobates catesbeianus* | -81.733 | 46.15 |
| *Lithobates catesbeianus* | -76.833 | 45.033 |
| *Lithobates catesbeianus* | -75.65 | 45.05 |
| *Lithobates catesbeianus* | -84.133 | 46.617 |
| *Lithobates catesbeianus* | -77.483 | 45.617 |
| *Lithobates catesbeianus* | -73.933 | 45.4 |
| *Lithobates catesbeianus* | -75.8 | 44.983 |
| *Lithobates catesbeianus* | -76.05 | 36.87 |
| *Lithobates catesbeianus* | -94.3473 | 30.40033 |
| *Lithobates catesbeianus* | -97.2958 | 30.98611 |
| *Lithobates catesbeianus* | -97.177 | 30.04738 |
| *Lithobates catesbeianus* | -98.6516 | 33.95105 |
| *Lithobates catesbeianus* | -100.457 | 35.9516 |
| *Lithobates catesbeianus* | -117.148 | 32.93814 |
| *Lithobates catesbeianus* | -116.27 | 35.97557 |
| *Lithobates catesbeianus* | -117.644 | 33.88312 |
| *Lithobates catesbeianus* | -108.99 | 25.7903 |
| *Lithobates catesbeianus* | -118.704 | 34.06419 |
| *Lithobates catesbeianus* | -119.026 | 36.06697 |
| *Lithobates catesbeianus* | -96.7101 | 28.97954 |
| *Lithobates catesbeianus* | -95.1945 | 39.1183 |
| *Lithobates catesbeianus* | -120.492 | 35.18546 |
| *Lithobates catesbeianus* | -111.105 | 31.39064 |
| *Lithobates catesbeianus* | -84.7525 | 39.33544 |
| *Lithobates catesbeianus* | -84.5978 | 41.46933 |
| *Lithobates catesbeianus* | -80.8337 | 41.41756 |
| *Lithobates catesbeianus* | -84.7463 | 39.3001 |
| *Lithobates catesbeianus* | -83.2631 | 39.65637 |
| *Lithobates catesbeianus* | -95.0839 | 38.51688 |
| *Lithobates catesbeianus* | -94.8323 | 37.79788 |
| *Lithobates catesbeianus* | 139.919 | 36.0374 |
| *Lithobates catesbeianus* | -83.9291 | 39.01881 |
| *Lithobates catesbeianus* | -83.6125 | 41.00642 |
| *Lithobates catesbeianus* | -114.715 | 33.38905 |
| *Lithobates catesbeianus* | -123.205 | 39.81769 |
| *Lithobates catesbeianus* | -100.122 | 28.63143 |
| *Lithobates catesbeianus* | -120.712 | 35.34102 |
| *Lithobates catesbeianus* | -122.559 | 39.2907 |
| *Lithobates catesbeianus* | -121.367 | 38.32455 |
| *Lithobates catesbeianus* | -123.081 | 38.43217 |
| *Lithobates catesbeianus* | -119.129 | 36.87969 |
| *Lithobates catesbeianus* | -96.4839 | 30.57042 |
| *Lithobates catesbeianus* | -100.513 | 36.83839 |
| *Lithobates catesbeianus* | -114.4 | 37.8 |
| *Lithobates catesbeianus* | -122.416 | 45.56262 |
| *Lithobates catesbeianus* | -116.191 | 43.60028 |
| *Lithobates catesbeianus* | -116.211 | 43.58661 |
| *Lithobates catesbeianus* | -108.08 | 33.34922 |
| *Lithobates catesbeianus* | -114.624 | 32.7269 |
| *Lithobates catesbeianus* | -111.661 | 33.5469 |
| *Lithobates catesbeianus* | -111.709 | 33.9836 |
| *Lithobates catesbeianus* | -122.8 | 45.7916 |
| *Lithobates catesbeianus* | -114.323 | 48.21161 |
| *Lithobates catesbeianus* | -95.1578 | 43.4983 |
| *Lithobates catesbeianus* | -83.0258 | 39.6445 |
| *Lithobates catesbeianus* | -74.4999 | 40.1671 |
| *Lithobates catesbeianus* | -89.4885 | 34.35978 |
| *Lithobates catesbeianus* | -97.7933 | 30.40685 |
| *Lithobates catesbeianus* | -118.745 | 34.62364 |
| *Lithobates catesbeianus* | -93.1204 | 35.1613 |
| *Lithobates catesbeianus* | -80.2497 | 38.88 |
| *Lithobates catesbeianus* | -79.19 | 40.46 |
| *Lithobates catesbeianus* | -84.49 | 30.58 |
| *Lithobates catesbeianus* | -81.3897 | 38.19002 |
| *Lithobates catesbeianus* | -87.21 | 38.38 |
| *Lithobates catesbeianus* | -75.26 | 39.86 |
| *Lithobates catesbeianus* | -81.7997 | 37.72003 |
| *Lithobates catesbeianus* | -95.4801 | 35.91004 |
| *Lithobates catesbeianus* | -94.9301 | 33.96009 |
| *Lithobates catesbeianus* | -79.7 | 42.07 |
| *Lithobates catesbeianus* | -79.73 | 41.64 |
| *Lithobates catesbeianus* | -94.7191 | 35.46359 |
| *Lithobates catesbeianus* | -97.4548 | 35.24746 |
| *Lithobates catesbeianus* | -96.6359 | 34.43339 |
| *Lithobates catesbeianus* | -94.7676 | 36.79667 |
| *Lithobates catesbeianus* | -94.7049 | 33.75051 |
| *Lithobates catesbeianus* | -94.5956 | 34.6454 |
| *Lithobates catesbeianus* | -97.2983 | 36.80583 |
| *Lithobates catesbeianus* | -82.3012 | 35.4363 |
| *Lithobates catesbeianus* | -82.2957 | 35.4409 |
| *Lithobates catesbeianus* | -82.2769 | 35.4487 |
| *Lithobates catesbeianus* | -78.4307 | 35.8415 |
| *Lithobates catesbeianus* | -78.5277 | 34.4814 |
| *Lithobates catesbeianus* | -78.6648 | 35.5177 |
| *Lithobates catesbeianus* | -78.9806 | 36.015 |
| *Lithobates catesbeianus* | -78.9776 | 36.014 |
| *Lithobates catesbeianus* | -82.5668 | 35.3884 |
| *Lithobates catesbeianus* | -81.4093 | 36.5198 |
| *Lithobates catesbeianus* | -79.1091 | 35.4161 |
| *Lithobates catesbeianus* | -75.5654 | 39.3116 |
| *Lithobates catesbeianus* | -75.2669 | 38.7613 |
| *Lithobates catesbeianus* | -75.2674 | 39.5277 |
| *Lithobates catesbeianus* | -74.1754 | 39.9697 |
| *Lithobates catesbeianus* | -77.3222 | 34.6293 |
| *Lithobates catesbeianus* | -77.025 | 37.4781 |
| *Lithobates catesbeianus* | -77.6103 | 37.6861 |
| *Lithobates catesbeianus* | -77.2186 | 37.4888 |
| *Lithobates catesbeianus* | -79.9252 | 34.9666 |
| *Lithobates catesbeianus* | -80.9443 | 35.4074 |
| *Lithobates catesbeianus* | -79.3306 | 35.9284 |
| *Lithobates catesbeianus* | -96.2112 | 30.72033 |
| *Lithobates catesbeianus* | -96.9712 | 32.63407 |
| *Lithobates catesbeianus* | -157.728 | 21.3328 |
| *Lithobates catesbeianus* | -76.3169 | 39.5258 |
| *Lithobates catesbeianus* | -91.7588 | 32.75522 |
| *Lithobates catesbeianus* | -95.2754 | 38.88692 |
| *Lithobates catesbeianus* | -83.3085 | 35.5534 |
| *Lithobates catesbeianus* | -116.214 | 43.61125 |
| *Lithobates catesbeianus* | -68.7103 | 45.6572 |
| *Lithobates catesbeianus* | -77.6078 | 39.62359 |
| *Lithobates catesbeianus* | -77.6772 | 39.0432 |
| *Lithobates catesbeianus* | -75.64 | 37.71 |
| *Lithobates catesbeianus* | -77.4683 | 36.8143 |
| *Lithobates catesbeianus* | -73.3431 | 43.09028 |
| *Lithobates catesbeianus* | -117.205 | 32.99928 |
| *Lithobates catesbeianus* | -77.0441 | 35.1085 |
| *Lithobates catesbeianus* | -90.1178 | 29.78347 |
| *Lithobates catesbeianus* | -82.75 | 46.66666 |
| *Lithobates catesbeianus* | -77.27 | 40.09 |
| *Lithobates catesbeianus* | -86.37 | 33.86 |
| *Lithobates catesbeianus* | -98.1302 | 36.74001 |
| *Lithobates catesbeianus* | -92.35 | 29.69 |
| *Lithobates catesbeianus* | -110.81 | 32.31 |
| *Lithobates catesbeianus* | -87.21 | 32.68 |
| *Lithobates catesbeianus* | -84.16 | 30.63 |
| *Lithobates catesbeianus* | -78.05 | 40.89 |
| *Lithobates catesbeianus* | -74.82 | 41.43 |
| *Lithobates catesbeianus* | -78.36 | 37.31 |
| *Lithobates catesbeianus* | -76.45 | 36.74 |
| *Lithobates catesbeianus* | -122.474 | 46.8237 |
| *Lithobates catesbeianus* | -91.1218 | 43.90795 |
| *Lithobates catesbeianus* | -117.132 | 32.77133 |
| *Lithobates catesbeianus* | -105.043 | 39.88613 |
| *Lithobates catesbeianus* | -122.44 | 47.25 |
| *Lithobates catesbeianus* | -122.92 | 47.47 |
| *Lithobates catesbeianus* | -76.724 | 35.35848 |
| *Lithobates catesbeianus* | -88.446 | 38.87194 |
| *Lithobates catesbeianus* | -97.0581 | 36.11603 |
| *Lithobates catesbeianus* | -122.2 | 47.61032 |
| *Lithobates catesbeianus* | -88.0517 | 42.62825 |
| *Lithobates catesbeianus* | -122.609 | 39.65625 |
| *Lithobates catesbeianus* | -96.1993 | 30.65074 |
| *Lithobates catesbeianus* | -77.1051 | 38.7545 |
| *Lithobates catesbeianus* | -97.2803 | 32.88403 |
| *Lithobates catesbeianus* | -121.723 | 37.39903 |
| *Lithobates catesbeianus* | -82.956 | 36.62688 |
| *Lithobates catesbeianus* | -72.2376 | 41.82391 |
| *Lithobates catesbeianus* | -97.3443 | 32.56889 |
| *Lithobates catesbeianus* | -76.7251 | 42.46393 |
| *Lithobates catesbeianus* | -71.1499 | 42.38857 |
| *Lithobates catesbeianus* | -72.5643 | 41.31446 |
| *Lithobates catesbeianus* | -71.5494 | 41.98861 |
| *Lithobates catesbeianus* | -96.9895 | 37.24033 |
| *Lithobates catesbeianus* | -81.3961 | 41.00898 |
| *Lithobates catesbeianus* | -123.403 | 39.03485 |
| *Lithobates catesbeianus* | -77.0538 | 38.93088 |
| *Lithobates catesbeianus* | -95.6874 | 31.65294 |
| *Lithobates catesbeianus* | -105.194 | 40.04352 |
| *Lithobates catesbeianus* | -73.2126 | 41.72276 |
| *Lithobates catesbeianus* | -78.9785 | 44.92748 |
| *Lithobates catesbeianus* | -90.6907 | 29.62846 |
| *Lithobates catesbeianus* | -78.8201 | 37.84581 |
| *Lithobates catesbeianus* | -96.9037 | 37.51965 |
| *Lithobates catesbeianus* | -123.912 | 46.08509 |
| *Lithobates catesbeianus* | -95.3493 | 32.60648 |
| *Lithobates catesbeianus* | -77.2569 | 38.98185 |
| *Lithobates catesbeianus* | -72.237 | 41.82369 |
| *Lithobates catesbeianus* | -123.948 | 46.15023 |
| *Lithobates catesbeianus* | -121.905 | 36.84963 |
| *Lithobates catesbeianus* | -118.191 | 34.0936 |
| *Lithobates catesbeianus* | -81.2009 | 22.28549 |
| *Lithobates catesbeianus* | -117.404 | 33.53546 |
| *Lithobates catesbeianus* | -73.5311 | 43.60683 |
| *Lithobates catesbeianus* | -74.4555 | 40.40935 |
| *Lithobates catesbeianus* | -121.624 | 37.26335 |
| *Lithobates catesbeianus* | -122.487 | 47.44738 |
| *Lithobates catesbeianus* | -122.061 | 37.04003 |
| *Lithobates catesbeianus* | -122.33 | 39.82292 |
| *Lithobates catesbeianus* | 126.3316 | 36.7166 |
| *Lithobates catesbeianus* | -95.8671 | 30.08203 |
| *Lithobates catesbeianus* | -78.2838 | 46.06248 |
| *Lithobates catesbeianus* | -106.891 | 33.80449 |
| *Lithobates catesbeianus* | -97.2644 | 37.73983 |
| *Lithobates catesbeianus* | -68.7748 | -31.5187 |
| *Lithobates catesbeianus* | -123.157 | 39.16582 |
| *Lithobates catesbeianus* | -122.811 | 38.16197 |
| *Lithobates catesbeianus* | -122.587 | 41.81596 |
| *Lithobates catesbeianus* | -109.347 | 32.43856 |
| *Lithobates catesbeianus* | -72.6093 | 41.75693 |
| *Lithobates catesbeianus* | -118.797 | 34.25548 |
| *Lithobates catesbeianus* | -95.9022 | 31.96198 |
| *Lithobates catesbeianus* | -96.806 | 28.30132 |
| *Lithobates catesbeianus* | -122.956 | 48.6877 |
| *Lithobates catesbeianus* | -84.3978 | 37.15784 |
| *Lithobates catesbeianus* | -94.2485 | 30.14133 |
| *Lithobates catesbeianus* | -79.0335 | 35.89923 |
| *Lithobates catesbeianus* | -98.0082 | 32.79462 |
| *Lithobates catesbeianus* | -81.3889 | 41.27816 |
| *Lithobates catesbeianus* | -95.9532 | 28.75984 |
| *Lithobates catesbeianus* | -76.4784 | 42.44224 |
| *Lithobates catesbeianus* | -76.4663 | 42.44127 |
| *Lithobates catesbeianus* | -74.4747 | 40.9869 |
| *Lithobates catesbeianus* | -74.0769 | 41.20917 |
| *Lithobates catesbeianus* | -73.804 | 41.16604 |
| *Lithobates catesbeianus* | -79.7073 | 42.06756 |
| *Lithobates catesbeianus* | -72.3611 | 44.6947 |
| *Lithobates catesbeianus* | -74.5497 | 40.7061 |
| *Lithobates catesbeianus* | -97.4266 | 35.24309 |
| *Lithobates catesbeianus* | -72.0713 | 41.5444 |
| *Lithobates catesbeianus* | -111.87 | 40.75 |
| *Lithobates catesbeianus* | -97.2611 | 30.51675 |
| *Lithobates catesbeianus* | -82.0489 | 41.3938 |
| *Lithobates catesbeianus* | -80.3336 | 33.1895 |
| *Lithobates catesbeianus* | -80.4667 | 42.59028 |
| *Lithobates catesbeianus* | -80.15 | 45.9 |
| *Lithobates catesbeianus* | -76.6858 | 44.76503 |
| *Lithobates catesbeianus* | -118.411 | 34.12024 |
| *Lithobates catesbeianus* | -80.3447 | 33.2183 |
| *Lithobates catesbeianus* | -83.753 | 43.6295 |
| *Lithobates catesbeianus* | -83.5679 | 45.9417 |
| *Lithobates catesbeianus* | -86.3491 | 43.4095 |
| *Lithobates catesbeianus* | -80.8667 | 43.9833 |
| *Lithobates catesbeianus* | -79.8202 | 44.4149 |
| *Lithobates catesbeianus* | -79.3271 | 42.9091 |
| *Lithobates catesbeianus* | -79.1671 | 42.9206 |
| *Lithobates catesbeianus* | -119.783 | 39.4666 |
| *Lithobates catesbeianus* | -118.967 | 39.483 |
| *Lithobates catesbeianus* | -82.0231 | 29.6032 |
| *Lithobates catesbeianus* | -84.8875 | 30.63681 |
| *Lithobates catesbeianus* | -84.8532 | 30.4121 |
| *Lithobates catesbeianus* | -82.3749 | 27.594 |
| *Lithobates catesbeianus* | -82.227 | 29.51589 |
| *Lithobates catesbeianus* | -89.4519 | 40.72238 |
| *Lithobates catesbeianus* | -84.5865 | 29.88526 |
| *Lithobates catesbeianus* | -95.5268 | 38.5797 |
| *Lithobates catesbeianus* | -95.7245 | 39.05858 |
| *Lithobates catesbeianus* | -84.6548 | 39.75837 |
| *Lithobates catesbeianus* | -95.5137 | 39.52719 |
| *Lithobates catesbeianus* | -95.624 | 39.50901 |
| *Lithobates catesbeianus* | -95.299 | 37.88917 |
| *Lithobates catesbeianus* | -95.2334 | 38.31499 |
| *Lithobates catesbeianus* | -102.026 | 39.67129 |
| *Lithobates catesbeianus* | -96.7429 | 39.12247 |
| *Lithobates catesbeianus* | -96.335 | 30.63278 |
| *Lithobates catesbeianus* | -85.0767 | 33.62111 |
| *Lithobates catesbeianus* | -94.3775 | 29.76639 |
| *Lithobates catesbeianus* | -92.6191 | 32.75516 |
| *Lithobates catesbeianus* | -98.0248 | 39.886 |
| *Lithobates catesbeianus* | -94.6872 | 37.1039 |
| *Lithobates catesbeianus* | -95.6928 | 38.56695 |
| *Lithobates catesbeianus* | -97.9409 | 38.21719 |
| *Lithobates catesbeianus* | -99.0491 | 38.87411 |
| *Lithobates catesbeianus* | -90.2854 | 38.63903 |
| *Lithobates catesbeianus* | -121.388 | 36.06473 |
| *Lithobates catesbeianus* | -90.5591 | 39.93118 |
| *Lithobates catesbeianus* | -120.687 | 35.33724 |
| *Lithobates catesbeianus* | -122.518 | 38.34747 |
| *Lithobates catesbeianus* | -119.125 | 36.86342 |
| *Lithobates catesbeianus* | -118.772 | 34.11175 |
| *Lithobates catesbeianus* | -108.42 | 25.55032 |
| *Lithobates catesbeianus* | -84.6665 | 39.57923 |
| *Lithobates catesbeianus* | -83.7517 | 38.9822 |
| *Lithobates catesbeianus* | -84.1264 | 39.43333 |
| *Lithobates catesbeianus* | -83.056 | 39.33179 |
| *Lithobates catesbeianus* | -84.5715 | 39.52554 |
| *Lithobates catesbeianus* | -80.5 | 45.56 |
| *Lithobates catesbeianus* | -81.12 | 28.61 |
| *Lithobates catesbeianus* | -82.53 | 29.4 |
| *Lithobates catesbeianus* | -79.05 | 40.66 |
| *Lithobates catesbeianus* | -86.26 | 39.16 |
| *Lithobates catesbeianus* | -86.26 | 39.07 |
| *Lithobates catesbeianus* | -94.8201 | 33.89009 |
| *Lithobates catesbeianus* | -82.4697 | 38.32001 |
| *Lithobates catesbeianus* | -79.92 | 40.5 |
| *Lithobates catesbeianus* | -80.02 | 40.49 |
| *Lithobates catesbeianus* | -82.21 | 29.59 |
| *Lithobates catesbeianus* | -78.46 | 40.01 |
| *Lithobates catesbeianus* | -96.6001 | 33.64009 |
| *Lithobates catesbeianus* | -95.2001 | 38.97998 |
| *Lithobates catesbeianus* | -77.92 | 39.99 |
| *Lithobates catesbeianus* | -81.89 | 29.33 |
| *Lithobates catesbeianus* | -76.83 | 37.25 |
| *Lithobates catesbeianus* | -75.6128 | 5.72306 |
| *Lithobates catesbeianus* | -76.433 | 44.8 |
| *Lithobates catesbeianus* | -82.683 | 41.75 |
| *Lithobates catesbeianus* | -73.583 | 45.033 |
| *Lithobates catesbeianus* | -76.317 | 45.183 |
| *Lithobates catesbeianus* | -75.733 | 45.267 |
| *Lithobates catesbeianus* | -76.367 | 44.6 |
| *Lithobates catesbeianus* | -75.4 | 45.25 |
| *Lithobates catesbeianus* | -74.033 | 46.522 |
| *Lithobates catesbeianus* | -75.65 | 45.068 |
| *Lithobates catesbeianus* | -76.012 | 45.606 |
| *Lithobates catesbeianus* | -76.068 | 45.598 |
| *Lithobates catesbeianus* | -76.155 | 45.657 |
| *Lithobates catesbeianus* | -74.483 | 45.75 |
| *Lithobates catesbeianus* | -75.695 | 45.322 |
| *Lithobates catesbeianus* | -76.324 | 46.2 |
| *Lithobates catesbeianus* | -74.133 | 45.45 |
| *Lithobates catesbeianus* | -75.797 | 45.458 |
| *Lithobates catesbeianus* | -76.131 | 45.186 |
| *Lithobates catesbeianus* | -73.995 | 45.42 |
| *Lithobates catesbeianus* | -76.57 | 45.179 |
| *Lithobates catesbeianus* | -79.4 | 44.95 |
| *Lithobates catesbeianus* | -87.333 | 30.717 |
| *Lithobates catesbeianus* | -84.917 | 39.633 |
| *Lithobates catesbeianus* | -75.652 | 44.877 |
| *Lithobates catesbeianus* | -65.233 | 44.383 |
| *Lithobates catesbeianus* | -76.6889 | 38.8583 |
| *Lithobates catesbeianus* | -86.4034 | 41.2023 |
| *Lithobates catesbeianus* | -89.9306 | 38.46502 |
| *Lithobates catesbeianus* | -85.1916 | 34.2643 |
| *Lithobates catesbeianus* | -109.763 | 32.03763 |
| *Lithobates catesbeianus* | -76.7952 | 39.0415 |
| *Lithobates catesbeianus* | -81.64 | 36.2208 |
| *Lithobates catesbeianus* | -77.54 | 37.83 |
| *Lithobates catesbeianus* | -80.1155 | 36.7993 |
| *Lithobates catesbeianus* | -122.785 | 38.61283 |
| *Lithobates catesbeianus* | -77.6944 | 45.84889 |
| *Lithobates catesbeianus* | -77.675 | 45.83472 |
| *Lithobates catesbeianus* | -98.3694 | 34.62757 |
| *Lithobates catesbeianus* | -94.7699 | 35.96619 |
| *Lithobates catesbeianus* | -94.4639 | 34.7081 |
| *Lithobates catesbeianus* | -94.6207 | 36.64867 |
| *Lithobates catesbeianus* | -94.7924 | 36.11009 |
| *Lithobates catesbeianus* | -94.8 | 36.39988 |
| *Lithobates catesbeianus* | -94.7357 | 35.48699 |
| *Lithobates catesbeianus* | -94.718 | 36.16739 |
| *Lithobates catesbeianus* | -94.5023 | 34.51872 |
| *Lithobates catesbeianus* | -75.6603 | 35.8883 |
| *Lithobates catesbeianus* | -80.0706 | 35.3714 |
| *Lithobates catesbeianus* | -77.946 | 35.7777 |
| *Lithobates catesbeianus* | -78.7922 | 35.6489 |
| *Lithobates catesbeianus* | -80.7401 | 34.823 |
| *Lithobates catesbeianus* | -81.7549 | 35.6407 |
| *Lithobates catesbeianus* | -78.1552 | 36.3984 |
| *Lithobates catesbeianus* | -79.1813 | 34.8861 |
| *Lithobates catesbeianus* | -83.2559 | 30.7253 |
| *Lithobates catesbeianus* | -75.2395 | 39.4461 |
| *Lithobates catesbeianus* | -74.5982 | 40.1068 |
| *Lithobates catesbeianus* | -80.7287 | 34.7353 |
| *Lithobates catesbeianus* | -83.2112 | 34.844 |
| *Lithobates catesbeianus* | -77.9833 | 33.9997 |
| *Lithobates catesbeianus* | -83.2439 | 34.814 |
| *Lithobates catesbeianus* | -77.3227 | 34.6252 |
| *Lithobates catesbeianus* | -81.7786 | 35.7262 |
| *Lithobates catesbeianus* | -78.5216 | 36.5804 |
| *Lithobates catesbeianus* | -82.7855 | 35.0717 |
| *Lithobates catesbeianus* | 126.3466 | 36.72733 |
| *Lithobates catesbeianus* | -83.2586 | 38.622 |
| *Lithobates catesbeianus* | -106.613 | 31.84702 |
| *Lithobates catesbeianus* | -118.175 | 34.18523 |
| *Lithobates catesbeianus* | -118.492 | 34.17619 |
| *Lithobates catesbeianus* | -117.268 | 34.24266 |
| *Lithobates catesbeianus* | -116.293 | 33.66336 |
| *Lithobates catesbeianus* | -117.858 | 33.65835 |
| *Lithobates catesbeianus* | 135.3728 | 34.80347 |
| *Lithobates catesbeianus* | -121.937 | 43.63608 |
| *Lithobates catesbeianus* | -110.55 | 32.11369 |
| *Lithobates catesbeianus* | -109.842 | 31.94073 |
| *Lithobates catesbeianus* | -71.4158 | 42.465 |
| *Lithobates catesbeianus* | -95.9333 | 41.45 |
| *Lithobates catesbeianus* | -114.727 | 32.6067 |
| *Lithobates catesbeianus* | -67.101 | 18.01385 |
| *Lithobates catesbeianus* | -117.545 | 33.56485 |
| *Lithobates catesbeianus* | -157.856 | 21.319 |
| *Lithobates catesbeianus* | -157.795 | 21.4114 |
| *Lithobates catesbeianus* | -159.536 | 22.0674 |
| *Lithobates catesbeianus* | -73.1777 | 44.01908 |
| *Lithobates catesbeianus* | -97.0272 | 30.38392 |
| *Lithobates catesbeianus* | -122.601 | 45.63454 |
| *Lithobates catesbeianus* | -97.2807 | 32.8837 |
| *Lithobates catesbeianus* | -84.4769 | 39.3438 |
| *Lithobates catesbeianus* | -75.9396 | 38.95539 |
| *Lithobates catesbeianus* | -95.6117 | 29.3779 |
| *Lithobates catesbeianus* | -73.1777 | 44.01912 |
| *Lithobates catesbeianus* | -72.4597 | 41.63136 |
| *Lithobates catesbeianus* | -118.896 | 36.55105 |
| *Lithobates catesbeianus* | -120.479 | 37.66512 |
| *Lithobates catesbeianus* | -122.141 | 37.93797 |
| *Lithobates catesbeianus* | -121.752 | 39.30376 |
| *Lithobates catesbeianus* | -79.9763 | 41.06337 |
| *Lithobates catesbeianus* | -82.9376 | 34.97169 |
| *Lithobates catesbeianus* | -116.866 | 36.4567 |
| *Lithobates catesbeianus* | -90.6089 | 39.36879 |
| *Lithobates catesbeianus* | -120.193 | 37.36762 |
| *Lithobates catesbeianus* | -123.085 | 49.09446 |
| *Lithobates catesbeianus* | -118.217 | 34.00812 |
| *Lithobates catesbeianus* | -121.696 | 37.23964 |
| *Lithobates catesbeianus* | -97.181 | 32.72702 |
| *Lithobates catesbeianus* | -97.4764 | 35.59574 |
| *Lithobates catesbeianus* | -71.2435 | 42.05779 |
| *Lithobates catesbeianus* | -105.101 | 39.84135 |
| *Lithobates catesbeianus* | -72.6398 | 41.76695 |
| *Lithobates catesbeianus* | -95.7179 | 29.6278 |
| *Lithobates catesbeianus* | -118.201 | 34.09767 |
| *Lithobates catesbeianus* | -61.3243 | 10.45503 |
| *Lithobates catesbeianus* | -71.1183 | 43.83444 |
| *Lithobates catesbeianus* | -71.4847 | 43.38472 |
| *Lithobates catesbeianus* | -79.593 | 43.83272 |
| *Lithobates catesbeianus* | -72.28 | 44.30791 |
| *Lithobates catesbeianus* | -94.7254 | 31.37468 |
| *Lithobates catesbeianus* | -69.4493 | -31.2391 |
| *Lithobates catesbeianus* | -95.22 | 39.02295 |
| *Lithobates catesbeianus* | -88.1431 | 41.82642 |
| *Lithobates catesbeianus* | -94.0985 | 32.68322 |
| *Lithobates catesbeianus* | -122.839 | 45.49821 |
| *Lithobates catesbeianus* | -121.613 | 37.23541 |
| *Lithobates catesbeianus* | -116.956 | 32.69122 |
| *Lithobates catesbeianus* | -72.6979 | 43.44301 |
| *Lithobates catesbeianus* | -122.724 | 38.08651 |
| *Lithobates catesbeianus* | -71.6485 | 43.12631 |
| *Lithobates catesbeianus* | -97.1 | 32.58773 |
| *Lithobates catesbeianus* | -96.3228 | 29.85193 |
| *Lithobates catesbeianus* | -95.8875 | 31.92945 |
| *Lithobates catesbeianus* | -116.383 | 35.03752 |
| *Lithobates catesbeianus* | -98.2586 | 36.79664 |
| *Lithobates catesbeianus* | -98.5458 | 28.55192 |
| *Lithobates catesbeianus* | -97.2204 | 30.09636 |
| *Lithobates catesbeianus* | -95.5701 | 29.77149 |
| *Lithobates catesbeianus* | -78.1685 | 42.43374 |
| *Lithobates catesbeianus* | 135.1026 | 34.66549 |
| *Lithobates catesbeianus* | -71.1425 | 42.99083 |
| *Lithobates catesbeianus* | -95.6414 | 29.37358 |
| *Lithobates catesbeianus* | -98.53 | 33.93979 |
| *Lithobates catesbeianus* | -89.0208 | 37.25472 |
| *Lithobates catesbeianus* | 7.78862 | 44.93572 |
| *Lithobates catesbeianus* | -94.4186 | 30.29102 |
| *Lithobates catesbeianus* | -96.2694 | 29.67346 |
| *Lithobates catesbeianus* | -96.8003 | 28.11696 |
| *Lithobates catesbeianus* | -75.7256 | 39.82182 |
| *Lithobates catesbeianus* | -97.4963 | 32.85355 |
| *Lithobates catesbeianus* | -76.551 | 44.38844 |
| *Lithobates catesbeianus* | -123.349 | 44.5202 |
| *Lithobates catesbeianus* | -75.0746 | 41.00257 |
| *Lithobates catesbeianus* | -76.4269 | 42.46501 |
| *Lithobates catesbeianus* | -75.498 | 45.50176 |
| *Lithobates catesbeianus* | -88.3793 | 40.19861 |
| *Lithobates catesbeianus* | -68.2463 | 44.39654 |
| *Lithobates catesbeianus* | -70.2777 | 41.70829 |
| *Lithobates catesbeianus* | -118.213 | 34.20068 |
| *Lithobates catesbeianus* | -97.4864 | 35.6374 |
| *Lithobates catesbeianus* | -78.1525 | 42.5566 |
| *Lithobates catesbeianus* | -82.0578 | 29.21646 |
| *Lithobates catesbeianus* | -123.943 | 46.17222 |
| *Lithobates catesbeianus* | -75.7605 | 39.71886 |
| *Lithobates catesbeianus* | -72.14 | 4.28 |
| *Lithobates catesbeianus* | -73.9529 | 42.07759 |
| *Lithobates catesbeianus* | -123.794 | 39.49134 |
| *Lithobates catesbeianus* | -78.3667 | 45.11666 |
| *Lithobates catesbeianus* | -76.2922 | 44.56308 |
| *Lithobates catesbeianus* | -157.817 | 21.41667 |
| *Lithobates catesbeianus* | 5.12999 | 51.14378 |
| *Lithobates catesbeianus* | 5.1301 | 51.14406 |
| *Lithobates catesbeianus* | -122.206 | 37.85943 |
| *Lithobates catesbeianus* | -83.05 | 40.4 |
| *Lithobates catesbeianus* | -68.2074 | 44.35717 |
| *Lithobates catesbeianus* | -73.3299 | 41.84748 |
| *Lithobates catesbeianus* | -96.783 | 20.183 |
| *Lithobates catesbeianus* | -74.9023 | 40.21633 |
| *Lithobates catesbeianus* | -72.5773 | 43.10536 |
| *Lithobates catesbeianus* | -84.1839 | 36.17859 |
| *Lithobates catesbeianus* | -87.0942 | 36.12395 |
| *Lithobates catesbeianus* | -89.3167 | 36.13345 |
| *Lithobates catesbeianus* | -82.7873 | 31.0354 |
| *Lithobates catesbeianus* | -76.9341 | 38.99037 |
| *Lithobates catesbeianus* | -74.8544 | 43.75314 |
| *Lithobates catesbeianus* | -83.0434 | 35.11899 |
| *Lithobates catesbeianus* | -81.9008 | 29.3731 |
| *Lithobates catesbeianus* | -88.0294 | 37.80553 |
| *Lithobates catesbeianus* | -83.5512 | 34.82929 |
| *Lithobates catesbeianus* | -77.2706 | 37.08841 |
| *Lithobates catesbeianus* | -69.9333 | 18.5 |
| *Lithobates catesbeianus* | -77.1002 | 36.9281 |
| *Lithobates catesbeianus* | -77.8623 | 37.6097 |
| *Lithobates catesbeianus* | -76.2 | 36.55 |
| *Lithobates catesbeianus* | -82.8062 | 41.6881 |
| *Lithobates catesbeianus* | -69.2498 | 45.5003 |
| *Lithobates catesbeianus* | -72.1165 | 43.4092 |
| *Lithobates catesbeianus* | -101.831 | 36.24668 |
| *Lithobates catesbeianus* | -109.71 | 32.7587 |
| *Lithobates catesbeianus* | -95.4188 | 36.54459 |
| *Lithobates catesbeianus* | -98.2472 | 34.59398 |
| *Lithobates catesbeianus* | -96.7239 | 35.27537 |
| *Lithobates catesbeianus* | -77.5011 | 39.4245 |
| *Lithobates catesbeianus* | -94.9553 | 34.7523 |
| *Lithobates catesbeianus* | -94.9009 | 35.88439 |
| *Lithobates catesbeianus* | -93.1541 | 36.27542 |
| *Lithobates catesbeianus* | -97.1472 | 34.42619 |
| *Lithobates catesbeianus* | -74.6425 | 39.84861 |
| *Lithobates catesbeianus* | -94.6648 | 36.13828 |
| *Lithobates catesbeianus* | -94.6131 | 34.6454 |
| *Lithobates catesbeianus* | -101.647 | 36.71213 |
| *Lithobates catesbeianus* | -98.5101 | 35.3508 |
| *Lithobates catesbeianus* | -78.7538 | 35.8572 |
| *Lithobates catesbeianus* | -77.9443 | 34.058 |
| *Lithobates catesbeianus* | -78.6397 | 35.5194 |
| *Lithobates catesbeianus* | -78.2048 | 35.8493 |
| *Lithobates catesbeianus* | -79.4052 | 34.8526 |
| *Lithobates catesbeianus* | -82.0384 | 35.8052 |
| *Lithobates catesbeianus* | -78.581 | 34.0141 |
| *Lithobates catesbeianus* | -81.0565 | 35.355 |
| *Lithobates catesbeianus* | -82.0307 | 35.7964 |
| *Lithobates catesbeianus* | -81.0204 | 36.5512 |
| *Lithobates catesbeianus* | -78.3634 | 34.163 |
| *Lithobates catesbeianus* | -80.4892 | 34.7205 |
| *Lithobates catesbeianus* | -75.4663 | 38.525 |
| *Lithobates catesbeianus* | -78.6224 | 34.8743 |
| *Lithobates catesbeianus* | -74.1265 | 40.3486 |
| *Lithobates catesbeianus* | -79.5617 | 35.1004 |
| *Lithobates catesbeianus* | -77.0694 | 37.4238 |
| *Lithobates catesbeianus* | -77.4833 | 37.6892 |
| *Lithobates catesbeianus* | -77.4742 | 37.5656 |
| *Lithobates catesbeianus* | -77.4678 | 37.8961 |
| *Lithobates catesbeianus* | -78.8003 | 36.7177 |
| *Lithobates catesbeianus* | -82.5371 | 35.7898 |
| *Lithobates catesbeianus* | -158.007 | 21.3727 |
| *Lithobates catesbeianus* | -94.698 | 29.7906 |
| *Lithobates catesbeianus* | -93.3243 | 35.51326 |
| *Lithobates catesbeianus* | -80.5515 | 44.26258 |
| *Lithobates catesbeianus* | -116.895 | 32.8662 |
| *Lithobates catesbeianus* | -82.1862 | 29.6467 |
| *Lithobates catesbeianus* | -84.0104 | 30.17724 |
| *Lithobates catesbeianus* | -84.1185 | 30.13998 |
| *Lithobates catesbeianus* | -84.5148 | 30.06394 |
| *Lithobates catesbeianus* | -82.2828 | 29.51178 |
| *Lithobates catesbeianus* | -81.2543 | 27.59284 |
| *Lithobates catesbeianus* | -86.2192 | 39.14592 |
| *Lithobates catesbeianus* | -111.55 | 31.55 |
| *Lithobates catesbeianus* | -77.26 | 46.555 |
| *Lithobates catesbeianus* | -80.383 | 42.583 |
| *Lithobates catesbeianus* | -72.35 | 46 |
| *Lithobates catesbeianus* | -75.25 | 45.6 |
| *Lithobates catesbeianus* | -77.124 | 44.108 |
| *Lithobates catesbeianus* | -122.617 | 49.133 |
| *Lithobates catesbeianus* | -75.669 | 44.988 |
| *Lithobates catesbeianus* | -76.122 | 45.625 |
| *Lithobates catesbeianus* | -75.814 | 45.506 |
| *Lithobates catesbeianus* | -76.225 | 45.682 |
| *Lithobates catesbeianus* | -76.407 | 45.181 |
| *Lithobates catesbeianus* | -76.185 | 45.59 |
| *Lithobates catesbeianus* | -75.987 | 45.638 |
| *Lithobates catesbeianus* | -75.817 | 46.083 |
| *Lithobates catesbeianus* | -75.617 | 45.083 |
| *Lithobates catesbeianus* | -76.422 | 46.328 |
| *Lithobates catesbeianus* | -76.183 | 45.917 |
| *Lithobates catesbeianus* | -75.912 | 46.245 |
| *Lithobates catesbeianus* | -75.817 | 45.2 |
| *Lithobates catesbeianus* | -74.448 | 45.041 |
| *Lithobates catesbeianus* | -76.653 | 45.638 |
| *Lithobates catesbeianus* | -76.653 | 46.059 |
| *Lithobates catesbeianus* | -75.983 | 45.783 |
| *Lithobates catesbeianus* | -79.317 | 45.033 |
| *Lithobates catesbeianus* | -82.05 | 46.25 |
| *Lithobates catesbeianus* | -75.683 | 45.367 |
| *Lithobates catesbeianus* | 0 | 45.483 |
| *Lithobates catesbeianus* | -79.8276 | 32.9764 |
| *Lithobates catesbeianus* | -84.8666 | 34.5 |
| *Lithobates catesbeianus* | -76.95 | 40.24 |
| *Lithobates catesbeianus* | -83.08 | 30.29 |
| *Lithobates catesbeianus* | -78.0496 | 39.49999 |
| *Lithobates catesbeianus* | -79.61 | 41.63 |
| *Lithobates catesbeianus* | -75.15 | 38.31 |
| *Lithobates catesbeianus* | -95.3901 | 38.90998 |
| *Lithobates catesbeianus* | -75.56 | 37.99 |
| *Lithobates catesbeianus* | -82.79 | 36.66 |
| *Lithobates catesbeianus* | -77.35 | 38.13 |
| *Lithobates catesbeianus* | -84.2643 | 39.70878 |
| *Lithobates catesbeianus* | -98.1424 | 38.5077 |
| *Lithobates catesbeianus* | -100.265 | 38.78504 |
| *Lithobates catesbeianus* | -121.287 | 37.99322 |
| *Lithobates catesbeianus* | -120.67 | 38.71875 |
| *Lithobates catesbeianus* | -122.048 | 37.21149 |
| *Lithobates catesbeianus* | -119.479 | 36.73045 |
| *Lithobates catesbeianus* | -121.326 | 37.05386 |
| *Lithobates catesbeianus* | -118.45 | 35.79931 |
| *Lithobates catesbeianus* | -122.733 | 42.68798 |
| *Lithobates catesbeianus* | -100.64 | 31.33075 |
| *Lithobates catesbeianus* | -122.169 | 37.42722 |
| *Lithobates catesbeianus* | -114.604 | 35.07278 |
| *Lithobates catesbeianus* | -108.943 | 39.11088 |
| *Lithobates catesbeianus* | -70.6161 | 41.41678 |
| *Lithobates catesbeianus* | -101.064 | 34.41527 |
| *Lithobates catesbeianus* | -122.476 | 38.26658 |
| *Lithobates catesbeianus* | -117.332 | 33.98585 |
| *Lithobates catesbeianus* | -124.217 | 40.7167 |
| *Lithobates catesbeianus* | -95.1497 | 43.6815 |
| *Lithobates catesbeianus* | -113.972 | 46.86187 |
| *Lithobates catesbeianus* | -97.014 | 38.34887 |
| *Lithobates catesbeianus* | -95.5862 | 37.06883 |
| *Lithobates catesbeianus* | -82.0555 | 39.57205 |
| *Lithobates catesbeianus* | -97.7477 | 38.05749 |
| *Lithobates catesbeianus* | -96.1687 | 37.2293 |
| *Lithobates catesbeianus* | -96.219 | 37.16973 |
| *Lithobates catesbeianus* | -95.272 | 38.17666 |
| *Lithobates catesbeianus* | -98.2074 | 38.5658 |
| *Lithobates catesbeianus* | -97.3338 | 37.08938 |
| *Lithobates catesbeianus* | -109.031 | 31.86429 |
| *Lithobates catesbeianus* | -117.307 | 34.55632 |
| *Lithobates catesbeianus* | -121.767 | 38.58976 |
| *Lithobates catesbeianus* | -117.761 | 33.78003 |
| *Lithobates catesbeianus* | -120.887 | 35.43573 |
| *Lithobates catesbeianus* | -98.7521 | 29.14478 |
| *Lithobates catesbeianus* | -97.4388 | 30.64394 |
| *Lithobates catesbeianus* | -96.9874 | 29.43007 |
| *Lithobates catesbeianus* | -97.2933 | 30.11122 |
| *Lithobates catesbeianus* | -97.2553 | 30.21528 |
| *Lithobates catesbeianus* | -121.985 | 39.01312 |
| *Lithobates catesbeianus* | -83.8972 | 39.18358 |
| *Lithobates catesbeianus* | -83.862 | 40.89897 |
| *Lithobates catesbeianus* | -84.3764 | 39.79889 |
| *Lithobates catesbeianus* | -84.7705 | 39.53483 |
| *Lithobates catesbeianus* | -84.6504 | 39.71367 |
| *Lithobates catesbeianus* | -84.5486 | 40.75692 |
| *Lithobates catesbeianus* | -83.8813 | 39.15528 |
| *Lithobates catesbeianus* | -80.8459 | 41.43468 |
| *Lithobates catesbeianus* | -84.6726 | 39.66683 |
| *Lithobates catesbeianus* | -83.6929 | 38.7192 |
| *Lithobates catesbeianus* | -84.5732 | 39.84386 |
| *Lithobates catesbeianus* | -79.8055 | 44.7581 |
| *Lithobates catesbeianus* | -76.2449 | 44.8343 |
| *Lithobates catesbeianus* | -79.7998 | 44.5167 |
| *Lithobates catesbeianus* | -117.373 | 33.5474 |
| *Lithobates catesbeianus* | -116.977 | 33.49586 |
| *Lithobates catesbeianus* | -73.7936 | 43.36472 |
| *Lithobates catesbeianus* | -122.057 | 47.1427 |
| *Lithobates catesbeianus* | -95.642 | 38.94081 |
| *Lithobates catesbeianus* | -73.3806 | 41.1789 |
| *Lithobates catesbeianus* | -105.173 | 40.20507 |
| *Lithobates catesbeianus* | -71.2481 | 42.2333 |
| *Lithobates catesbeianus* | -97.3487 | 30.31297 |
| *Lithobates catesbeianus* | -74.6258 | 40.82357 |
| *Lithobates catesbeianus* | -94.7368 | 37.02432 |
| *Lithobates catesbeianus* | -121.679 | 37.26521 |
| *Lithobates catesbeianus* | -121.043 | 38.89224 |
| *Lithobates catesbeianus* | -121.722 | 39.03509 |
| *Lithobates catesbeianus* | -118.411 | 34.11959 |
| *Lithobates catesbeianus* | -71.7606 | 42.1939 |
| *Lithobates catesbeianus* | -70.8436 | 42.6361 |
| *Lithobates catesbeianus* | -122.419 | 37.60491 |
| *Lithobates catesbeianus* | -97.3987 | 32.3326 |
| *Lithobates catesbeianus* | 7.7878 | 44.93561 |
| *Lithobates catesbeianus* | -94.9414 | 38.80203 |
| *Lithobates catesbeianus* | -95.9715 | 30.24031 |
| *Lithobates catesbeianus* | -108.532 | 33.04488 |
| *Lithobates catesbeianus* | -94.3912 | 29.57317 |
| *Lithobates catesbeianus* | -123.096 | 48.50955 |
| *Lithobates catesbeianus* | -95.0493 | 31.18746 |
| *Lithobates catesbeianus* | -95.8971 | 41.0079 |
| *Lithobates catesbeianus* | -107.923 | 32.55194 |
| *Lithobates catesbeianus* | -123.087 | 45.50997 |
| *Lithobates catesbeianus* | -97.0466 | 33.12323 |
| *Lithobates catesbeianus* | 127.2612 | 36.15645 |
| *Lithobates catesbeianus* | -97.1697 | 30.08793 |
| *Lithobates catesbeianus* | -81.3014 | 41.43151 |
| *Lithobates catesbeianus* | -70.3048 | 43.68415 |
| *Lithobates catesbeianus* | -102.124 | 31.96724 |
| *Lithobates catesbeianus* | -99.2786 | 34.06881 |
| *Lithobates catesbeianus* | -122.487 | 47.44737 |
| *Lithobates catesbeianus* | -73.2773 | 44.22647 |
| *Lithobates catesbeianus* | -77.0366 | 39.05942 |
| *Lithobates catesbeianus* | -97.0269 | 33.0344 |
| *Lithobates catesbeianus* | -72.3077 | 44.29373 |
| *Lithobates catesbeianus* | -105.084 | 40.58526 |
| *Lithobates catesbeianus* | -97.0266 | 30.38318 |
| *Lithobates catesbeianus* | -97.1698 | 30.08756 |
| *Lithobates catesbeianus* | -88.1777 | 41.34508 |
| *Lithobates catesbeianus* | -71.075 | 42.05559 |
| *Lithobates catesbeianus* | -122.488 | 47.44734 |
| *Lithobates catesbeianus* | -97.7004 | 29.44309 |
| *Lithobates catesbeianus* | -75.4981 | 45.50157 |
| *Lithobates catesbeianus* | -71.5458 | 42.84054 |
| *Lithobates catesbeianus* | -88.1854 | 41.79609 |
| *Lithobates catesbeianus* | -72.608 | 41.7587 |
| *Lithobates catesbeianus* | -72.1979 | 41.83094 |
| *Lithobates catesbeianus* | -82.7347 | 42.16394 |
| *Lithobates catesbeianus* | -95.5361 | 30.53018 |
| *Lithobates catesbeianus* | -96.0958 | 28.65673 |
| *Lithobates catesbeianus* | -71.2066 | 42.41613 |
| *Lithobates catesbeianus* | -95.452 | 29.76485 |
| *Lithobates catesbeianus* | -118.201 | 34.0977 |
| *Lithobates catesbeianus* | -123.244 | 49.2536 |
| *Lithobates catesbeianus* | -75.9007 | 44.35119 |
| *Lithobates catesbeianus* | -75.9901 | 43.24617 |
| *Lithobates catesbeianus* | -73.7336 | 43.62957 |
| *Lithobates catesbeianus* | -91.1224 | 30.36267 |
| *Lithobates catesbeianus* | -64.9615 | 45.5881 |
| *Lithobates catesbeianus* | -121.821 | 37.50394 |
| *Lithobates catesbeianus* | -94.8338 | 35.67791 |
| *Lithobates catesbeianus* | -97.934 | 29.59019 |
| *Lithobates catesbeianus* | -74.884 | 39.00049 |
| *Lithobates catesbeianus* | -82.8 | 39.75 |
| *Lithobates catesbeianus* | -76.2502 | 4.18362 |
| *Lithobates catesbeianus* | -100.927 | 40.58485 |
| *Lithobates catesbeianus* | -73.5489 | 41.1328 |
| *Lithobates catesbeianus* | -72.7005 | 41.3348 |
| *Lithobates catesbeianus* | -73.2925 | 41.2018 |
| *Lithobates catesbeianus* | -73.2129 | 41.72236 |
| *Lithobates catesbeianus* | -72.546 | 41.46757 |
| *Lithobates catesbeianus* | -94.8278 | 36.4608 |
| *Lithobates catesbeianus* | -72.6437 | 41.34325 |
| *Lithobates catesbeianus* | -84.726 | 40.12083 |
| *Lithobates catesbeianus* | -82.0979 | 39.43956 |
| *Lithobates catesbeianus* | -84.0154 | 39.65836 |
| *Lithobates catesbeianus* | -82.0296 | 40.50903 |
| *Lithobates catesbeianus* | -83.0881 | 39.13556 |
| *Lithobates catesbeianus* | -84.6951 | 39.30422 |
| *Lithobates catesbeianus* | -83.85 | 38.06627 |
| *Lithobates catesbeianus* | -84.5445 | 41.38277 |
| *Lithobates catesbeianus* | -82.4689 | 39.08767 |
| *Lithobates catesbeianus* | -84.3908 | 40.59022 |
| *Lithobates catesbeianus* | -84.0627 | 39.40797 |
| *Lithobates catesbeianus* | -83.4543 | 40.23893 |
| *Lithobates catesbeianus* | -82.8465 | 38.90253 |
| *Lithobates catesbeianus* | -115.901 | 36.06811 |
| *Lithobates catesbeianus* | -117.14 | 32.77333 |
| *Lithobates catesbeianus* | -116.82 | 33.11238 |
| *Lithobates catesbeianus* | -116.976 | 33.4944 |
| *Lithobates catesbeianus* | -95.3586 | 34.9132 |
| *Lithobates catesbeianus* | -95.3072 | 34.9915 |
| *Lithobates catesbeianus* | -94.8331 | 34.8985 |
| *Lithobates catesbeianus* | -98.3701 | 34.76797 |
| *Lithobates catesbeianus* | -94.9653 | 34.4249 |
| *Lithobates catesbeianus* | -96.1823 | 34.50509 |
| *Lithobates catesbeianus* | -94.6115 | 35.92048 |
| *Lithobates catesbeianus* | -94.7003 | 36.19638 |
| *Lithobates catesbeianus* | -97.3628 | 35.64546 |
| *Lithobates catesbeianus* | -82.1738 | 28.8455 |
| *Lithobates catesbeianus* | -79.3015 | 35.1734 |
| *Lithobates catesbeianus* | -78.4564 | 36.1031 |
| *Lithobates catesbeianus* | -78.5883 | 35.302 |
| *Lithobates catesbeianus* | -75.6684 | 35.9875 |
| *Lithobates catesbeianus* | -77.9563 | 34.0234 |
| *Lithobates catesbeianus* | -78.5418 | 33.8905 |
| *Lithobates catesbeianus* | -78.4484 | 34.6624 |
| *Lithobates catesbeianus* | -79.071 | 34.4918 |
| *Lithobates catesbeianus* | -78.2335 | 34.0091 |
| *Lithobates catesbeianus* | -78.0663 | 33.9071 |
| *Lithobates catesbeianus* | -79.0062 | 36.0089 |
| *Lithobates catesbeianus* | -81.645 | 36.2412 |
| *Lithobates catesbeianus* | -74.2914 | 39.7301 |
| *Lithobates catesbeianus* | -79.6538 | 35.8631 |
| *Lithobates catesbeianus* | -77.5417 | 37.6858 |
| *Lithobates catesbeianus* | -78.4734 | 37.0408 |
| *Lithobates catesbeianus* | -76.1712 | 35.7304 |
| *Lithobates catesbeianus* | -76.3907 | 37.7299 |
| *Lithobates catesbeianus* | -90.1174 | 29.78276 |
| *Lithobates catesbeianus* | -155.605 | 20.116 |
| *Lithobates catesbeianus* | -76.1591 | 39.6226 |
| *Lithobates catesbeianus* | -83.2377 | 42.31642 |
| *Lithobates catesbeianus* | -81.5171 | 40.0937 |
| *Lithobates catesbeianus* | -73.4353 | 44.18389 |
| *Lithobates catesbeianus* | -81.2736 | 39.60852 |
| *Lithobates catesbeianus* | -73.9258 | 42.80087 |
| *Lithobates catesbeianus* | -89.9355 | 46.47601 |
| *Lithobates catesbeianus* | -82.29 | 37.2 |
| *Lithobates catesbeianus* | -71.0822 | 42.6125 |
| *Lithobates catesbeianus* | -81.0848 | 36.9484 |
| *Lithobates catesbeianus* | -98.9061 | 43.12805 |
| *Lithobates catesbeianus* | -106.2 | 31.55019 |
| *Lithobates catesbeianus* | -73.9466 | 41.38365 |
| *Lithobates catesbeianus* | -77.6906 | 37.5595 |
| *Lithobates catesbeianus* | -78.5747 | 38.69796 |
| *Lithobates catesbeianus* | -83.4447 | 31.7225 |
| *Lithobates catesbeianus* | -94.655 | 37.0743 |
| *Lithobates catesbeianus* | -99.8671 | 37.3974 |
| *Lithobates catesbeianus* | -97.1353 | 39.92998 |
| *Lithobates catesbeianus* | -75.6864 | 5.24153 |
| *Lithobates catesbeianus* | -77.2593 | 37.2875 |
| *Lithobates catesbeianus* | -77.6678 | 45.88444 |
| *Lithobates catesbeianus* | -117.356 | 33.16986 |
| *Lithobates catesbeianus* | 5.12605 | 51.1455 |
| *Lithobates catesbeianus* | 5.12989 | 51.14268 |
| *Lithobates catesbeianus* | 5.12873 | 51.1441 |
| *Lithobates catesbeianus* | 5.12885 | 51.14409 |
| *Lithobates catesbeianus* | 5.12898 | 51.14384 |
| *Lithobates catesbeianus* | -97.1629 | 30.26067 |
| *Lithobates catesbeianus* | -77.1126 | 38.75453 |
| *Lithobates catesbeianus* | -83.4313 | 39.0237 |
| *Lithobates catesbeianus* | -83.1454 | 39.0854 |
| *Lithobates catesbeianus* | -83.0669 | 39.0774 |
| *Lithobates catesbeianus* | -76.75 | 39.0004 |
| *Lithobates catesbeianus* | -80.18 | 40.45 |
| *Lithobates catesbeianus* | -82.42 | 35.61 |
| *Lithobates catesbeianus* | -82.69 | 35.14 |
| *Lithobates catesbeianus* | -79.24 | 40.14 |
| *Lithobates catesbeianus* | -92.65 | 32.29 |
| *Lithobates catesbeianus* | -94.8388 | 38.26545 |
| *Lithobates catesbeianus* | -89.3387 | 36.53384 |
| *Lithobates catesbeianus* | -79.94 | 40.46 |
| *Lithobates catesbeianus* | -76.59 | 37.5 |
| *Lithobates catesbeianus* | -77.87 | 36.92 |
| *Lithobates catesbeianus* | -76.74 | 36.75 |
| *Lithobates catesbeianus* | -76.01 | 36.64 |
| *Lithobates catesbeianus* | -76.12 | 36.76 |
| *Lithobates catesbeianus* | -76.45 | 37.11 |
| *Lithobates catesbeianus* | -78.783 | 43.867 |
| *Lithobates catesbeianus* | -76.25 | 45.05 |
| *Lithobates catesbeianus* | -75.95 | 45.367 |
| *Lithobates catesbeianus* | -77.193 | 43.976 |
| *Lithobates catesbeianus* | -76.068 | 45.591 |
| *Lithobates catesbeianus* | -75.932 | 45.636 |
| *Lithobates catesbeianus* | -80.417 | 44.567 |
| *Lithobates catesbeianus* | -67.133 | 45.167 |
| *Lithobates catesbeianus* | -66.063 | 44.128 |
| *Lithobates catesbeianus* | -75.776 | 46.165 |
| *Lithobates catesbeianus* | -76.65 | 45.017 |
| *Lithobates catesbeianus* | -76.208 | 44.556 |
| *Lithobates catesbeianus* | -76.75 | 45.422 |
| *Lithobates catesbeianus* | -75.709 | 45.26 |
| *Lithobates catesbeianus* | -76.193 | 45.903 |
| *Lithobates catesbeianus* | -71.45 | 42.433 |
| *Lithobates catesbeianus* | -67.083 | 45.233 |
| *Lithobates catesbeianus* | -76.65 | 45.483 |
| *Lithobates catesbeianus* | -75.717 | 45.7 |
| *Lithobates catesbeianus* | -65.217 | 44.383 |
| *Lithobates catesbeianus* | -106.591 | 31.87613 |
| *Lithobates catesbeianus* | -94.6602 | 29.6283 |
| *Lithobates catesbeianus* | -98.5464 | 28.53299 |
| *Lithobates catesbeianus* | -120.555 | 34.6777 |
| *Lithobates catesbeianus* | -109.963 | 34.17278 |
| *Lithobates catesbeianus* | -121.567 | 36.94709 |
| *Lithobates catesbeianus* | -121.748 | 37.19165 |
| *Lithobates catesbeianus* | -94.7264 | 31.47166 |
| *Lithobates catesbeianus* | -95.9441 | 30.85681 |
| *Lithobates catesbeianus* | -96.3344 | 30.64583 |
| *Lithobates catesbeianus* | -97.5677 | 30.07397 |
| *Lithobates catesbeianus* | -94.1146 | 29.76398 |
| *Lithobates catesbeianus* | -114.087 | 36.79172 |
| *Lithobates catesbeianus* | -105.06 | 39.95214 |
| *Lithobates catesbeianus* | -118.611 | 35.55745 |
| *Lithobates catesbeianus* | -124.108 | 42.02011 |
| *Lithobates catesbeianus* | -117.79 | 33.85901 |
| *Lithobates catesbeianus* | -118.214 | 34.08606 |
| *Lithobates catesbeianus* | -118.702 | 34.0807 |
| *Lithobates catesbeianus* | -117.579 | 33.38369 |
| *Lithobates catesbeianus* | -95.2236 | 38.92657 |
| *Lithobates catesbeianus* | -119.878 | 34.55583 |
| *Lithobates catesbeianus* | -120.613 | 35.39361 |
| *Lithobates catesbeianus* | -97.8324 | 37.38611 |
| *Lithobates catesbeianus* | -100.917 | 38.68385 |
| *Lithobates catesbeianus* | -97.3778 | 38.36915 |
| *Lithobates catesbeianus* | -97.7618 | 38.77025 |
| *Lithobates catesbeianus* | -97.2599 | 38.94745 |
| *Lithobates catesbeianus* | -96.1948 | 38.86165 |
| *Lithobates catesbeianus* | -96.4369 | 39.12387 |
| *Lithobates catesbeianus* | -100.112 | 38.97746 |
| *Lithobates catesbeianus* | -84.1789 | 30.07494 |
| *Lithobates catesbeianus* | -82.0822 | 27.5917 |
| *Lithobates catesbeianus* | -84.2454 | 30.47981 |
| *Lithobates catesbeianus* | -83.4956 | 30.55282 |
| *Lithobates catesbeianus* | -81.9908 | 29.71969 |
| *Lithobates catesbeianus* | -109.265 | 31.33994 |
| *Lithobates catesbeianus* | -120.071 | 34.64938 |
| *Lithobates catesbeianus* | -119.386 | 46.83 |
| *Lithobates catesbeianus* | -114.464 | 32.9728 |
| *Lithobates catesbeianus* | -114.494 | 32.8231 |
| *Lithobates catesbeianus* | -78.8386 | 42.8371 |
| *Lithobates catesbeianus* | -81.0139 | 41.8501 |
| *Lithobates catesbeianus* | -83.389 | 41.6823 |
| *Lithobates catesbeianus* | -76.396 | 44.6117 |
| *Lithobates catesbeianus* | -78.7948 | 43.8702 |
| *Lithobates catesbeianus* | -103.801 | 40.2704 |
| *Lithobates catesbeianus* | -105.238 | 40.16898 |
| *Lithobates catesbeianus* | -78.7139 | 35.81147 |
| *Lithobates catesbeianus* | -97.1699 | 30.08802 |
| *Lithobates catesbeianus* | -118.428 | 33.96658 |
| *Lithobates catesbeianus* | -76.6074 | 42.41001 |
| *Lithobates catesbeianus* | -81.4911 | 45.97113 |
| *Lithobates catesbeianus* | -83.9821 | 40.19708 |
| *Lithobates catesbeianus* | -95.6823 | 31.66874 |
| *Lithobates catesbeianus* | -93.0746 | 36.56668 |
| *Lithobates catesbeianus* | -76.6357 | 35.46448 |
| *Lithobates catesbeianus* | -120.733 | 41.89236 |
| *Lithobates catesbeianus* | -120.842 | 38.64525 |
| *Lithobates catesbeianus* | -115.224 | 37.53159 |
| *Lithobates catesbeianus* | -120.233 | 37.35862 |
| *Lithobates catesbeianus* | -77.1902 | 40.207 |
| *Lithobates catesbeianus* | -70.6665 | 41.8311 |
| *Lithobates catesbeianus* | -69.6467 | 46.14333 |
| *Lithobates catesbeianus* | -71.5363 | 41.8826 |
| *Lithobates catesbeianus* | -76.5959 | 39.24386 |
| *Lithobates catesbeianus* | -97.1172 | 39.38633 |
| *Lithobates catesbeianus* | -71.9398 | 43.41066 |
| *Lithobates catesbeianus* | -121.896 | 36.94596 |
| *Lithobates catesbeianus* | -97.8067 | 30.24205 |
| *Lithobates catesbeianus* | -118.728 | 34.09881 |
| *Lithobates catesbeianus* | -123.409 | 46.14783 |
| *Lithobates catesbeianus* | -97.4863 | 35.63776 |
| *Lithobates catesbeianus* | -122.025 | 38.49043 |
| *Lithobates catesbeianus* | -97.5823 | 29.58774 |
| *Lithobates catesbeianus* | -122.488 | 47.44725 |
| *Lithobates catesbeianus* | -81.4657 | 29.17073 |
| *Lithobates catesbeianus* | -83.3511 | 33.88733 |
| *Lithobates catesbeianus* | -71.0396 | 42.23383 |
| *Lithobates catesbeianus* | -71.0397 | 42.23387 |
| *Lithobates catesbeianus* | -73.0066 | 44.35122 |
| *Lithobates catesbeianus* | -97.4762 | 35.59513 |
| *Lithobates catesbeianus* | -122.634 | 38.08016 |
| *Lithobates catesbeianus* | -77.1315 | 38.83208 |
| *Lithobates catesbeianus* | -98.9422 | 33.85336 |
| *Lithobates catesbeianus* | -122.654 | 38.453 |
| *Lithobates catesbeianus* | -83.109 | 40.12179 |
| *Lithobates catesbeianus* | -121.845 | 39.72379 |
| *Lithobates catesbeianus* | -97.4585 | 30.14075 |
| *Lithobates catesbeianus* | -106.851 | 34.3037 |
| *Lithobates catesbeianus* | -117.6 | 33.92682 |
| *Lithobates catesbeianus* | -96.299 | 31.16907 |
| *Lithobates catesbeianus* | -120.057 | 38.93527 |
| *Lithobates catesbeianus* | -94.5545 | 29.60767 |
| *Lithobates catesbeianus* | -96.9551 | 33.06684 |
| *Lithobates catesbeianus* | -73.0963 | 44.53566 |
| *Lithobates catesbeianus* | -76.9415 | 38.91265 |
| *Lithobates catesbeianus* | -90.814 | 38.47766 |
| *Lithobates catesbeianus* | -84.8978 | 37.54376 |
| *Lithobates catesbeianus* | -74.4547 | 40.40912 |
| *Lithobates catesbeianus* | -96.2303 | 29.72674 |
| *Lithobates catesbeianus* | -122.817 | 38.08537 |
| *Lithobates catesbeianus* | -121.802 | 37.39687 |
| *Lithobates catesbeianus* | -118.957 | 34.16845 |
| *Lithobates catesbeianus* | -75.486 | 45.50019 |
| *Lithobates catesbeianus* | 126.343 | 36.65603 |
| *Lithobates catesbeianus* | -77.2544 | 38.9987 |
| *Lithobates catesbeianus* | -76.514 | 42.44377 |
| *Lithobates catesbeianus* | -76.27 | 42.13311 |
| *Lithobates catesbeianus* | -73.1345 | 41.7052 |
| *Lithobates catesbeianus* | -72.8156 | 41.2667 |
| *Lithobates catesbeianus* | -97.1078 | 32.75 |
| *Lithobates catesbeianus* | -97.6441 | 30.6834 |
| *Lithobates catesbeianus* | -82.6369 | 41.82639 |
| *Lithobates catesbeianus* | -78.3472 | 45.62472 |
| *Lithobates catesbeianus* | -82.4101 | 42.47185 |
| *Lithobates catesbeianus* | -116.4 | 36.4 |
| *Lithobates catesbeianus* | -85.1948 | 30.75331 |
| *Lithobates catesbeianus* | -84.3074 | 30.35257 |
| *Lithobates catesbeianus* | -82.1631 | 28.80806 |
| *Lithobates catesbeianus* | -82.3485 | 29.67706 |
| *Lithobates catesbeianus* | -84.3653 | 30.32222 |
| *Lithobates catesbeianus* | -90.1155 | 30.43887 |
| *Lithobates catesbeianus* | -83.0447 | 30.07781 |
| *Lithobates catesbeianus* | -83.3809 | 29.55784 |
| *Lithobates catesbeianus* | -84.2135 | 30.14992 |
| *Lithobates catesbeianus* | -82.2279 | 29.51559 |
| *Lithobates catesbeianus* | -77.1882 | 37.3731 |
| *Lithobates catesbeianus* | -78.2513 | 34.5552 |
| *Lithobates catesbeianus* | -122.198 | 37.84885 |
| *Lithobates catesbeianus* | 5.13046 | 51.14503 |
| *Lithobates catesbeianus* | 5.12903 | 51.14417 |
| *Lithobates catesbeianus* | -121.814 | 37.50543 |
| *Lithobates catesbeianus* | -76.2311 | 44.77145 |
| *Lithobates catesbeianus* | -76.4586 | 3.60408 |
| *Lithobates catesbeianus* | -116.713 | 33.1231 |
| *Lithobates catesbeianus* | -99.106 | 18.442 |
| *Lithobates catesbeianus* | -121.527 | 38.42051 |
| *Lithobates catesbeianus* | -121.254 | 37.45934 |
| *Lithobates catesbeianus* | -122.409 | 37.26672 |
| *Lithobates catesbeianus* | -121.825 | 36.4457 |
| *Lithobates catesbeianus* | -122.279 | 38.91015 |
| *Lithobates catesbeianus* | -121.784 | 37.70056 |
| *Lithobates catesbeianus* | -96.4603 | 30.54973 |
| *Lithobates catesbeianus* | -119.469 | 36.74994 |
| *Lithobates catesbeianus* | -100.103 | 39.11774 |
| *Lithobates catesbeianus* | -95.7301 | 39.0547 |
| *Lithobates catesbeianus* | -94.7723 | 39.17109 |
| *Lithobates catesbeianus* | -97.0768 | 38.31581 |
| *Lithobates catesbeianus* | -92.8151 | 42.67226 |
| *Lithobates catesbeianus* | 138.886 | 36.2308 |
| *Lithobates catesbeianus* | -75.6433 | 5.73417 |
| *Lithobates catesbeianus* | -72.783 | 47.433 |
| *Lithobates catesbeianus* | -75.75 | 45.4 |
| *Lithobates catesbeianus* | -75.908 | 45.133 |
| *Lithobates catesbeianus* | -76.015 | 45.642 |
| *Lithobates catesbeianus* | -76.067 | 45.597 |
| *Lithobates catesbeianus* | -75.9 | 45.367 |
| *Lithobates catesbeianus* | -76.342 | 46.306 |
| *Lithobates catesbeianus* | -75.75 | 45.367 |
| *Lithobates catesbeianus* | -82.672 | 41.745 |
| *Lithobates catesbeianus* | -79.383 | 46.303 |
| *Lithobates catesbeianus* | -75.873 | 45.501 |
| *Lithobates catesbeianus* | -71.783 | 42.367 |
| *Lithobates catesbeianus* | -72.033 | 42.35 |
| *Lithobates catesbeianus* | -80.033 | 45.3 |
| *Lithobates catesbeianus* | -76.15 | 45.083 |
| *Lithobates catesbeianus* | -77.267 | 44.517 |
| *Lithobates catesbeianus* | -65.867 | 45.35 |
| *Lithobates catesbeianus* | -78.583 | 45.55 |
| *Lithobates catesbeianus* | -122.514 | 47.0318 |
| *Lithobates catesbeianus* | -76.0858 | 43.2781 |
| *Lithobates catesbeianus* | -83.8068 | 39.37778 |
| *Lithobates catesbeianus* | -84.3756 | 41.55052 |
| *Lithobates catesbeianus* | -83.6097 | 38.80323 |
| *Lithobates catesbeianus* | -84.4316 | 39.54686 |
| *Lithobates catesbeianus* | -84.1891 | 40.68644 |
| *Lithobates catesbeianus* | -94.637 | 37.0453 |
| *Lithobates catesbeianus* | -94.6318 | 37.12896 |
| *Lithobates catesbeianus* | -101.371 | 37.5858 |
| *Lithobates catesbeianus* | -94.6993 | 37.03765 |
| *Lithobates catesbeianus* | -80.0003 | 35.5007 |
| *Lithobates catesbeianus* | -83.006 | 40.0049 |
| *Lithobates catesbeianus* | -83.5435 | 41.6249 |
| *Lithobates catesbeianus* | -74.65 | 40.34 |
| *Lithobates catesbeianus* | -75.87 | 41.96 |
| *Lithobates catesbeianus* | -78.39 | 40.46 |
| *Lithobates catesbeianus* | -81.5497 | 37.57003 |
| *Lithobates catesbeianus* | -82.2097 | 38.47001 |
| *Lithobates catesbeianus* | -121.061 | 39.22982 |
| *Lithobates catesbeianus* | -74.9 | 39.78 |
| *Lithobates catesbeianus* | -78.21 | 41.97 |
| *Lithobates catesbeianus* | -79.55 | 41.98 |
| *Lithobates catesbeianus* | -89.13 | 39.15 |
| *Lithobates catesbeianus* | -95.2301 | 38.91998 |
| *Lithobates catesbeianus* | -76.08 | 38.94 |
| *Lithobates catesbeianus* | -76.59 | 36.58 |
| *Lithobates catesbeianus* | -76.7 | 36.69 |
| *Lithobates catesbeianus* | -108.205 | 33.17904 |
| *Lithobates catesbeianus* | -111.143 | 31.39547 |
| *Lithobates catesbeianus* | -116.313 | 33.83066 |
| *Lithobates catesbeianus* | -114.742 | 33.4316 |
| *Lithobates catesbeianus* | -113.584 | 37.10415 |
| *Lithobates catesbeianus* | -117.31 | 33.37646 |
| *Lithobates catesbeianus* | -117.061 | 35.73873 |
| *Lithobates catesbeianus* | -155.094 | 19.70983 |
| *Lithobates catesbeianus* | -156.912 | 20.81678 |
| *Lithobates catesbeianus* | -123.21 | 44.69929 |
| *Lithobates catesbeianus* | -118.795 | 45.60541 |
| *Lithobates catesbeianus* | -122.757 | 45.8089 |
| *Lithobates catesbeianus* | -111.518 | 34.84333 |
| *Lithobates catesbeianus* | -92.4046 | 47.36915 |
| *Lithobates catesbeianus* | -93.216 | 44.02961 |
| *Lithobates catesbeianus* | -95.0427 | 43.3491 |
| *Lithobates catesbeianus* | -105.245 | 40.15937 |
| *Lithobates catesbeianus* | -105.872 | 37.48011 |
| *Lithobates catesbeianus* | -105.093 | 39.55845 |
| *Lithobates catesbeianus* | -96.4122 | 30.6053 |
| *Lithobates catesbeianus* | -95.9301 | 29.92591 |
| *Lithobates catesbeianus* | -96.3344 | 30.65694 |
| *Lithobates catesbeianus* | -96.7043 | 30.79722 |
| *Lithobates catesbeianus* | -95.9428 | 30.01091 |
| *Lithobates catesbeianus* | -98.4751 | 34.05687 |
| *Lithobates catesbeianus* | -97.6853 | 28.89556 |
| *Lithobates catesbeianus* | -96.2969 | 30.635 |
| *Lithobates catesbeianus* | -98.3719 | 30.25583 |
| *Lithobates catesbeianus* | -67.1892 | 44.84555 |
| *Lithobates catesbeianus* | -77.7028 | 45.88556 |
| *Lithobates catesbeianus* | -76.8167 | 45.08333 |
| *Lithobates catesbeianus* | -70.6155 | 41.42167 |
| *Lithobates catesbeianus* | -123.097 | 38.53002 |
| *Lithobates catesbeianus* | -116.577 | 32.98556 |
| *Lithobates catesbeianus* | -94.6141 | 33.92831 |
| *Lithobates catesbeianus* | -100.014 | 35.91385 |
| *Lithobates catesbeianus* | -97.1315 | 34.17599 |
| *Lithobates catesbeianus* | -97.1436 | 34.0426 |
| *Lithobates catesbeianus* | -95.2652 | 34.9768 |
| *Lithobates catesbeianus* | -95.2978 | 34.942 |
| *Lithobates catesbeianus* | -98.3684 | 34.28914 |
| *Lithobates catesbeianus* | -94.67 | 34.4268 |
| *Lithobates catesbeianus* | -94.5525 | 33.87701 |
| *Lithobates catesbeianus* | -98.182 | 36.83863 |
| *Lithobates catesbeianus* | -97.8443 | 33.7984 |
| *Lithobates catesbeianus* | -94.5396 | 36.18818 |
| *Lithobates catesbeianus* | -94.3893 | 35.38593 |
| *Lithobates catesbeianus* | -97.177 | 35.21757 |
| *Lithobates catesbeianus* | -106.657 | 35.02901 |
| *Lithobates catesbeianus* | -94.5937 | 36.08028 |
| *Lithobates catesbeianus* | -100.494 | 36.75253 |
| *Lithobates catesbeianus* | -94.6412 | 36.54568 |
| *Lithobates catesbeianus* | -95.1617 | 35.75467 |
| *Lithobates catesbeianus* | -78.6387 | 35.6335 |
| *Lithobates catesbeianus* | -76.8044 | 34.966 |
| *Lithobates catesbeianus* | -78.0835 | 34.1203 |
| *Lithobates catesbeianus* | -79.7382 | 34.917 |
| *Lithobates catesbeianus* | -81.4901 | 33.549 |
| *Lithobates catesbeianus* | -157.807 | 21.3124 |
| *Lithobates catesbeianus* | -81.9056 | 32.74 |
| *Lithobates catesbeianus* | -73.5908 | 44.21611 |
| *Lithobates catesbeianus* | -100.271 | 36.23289 |
| *Lithobates catesbeianus* | -94.58 | 37.2458 |
| *Lithobates catesbeianus* | -95.1954 | 37.34001 |
| *Lithobates catesbeianus* | -99.6749 | 33.94339 |
| *Lithobates catesbeianus* | -107.276 | 33.08794 |
| *Lithobates catesbeianus* | -95.1763 | 36.62692 |
| *Lithobates catesbeianus* | -78.21 | 39.36 |
| *Lithobates catesbeianus* | -80.0222 | 41.47463 |
| *Lithobates catesbeianus* | -82.6159 | 36.9497 |
| *Lithobates catesbeianus* | -77.1 | 34.93 |
| *Lithobates catesbeianus* | -77.343 | 39.1284 |
| *Lithobates catesbeianus* | -72.6548 | 41.5475 |
| *Lithobates catesbeianus* | -77.55 | 37.7 |
| *Lithobates catesbeianus* | -79.7453 | 36.811 |
| *Lithobates catesbeianus* | -75.68 | 37.68 |
| *Lithobates catesbeianus* | -82.5543 | 36.6817 |
| *Lithobates catesbeianus* | -122.98 | 43.94272 |
| *Lithobates catesbeianus* | -77.9784 | 34.8948 |
| *Lithobates catesbeianus* | -82.301 | 35.9024 |
| *Lithobates catesbeianus* | -80.4111 | 36.5432 |
| *Lithobates catesbeianus* | -78.5293 | 34.6406 |
| *Lithobates catesbeianus* | -81.9467 | 35.9856 |
| *Lithobates catesbeianus* | -78.5272 | 34.166 |
| *Lithobates catesbeianus* | -81.8818 | 27.2238 |
| *Lithobates catesbeianus* | -81.4938 | 36.4856 |
| *Lithobates catesbeianus* | -74.5751 | 39.4784 |
| *Lithobates catesbeianus* | -75.4952 | 38.567 |
| *Lithobates catesbeianus* | -77.0602 | 34.7659 |
| *Lithobates catesbeianus* | -77.5019 | 37.6656 |
| *Lithobates catesbeianus* | -77.7333 | 36.9456 |
| *Lithobates catesbeianus* | -77.5162 | 37.6057 |
| *Lithobates catesbeianus* | -82.0141 | 34.1444 |
| *Lithobates catesbeianus* | -77.35 | 45.24 |
| *Lithobates catesbeianus* | -78.53 | 44.63 |
| *Lithobates catesbeianus* | -122.106 | 37.062 |
| *Lithobates catesbeianus* | -75.6241 | 35.95739 |
| *Lithobates catesbeianus* | -121.872 | 37.21744 |
| *Lithobates catesbeianus* | -117.772 | 33.81955 |
| *Lithobates catesbeianus* | -73.2685 | 43.63804 |
| *Lithobates catesbeianus* | -108.624 | 33.83118 |
| *Lithobates catesbeianus* | -122.605 | 37.95328 |
| *Lithobates catesbeianus* | -70.2394 | 43.70642 |
| *Lithobates catesbeianus* | -70.4976 | 43.92656 |
| *Lithobates catesbeianus* | -75.9394 | 38.95494 |
| *Lithobates catesbeianus* | -105.201 | 39.96046 |
| *Lithobates catesbeianus* | -103.695 | 41.64103 |
| *Lithobates catesbeianus* | -76.7543 | 37.38558 |
| *Lithobates catesbeianus* | -123.743 | 39.23957 |
| *Lithobates catesbeianus* | -82.2584 | 28.1158 |
| *Lithobates catesbeianus* | -66.252 | 18.19414 |
| *Lithobates catesbeianus* | -122.25 | 37.89602 |
| *Lithobates catesbeianus* | -87.6507 | 33.14703 |
| *Lithobates catesbeianus* | -96.9551 | 33.06813 |
| *Lithobates catesbeianus* | -77.6607 | 45.88738 |
| *Lithobates catesbeianus* | -97.1478 | 32.84849 |
| *Lithobates catesbeianus* | -72.4689 | 42.98983 |
| *Lithobates catesbeianus* | -98.5552 | 33.84991 |
| *Lithobates catesbeianus* | -121.238 | 37.47503 |
| *Lithobates catesbeianus* | 128.397 | 35.61928 |
| *Lithobates catesbeianus* | -76.9476 | 38.91201 |
| *Lithobates catesbeianus* | -115.615 | 33.17752 |
| *Lithobates catesbeianus* | -97.397 | 32.33012 |
| *Lithobates catesbeianus* | -92.2109 | 38.89677 |
| *Lithobates catesbeianus* | -78.681 | 34.24939 |
| *Lithobates catesbeianus* | -95.8922 | 31.93987 |
| *Lithobates catesbeianus* | -79.4733 | 36.0381 |
| *Lithobates catesbeianus* | -96.6694 | 28.45413 |
| *Lithobates catesbeianus* | -123.867 | 46.15437 |
| *Lithobates catesbeianus* | -71.7565 | 42.67609 |
| *Lithobates catesbeianus* | -91.1102 | 40.80004 |
| *Lithobates catesbeianus* | -77.1222 | 38.8602 |
| *Lithobates catesbeianus* | -75.68 | 44.58845 |
| *Lithobates catesbeianus* | -74.6305 | 45.16267 |
| *Lithobates catesbeianus* | -92.6823 | 42.78715 |
| *Lithobates catesbeianus* | -76.4132 | 40.04205 |
| *Lithobates catesbeianus* | -96.9958 | 32.98129 |
| *Lithobates catesbeianus* | -93.6092 | 34.38829 |
| *Lithobates catesbeianus* | -76.7027 | 38.7847 |
| *Lithobates catesbeianus* | 126.1534 | 36.80383 |
| *Lithobates catesbeianus* | -83.9937 | 42.47068 |
| *Lithobates catesbeianus* | -96.729 | 33.20036 |
| *Lithobates catesbeianus* | -91.9477 | 35.45597 |
| *Lithobates catesbeianus* | -74.2605 | 40.58748 |
| *Lithobates catesbeianus* | -73.5051 | 41.15535 |
| *Lithobates catesbeianus* | -106.307 | 31.64272 |
| *Lithobates catesbeianus* | -96.8062 | 28.30155 |
| *Lithobates catesbeianus* | -85.188 | 45.35113 |
| *Lithobates catesbeianus* | 126.7161 | 35.53324 |
| *Lithobates catesbeianus* | -122.941 | 49.24461 |
| *Lithobates catesbeianus* | -73.5832 | 41.33208 |
| *Lithobates catesbeianus* | -122.583 | 38.22417 |
| *Lithobates catesbeianus* | -95.9924 | 31.81973 |
| *Lithobates catesbeianus* | -112.025 | 34.77054 |
| *Lithobates catesbeianus* | -82.6087 | 35.49764 |
| *Lithobates catesbeianus* | -81.1938 | 37.7842 |
| *Lithobates catesbeianus* | -74.3786 | 40.68637 |
| *Lithobates catesbeianus* | 4.75683 | 51.40573 |
| *Lithobates catesbeianus* | -94.3899 | 29.57409 |
| *Lithobates catesbeianus* | -81.9128 | 36.2814 |
| *Lithobates catesbeianus* | -77.6224 | 36.4347 |
| *Lithobates catesbeianus* | -84.158 | 30.4635 |
| *Lithobates catesbeianus* | -97.252 | 35.11775 |
| *Lithobates catesbeianus* | 5.12906 | 51.14437 |
| *Lithobates catesbeianus* | 5.01378 | 51.27079 |
| *Lithobates catesbeianus* | -122.667 | 37.95729 |
| *Lithobates catesbeianus* | -76.2061 | 4.28844 |
| *Lithobates catesbeianus* | -76.2303 | 4.23041 |
| *Lithobates catesbeianus* | -120.936 | 38.17774 |
| *Lithobates catesbeianus* | -97.7797 | 30.28592 |
| *Lithobates catesbeianus* | -81.1625 | 45.97337 |
| *Lithobates catesbeianus* | -116.946 | 33.039 |
| *Lithobates catesbeianus* | -77.6704 | 34.4286 |
| *Lithobates catesbeianus* | -76.5503 | 43.4299 |
| *Lithobates catesbeianus* | -96.5768 | 39.09414 |
| *Lithobates catesbeianus* | -95.2947 | 37.32006 |
| *Lithobates catesbeianus* | -94.8502 | 38.59368 |
| *Lithobates catesbeianus* | -77.1314 | 35.0279 |
| *Lithobates catesbeianus* | -96.5312 | 39.2308 |
| *Lithobates catesbeianus* | -94.922 | 38.96806 |
| *Lithobates catesbeianus* | -96.6126 | 38.88469 |
| *Lithobates catesbeianus* | -96.5198 | 38.95025 |
| *Lithobates catesbeianus* | 0.49374 | 50.84205 |
| *Lithobates catesbeianus* | -73.017 | 46.7 |
| *Lithobates catesbeianus* | -75.633 | 45.183 |
| *Lithobates catesbeianus* | -73.978 | 45.4 |
| *Lithobates catesbeianus* | -67.117 | 45.217 |
| *Lithobates catesbeianus* | -75.717 | 45.667 |
| *Lithobates catesbeianus* | -75.5 | 44.933 |
| *Lithobates catesbeianus* | -77.017 | 43.883 |
| *Lithobates catesbeianus* | -75.264 | 45.554 |
| *Lithobates catesbeianus* | -75.933 | 45.138 |
| *Lithobates catesbeianus* | -75.754 | 44.863 |
| *Lithobates catesbeianus* | -76.215 | 45.637 |
| *Lithobates catesbeianus* | -75.831 | 45.523 |
| *Lithobates catesbeianus* | -75.5 | 45.5 |
| *Lithobates catesbeianus* | -76.098 | 45.599 |
| *Lithobates catesbeianus* | -76.026 | 45.603 |
| *Lithobates catesbeianus* | -63.867 | 45.733 |
| *Lithobates catesbeianus* | -75.3 | 45.57 |
| *Lithobates catesbeianus* | -75.883 | 45.533 |
| *Lithobates catesbeianus* | -75.921 | 45.404 |
| *Lithobates catesbeianus* | -76 | 44.533 |
| *Lithobates catesbeianus* | -82.233 | 46.233 |
| *Lithobates catesbeianus* | -76.067 | 45.633 |
| *Lithobates catesbeianus* | -63.917 | 45.717 |
| *Lithobates catesbeianus* | -65.05 | 44.383 |
| *Lithobates catesbeianus* | -83.7303 | 33.4563 |
| *Lithobates catesbeianus* | -82.39 | 29.7 |
| *Lithobates catesbeianus* | -79.5196 | 39.45 |
| *Lithobates catesbeianus* | -78.31 | 42.05 |
| *Lithobates catesbeianus* | -80.12 | 40.25 |
| *Lithobates catesbeianus* | -79.49 | 40.58 |
| *Lithobates catesbeianus* | -111.94 | 33.43 |
| *Lithobates catesbeianus* | -69.91 | 44.29 |
| *Lithobates catesbeianus* | -81.61 | 36.97 |
| *Lithobates catesbeianus* | -76.55 | 37.18 |
| *Lithobates catesbeianus* | -116.779 | 33.2436 |
| *Lithobates catesbeianus* | -83.6115 | 40.99342 |
| *Lithobates catesbeianus* | -84.8047 | 39.58061 |
| *Lithobates catesbeianus* | -84.0121 | 41.11045 |
| *Lithobates catesbeianus* | -80.8396 | 39.73593 |
| *Lithobates catesbeianus* | -83.9974 | 39.23925 |
| *Lithobates catesbeianus* | -80.5573 | 41.5139 |
| *Lithobates catesbeianus* | -83.4608 | 40.2618 |
| *Lithobates catesbeianus* | -83.8074 | 39.01708 |
| *Lithobates catesbeianus* | -83.9627 | 39.58247 |
| *Lithobates catesbeianus* | -94.6382 | 37.0592 |
| *Lithobates catesbeianus* | -99.311 | 18.478 |
| *Lithobates catesbeianus* | 135.6647 | 34.82361 |
| *Lithobates catesbeianus* | -97.7599 | 21.10408 |
| *Lithobates catesbeianus* | -119.927 | 37.80761 |
| *Lithobates catesbeianus* | -121.015 | 39.97289 |
| *Lithobates catesbeianus* | -119.545 | 35.56186 |
| *Lithobates catesbeianus* | -77.0563 | 39.23836 |
| *Lithobates catesbeianus* | -121.29 | 37.04542 |
| *Lithobates catesbeianus* | -81.051 | 41.53508 |
| *Lithobates catesbeianus* | -100.272 | 35.45527 |
| *Lithobates catesbeianus* | -87.5407 | 30.76643 |
| *Lithobates catesbeianus* | -82.2053 | 29.5131 |
| *Lithobates catesbeianus* | -82.4815 | 27.881 |
| *Lithobates catesbeianus* | -84.3078 | 30.35279 |
| *Lithobates catesbeianus* | -87.3498 | 30.9338 |
| *Lithobates catesbeianus* | -86.2406 | 39.20967 |
| *Lithobates catesbeianus* | -106.231 | 31.5859 |
| *Lithobates catesbeianus* | -82.3224 | 40.2267 |
| *Lithobates catesbeianus* | -81.0001 | 34.0004 |
| *Lithobates catesbeianus* | -82.804 | 41.6902 |
| *Lithobates catesbeianus* | -84.4408 | 41.056 |
| *Lithobates catesbeianus* | -83.4501 | 39.2894 |
| *Lithobates catesbeianus* | -80.9782 | 41.1884 |
| *Lithobates catesbeianus* | -120.555 | 34.67767 |
| *Lithobates catesbeianus* | -122.401 | 45.56818 |
| *Lithobates catesbeianus* | -119.574 | 44.81733 |
| *Lithobates catesbeianus* | -116.158 | 35.87774 |
| *Lithobates catesbeianus* | -110.635 | 32.71393 |
| *Lithobates catesbeianus* | -109.239 | 31.59466 |
| *Lithobates catesbeianus* | -111.55 | 31.55 |
| *Lithobates catesbeianus* | -114.532 | 32.7777 |
| *Lithobates catesbeianus* | -111.228 | 34.7814 |
| *Lithobates catesbeianus* | -66.1453 | 18.44183 |
| *Lithobates catesbeianus* | -114.677 | 35.63916 |
| *Lithobates catesbeianus* | -117.597 | 33.52564 |
| *Lithobates catesbeianus* | -115.174 | 36.14896 |
| *Lithobates catesbeianus* | -118.088 | 33.79398 |
| *Lithobates catesbeianus* | -116.252 | 33.85011 |
| *Lithobates catesbeianus* | -117.947 | 36.53815 |
| *Lithobates catesbeianus* | -96.1702 | 32.395 |
| *Lithobates catesbeianus* | -101.148 | 33.54333 |
| *Lithobates catesbeianus* | -98.4022 | 33.57594 |
| *Lithobates catesbeianus* | -117.076 | 32.65427 |
| *Lithobates catesbeianus* | -119.301 | 34.48417 |
| *Lithobates catesbeianus* | -124.669 | 48.1531 |
| *Lithobates catesbeianus* | -74.4437 | 4.30823 |
| *Lithobates catesbeianus* | -71.844 | 41.797 |
| *Lithobates catesbeianus* | -73.2835 | 41.24628 |
| *Lithobates catesbeianus* | -95.3376 | 35.0257 |
| *Lithobates catesbeianus* | -90.2 | 38.63 |
| *Lithobates catesbeianus* | -77.7706 | 37.8664 |
| *Lithobates catesbeianus* | -82.5289 | 31.1243 |
| *Lithobates catesbeianus* | -99.7584 | 33.98783 |
| *Lithobates catesbeianus* | -76.8122 | 39.0568 |
| *Lithobates catesbeianus* | -79 | 37.98 |
| *Lithobates catesbeianus* | -77.16 | 38.63 |
| *Lithobates catesbeianus* | -109.762 | 32.01883 |
| *Lithobates catesbeianus* | -121.157 | 38.76247 |
| *Lithobates catesbeianus* | -77.2233 | 37.1507 |
| *Lithobates catesbeianus* | -77.58 | 37.67 |
| *Lithobates catesbeianus* | -82.843 | 36.919 |
| *Lithobates catesbeianus* | -79.87 | 37.89 |
| *Lithobates catesbeianus* | -76.43 | 36.81 |
| *Lithobates catesbeianus* | -96.5642 | 44.6967 |
| *Lithobates catesbeianus* | -76.9239 | 39.4108 |
| *Lithobates catesbeianus* | -94.6676 | 34.4412 |
| *Lithobates catesbeianus* | -96.9499 | 35.21677 |
| *Lithobates catesbeianus* | -102.657 | 36.90242 |
| *Lithobates catesbeianus* | -94.5716 | 36.10378 |
| *Lithobates catesbeianus* | -96.6377 | 34.44739 |
| *Lithobates catesbeianus* | -98.5671 | 34.65267 |
| *Lithobates catesbeianus* | -98.5695 | 34.71097 |
| *Lithobates catesbeianus* | -99.734 | 35.56155 |
| *Lithobates catesbeianus* | -94.9233 | 35.84929 |
| *Lithobates catesbeianus* | -96.9767 | 34.23809 |
| *Lithobates catesbeianus* | -94.8069 | 36.13829 |
| *Lithobates catesbeianus* | -94.533 | 35.72883 |
| *Lithobates catesbeianus* | -77.9215 | 34.127 |
| *Lithobates catesbeianus* | -78.3531 | 35.9318 |
| *Lithobates catesbeianus* | -82.2892 | 29.6522 |
| *Lithobates catesbeianus* | -78.6848 | 35.634 |
| *Lithobates catesbeianus* | -78.9689 | 36.0032 |
| *Lithobates catesbeianus* | -82.9969 | 35.223 |
| *Lithobates catesbeianus* | -75.4502 | 39.4616 |
| *Lithobates catesbeianus* | -81.0766 | 33.6609 |
| *Lithobates catesbeianus* | -80.844 | 37.4921 |
| *Lithobates catesbeianus* | -77.5664 | 37.6903 |
| *Lithobates catesbeianus* | -77.2049 | 37.5146 |
| *Lithobates catesbeianus* | -76.726 | 37.3878 |
| *Lithobates catesbeianus* | -80.8291 | 35.5107 |
| *Lithobates catesbeianus* | -79.8224 | 36.8964 |
| *Lithobates catesbeianus* | -80.4204 | 34.6735 |
| *Lithobates catesbeianus* | -80.333 | 45.4 |
| *Lithobates catesbeianus* | -88.9295 | 37.37708 |
| *Lithobates catesbeianus* | -76.7697 | 39.98143 |
| *Lithobates catesbeianus* | -103.554 | 29.32777 |
| *Lithobates catesbeianus* | -90.7395 | 38.71646 |
| *Lithobates catesbeianus* | -124.103 | 44.06901 |
| *Lithobates catesbeianus* | -122.161 | 37.92723 |
| *Lithobates catesbeianus* | -121.635 | 37.24676 |
| *Lithobates catesbeianus* | -95.255 | 30.93883 |
| *Lithobates catesbeianus* | -86.3593 | 41.54932 |
| *Lithobates catesbeianus* | -81.4561 | 41.0795 |
| *Lithobates catesbeianus* | -79.9061 | 40.54407 |
| *Lithobates catesbeianus* | -122.988 | 45.38306 |
| *Lithobates catesbeianus* | -77.7338 | 43.19659 |
| *Lithobates catesbeianus* | -122.864 | 42.26506 |
| *Lithobates catesbeianus* | -121.292 | 37.77788 |
| *Lithobates catesbeianus* | -120.453 | 37.85131 |
| *Lithobates catesbeianus* | -116.316 | 36.46969 |
| *Lithobates catesbeianus* | -122.98 | 38.44559 |
| *Lithobates catesbeianus* | -70.6694 | 41.51995 |
| *Lithobates catesbeianus* | -71.1611 | 42.625 |
| *Lithobates catesbeianus* | -74.4536 | 40.4486 |
| *Lithobates catesbeianus* | -71.0905 | 42.2351 |
| *Lithobates catesbeianus* | -97.0728 | 27.83472 |
| *Lithobates catesbeianus* | -78.3079 | 37.38511 |
| *Lithobates catesbeianus* | -71.6415 | 43.11375 |
| *Lithobates catesbeianus* | -74.453 | 40.41081 |
| *Lithobates catesbeianus* | -83.1909 | 41.62769 |
| *Lithobates catesbeianus* | -97.1898 | 29.72117 |
| *Lithobates catesbeianus* | -76.4629 | 44.43059 |
| *Lithobates catesbeianus* | -122.877 | 45.22419 |
| *Lithobates catesbeianus* | -71.48 | 42.93295 |
| *Lithobates catesbeianus* | -121.342 | 38.04688 |
| *Lithobates catesbeianus* | -121.678 | 37.2985 |
| *Lithobates catesbeianus* | -69.9958 | 41.88168 |
| *Lithobates catesbeianus* | -77.0569 | 38.77101 |
| *Lithobates catesbeianus* | -95.8585 | 29.97831 |
| *Lithobates catesbeianus* | -75.4923 | 45.50375 |
| *Lithobates catesbeianus* | -76.7898 | 39.15211 |
| *Lithobates catesbeianus* | -81.4412 | 41.27781 |
| *Lithobates catesbeianus* | -68.2079 | 44.3628 |
| *Lithobates catesbeianus* | -81.457 | 41.04226 |
| *Lithobates catesbeianus* | -94.556 | 29.6115 |
| *Lithobates catesbeianus* | -96.9337 | 32.77364 |
| *Lithobates catesbeianus* | -96.1678 | 30.72046 |
| *Lithobates catesbeianus* | -94.5546 | 29.60734 |
| *Lithobates catesbeianus* | -95.9377 | 33.59174 |
| *Lithobates catesbeianus* | -117.264 | 33.24053 |
| *Lithobates catesbeianus* | -71.1256 | 42.3747 |
| *Lithobates catesbeianus* | -71.5589 | 41.8139 |
| *Lithobates catesbeianus* | -69.415 | 44.28 |
| *Lithobates catesbeianus* | -72.3394 | 42.6819 |
| *Lithobates catesbeianus* | -117.729 | 33.62148 |
| *Lithobates catesbeianus* | -72.5043 | 44.33217 |
| *Lithobates catesbeianus* | -121.272 | 38.07898 |
| *Lithobates catesbeianus* | -120.935 | 37.6113 |
| *Lithobates catesbeianus* | -118.21 | 34.20152 |
| *Lithobates catesbeianus* | -72.6618 | 41.64905 |
| *Lithobates catesbeianus* | -122.817 | 38.08544 |
| *Lithobates catesbeianus* | -122.207 | 37.8592 |
| *Lithobates catesbeianus* | -96.8052 | 28.30465 |
| *Lithobates catesbeianus* | -97.4 | 29.2837 |
| *Lithobates catesbeianus* | -96.4088 | 30.51482 |
| *Lithobates catesbeianus* | -122.494 | 47.44728 |
| *Lithobates catesbeianus* | -73.2913 | 41.82028 |
| *Lithobates catesbeianus* | -97.6804 | 37.54021 |
| *Lithobates catesbeianus* | -95.1214 | 39.08927 |
| *Lithobates catesbeianus* | -74.9489 | 45.64424 |
| *Lithobates catesbeianus* | -77.2853 | 38.80851 |
| *Lithobates catesbeianus* | -68.2078 | 44.36287 |
| *Lithobates catesbeianus* | -71.1461 | 42.39619 |
| *Lithobates catesbeianus* | -82.8343 | 39.75921 |
| *Lithobates catesbeianus* | -75.5018 | 45.49903 |
| *Lithobates catesbeianus* | -96.9571 | 33.0678 |
| *Lithobates catesbeianus* | -72.3683 | 40.98947 |
| *Lithobates catesbeianus* | -97.4864 | 35.63745 |
| *Lithobates catesbeianus* | -80.2429 | 42.5613 |
| *Lithobates catesbeianus* | -76.7741 | 42.06478 |
| *Lithobates catesbeianus* | -75.1119 | 43.59111 |
| *Lithobates catesbeianus* | -88.8743 | 39.77145 |
| *Lithobates catesbeianus* | -79.8575 | 40.32049 |
| *Lithobates catesbeianus* | -77.47 | 37.59 |
| *Lithobates catesbeianus* | -85.1413 | 30.78966 |
| *Lithobates catesbeianus* | -82.7401 | 29.34848 |
| *Lithobates catesbeianus* | -85.8433 | 30.95025 |
| *Lithobates catesbeianus* | -83.9762 | 30.5259 |
| *Lithobates catesbeianus* | -82.535 | 29.08103 |
| *Lithobates catesbeianus* | -84.3558 | 30.52872 |
| *Lithobates catesbeianus* | -83.497 | 30.41406 |
| *Lithobates catesbeianus* | -81.9966 | 29.68369 |
| *Lithobates catesbeianus* | -120.467 | 39.71667 |
| *Lithobates catesbeianus* | -82.6123 | 35.7944 |
| *Lithobates catesbeianus* | -72.6065 | 43.17168 |
| *Lithobates catesbeianus* | -97.3904 | 35.2115 |
| *Lithobates catesbeianus* | 5.13004 | 51.1438 |
| *Lithobates catesbeianus* | -79.9117 | 40.44021 |
| *Lithobates catesbeianus* | -68.9264 | 45.3233 |
| *Lithobates catesbeianus* | -122.649 | 37.95749 |
| *Lithobates catesbeianus* | -75.9985 | 4.87757 |
| *Lithobates catesbeianus* | -76.3196 | 3.88848 |
| *Lithobates catesbeianus* | -76.7409 | 44.19402 |
| *Lithobates catesbeianus* | -96.799 | 39.06386 |
| *Lithobates catesbeianus* | -94.9782 | 32.51914 |
| *Lithobates catesbeianus* | -77.1429 | 39.56406 |
| *Lithobates catesbeianus* | -79.08 | 37.55 |
| *Lithobates catesbeianus* | -77.13 | 37.63 |
| *Lithobates catesbeianus* | -77.85 | 37.72 |
| *Lithobates catesbeianus* | -112.896 | 27.28217 |
| *Lithobates catesbeianus* | -117.625 | 33.51918 |
| *Lithobates catesbeianus* | 139.1485 | 35.25375 |
| *Lithobates catesbeianus* | -106.306 | 31.62718 |
| *Lithobates catesbeianus* | -103.769 | 30.51997 |
| *Lithobates catesbeianus* | -120.56 | 34.67643 |
| *Lithobates catesbeianus* | -120.54 | 34.68064 |
| *Lithobates catesbeianus* | -119.163 | 34.56162 |
| *Lithobates catesbeianus* | -80.8378 | 39.8078 |
| *Lithobates catesbeianus* | -80.5001 | 38.5004 |
| *Lithobates catesbeianus* | -94.2228 | 33.941 |
| *Lithobates catesbeianus* | -76.9057 | 40.2725 |
| *Lithobates catesbeianus* | -84.6859 | 39.61522 |
| *Lithobates catesbeianus* | -84.6199 | 39.24596 |
| *Lithobates catesbeianus* | -94.7197 | 37.0228 |
| *Lithobates catesbeianus* | -99.5547 | 39.0438 |
| *Lithobates catesbeianus* | -101.87 | 37.1031 |
| *Lithobates catesbeianus* | -118.211 | 36.97572 |
| *Lithobates catesbeianus* | -118.645 | 35.70742 |
| *Lithobates catesbeianus* | -121.564 | 41.94811 |
| *Lithobates catesbeianus* | -110.453 | 31.42788 |
| *Lithobates catesbeianus* | -121.778 | 37.22018 |
| *Lithobates catesbeianus* | -122.333 | 38.92647 |
| *Lithobates catesbeianus* | -121.036 | 39.95426 |
| *Lithobates catesbeianus* | -122.618 | 38.41087 |
| *Lithobates catesbeianus* | -119.244 | 36.93333 |
| *Lithobates catesbeianus* | -90.0668 | 46.16081 |
| *Lithobates catesbeianus* | -118.711 | 35.48175 |
| *Lithobates catesbeianus* | -100.29 | 35.49921 |
| *Lithobates catesbeianus* | -77.115 | 43.1352 |
| *Lithobates catesbeianus* | -74.4481 | 45.5645 |
| *Lithobates catesbeianus* | -80.8192 | 44.5201 |
| *Lithobates catesbeianus* | -79.4881 | 44.436 |
| *Lithobates catesbeianus* | -97.5668 | 39.11136 |
| *Lithobates catesbeianus* | -97.9707 | 38.54109 |
| *Lithobates catesbeianus* | -95.6987 | 37.16723 |
| *Lithobates catesbeianus* | -97.2699 | 39.37913 |
| *Lithobates catesbeianus* | -70.0296 | 41.27368 |
| *Lithobates catesbeianus* | -121.574 | 39.71405 |
| *Lithobates catesbeianus* | -111.544 | 33.55356 |
| *Lithobates catesbeianus* | -66.9795 | 18.41773 |
| *Lithobates catesbeianus* | -121.449 | 43.85755 |
| *Lithobates catesbeianus* | -105.875 | 37.4823 |
| *Lithobates catesbeianus* | -122.339 | 40.84965 |
| *Lithobates catesbeianus* | -114.699 | 32.62781 |
| *Lithobates catesbeianus* | -96.5685 | 30.54604 |
| *Lithobates catesbeianus* | -98.5014 | 30.27417 |
| *Lithobates catesbeianus* | -117.101 | 46.4166 |
| *Lithobates catesbeianus* | -122.574 | 47.38897 |
| *Lithobates catesbeianus* | -94.9701 | 39.38997 |
| *Lithobates catesbeianus* | -80.6697 | 39.55999 |
| *Lithobates catesbeianus* | -76.73 | 39.79 |
| *Lithobates catesbeianus* | -74.96 | 41.14 |
| *Lithobates catesbeianus* | -78.0596 | 39.49999 |
| *Lithobates catesbeianus* | -78.13 | 41.77 |
| *Lithobates catesbeianus* | -79.89 | 40.78 |
| *Lithobates catesbeianus* | -75.73 | 41.57 |
| *Lithobates catesbeianus* | -118.684 | 34.04278 |
| *Lithobates catesbeianus* | -84.7308 | 39.3077 |
| *Lithobates catesbeianus* | -84.5068 | 39.38958 |
| *Lithobates catesbeianus* | -83.749 | 38.724 |
| *Lithobates catesbeianus* | -83.9942 | 38.90593 |
| *Lithobates catesbeianus* | -84.6956 | 39.85789 |
| *Lithobates catesbeianus* | -83.5751 | 39.57508 |
| *Lithobates catesbeianus* | -75.6586 | 5.7625 |
| *Lithobates catesbeianus* | -65.067 | 44.4 |
| *Lithobates catesbeianus* | -76.267 | 45.35 |
| *Lithobates catesbeianus* | -82.433 | 42.317 |
| *Lithobates catesbeianus* | -74.525 | 45.733 |
| *Lithobates catesbeianus* | -76.667 | 45.583 |
| *Lithobates catesbeianus* | -75.475 | 45.507 |
| *Lithobates catesbeianus* | -76.09 | 45.622 |
| *Lithobates catesbeianus* | -74.976 | 45.522 |
| *Lithobates catesbeianus* | -75.75 | 45.483 |
| *Lithobates catesbeianus* | -74.666 | 45.033 |
| *Lithobates catesbeianus* | -75.655 | 44.873 |
| *Lithobates catesbeianus* | -75.844 | 46.178 |
| *Lithobates catesbeianus* | -122.75 | 49.117 |
| *Lithobates catesbeianus* | -80.25 | 42.567 |
| *Lithobates catesbeianus* | -75.34 | 45.526 |
| *Lithobates catesbeianus* | -75.917 | 45.533 |
| *Lithobates catesbeianus* | -76.2 | 45.667 |
| *Lithobates catesbeianus* | -97.1434 | 34.22689 |
| *Lithobates catesbeianus* | -97.8898 | 36.24583 |
| *Lithobates catesbeianus* | -99.7578 | 34.90076 |
| *Lithobates catesbeianus* | -92.6606 | 30.81248 |
| *Lithobates catesbeianus* | -97.1643 | 35.23227 |
| *Lithobates catesbeianus* | -94.7778 | 36.29049 |
| *Lithobates catesbeianus* | -97.2983 | 36.93603 |
| *Lithobates catesbeianus* | -79.4293 | 35.1315 |
| *Lithobates catesbeianus* | -83.6949 | 35.1583 |
| *Lithobates catesbeianus* | -81.13 | 35.3602 |
| *Lithobates catesbeianus* | -75.7077 | 35.9374 |
| *Lithobates catesbeianus* | -79.1812 | 34.8857 |
| *Lithobates catesbeianus* | -81.6451 | 36.2439 |
| *Lithobates catesbeianus* | -84.3892 | 36.6479 |
| *Lithobates catesbeianus* | -79.4027 | 35.1465 |
| *Lithobates catesbeianus* | -77.9156 | 34.1831 |
| *Lithobates catesbeianus* | -77.4736 | 37.6458 |
| *Lithobates catesbeianus* | -77.5664 | 37.6944 |
| *Lithobates catesbeianus* | -77.5483 | 37.6933 |
| *Lithobates catesbeianus* | -82.1153 | 35.6691 |
| *Lithobates catesbeianus* | -78.6634 | 36.6464 |
| *Lithobates catesbeianus* | -76.9931 | 37.3424 |
| *Lithobates catesbeianus* | -76.7077 | 36.9076 |
| *Lithobates catesbeianus* | -78.2666 | 37.8581 |
| *Lithobates catesbeianus* | -75.2105 | 38.7358 |
| *Lithobates catesbeianus* | -96.8848 | 35.92903 |
| *Lithobates catesbeianus* | -157.964 | 21.3874 |
| *Lithobates catesbeianus* | 126.838 | 36.74549 |
| *Lithobates catesbeianus* | -76.6961 | 39.88882 |
| *Lithobates catesbeianus* | -88.9301 | 37.37639 |
| *Lithobates catesbeianus* | -94.1462 | 32.66279 |
| *Lithobates catesbeianus* | -84.3888 | 40.16029 |
| *Lithobates catesbeianus* | -79.3097 | 44.72611 |
| *Lithobates catesbeianus* | -92.7146 | 32.62785 |
| *Lithobates catesbeianus* | -76.4519 | 42.47919 |
| *Lithobates catesbeianus* | -116.426 | 43.2939 |
| *Lithobates catesbeianus* | -122.22 | 37.41453 |
| *Lithobates catesbeianus* | -123.889 | 40.27731 |
| *Lithobates catesbeianus* | -122.214 | 37.83263 |
| *Lithobates catesbeianus* | -92.0757 | 38.58191 |
| *Lithobates catesbeianus* | -98.4933 | 29.42389 |
| *Lithobates catesbeianus* | -70.9063 | 42.65129 |
| *Lithobates catesbeianus* | -94.3894 | 29.57333 |
| *Lithobates catesbeianus* | -97.1705 | 30.08762 |
| *Lithobates catesbeianus* | -110.446 | 31.43011 |
| *Lithobates catesbeianus* | -116.286 | 35.04233 |
| *Lithobates catesbeianus* | -97.4521 | 28.30133 |
| *Lithobates catesbeianus* | -97.8051 | 30.24339 |
| *Lithobates catesbeianus* | -83.2066 | 39.91852 |
| *Lithobates catesbeianus* | -72.5252 | 44.52833 |
| *Lithobates catesbeianus* | -97.7427 | 30.24639 |
| *Lithobates catesbeianus* | -79.5442 | 45.16331 |
| *Lithobates catesbeianus* | -71.0243 | 42.04014 |
| *Lithobates catesbeianus* | -95.258 | 40.09782 |
| *Lithobates catesbeianus* | -73.3735 | 45.55096 |
| *Lithobates catesbeianus* | -122.896 | 48.48692 |
| *Lithobates catesbeianus* | -120.622 | 39.61671 |
| *Lithobates catesbeianus* | -122.756 | 45.79961 |
| *Lithobates catesbeianus* | -75.4924 | 45.50245 |
| *Lithobates catesbeianus* | -81.4564 | 41.07944 |
| *Lithobates catesbeianus* | -116.187 | 32.61768 |
| *Lithobates catesbeianus* | -73.1385 | 44.15893 |
| *Lithobates catesbeianus* | 8.17732 | 45.21953 |
| *Lithobates catesbeianus* | -96.102 | 30.64783 |
| *Lithobates catesbeianus* | 126.492 | 36.66291 |
| *Lithobates catesbeianus* | 126.4755 | 36.66456 |
| *Lithobates catesbeianus* | -123.409 | 46.1468 |
| *Lithobates catesbeianus* | -94.7072 | 31.33149 |
| *Lithobates catesbeianus* | -95.5991 | 29.37392 |
| *Lithobates catesbeianus* | -122.029 | 38.4917 |
| *Lithobates catesbeianus* | -96.7797 | 32.95539 |
| *Lithobates catesbeianus* | -74.1829 | 40.0926 |
| *Lithobates catesbeianus* | -74.7636 | 45.10778 |
| *Lithobates catesbeianus* | -81.9728 | 41.00113 |
| *Lithobates catesbeianus* | -71.1568 | 42.39536 |
| *Lithobates catesbeianus* | -118.213 | 34.20092 |
| *Lithobates catesbeianus* | -72.6082 | 41.75898 |
| *Lithobates catesbeianus* | -95.8807 | 31.9196 |
| *Lithobates catesbeianus* | -71.3365 | 42.2546 |
| *Lithobates catesbeianus* | -71.1473 | 42.38644 |
| *Lithobates catesbeianus* | -122.2 | 37.10575 |
| *Lithobates catesbeianus* | -121.368 | 38.05224 |
| *Lithobates catesbeianus* | -80.4315 | 45.69552 |
| *Lithobates catesbeianus* | -76.9096 | 44.75724 |
| *Lithobates catesbeianus* | -70.9217 | 44.775 |
| *Lithobates catesbeianus* | -155.055 | 19.73989 |
| *Lithobates catesbeianus* | -121.766 | 47.4329 |
| *Lithobates catesbeianus* | -71.2871 | 44.07095 |
| *Lithobates catesbeianus* | -117.063 | 32.55455 |
| *Lithobates catesbeianus* | -118.427 | 33.96626 |
| *Lithobates catesbeianus* | -79.0559 | 35.9132 |
| *Lithobates catesbeianus* | -97.1701 | 30.08808 |
| *Lithobates catesbeianus* | -121.719 | 37.37647 |
| *Lithobates catesbeianus* | -95.9378 | 33.59219 |
| *Lithobates catesbeianus* | -81.382 | 46.02198 |
| *Lithobates catesbeianus* | -73.3997 | 43.57337 |
| *Lithobates catesbeianus* | -97.2807 | 32.88374 |
| *Lithobates catesbeianus* | -69.2622 | 44.9211 |
| *Lithobates catesbeianus* | -71.8786 | 44.9165 |
| *Lithobates catesbeianus* | -74.7429 | 42.59394 |
| *Lithobates catesbeianus* | -89.4 | 36.38427 |
| *Lithobates catesbeianus* | -81.2597 | 36.3636 |
| *Lithobates catesbeianus* | -121.159 | 40.211 |
| *Lithobates catesbeianus* | -79.0333 | 45.4666 |
| *Lithobates catesbeianus* | -82.1256 | 42.8275 |
| *Lithobates catesbeianus* | -82.6333 | 46.333 |
| *Lithobates catesbeianus* | -79.2143 | 43.08143 |
| *Lithobates catesbeianus* | -120.343 | 38.26803 |
| *Lithobates catesbeianus* | -84.0625 | 42.09861 |
| *Lithobates catesbeianus* | -109.549 | 38.57333 |
| *Lithobates catesbeianus* | -73.3323 | 41.30741 |
| *Lithobates catesbeianus* | -74.7592 | 7.83821 |
| *Lithobates catesbeianus* | -75.8531 | 4.86677 |
| *Lithobates catesbeianus* | -95.3522 | 34.53188 |
| *Lithobates catesbeianus* | -76.231 | 44.77823 |
| *Lithobates catesbeianus* | -110.21 | 31.8383 |
| *Lithobates catesbeianus* | -96.9561 | 33.06723 |
| *Lithobates catesbeianus* | -76.4641 | 3.57695 |
| *Lithobates catesbeianus* | -155.277 | 19.4436 |
| *Lithobates catesbeianus* | -79.6342 | 35.0101 |
| *Lithobates catesbeianus* | -84.228 | 45.9891 |
| *Lithobates catesbeianus* | -79.9973 | 43.5167 |
| *Lithobates catesbeianus* | -95.1242 | 43.43524 |
| *Lithobates catesbeianus* | -95.1733 | 43.34774 |
| *Lithobates catesbeianus* | -117.622 | 33.70945 |
| *Lithobates catesbeianus* | -117.676 | 33.74753 |
| *Lithobates catesbeianus* | -117.695 | 33.62514 |
| *Lithobates catesbeianus* | -122.453 | 47.12757 |
| *Lithobates catesbeianus* | -114.571 | 33.63975 |
| *Lithobates catesbeianus* | -118.282 | 33.87268 |
| *Lithobates catesbeianus* | -117.848 | 34.31923 |
| *Lithobates catesbeianus* | -117.789 | 33.66829 |
| *Lithobates catesbeianus* | -121.081 | 35.56412 |
| *Lithobates catesbeianus* | -117.563 | 33.94606 |
| *Lithobates catesbeianus* | -101.756 | 32.73806 |
| *Lithobates catesbeianus* | -102.816 | 33.04994 |
| *Lithobates catesbeianus* | -94.9131 | 31.14273 |
| *Lithobates catesbeianus* | -96.432 | 30.42094 |
| *Lithobates catesbeianus* | -97.3409 | 30.16408 |
| *Lithobates catesbeianus* | -98.3526 | 31.17522 |
| *Lithobates catesbeianus* | -84.2889 | 39.88136 |
| *Lithobates catesbeianus* | -80.854 | 39.73593 |
| *Lithobates catesbeianus* | -81.6066 | 40.01272 |
| *Lithobates catesbeianus* | -84.0007 | 39.76433 |
| *Lithobates catesbeianus* | -84.2526 | 39.31812 |
| *Lithobates catesbeianus* | -83.0332 | 40.14665 |
| *Lithobates catesbeianus* | -84.8064 | 39.27197 |
| *Lithobates catesbeianus* | -116.913 | 32.85838 |
| *Lithobates catesbeianus* | -85.8827 | 32.78183 |
| *Lithobates catesbeianus* | -84.2188 | 39.53667 |
| *Lithobates catesbeianus* | -82.9035 | 39.14942 |
| *Lithobates catesbeianus* | -83.5548 | 41.08011 |
| *Lithobates catesbeianus* | -98.2028 | 38.2046 |
| *Lithobates catesbeianus* | -101.784 | 39.0456 |
| *Lithobates catesbeianus* | -94.7603 | 37.0391 |
| *Lithobates catesbeianus* | -94.74 | 37.8046 |
| *Lithobates catesbeianus* | -94.0283 | 30.71375 |
| *Lithobates catesbeianus* | -90.3079 | 38.62674 |
| *Lithobates catesbeianus* | -121.809 | 39.7447 |
| *Lithobates catesbeianus* | -117.155 | 32.89601 |
| *Lithobates catesbeianus* | -120.849 | 37.05833 |
| *Lithobates catesbeianus* | -119.286 | 36.49958 |
| *Lithobates catesbeianus* | -122.411 | 39.04775 |
| *Lithobates catesbeianus* | -121.796 | 37.63818 |
| *Lithobates catesbeianus* | -122.418 | 37.72332 |
| *Lithobates catesbeianus* | -117.369 | 33.29736 |
| *Lithobates catesbeianus* | -116.379 | 43.68294 |
| *Lithobates catesbeianus* | -115.154 | 36.17412 |
| *Lithobates catesbeianus* | -120.603 | 34.69167 |
| *Lithobates catesbeianus* | -119.831 | 34.42611 |
| *Lithobates catesbeianus* | -65.083 | 44.45 |
| *Lithobates catesbeianus* | -79.317 | 42.883 |
| *Lithobates catesbeianus* | -75.8 | 45.85 |
| *Lithobates catesbeianus* | -76.792 | 44.279 |
| *Lithobates catesbeianus* | -75.83 | 45.51 |
| *Lithobates catesbeianus* | -75.848 | 45.52 |
| *Lithobates catesbeianus* | -75.286 | 45.537 |
| *Lithobates catesbeianus* | -76.108 | 45.606 |
| *Lithobates catesbeianus* | -76.101 | 45.599 |
| *Lithobates catesbeianus* | -75.967 | 46.117 |
| *Lithobates catesbeianus* | -76.216 | 45.676 |
| *Lithobates catesbeianus* | -75.688 | 45.383 |
| *Lithobates catesbeianus* | -75.424 | 45.51 |
| *Lithobates catesbeianus* | -76.217 | 45.467 |
| *Lithobates catesbeianus* | -73.969 | 45.419 |
| *Lithobates catesbeianus* | -76.75 | 45.423 |
| *Lithobates catesbeianus* | -75.686 | 44.894 |
| *Lithobates catesbeianus* | -76.033 | 45.7 |
| *Lithobates catesbeianus* | -76.1 | 46.083 |
| *Lithobates catesbeianus* | -77.19 | 40.86 |
| *Lithobates catesbeianus* | -80.08 | 40.4 |
| *Lithobates catesbeianus* | -116.43 | 43.29 |
| *Lithobates catesbeianus* | -82.02 | 29.6 |
| *Lithobates catesbeianus* | -79.15 | 40.62 |
| *Lithobates catesbeianus* | -80.41 | 41.44 |
| *Lithobates catesbeianus* | -76.87 | 41.15 |
| *Lithobates catesbeianus* | -82.93 | 29.24 |
| *Lithobates catesbeianus* | -75.86 | 39.55 |
| *Lithobates catesbeianus* | -79.13 | 41.94 |
| *Lithobates catesbeianus* | -75.01 | 40.18 |
| *Lithobates catesbeianus* | -79.49 | 39.86 |
| *Lithobates catesbeianus* | -112.78 | 34.44 |
| *Lithobates catesbeianus* | -94.8078 | 37.64884 |
| *Lithobates catesbeianus* | -75.35 | 37.94 |
| *Lithobates catesbeianus* | -76.32 | 36.67 |
| *Lithobates catesbeianus* | -118.734 | 34.61678 |
| *Lithobates catesbeianus* | -76.1666 | 44.48333 |
| *Lithobates catesbeianus* | -79.1225 | 44.11196 |
| *Lithobates catesbeianus* | -97.4415 | 35.32616 |
| *Lithobates catesbeianus* | -94.8727 | 35.46339 |
| *Lithobates catesbeianus* | -99.287 | 34.87676 |
| *Lithobates catesbeianus* | -96.8003 | 33.8817 |
| *Lithobates catesbeianus* | -95.3036 | 36.18079 |
| *Lithobates catesbeianus* | -102.741 | 36.95922 |
| *Lithobates catesbeianus* | -96.9581 | 36.89264 |
| *Lithobates catesbeianus* | -99.0781 | 34.6212 |
| *Lithobates catesbeianus* | -97.9894 | 34.13719 |
| *Lithobates catesbeianus* | -79.0996 | 36.0753 |
| *Lithobates catesbeianus* | -75.6674 | 35.9878 |
| *Lithobates catesbeianus* | -75.5429 | 35.2351 |
| *Lithobates catesbeianus* | -76.1546 | 36.1335 |
| *Lithobates catesbeianus* | -78.966 | 35.4511 |
| *Lithobates catesbeianus* | -81.1191 | 35.337 |
| *Lithobates catesbeianus* | -79.4591 | 35.0431 |
| *Lithobates catesbeianus* | -81.0623 | 36.5483 |
| *Lithobates catesbeianus* | -81.0547 | 36.3328 |
| *Lithobates catesbeianus* | -86.9387 | 35.6451 |
| *Lithobates catesbeianus* | -75.4617 | 39.5054 |
| *Lithobates catesbeianus* | -76.443 | 36.9011 |
| *Lithobates catesbeianus* | -77.4417 | 37.6681 |
| *Lithobates catesbeianus* | -77.5024 | 37.5354 |
| *Lithobates catesbeianus* | -82.5389 | 35.7918 |
| *Lithobates catesbeianus* | -81.5352 | 41.12341 |
| *Lithobates catesbeianus* | -75.2596 | 39.89088 |
| *Lithobates catesbeianus* | -105.091 | 39.49487 |
| *Lithobates catesbeianus* | -106.891 | 33.80476 |
| *Lithobates catesbeianus* | -95.1496 | 39.00238 |
| *Lithobates catesbeianus* | -96.0279 | 37.60778 |
| *Lithobates catesbeianus* | -96.3959 | 38.46359 |
| *Lithobates catesbeianus* | -96.6454 | 38.37331 |
| *Lithobates catesbeianus* | -95.2262 | 38.98054 |
| *Lithobates catesbeianus* | -85.4128 | 42.61067 |
| *Lithobates catesbeianus* | -98.3481 | 39.51696 |
| *Lithobates catesbeianus* | -101.542 | 38.64606 |
| *Lithobates catesbeianus* | -76.9539 | 37.6403 |
| *Lithobates catesbeianus* | -80.39 | 42.58 |
| *Lithobates catesbeianus* | -84.4997 | 30.02236 |
| *Lithobates catesbeianus* | -84.9974 | 30.0275 |
| *Lithobates catesbeianus* | -81.646 | 30.3563 |
| *Lithobates catesbeianus* | -82.9887 | 29.55942 |
| *Lithobates catesbeianus* | -84.4394 | 30.04636 |
| *Lithobates catesbeianus* | -82.1517 | 29.33814 |
| *Lithobates catesbeianus* | -81.3 | 27.696 |
| *Lithobates catesbeianus* | -82.3317 | 29.4803 |
| *Lithobates catesbeianus* | -123.746 | 46.18482 |
| *Lithobates catesbeianus* | -124.233 | 43.40829 |
| *Lithobates catesbeianus* | -109.201 | 32.2498 |
| *Lithobates catesbeianus* | -110.961 | 31.45098 |
| *Lithobates catesbeianus* | -118.234 | 34.08851 |
| *Lithobates catesbeianus* | -119.148 | 45.8639 |
| *Lithobates catesbeianus* | -119.626 | 46.6886 |
| *Lithobates catesbeianus* | -70.8167 | 41.2556 |
| *Lithobates catesbeianus* | -119.361 | 35.9172 |
| *Lithobates catesbeianus* | -114.506 | 34.7953 |
| *Lithobates catesbeianus* | -114.455 | 34.6611 |
| *Lithobates catesbeianus* | -80.146 | 41.64418 |
| *Lithobates catesbeianus* | -77.4311 | 39.2951 |
| *Lithobates catesbeianus* | -82.6928 | 34.9432 |
| *Lithobates catesbeianus* | -83.9697 | 35.9384 |
| *Lithobates catesbeianus* | -77.46 | 37.88 |
| *Lithobates catesbeianus* | -78.1455 | 36.8698 |
| *Lithobates catesbeianus* | -110.847 | 33.7125 |
| *Lithobates catesbeianus* | -117.337 | 33.32478 |
| *Lithobates catesbeianus* | -80.5542 | 41.9476 |
| *Lithobates catesbeianus* | -83.6124 | 39.927 |
| *Lithobates catesbeianus* | -77.1043 | 38.75484 |
| *Lithobates catesbeianus* | -77.1047 | 38.75417 |
| *Lithobates catesbeianus* | -76.6093 | 42.50546 |
| *Lithobates catesbeianus* | -96.0995 | 30.78663 |
| *Lithobates catesbeianus* | -122.99 | 45.28067 |
| *Lithobates catesbeianus* | -76.3867 | 44.40971 |
| *Lithobates catesbeianus* | -71.1481 | 42.38784 |
| *Lithobates catesbeianus* | -72.8993 | 44.31833 |
| *Lithobates catesbeianus* | -121.669 | 37.28613 |
| *Lithobates catesbeianus* | -85.3044 | 30.78459 |
| *Lithobates catesbeianus* | -71.2675 | 42.3321 |
| *Lithobates catesbeianus* | -71.7239 | 41.46222 |
| *Lithobates catesbeianus* | -101.901 | 34.98051 |
| *Lithobates catesbeianus* | -122.45 | 47.11 |
| *Lithobates catesbeianus* | -121.884 | 42.51876 |
| *Lithobates catesbeianus* | -112.9 | 27.3 |
| *Lithobates catesbeianus* | -95.1958 | 16.18329 |
| *Lithobates catesbeianus* | -122.778 | 38.61257 |
| *Lithobates catesbeianus* | -98.4031 | 33.6377 |
| *Lithobates catesbeianus* | -83.6806 | 41.23755 |
| *Lithobates catesbeianus* | -117.043 | 32.83956 |
| *Lithobates catesbeianus* | -117.043 | 32.83953 |
| *Lithobates catesbeianus* | -121.367 | 38.05459 |
| *Lithobates catesbeianus* | -88.0713 | 41.81423 |
| *Lithobates catesbeianus* | -122.744 | 38.52642 |
| *Lithobates catesbeianus* | -72.3061 | 41.76593 |
| *Lithobates catesbeianus* | -75.8139 | 45.50577 |
| *Lithobates catesbeianus* | -109.035 | 31.8919 |
| *Lithobates catesbeianus* | -64.953 | 45.5929 |
| *Lithobates catesbeianus* | -118.244 | 34.10572 |
| *Lithobates catesbeianus* | -115.689 | 33.09284 |
| *Lithobates catesbeianus* | -98.0181 | 29.15887 |
| *Lithobates catesbeianus* | -95.3857 | 30.03578 |
| *Lithobates catesbeianus* | -76.8731 | 42.36959 |
| *Lithobates catesbeianus* | -86.6367 | 36.35527 |
| *Lithobates catesbeianus* | -109.779 | 23.36044 |
| *Lithobates catesbeianus* | -95.2218 | 29.56639 |
| *Lithobates catesbeianus* | -122.713 | 47.07255 |
| *Lithobates catesbeianus* | -76.7021 | 38.77967 |
| *Lithobates catesbeianus* | -97.4596 | 32.82605 |
| *Lithobates catesbeianus* | -95.9048 | 31.9405 |
| *Lithobates catesbeianus* | -123.234 | 49.17581 |
| *Lithobates catesbeianus* | -81.5798 | 41.19374 |
| *Lithobates catesbeianus* | -96.7672 | 31.92831 |
| *Lithobates catesbeianus* | 126.9414 | 35.92805 |
| *Lithobates catesbeianus* | -72.6095 | 41.75715 |
| *Lithobates catesbeianus* | -71.1813 | 42.43348 |
| *Lithobates catesbeianus* | -103.74 | 25.42625 |
| *Lithobates catesbeianus* | -118.212 | 34.20236 |
| *Lithobates catesbeianus* | -97.4863 | 35.63749 |
| *Lithobates catesbeianus* | -90.0759 | 35.89577 |
| *Lithobates catesbeianus* | -122.713 | 47.07227 |
| *Lithobates catesbeianus* | -123.085 | 45.51027 |
| *Lithobates catesbeianus* | -97.937 | 29.88806 |
| *Lithobates catesbeianus* | -75.4973 | 45.50236 |
| *Lithobates catesbeianus* | -75.6521 | 45.44592 |
| *Lithobates catesbeianus* | -76.0127 | 44.30212 |
| *Lithobates catesbeianus* | -95.2425 | 39.0417 |
| *Lithobates catesbeianus* | -76.7917 | 42.00972 |
| *Lithobates catesbeianus* | -73.1652 | 41.71356 |
| *Lithobates catesbeianus* | -73.4547 | 41.3778 |
| *Lithobates catesbeianus* | -73.154 | 42.032 |
| *Lithobates catesbeianus* | -82.3536 | 28.9906 |
| *Lithobates catesbeianus* | -77.9931 | 35.35 |
| *Lithobates catesbeianus* | -95.8797 | 34.42737 |
| *Lithobates catesbeianus* | -97.8535 | 29.997 |
| *Lithobates catesbeianus* | -84.644 | 40.09717 |
| *Lithobates catesbeianus* | -74.8173 | 4.27528 |
| *Lithobates catesbeianus* | -100.3 | 37.2 |
| *Lithobates catesbeianus* | -83.8543 | 41.41777 |
| *Lithobates catesbeianus* | -94.7635 | 33.78324 |
| *Lithobates catesbeianus* | 5.12891 | 51.14484 |
| *Lithobates catesbeianus* | 5.1294 | 51.14313 |
| *Lithobates catesbeianus* | 5.01525 | 51.2683 |
| *Lithobates catesbeianus* | -96.1835 | 36.7292 |
| *Lithobates catesbeianus* | -80.2833 | 45.91666 |
| *Lithobates catesbeianus* | -72.5737 | 44.28503 |
| *Lithobates catesbeianus* | 138.881 | 36.2739 |
| *Lithobates catesbeianus* | 139.091 | 36.3642 |
| *Lithobates catesbeianus* | 103.7883 | 1.34806 |
| *Lithobates catesbeianus* | -101.323 | 38.9042 |
| *Lithobates catesbeianus* | -114.602 | 33.60525 |
| *Lithobates catesbeianus* | -117.688 | 33.87289 |
| *Lithobates catesbeianus* | -124.097 | 43.87512 |
| *Lithobates catesbeianus* | -118.702 | 34.08234 |
| *Lithobates catesbeianus* | -71.9575 | 42.8706 |
| *Lithobates catesbeianus* | -118.035 | 33.79591 |
| *Lithobates catesbeianus* | -118.238 | 34.09479 |
| *Lithobates catesbeianus* | -117.385 | 33.23868 |
| *Lithobates catesbeianus* | -120.981 | 41.04622 |
| *Lithobates catesbeianus* | -116.982 | 48.31243 |
| *Lithobates catesbeianus* | -122.534 | 38.31419 |
| *Lithobates catesbeianus* | -155.632 | 19.58049 |
| *Lithobates catesbeianus* | -109.278 | 31.33707 |
| *Lithobates catesbeianus* | -122.303 | 45.5675 |
| *Lithobates catesbeianus* | -111.085 | 31.455 |
| *Lithobates catesbeianus* | -117.447 | 33.60602 |
| *Lithobates catesbeianus* | -114.375 | 47.32965 |
| *Lithobates catesbeianus* | -96.2162 | 30.45837 |
| *Lithobates catesbeianus* | -97.2417 | 30.21626 |
| *Lithobates catesbeianus* | -96.5029 | 30.4858 |
| *Lithobates catesbeianus* | -98.1334 | 26.48367 |
| *Lithobates catesbeianus* | -88.2505 | 29.19897 |
| *Lithobates catesbeianus* | -99.2279 | 33.52513 |
| *Lithobates catesbeianus* | -98.4894 | 29.28361 |
| *Lithobates catesbeianus* | -96.4602 | 32.93114 |
| *Lithobates catesbeianus* | -99.451 | 18.647 |
| *Lithobates catesbeianus* | -86.351 | 43.4134 |
| *Lithobates catesbeianus* | -84.2529 | 39.31814 |
| *Lithobates catesbeianus* | -83.3277 | 41.3277 |
| *Lithobates catesbeianus* | -83.448 | 40.20923 |
| *Lithobates catesbeianus* | -84.7889 | 39.41432 |
| *Lithobates catesbeianus* | -84.1823 | 39.3645 |
| *Lithobates catesbeianus* | -83.8455 | 41.3864 |
| *Lithobates catesbeianus* | -83.99 | 39.23831 |
| *Lithobates catesbeianus* | -84.7045 | 39.8726 |
| *Lithobates catesbeianus* | -84.2696 | 39.39277 |
| *Lithobates catesbeianus* | -116.93 | 32.664 |
| *Lithobates catesbeianus* | -83.2434 | 39.42746 |
| *Lithobates catesbeianus* | -122.178 | 40.3919 |
| *Lithobates catesbeianus* | -121.341 | 37.42641 |
| *Lithobates catesbeianus* | -121.337 | 37.42852 |
| *Lithobates catesbeianus* | -120.312 | 38.06214 |
| *Lithobates catesbeianus* | -122.213 | 37.35401 |
| *Lithobates catesbeianus* | -121.328 | 37.05904 |
| *Lithobates catesbeianus* | -121.125 | 35.59515 |
| *Lithobates catesbeianus* | -123.378 | 41.79333 |
| *Lithobates catesbeianus* | -72.1875 | 42.4875 |
| *Lithobates catesbeianus* | -98.0342 | 39.8837 |
| *Lithobates catesbeianus* | -99.2044 | 38.1599 |
| *Lithobates catesbeianus* | -94.6588 | 37.0398 |
| *Lithobates catesbeianus* | -117.128 | 33.07993 |
| *Lithobates catesbeianus* | -117.232 | 32.92582 |
| *Lithobates catesbeianus* | -109.031 | 32.03847 |
| *Lithobates catesbeianus* | -100.38 | 43.23 |
| *Lithobates catesbeianus* | -95.8226 | 39.50691 |
| *Lithobates catesbeianus* | -100.917 | 38.68968 |
| *Lithobates catesbeianus* | -94.6995 | 37.03308 |
| *Lithobates catesbeianus* | -95.2215 | 37.79083 |
| *Lithobates catesbeianus* | -96.1879 | 38.40887 |
| *Lithobates catesbeianus* | -82.4209 | 29.92928 |
| *Lithobates catesbeianus* | -81.7451 | 29.59091 |
| *Lithobates catesbeianus* | -76.4816 | 42.30832 |
| *Lithobates catesbeianus* | -75.667 | 45.383 |
| *Lithobates catesbeianus* | -72.783 | 46.75 |
| *Lithobates catesbeianus* | -75.983 | 45.75 |
| *Lithobates catesbeianus* | -75.678 | 45.06 |
| *Lithobates catesbeianus* | -76.09 | 45.604 |
| *Lithobates catesbeianus* | -81.571 | 45.194 |
| *Lithobates catesbeianus* | -75.702 | 45.428 |
| *Lithobates catesbeianus* | -75.821 | 45.462 |
| *Lithobates catesbeianus* | -80.383 | 42.567 |
| *Lithobates catesbeianus* | -83.517 | 43.033 |
| *Lithobates catesbeianus* | -76.237 | 45.245 |
| *Lithobates catesbeianus* | -76.198 | 44.539 |
| *Lithobates catesbeianus* | -76.05 | 46.083 |
| *Lithobates catesbeianus* | -80.0397 | 39.15 |
| *Lithobates catesbeianus* | -79.8097 | 39.01 |
| *Lithobates catesbeianus* | -81.43 | 31.73 |
| *Lithobates catesbeianus* | -79.73 | 40.2 |
| *Lithobates catesbeianus* | -75.65 | 40.65 |
| *Lithobates catesbeianus* | -74.93 | 40.21 |
| *Lithobates catesbeianus* | -98.1802 | 36.24002 |
| *Lithobates catesbeianus* | -100.949 | 38.79306 |
| *Lithobates catesbeianus* | -80.8897 | 37.71003 |
| *Lithobates catesbeianus* | -76.34 | 39.8 |
| *Lithobates catesbeianus* | -77.64 | 36.63 |
| *Lithobates catesbeianus* | -76.25 | 36.56 |
| *Lithobates catesbeianus* | -76.89 | 37.41 |
| *Lithobates catesbeianus* | -117.819 | 46.6265 |
| *Lithobates catesbeianus* | -79.3708 | 38.6056 |
| *Lithobates catesbeianus* | -79.9309 | 32.7766 |
| *Lithobates catesbeianus* | -83.9329 | 34.10944 |
| *Lithobates catesbeianus* | -89.3537 | 36.38019 |
| *Lithobates catesbeianus* | -70.4172 | 18.98486 |
| *Lithobates catesbeianus* | -78.7624 | 39.65563 |
| *Lithobates catesbeianus* | -85.2014 | 34.6988 |
| *Lithobates catesbeianus* | -77.1182 | 36.6111 |
| *Lithobates catesbeianus* | -78.0678 | 37.4983 |
| *Lithobates catesbeianus* | -82.4432 | 40.0715 |
| *Lithobates catesbeianus* | -93.8788 | 34.3926 |
| *Lithobates catesbeianus* | -106.998 | 32.55713 |
| *Lithobates catesbeianus* | -82.7489 | 28.8184 |
| *Lithobates catesbeianus* | -95.2928 | 34.8707 |
| *Lithobates catesbeianus* | -95.4697 | 34.6754 |
| *Lithobates catesbeianus* | -98.5391 | 36.89829 |
| *Lithobates catesbeianus* | -97.7529 | 33.984 |
| *Lithobates catesbeianus* | -98.5912 | 34.71067 |
| *Lithobates catesbeianus* | -98.9212 | 36.18292 |
| *Lithobates catesbeianus* | -82.9297 | 35.1247 |
| *Lithobates catesbeianus* | -75.6658 | 35.9887 |
| *Lithobates catesbeianus* | -75.6671 | 35.9875 |
| *Lithobates catesbeianus* | -80.0176 | 35.3922 |
| *Lithobates catesbeianus* | -79.8922 | 35.5865 |
| *Lithobates catesbeianus* | -78.3846 | 34.1186 |
| *Lithobates catesbeianus* | -79.1349 | 36.0773 |
| *Lithobates catesbeianus* | -78.3724 | 35.6223 |
| *Lithobates catesbeianus* | -82.2036 | 35.6178 |
| *Lithobates catesbeianus* | -77.5369 | 37.7422 |
| *Lithobates catesbeianus* | -78.3099 | 36.6168 |
| *Lithobates catesbeianus* | -78.3047 | 36.5022 |
| *Lithobates catesbeianus* | -77.3393 | 37.6116 |
| *Lithobates catesbeianus* | -77.1587 | 37.3356 |
| *Lithobates catesbeianus* | -88.9299 | 37.37657 |
| *Lithobates catesbeianus* | -122.224 | 37.37364 |
| *Lithobates catesbeianus* | -157.851 | 21.4914 |
| *Lithobates catesbeianus* | -84.6624 | 40.02114 |
| *Lithobates catesbeianus* | -87.865 | 41.77179 |
| *Lithobates catesbeianus* | -73.4119 | 43.57682 |
| *Lithobates catesbeianus* | -122.703 | 38.20063 |
| *Lithobates catesbeianus* | -95.9045 | 31.9405 |
| *Lithobates catesbeianus* | -117.252 | 33.4 |
| *Lithobates catesbeianus* | -88.1851 | 41.79596 |
| *Lithobates catesbeianus* | -118.431 | 33.97092 |
| *Lithobates catesbeianus* | -96.8597 | 37.51979 |
| *Lithobates catesbeianus* | -86.3818 | 35.31456 |
| *Lithobates catesbeianus* | -76.0423 | 44.50439 |
| *Lithobates catesbeianus* | -97.1481 | 33.07576 |
| *Lithobates catesbeianus* | -121 | 37.65775 |
| *Lithobates catesbeianus* | -120.698 | 35.75045 |
| *Lithobates catesbeianus* | -120.189 | 37.36813 |
| *Lithobates catesbeianus* | -120.292 | 36.89315 |
| *Lithobates catesbeianus* | -123 | 38.71798 |
| *Lithobates catesbeianus* | -123.315 | 38.66475 |
| *Lithobates catesbeianus* | -119.637 | 37.45175 |
| *Lithobates catesbeianus* | -118.41 | 34.12161 |
| *Lithobates catesbeianus* | -86.3502 | 41.5529 |
| *Lithobates catesbeianus* | -96.5899 | 32.87625 |
| *Lithobates catesbeianus* | -82.83 | 42.17338 |
| *Lithobates catesbeianus* | -121.653 | 37.24273 |
| *Lithobates catesbeianus* | -95.6189 | 29.51552 |
| *Lithobates catesbeianus* | 126.4442 | 36.74652 |
| *Lithobates catesbeianus* | -70.3045 | 43.68315 |
| *Lithobates catesbeianus* | -95.9046 | 41.00893 |
| *Lithobates catesbeianus* | -97.1021 | 32.58719 |
| *Lithobates catesbeianus* | -122.828 | 43.93193 |
| *Lithobates catesbeianus* | -74.4886 | 40.71423 |
| *Lithobates catesbeianus* | -68.7849 | 44.99002 |
| *Lithobates catesbeianus* | -118.277 | 34.05863 |
| *Lithobates catesbeianus* | -122.763 | 38.09688 |
| *Lithobates catesbeianus* | -97.7921 | 30.40455 |
| *Lithobates catesbeianus* | -76.4472 | 40.05919 |
| *Lithobates catesbeianus* | -98.354 | 35.43916 |
| *Lithobates catesbeianus* | -69.0376 | 45.87018 |
| *Lithobates catesbeianus* | -98.9021 | 29.27443 |
| *Lithobates catesbeianus* | -94.1376 | 30.9294 |
| *Lithobates catesbeianus* | -96.336 | 30.59183 |
| *Lithobates catesbeianus* | -81.5181 | 41.23238 |
| *Lithobates catesbeianus* | -79.7381 | 46.05148 |
| *Lithobates catesbeianus* | -117.024 | 32.67086 |
| *Lithobates catesbeianus* | -73.8103 | 42.67667 |
| *Lithobates catesbeianus* | -70.6439 | 41.3625 |
| *Lithobates catesbeianus* | -71.075 | 41.5222 |
| *Lithobates catesbeianus* | -70.8406 | 41.8753 |
| *Lithobates catesbeianus* | -73.6836 | 40.98056 |
| *Lithobates catesbeianus* | -73.0922 | 42.1931 |
| *Lithobates catesbeianus* | -95.1783 | 32.58935 |
| *Lithobates catesbeianus* | -118.412 | 34.12052 |
| *Lithobates catesbeianus* | -121.993 | 39.37803 |
| *Lithobates catesbeianus* | -71.584 | 41.38114 |
| *Lithobates catesbeianus* | -79.584 | 45.17096 |
| *Lithobates catesbeianus* | -95.7229 | 40.48541 |
| *Lithobates catesbeianus* | -122.058 | 37.02789 |
| *Lithobates catesbeianus* | -77.1045 | 38.75453 |
| *Lithobates catesbeianus* | -121.487 | 37.10545 |
| *Lithobates catesbeianus* | -96.9921 | 32.82829 |
| *Lithobates catesbeianus* | -90.1008 | 30.47509 |
| *Lithobates catesbeianus* | -76.4762 | 42.45229 |
| *Lithobates catesbeianus* | -82.2982 | 36.1764 |
| *Lithobates catesbeianus* | -72.5118 | 41.51583 |
| *Lithobates catesbeianus* | -83.8904 | 30.4741 |
| *Lithobates catesbeianus* | -83.5944 | 29.83072 |
| *Lithobates catesbeianus* | -82.2405 | 29.6736 |
| *Lithobates catesbeianus* | -77.7312 | 34.5288 |
| *Lithobates catesbeianus* | -97.3909 | 35.21147 |
| *Lithobates catesbeianus* | 5.12899 | 51.14385 |
| *Lithobates catesbeianus* | 5.12873 | 51.14359 |
| *Lithobates catesbeianus* | -94.2401 | 31.70207 |
| *Lithobates catesbeianus* | -73.4667 | 3.9 |
| *Lithobates catesbeianus* | -78.1833 | 45.2 |
| *Lithobates catesbeianus* | -79.8435 | 43.15197 |
| *Lithobates catesbeianus* | -105.853 | 37.47252 |
| *Lithobates catesbeianus* | -74.4458 | 39.46993 |
| *Lithobates catesbeianus* | -97.4592 | 35.16495 |
| *Lithobates catesbeianus* | -110.133 | 31.5333 |
| *Lithobates catesbeianus* | -87.9336 | 42.42423 |
| *Lithobates catesbeianus* | -95.9558 | 37.56861 |
| *Lithobates catesbeianus* | -96.9696 | 37.26833 |
| *Lithobates catesbeianus* | -84.8526 | 34.24943 |
| *Lithobates catesbeianus* | -84.1058 | 33.7121 |
| *Lithobates catesbeianus* | -97.1304 | 37.35307 |
| *Lithobates catesbeianus* | -91.0487 | 43.24637 |
| *Lithobates catesbeianus* | -73.0875 | 43.9089 |
| *Lithobates catesbeianus* | -77.24 | 38.14 |
| *Lithobates catesbeianus* | -76.41 | 36.86 |
| *Lithobates catesbeianus* | -78.3209 | 36.6331 |
| *Lithobates catesbeianus* | -118.305 | 35.6645 |
| *Lithobates catesbeianus* | -106.618 | 31.91534 |
| *Lithobates catesbeianus* | -106.593 | 31.841 |
| *Lithobates catesbeianus* | -97.3277 | 30.3617 |
| *Lithobates catesbeianus* | -92.2571 | 38.0745 |
| *Lithobates catesbeianus* | -82.7491 | 41.5431 |
| *Lithobates catesbeianus* | -96.8567 | 35.07257 |
| *Lithobates catesbeianus* | -95.9092 | 36.2163 |
| *Lithobates catesbeianus* | -102.885 | 36.85732 |
| *Lithobates catesbeianus* | -78.0484 | 34.745 |
| *Lithobates catesbeianus* | -78.5814 | 35.8585 |
| *Lithobates catesbeianus* | -78.7062 | 35.7947 |
| *Lithobates catesbeianus* | -78.4387 | 35.6096 |
| *Lithobates catesbeianus* | -79.0011 | 36.0749 |
| *Lithobates catesbeianus* | -79.1441 | 36.1027 |
| *Lithobates catesbeianus* | -81.2012 | 36.568 |
| *Lithobates catesbeianus* | -79.3969 | 34.8737 |
| *Lithobates catesbeianus* | -78.4773 | 35.1046 |
| *Lithobates catesbeianus* | -79.3679 | 35.1537 |
| *Lithobates catesbeianus* | -87.5387 | 36.3486 |
| *Lithobates catesbeianus* | -74.6676 | 40.066 |
| *Lithobates catesbeianus* | -79.3578 | 35.1536 |
| *Lithobates catesbeianus* | -80.1062 | 34.7628 |
| *Lithobates catesbeianus* | -77.5322 | 37.7125 |
| *Lithobates catesbeianus* | -77.4775 | 37.6289 |
| *Lithobates catesbeianus* | -78.5382 | 36.7309 |
| *Lithobates catesbeianus* | -79.0589 | 36.0403 |
| *Lithobates catesbeianus* | -78.1396 | 39.0604 |
| *Lithobates catesbeianus* | -93.1497 | 44.4613 |
| *Lithobates catesbeianus* | -98.957 | 18.524 |
| *Lithobates catesbeianus* | -76.316 | 43.1937 |
| *Lithobates catesbeianus* | -80.0965 | 43.5781 |
| *Lithobates catesbeianus* | -81.0291 | 43.246 |
| *Lithobates catesbeianus* | -81.0491 | 39.79914 |
| *Lithobates catesbeianus* | -84.7391 | 39.40933 |
| *Lithobates catesbeianus* | -84.6584 | 39.35103 |
| *Lithobates catesbeianus* | -84.8047 | 39.84444 |
| *Lithobates catesbeianus* | -83.9275 | 39.8193 |
| *Lithobates catesbeianus* | -83.196 | 40.0428 |
| *Lithobates catesbeianus* | -81.9097 | 38.88264 |
| *Lithobates catesbeianus* | -84.5025 | 39.53918 |
| *Lithobates catesbeianus* | -118.304 | 33.89619 |
| *Lithobates catesbeianus* | -117.234 | 33.37661 |
| *Lithobates catesbeianus* | -115.214 | 37.59843 |
| *Lithobates catesbeianus* | -80.06 | 40.46 |
| *Lithobates catesbeianus* | -80.18 | 40.93 |
| *Lithobates catesbeianus* | -83.21 | 35.06 |
| *Lithobates catesbeianus* | -81.25 | 28.73 |
| *Lithobates catesbeianus* | -80.18 | 39.89 |
| *Lithobates catesbeianus* | -78.9196 | 39.46999 |
| *Lithobates catesbeianus* | -75.29 | 39.94 |
| *Lithobates catesbeianus* | -73.26 | 43.75 |
| *Lithobates catesbeianus* | -73.98 | 40.87 |
| *Lithobates catesbeianus* | -79.95 | 40.85 |
| *Lithobates catesbeianus* | -80.51 | 44.31 |
| *Lithobates catesbeianus* | -102.94 | 36.92999 |
| *Lithobates catesbeianus* | -78.01 | 40.82 |
| *Lithobates catesbeianus* | -81.08 | 31.97 |
| *Lithobates catesbeianus* | -75.2 | 41.31 |
| *Lithobates catesbeianus* | -76.56 | 36.71 |
| *Lithobates catesbeianus* | -82.06 | 36.93 |
| *Lithobates catesbeianus* | -77.32 | 38.07 |
| *Lithobates catesbeianus* | -72.75 | 46.717 |
| *Lithobates catesbeianus* | -80.483 | 42.567 |
| *Lithobates catesbeianus* | -76.367 | 44.533 |
| *Lithobates catesbeianus* | -75.783 | 45.617 |
| *Lithobates catesbeianus* | -76.317 | 44.583 |
| *Lithobates catesbeianus* | -75.822 | 44.813 |
| *Lithobates catesbeianus* | -76.624 | 44.819 |
| *Lithobates catesbeianus* | -75.913 | 45.596 |
| *Lithobates catesbeianus* | -76.049 | 45.617 |
| *Lithobates catesbeianus* | -76.067 | 45.483 |
| *Lithobates catesbeianus* | -75.721 | 45.583 |
| *Lithobates catesbeianus* | -77.058 | 45.633 |
| *Lithobates catesbeianus* | -65.241 | 44.216 |
| *Lithobates catesbeianus* | -66.039 | 45.283 |
| *Lithobates catesbeianus* | -76.435 | 44.506 |
| *Lithobates catesbeianus* | -76.252 | 45.421 |
| *Lithobates catesbeianus* | -79.743 | 46.681 |
| *Lithobates catesbeianus* | -75.7 | 45.417 |
| *Lithobates catesbeianus* | -78.033 | 45.1 |
| *Lithobates catesbeianus* | -77.233 | 45.583 |
| *Lithobates catesbeianus* | -84.5504 | 32.52225 |
| *Lithobates catesbeianus* | -95.8487 | 37.80333 |
| *Lithobates catesbeianus* | -96.042 | 37.11129 |
| *Lithobates catesbeianus* | -95.9595 | 37.61861 |
| *Lithobates catesbeianus* | -85.787 | 42.29929 |
| *Lithobates catesbeianus* | -94.9161 | 38.95532 |
| *Lithobates catesbeianus* | -96.4017 | 30.62806 |
| *Lithobates catesbeianus* | -96.3344 | 30.6425 |
| *Lithobates catesbeianus* | -96.5533 | 30.63417 |
| *Lithobates catesbeianus* | -70.6736 | 41.52639 |
| *Lithobates catesbeianus* | -101.678 | 35.7055 |
| *Lithobates catesbeianus* | -114.683 | 35.066 |
| *Lithobates catesbeianus* | -70.0185 | 41.9319 |
| *Lithobates catesbeianus* | -110.813 | 31.6875 |
| *Lithobates catesbeianus* | -111.089 | 32.17841 |
| *Lithobates catesbeianus* | -110.584 | 31.38343 |
| *Lithobates catesbeianus* | -110.617 | 43.63923 |
| *Lithobates catesbeianus* | -124.155 | 40.26027 |
| *Lithobates catesbeianus* | -104.958 | 39.24025 |
| *Lithobates catesbeianus* | -105.116 | 39.79897 |
| *Lithobates catesbeianus* | -118.102 | 34.06066 |
| *Lithobates catesbeianus* | -116.22 | 35.82563 |
| *Lithobates catesbeianus* | -117.683 | 33.63491 |
| *Lithobates catesbeianus* | -115.601 | 33.1257 |
| *Lithobates catesbeianus* | -94.7227 | 36.19517 |
| *Lithobates catesbeianus* | -120.101 | 34.58498 |
| *Lithobates catesbeianus* | -122.6 | 40.0775 |
| *Lithobates catesbeianus* | -121.572 | 36.85679 |
| *Lithobates catesbeianus* | 102.2803 | 27.82878 |
| *Lithobates catesbeianus* | -121.324 | 39.70504 |
| *Lithobates catesbeianus* | -122.185 | 38.83119 |
| *Lithobates catesbeianus* | -121.013 | 37.94018 |
| *Lithobates catesbeianus* | -82.056 | 29.47159 |
| *Lithobates catesbeianus* | -94.6928 | 37.0895 |
| *Lithobates catesbeianus* | -98.5196 | 37.53233 |
| *Lithobates catesbeianus* | -94.9234 | 37.27844 |
| *Lithobates catesbeianus* | -75.9331 | 38.95344 |
| *Lithobates catesbeianus* | -77.1042 | 38.75466 |
| *Lithobates catesbeianus* | -77.4318 | 39.29462 |
| *Lithobates catesbeianus* | -70.5617 | 44.96622 |
| *Lithobates catesbeianus* | -108.595 | 32.84506 |
| *Lithobates catesbeianus* | -122.713 | 47.07394 |
| *Lithobates catesbeianus* | -97.6542 | 30.21984 |
| *Lithobates catesbeianus* | -122.66 | 38.21607 |
| *Lithobates catesbeianus* | -76.4347 | 38.39453 |
| *Lithobates catesbeianus* | -118.441 | 34.06613 |
| *Lithobates catesbeianus* | -102.953 | 29.17813 |
| *Lithobates catesbeianus* | -90.1175 | 29.78309 |
| *Lithobates catesbeianus* | -90.1174 | 29.78371 |
| *Lithobates catesbeianus* | -120.622 | 39.61662 |
| *Lithobates catesbeianus* | -119.826 | 39.54887 |
| *Lithobates catesbeianus* | -118.009 | 37.79655 |
| *Lithobates catesbeianus* | -118.995 | 35.41202 |
| *Lithobates catesbeianus* | -102.118 | 31.96434 |
| *Lithobates catesbeianus* | -73.1776 | 44.0191 |
| *Lithobates catesbeianus* | -77.5986 | 45.8832 |
| *Lithobates catesbeianus* | -72.1766 | 44.39486 |
| *Lithobates catesbeianus* | -82.2611 | 38.58823 |
| *Lithobates catesbeianus* | -77.8049 | 39.48525 |
| *Lithobates catesbeianus* | -77.9218 | 38.01513 |
| *Lithobates catesbeianus* | -71.7025 | 44.76735 |
| *Lithobates catesbeianus* | -76.9378 | 38.99348 |
| *Lithobates catesbeianus* | -73.1416 | 44.15533 |
| *Lithobates catesbeianus* | -122.654 | 38.45376 |
| *Lithobates catesbeianus* | -72.6092 | 41.75702 |
| *Lithobates catesbeianus* | -73.0817 | 43.78918 |
| *Lithobates catesbeianus* | -105.59 | 36.39921 |
| *Lithobates catesbeianus* | -122.757 | 47.18075 |
| *Lithobates catesbeianus* | -97.4864 | 35.63739 |
| *Lithobates catesbeianus* | -72.1704 | 44.32862 |
| *Lithobates catesbeianus* | -81.1882 | 35.82004 |
| *Lithobates catesbeianus* | -80.7802 | 35.4649 |
| *Lithobates catesbeianus* | -71.364 | 43.54272 |
| *Lithobates catesbeianus* | -71.9375 | 42.0281 |
| *Lithobates catesbeianus* | -120.66 | 35.28275 |
| *Lithobates catesbeianus* | -122.174 | 37.36314 |
| *Lithobates catesbeianus* | -99.6204 | 33.30747 |
| *Lithobates catesbeianus* | -96.5167 | 32.72201 |
| *Lithobates catesbeianus* | -93.0821 | 36.57139 |
| *Lithobates catesbeianus* | -87.8571 | 41.64198 |
| *Lithobates catesbeianus* | -75.5009 | 45.50041 |
| *Lithobates catesbeianus* | -76.7011 | 38.78171 |
| *Lithobates catesbeianus* | -121.76 | 36.94009 |
| *Lithobates catesbeianus* | -117.289 | 33.05137 |
| *Lithobates catesbeianus* | -81.9403 | 41.01626 |
| *Lithobates catesbeianus* | -98.5114 | 33.93315 |
| *Lithobates catesbeianus* | 126.3464 | 36.72623 |
| *Lithobates catesbeianus* | -122.487 | 47.44724 |
| *Lithobates catesbeianus* | -97.5971 | 28.34539 |
| *Lithobates catesbeianus* | -82.2719 | 33.46302 |
| *Lithobates catesbeianus* | -73.9717 | 40.77878 |
| *Lithobates catesbeianus* | -77.1101 | 38.7517 |
| *Lithobates catesbeianus* | -74.3663 | 41.74385 |
| *Lithobates catesbeianus* | -95.2397 | 39.0283 |
| *Lithobates catesbeianus* | -74.6269 | 40.7122 |
| *Lithobates catesbeianus* | -73.3145 | 41.65229 |
| *Lithobates catesbeianus* | -71.9227 | 41.58521 |
| *Lithobates catesbeianus* | -72.0847 | 41.53248 |
| *Lithobates catesbeianus* | -72.8381 | 41.3243 |
| *Lithobates catesbeianus* | -72.0736 | 41.54892 |
| *Lithobates catesbeianus* | -122.595 | 39.25993 |
| *Lithobates catesbeianus* | -117.378 | 33.28869 |
| *Lithobates catesbeianus* | -120.42 | 38.0175 |
| *Lithobates catesbeianus* | -100.324 | 35.60007 |
| *Lithobates catesbeianus* | -98.4707 | 37.47102 |
| *Lithobates catesbeianus* | -94.6995 | 37.03766 |
| *Lithobates catesbeianus* | -95.2182 | 32.5305 |
| *Lithobates catesbeianus* | -97.2585 | 39.02608 |
| *Lithobates catesbeianus* | -97.3029 | 38.07166 |
| *Lithobates catesbeianus* | -98.1132 | 37.64943 |
| *Lithobates catesbeianus* | -95.8718 | 38.41555 |
| *Lithobates catesbeianus* | -97.107 | 39.91722 |
| *Lithobates catesbeianus* | -99.413 | 37.89128 |
| *Lithobates catesbeianus* | -101.998 | 37.52081 |
| *Lithobates catesbeianus* | -96.774 | 39.10553 |
| *Lithobates catesbeianus* | -96.4171 | 30.88892 |
| *Lithobates catesbeianus* | -96.3344 | 30.67139 |
| *Lithobates catesbeianus* | -96.4236 | 30.55917 |
| *Lithobates catesbeianus* | -96.4097 | 29.88598 |
| *Lithobates catesbeianus* | -96.3299 | 31.67311 |
| *Lithobates catesbeianus* | -99.2085 | 33.48486 |
| *Lithobates catesbeianus* | -96.5608 | 31.62124 |
| *Lithobates catesbeianus* | -94.1375 | 29.84809 |
| *Lithobates catesbeianus* | -98.6247 | 34.09602 |
| *Lithobates catesbeianus* | -98.2342 | 33.82444 |
| *Lithobates catesbeianus* | -84.2539 | 41.25387 |
| *Lithobates catesbeianus* | -81.07 | 31.98 |
| *Lithobates catesbeianus* | -84.7584 | 39.35585 |
| *Lithobates catesbeianus* | -83.7705 | 39.67145 |
| *Lithobates catesbeianus* | -83.1558 | 38.80895 |
| *Lithobates catesbeianus* | -84.2707 | 39.3933 |
| *Lithobates catesbeianus* | -84.2688 | 39.6825 |
| *Lithobates catesbeianus* | -95.0981 | 43.47718 |
| *Lithobates catesbeianus* | -117.259 | 34.47872 |
| *Lithobates catesbeianus* | -120.584 | 37.64056 |
| *Lithobates catesbeianus* | -114.737 | 33.43879 |
| *Lithobates catesbeianus* | -118.385 | 34.26041 |
| *Lithobates catesbeianus* | -117.583 | 33.40422 |
| *Lithobates catesbeianus* | -121.779 | 37.18508 |
| *Lithobates catesbeianus* | -117.645 | 33.89034 |
| *Lithobates catesbeianus* | -117.98 | 36.54767 |
| *Lithobates catesbeianus* | -117.236 | 32.91305 |
| *Lithobates catesbeianus* | -75.609 | 45.657 |
| *Lithobates catesbeianus* | -80.217 | 42.575 |
| *Lithobates catesbeianus* | -81.717 | 45.217 |
| *Lithobates catesbeianus* | -76.667 | 46.217 |
| *Lithobates catesbeianus* | -64.32 | 44.562 |
| *Lithobates catesbeianus* | -75.824 | 45.507 |
| *Lithobates catesbeianus* | -76.202 | 45.651 |
| *Lithobates catesbeianus* | -64.233 | 45.9 |
| *Lithobates catesbeianus* | -76.651 | 46.225 |
| *Lithobates catesbeianus* | -77.5 | 44.583 |
| *Lithobates catesbeianus* | -77.626 | 46.17 |
| *Lithobates catesbeianus* | -75.667 | 44.868 |
| *Lithobates catesbeianus* | -75.669 | 44.984 |
| *Lithobates catesbeianus* | -75.828 | 45.558 |
| *Lithobates catesbeianus* | -66.25 | 18.467 |
| *Lithobates catesbeianus* | -76.5 | 45.5 |
| *Lithobates catesbeianus* | -122.967 | 49.233 |
| *Lithobates catesbeianus* | -75.717 | 45.417 |
| *Lithobates catesbeianus* | -79.1596 | 39.11 |
| *Lithobates catesbeianus* | -80.13 | 40.26 |
| *Lithobates catesbeianus* | -92.71 | 32.25 |
| *Lithobates catesbeianus* | -102.87 | 36.81999 |
| *Lithobates catesbeianus* | -79.05 | 38.02 |
| *Lithobates catesbeianus* | -76.01 | 36.77 |
| *Lithobates catesbeianus* | -76.85 | 36.82 |
| *Lithobates catesbeianus* | -121.777 | 48.5597 |
| *Lithobates catesbeianus* | -122.823 | 47.0198 |
| *Lithobates catesbeianus* | -74.7489 | 39.2014 |
| *Lithobates catesbeianus* | -78.3275 | 34.7734 |
| *Lithobates catesbeianus* | -74.4547 | 40.40942 |
| *Lithobates catesbeianus* | -97.2518 | 35.11789 |
| *Lithobates catesbeianus* | 4.74745 | 51.46761 |
| *Lithobates catesbeianus* | 5.12747 | 51.1434 |
| *Lithobates catesbeianus* | 5.12891 | 51.14358 |
| *Lithobates catesbeianus* | -98.0852 | 29.96305 |
| *Lithobates catesbeianus* | -121.741 | 38.54491 |
| *Lithobates catesbeianus* | -76.3823 | 3.81542 |
| *Lithobates catesbeianus* | -95.4639 | 32.62908 |
| *Lithobates catesbeianus* | -83.2018 | 41.61016 |
| *Lithobates catesbeianus* | -87.236 | 30.96419 |
| *Lithobates catesbeianus* | -82.9187 | 29.79912 |
| *Lithobates catesbeianus* | -81.9187 | 27.7692 |
| *Lithobates catesbeianus* | -81.8843 | 29.3289 |
| *Lithobates catesbeianus* | -84.2857 | 30.4448 |
| *Lithobates catesbeianus* | -82.1733 | 28.0381 |
| *Lithobates catesbeianus* | -81.749 | 28.004 |
| *Lithobates catesbeianus* | -82.2783 | 29.5055 |
| *Lithobates catesbeianus* | -78.3791 | 45.83716 |
| *Lithobates catesbeianus* | -64.9522 | 45.59379 |
| *Lithobates catesbeianus* | -116.987 | 32.84498 |
| *Lithobates catesbeianus* | -97.7344 | 30.43018 |
| *Lithobates catesbeianus* | -110.998 | 31.42958 |
| *Lithobates catesbeianus* | -99.1221 | 39.66124 |
| *Lithobates catesbeianus* | -78.9 | 45.95 |
| *Lithobates catesbeianus* | -74.5167 | 45.08333 |
| *Lithobates catesbeianus* | -83.1478 | 42.8845 |
| *Lithobates catesbeianus* | -80.3645 | 43.0829 |
| *Lithobates catesbeianus* | -80.502 | 44.1055 |
| *Lithobates catesbeianus* | -77.597 | 45.382 |
| *Lithobates catesbeianus* | -88.0045 | 44.6568 |
| *Lithobates catesbeianus* | -111.967 | 40.96123 |
| *Lithobates catesbeianus* | -106.799 | 34.45972 |
| *Lithobates catesbeianus* | -157.975 | 21.44923 |
| *Lithobates catesbeianus* | -110.879 | 31.4857 |
| *Lithobates catesbeianus* | -122.769 | 38.44009 |
| *Lithobates catesbeianus* | -116.804 | 33.78331 |
| *Lithobates catesbeianus* | -95.4386 | 45.9169 |
| *Lithobates catesbeianus* | -92.9094 | 44.99902 |
| *Lithobates catesbeianus* | -93.283 | 44.34809 |
| *Lithobates catesbeianus* | -66.1397 | 18.4431 |
| *Lithobates catesbeianus* | -76.9739 | 37.6197 |
| *Lithobates catesbeianus* | -77.9463 | 39.34742 |
| *Lithobates catesbeianus* | -82.8671 | 34.74319 |
| *Lithobates catesbeianus* | -77.3705 | 39.45256 |
| *Lithobates catesbeianus* | -80.5844 | 40.93951 |
| *Lithobates catesbeianus* | -75.0764 | 38.7208 |
| *Lithobates catesbeianus* | -76.49 | 37.09 |
| *Lithobates catesbeianus* | -76.9701 | 37.1967 |
| *Lithobates catesbeianus* | -82.9192 | 36.7957 |
| *Lithobates catesbeianus* | -79.87 | 37.88 |
| *Lithobates catesbeianus* | -76.8979 | 37.1908 |
| *Lithobates catesbeianus* | -82.5323 | 39.9 |
| *Lithobates catesbeianus* | -106.532 | 31.80288 |
| *Lithobates catesbeianus* | -95.923 | 36.1334 |
| *Lithobates catesbeianus* | -95.3495 | 34.9129 |
| *Lithobates catesbeianus* | -98.1849 | 34.76857 |
| *Lithobates catesbeianus* | -98.6121 | 34.76627 |
| *Lithobates catesbeianus* | -95.2494 | 35.3101 |
| *Lithobates catesbeianus* | -95.2561 | 33.18871 |
| *Lithobates catesbeianus* | -94.7151 | 34.03101 |
| *Lithobates catesbeianus* | -96.9856 | 35.26117 |
| *Lithobates catesbeianus* | -100.208 | 36.56503 |
| *Lithobates catesbeianus* | -94.6422 | 34.09331 |
| *Lithobates catesbeianus* | -99.5676 | 35.21005 |
| *Lithobates catesbeianus* | -95.9068 | 34.45539 |
| *Lithobates catesbeianus* | -81.5859 | 35.5896 |
| *Lithobates catesbeianus* | -80.2737 | 36.4096 |
| *Lithobates catesbeianus* | -78.6081 | 35.4734 |
| *Lithobates catesbeianus* | -80.71 | 36.1182 |
| *Lithobates catesbeianus* | -79.7274 | 33.0174 |
| *Lithobates catesbeianus* | -84.3687 | 35.9177 |
| *Lithobates catesbeianus* | -81.1829 | 36.5515 |
| *Lithobates catesbeianus* | -75.4662 | 38.5251 |
| *Lithobates catesbeianus* | -75.8091 | 39.3084 |
| *Lithobates catesbeianus* | -79.3915 | 35.1523 |
| *Lithobates catesbeianus* | -75.2262 | 38.7899 |
| *Lithobates catesbeianus* | -81.3801 | 34.9822 |
| *Lithobates catesbeianus* | -80.7811 | 35.4454 |
| *Lithobates catesbeianus* | -77.0322 | 37.4986 |
| *Lithobates catesbeianus* | -74.6482 | 41.275 |
| *Lithobates catesbeianus* | -76.2063 | 35.8371 |
| *Lithobates catesbeianus* | -78.2029 | 38.1639 |
| *Lithobates catesbeianus* | -120.544 | 34.68047 |
| *Lithobates catesbeianus* | -120.573 | 34.77228 |
| *Lithobates catesbeianus* | -76.8861 | 40.30774 |
| *Lithobates catesbeianus* | -103.622 | 29.54799 |
| *Lithobates catesbeianus* | -97.3191 | 35.13887 |
| *Lithobates catesbeianus* | -77.928 | 39.99512 |
| *Lithobates catesbeianus* | -119.107 | 39.12185 |
| *Lithobates catesbeianus* | -95.0641 | 31.89391 |
| *Lithobates catesbeianus* | -96.1773 | 30.81176 |
| *Lithobates catesbeianus* | -73.3996 | 43.5731 |
| *Lithobates catesbeianus* | -117.937 | 33.90881 |
| *Lithobates catesbeianus* | -95.8811 | 31.93052 |
| *Lithobates catesbeianus* | -71.4449 | 43.71594 |
| *Lithobates catesbeianus* | -84.7324 | 40.14842 |
| *Lithobates catesbeianus* | -75.9999 | 39.99411 |
| *Lithobates catesbeianus* | -73.1389 | 43.80567 |
| *Lithobates catesbeianus* | -121.58 | 37.06097 |
| *Lithobates catesbeianus* | -121.452 | 43.82157 |
| *Lithobates catesbeianus* | -69.996 | 41.88231 |
| *Lithobates catesbeianus* | -119.96 | 37.48041 |
| *Lithobates catesbeianus* | -122.191 | 39.2487 |
| *Lithobates catesbeianus* | -117.314 | 34.58093 |
| *Lithobates catesbeianus* | -120.17 | 37.37212 |
| *Lithobates catesbeianus* | -82.0575 | 29.21666 |
| *Lithobates catesbeianus* | -118.399 | 37.46906 |
| *Lithobates catesbeianus* | -122.25 | 37.89619 |
| *Lithobates catesbeianus* | -91.1636 | 30.3781 |
| *Lithobates catesbeianus* | -75.5624 | 40.41291 |
| *Lithobates catesbeianus* | -122.95 | 45.22069 |
| *Lithobates catesbeianus* | -77.1042 | 38.75467 |
| *Lithobates catesbeianus* | -68.0215 | 45.2382 |
| *Lithobates catesbeianus* | -95.9116 | 29.80687 |
| *Lithobates catesbeianus* | -71.2533 | 43.0095 |
| *Lithobates catesbeianus* | -95.0493 | 31.18731 |
| *Lithobates catesbeianus* | -121.777 | 37.20634 |
| *Lithobates catesbeianus* | -122.152 | 37.12198 |
| *Lithobates catesbeianus* | -119.556 | 37.08708 |
| *Lithobates catesbeianus* | -95.8935 | 36.21431 |
| *Lithobates catesbeianus* | -97.9364 | 29.88885 |
| *Lithobates catesbeianus* | -88.7997 | 37.23164 |
| *Lithobates catesbeianus* | -98.4585 | 28.69538 |
| *Lithobates catesbeianus* | -123.313 | 44.4223 |
| *Lithobates catesbeianus* | -121.662 | 37.25433 |
| *Lithobates catesbeianus* | -97.2257 | 29.78461 |
| *Lithobates catesbeianus* | 126.346 | 36.721 |
| *Lithobates catesbeianus* | -82.4182 | 39.978 |
| *Lithobates catesbeianus* | -74.4884 | 40.71417 |
| *Lithobates catesbeianus* | 126.3464 | 36.72645 |
| *Lithobates catesbeianus* | -81.9393 | 41.01634 |
| *Lithobates catesbeianus* | -117.043 | 32.83951 |
| *Lithobates catesbeianus* | -122.035 | 37.20798 |
| *Lithobates catesbeianus* | -94.8478 | 38.95901 |
| *Lithobates catesbeianus* | -121.532 | 37.16859 |
| *Lithobates catesbeianus* | -76.3199 | 37.43708 |
| *Lithobates catesbeianus* | -76.702 | 38.78542 |
| *Lithobates catesbeianus* | -122.489 | 47.44756 |
| *Lithobates catesbeianus* | -95.5977 | 29.37544 |
| *Lithobates catesbeianus* | -118.411 | 34.12219 |
| *Lithobates catesbeianus* | -94.4431 | 30.3858 |
| *Lithobates catesbeianus* | -122.745 | 38.52676 |
| *Lithobates catesbeianus* | -118.41 | 34.12008 |
| *Lithobates catesbeianus* | -81.1804 | 35.81742 |
| *Lithobates catesbeianus* | -76.5967 | 39.24357 |
| *Lithobates catesbeianus* | -65.061 | 45.62178 |
| *Lithobates catesbeianus* | -123.399 | 46.23188 |
| *Lithobates catesbeianus* | -74.3105 | 42.08562 |
| *Lithobates catesbeianus* | -74.5319 | 40.5967 |
| *Lithobates catesbeianus* | -82.7773 | 35.0648 |
| *Lithobates catesbeianus* | -76.5283 | 42.41533 |
| *Lithobates catesbeianus* | -81.3902 | 36.5041 |
| *Lithobates catesbeianus* | 5.12893 | 51.14381 |
| *Lithobates catesbeianus* | 5.13023 | 51.14511 |
| *Lithobates catesbeianus* | -122.143 | 37.93387 |
| *Lithobates catesbeianus* | -122.658 | 37.95576 |
| *Lithobates catesbeianus* | -122.65 | 37.95828 |
| *Lithobates catesbeianus* | -76.2967 | 3.98474 |
| *Lithobates catesbeianus* | -81.9923 | 29.1833 |
| *Lithobates catesbeianus* | -81.3923 | 26.95357 |
| *Lithobates catesbeianus* | -122.579 | 48.83222 |
| *Lithobates catesbeianus* | -72.1025 | 44.71477 |
| *Lithobates catesbeianus* | -97.7761 | 30.28075 |
| *Lithobates catesbeianus* | -87.65 | 41.85 |
| *Lithobates catesbeianus* | -94.6369 | 37.0409 |
| *Lithobates catesbeianus* | -99.3066 | 38.3722 |
| *Lithobates catesbeianus* | -100.082 | 38.04914 |
| *Lithobates catesbeianus* | -97.1625 | 39.90809 |
| *Lithobates catesbeianus* | -94.6856 | 37.1647 |
| *Lithobates catesbeianus* | -84.4684 | 39.52743 |
| *Lithobates catesbeianus* | -84.1305 | 39.43903 |
| *Lithobates catesbeianus* | -84.7907 | 39.37324 |
| *Lithobates catesbeianus* | -82.2557 | 39.70444 |
| *Lithobates catesbeianus* | -115.671 | 32.6789 |
| *Lithobates catesbeianus* | -112.896 | 27.28194 |
| *Lithobates catesbeianus* | -106.592 | 31.83292 |
| *Lithobates catesbeianus* | -120.551 | 34.77686 |
| *Lithobates catesbeianus* | -80.7681 | 40.772 |
| *Lithobates catesbeianus* | -82.4913 | 39.9327 |
| *Lithobates catesbeianus* | -81.8721 | 40.2968 |
| *Lithobates catesbeianus* | -80.8827 | 41.2396 |
| *Lithobates catesbeianus* | -96.6354 | 28.553 |
| *Lithobates catesbeianus* | -96.2158 | 30.56225 |
| *Lithobates catesbeianus* | -97.0724 | 29.34483 |
| *Lithobates catesbeianus* | -82.5944 | 31.83139 |
| *Lithobates catesbeianus* | -96.4689 | 30.10587 |
| *Lithobates catesbeianus* | -94.9735 | 29.90433 |
| *Lithobates catesbeianus* | -95.7219 | 29.05917 |
| *Lithobates catesbeianus* | -95.8929 | 31.94131 |
| *Lithobates catesbeianus* | -122 | 39.20933 |
| *Lithobates catesbeianus* | -120.312 | 37.96659 |
| *Lithobates catesbeianus* | -118.216 | 35.83467 |
| *Lithobates catesbeianus* | -109.045 | 32.06342 |
| *Lithobates catesbeianus* | -109.258 | 31.3751 |
| *Lithobates catesbeianus* | -115.116 | 37.27524 |
| *Lithobates catesbeianus* | -122.65 | 45.3383 |
| *Lithobates catesbeianus* | -114.551 | 32.7172 |
| *Lithobates catesbeianus* | -114.153 | 34.3542 |
| *Lithobates catesbeianus* | -114.125 | 34.2939 |
| *Lithobates catesbeianus* | -114.323 | 34.4839 |
| *Lithobates catesbeianus* | -111.665 | 34.2864 |
| *Lithobates catesbeianus* | -65.9144 | 18.17283 |
| *Lithobates catesbeianus* | -94.3735 | 46.89721 |
| *Lithobates catesbeianus* | -74.6453 | 39.6615 |
| *Lithobates catesbeianus* | -95.0983 | 43.4408 |
| *Lithobates catesbeianus* | -95.1893 | 43.3431 |
| *Lithobates catesbeianus* | -79.17 | 38.3 |
| *Lithobates catesbeianus* | -83.2547 | 35.08008 |
| *Lithobates catesbeianus* | -78.52 | 34.7 |
| *Lithobates catesbeianus* | -76.9661 | 38.9104 |
| *Lithobates catesbeianus* | -82.2465 | 35.4393 |
| *Lithobates catesbeianus* | -70.4225 | 18.97263 |
| *Lithobates catesbeianus* | -84.5881 | 36.6228 |
| *Lithobates catesbeianus* | -77.08 | 38.7 |
| *Lithobates catesbeianus* | -93.2819 | 32.42546 |
| *Lithobates catesbeianus* | -83.2094 | 31.8428 |
| *Lithobates catesbeianus* | -91.8595 | 32.4864 |
| *Lithobates catesbeianus* | -95.095 | 34.9993 |
| *Lithobates catesbeianus* | -94.6933 | 36.65197 |
| *Lithobates catesbeianus* | -95.0484 | 36.38079 |
| *Lithobates catesbeianus* | -95.3072 | 35.006 |
| *Lithobates catesbeianus* | -96.5661 | 36.68085 |
| *Lithobates catesbeianus* | -95.8581 | 34.95039 |
| *Lithobates catesbeianus* | -97.9425 | 35.32476 |
| *Lithobates catesbeianus* | -98.7732 | 33.194 |
| *Lithobates catesbeianus* | -73.9803 | 40.9836 |
| *Lithobates catesbeianus* | -98.1299 | 36.10824 |
| *Lithobates catesbeianus* | -95.5016 | 33.87701 |
| *Lithobates catesbeianus* | -82.2454 | 35.43058 |
| *Lithobates catesbeianus* | -78.267 | 34.3776 |
| *Lithobates catesbeianus* | -78.1487 | 34.006 |
| *Lithobates catesbeianus* | -78.3624 | 34.1666 |
| *Lithobates catesbeianus* | -76.4625 | 35.7682 |
| *Lithobates catesbeianus* | -78.9344 | 36.0719 |
| *Lithobates catesbeianus* | -78.025 | 35.9681 |
| *Lithobates catesbeianus* | -74.7828 | 39.1197 |
| *Lithobates catesbeianus* | -75.3135 | 39.5896 |
| *Lithobates catesbeianus* | -77.3144 | 34.6237 |
| *Lithobates catesbeianus* | -77.5294 | 37.7047 |
| *Lithobates catesbeianus* | -77.5281 | 37.5281 |
| *Lithobates catesbeianus* | -80.5696 | 35.3647 |
| *Lithobates catesbeianus* | -81.626 | 36.9301 |
| *Lithobates catesbeianus* | -76.6959 | 38.94324 |
| *Lithobates catesbeianus* | -95.2479 | 38.95942 |
| *Lithobates catesbeianus* | -95.2459 | 38.95748 |
| *Lithobates catesbeianus* | -100.47 | 37.16971 |
| *Lithobates catesbeianus* | -100.933 | 39.87599 |
| *Lithobates catesbeianus* | -85.6023 | 36.36044 |
| *Lithobates catesbeianus* | -84.1669 | 39.02218 |
| *Lithobates catesbeianus* | -84.6835 | 39.27211 |
| *Lithobates catesbeianus* | -99.3433 | 37.26386 |
| *Lithobates catesbeianus* | -94.8479 | 38.84776 |
| *Lithobates catesbeianus* | -95.4287 | 38.8739 |
| *Lithobates catesbeianus* | -96.3713 | 38.49205 |
| *Lithobates catesbeianus* | -99.3183 | 39.39746 |
| *Lithobates catesbeianus* | -120.333 | 37.70972 |
| *Lithobates catesbeianus* | -123.186 | 39.83631 |
| *Lithobates catesbeianus* | -96.217 | 30.62769 |
| *Lithobates catesbeianus* | -108.64 | 25.67031 |
| *Lithobates catesbeianus* | -117.189 | 34.24619 |
| *Lithobates catesbeianus* | -106.825 | 32.41064 |
| *Lithobates catesbeianus* | -115.834 | 33.3334 |
| *Lithobates catesbeianus* | -116.515 | 33.88961 |
| *Lithobates catesbeianus* | -114.75 | 33.406 |
| *Lithobates catesbeianus* | -113.17 | 26.88024 |
| *Lithobates catesbeianus* | 139.046 | 36.4722 |
| *Lithobates catesbeianus* | -120.501 | 34.66778 |
| *Lithobates catesbeianus* | -120.491 | 34.67194 |
| *Lithobates catesbeianus* | -75.45 | 45.467 |
| *Lithobates catesbeianus* | -73.8 | 45.6 |
| *Lithobates catesbeianus* | -75.417 | 45.7 |
| *Lithobates catesbeianus* | -76 | 45.417 |
| *Lithobates catesbeianus* | -76.013 | 45.636 |
| *Lithobates catesbeianus* | -81.537 | 45.212 |
| *Lithobates catesbeianus* | -81.57 | 45.194 |
| *Lithobates catesbeianus* | -80.9 | 46.517 |
| *Lithobates catesbeianus* | -72.526 | 45.575 |
| *Lithobates catesbeianus* | -76.199 | 45.894 |
| *Lithobates catesbeianus* | -76.183 | 45.217 |
| *Lithobates catesbeianus* | -75.8 | 45.5 |
| *Lithobates catesbeianus* | -79.3496 | 38.60001 |
| *Lithobates catesbeianus* | -80.38 | 41.3 |
| *Lithobates catesbeianus* | -80.08 | 41.06 |
| *Lithobates catesbeianus* | -81.0797 | 38.46001 |
| *Lithobates catesbeianus* | -81.09 | 32.08 |
| *Lithobates catesbeianus* | -75.47 | 40.08 |
| *Lithobates catesbeianus* | -75.1 | 40.21 |
| *Lithobates catesbeianus* | -155.24 | 19.71 |
| *Lithobates catesbeianus* | -79.9 | 40.43 |
| *Lithobates catesbeianus* | -82.21 | 37.28 |
| *Lithobates catesbeianus* | -76.56 | 36.7 |
| *Lithobates catesbeianus* | -80.55 | 37.11 |
| *Lithobates catesbeianus* | -76.5 | 37.1 |
| *Lithobates catesbeianus* | -81.0869 | 41.4879 |
| *Lithobates catesbeianus* | -122.713 | 47.07271 |
| *Lithobates catesbeianus* | -81 | 25.7739 |
| *Lithobates catesbeianus* | -73.1291 | 41.25268 |
| *Lithobates catesbeianus* | -72.5132 | 41.50533 |
| *Lithobates catesbeianus* | -75.38 | 39.92 |
| *Lithobates catesbeianus* | -101.967 | 35.86253 |
| *Lithobates catesbeianus* | -81.1912 | 22.83883 |
| *Lithobates catesbeianus* | -119.68 | 37.38552 |
| *Lithobates catesbeianus* | -95.4608 | 29.84674 |
| *Lithobates catesbeianus* | -118.411 | 34.11938 |
| *Lithobates catesbeianus* | -101.79 | 37.12417 |
| *Lithobates catesbeianus* | -122.209 | 37.85972 |
| *Lithobates catesbeianus* | -79.0566 | 35.83849 |
| *Lithobates catesbeianus* | -97.1304 | 33.0605 |
| *Lithobates catesbeianus* | -97.4865 | 35.63755 |
| *Lithobates catesbeianus* | -116.812 | 32.60738 |
| *Lithobates catesbeianus* | -107.923 | 32.5517 |
| *Lithobates catesbeianus* | -75.502 | 45.49898 |
| *Lithobates catesbeianus* | -97.8317 | 30.27164 |
| *Lithobates catesbeianus* | -97.5097 | 32.73844 |
| *Lithobates catesbeianus* | -74.6585 | 40.2328 |
| *Lithobates catesbeianus* | -123.967 | 49.17327 |
| *Lithobates catesbeianus* | -95.9121 | 36.14958 |
| *Lithobates catesbeianus* | -122.979 | 49.2306 |
| *Lithobates catesbeianus* | -75.4863 | 45.50194 |
| *Lithobates catesbeianus* | -75.8567 | 44.47856 |
| *Lithobates catesbeianus* | -88.1248 | 41.8729 |
| *Lithobates catesbeianus* | -70.3014 | 43.68263 |
| *Lithobates catesbeianus* | -76.5807 | 39.28867 |
| *Lithobates catesbeianus* | -121.442 | 38.08895 |
| *Lithobates catesbeianus* | -91.7476 | 30.27971 |
| *Lithobates catesbeianus* | 126.3466 | 36.72943 |
| *Lithobates catesbeianus* | -122.489 | 47.44729 |
| *Lithobates catesbeianus* | 7.77453 | 44.90778 |
| *Lithobates catesbeianus* | -92.4135 | 38.83336 |
| *Lithobates catesbeianus* | -97.8829 | 30.58409 |
| *Lithobates catesbeianus* | -91.8355 | 37.53362 |
| *Lithobates catesbeianus* | -122.826 | 38.69453 |
| *Lithobates catesbeianus* | -95.4453 | 29.01632 |
| *Lithobates catesbeianus* | -72.6094 | 41.75696 |
| *Lithobates catesbeianus* | -122.652 | 38.44985 |
| *Lithobates catesbeianus* | -99.8508 | 32.26456 |
| *Lithobates catesbeianus* | -73.1776 | 44.01912 |
| *Lithobates catesbeianus* | -82.5562 | 40.69931 |
| *Lithobates catesbeianus* | -88.1726 | 41.86284 |
| *Lithobates catesbeianus* | -121.721 | 38.7997 |
| *Lithobates catesbeianus* | -98.0082 | 32.79435 |
| *Lithobates catesbeianus* | -122.653 | 38.45934 |
| *Lithobates catesbeianus* | -66.6254 | 45.66256 |
| *Lithobates catesbeianus* | -72.5621 | 41.31402 |
| *Lithobates catesbeianus* | -75.4964 | 45.50275 |
| *Lithobates catesbeianus* | -75.485 | 45.49956 |
| *Lithobates catesbeianus* | -73.3576 | 43.68322 |
| *Lithobates catesbeianus* | -83.2171 | 39.91355 |
| *Lithobates catesbeianus* | -74.3138 | 41.95373 |
| *Lithobates catesbeianus* | -82.6559 | 38.50569 |
| *Lithobates catesbeianus* | -90.075 | 29.9544 |
| *Lithobates catesbeianus* | -72.9475 | 41.2706 |
| *Lithobates catesbeianus* | -75.7998 | 36.2739 |
| *Lithobates catesbeianus* | -79.6517 | 43.025 |
| *Lithobates catesbeianus* | -74.7663 | 40.35844 |
| *Lithobates catesbeianus* | -120.347 | 39.7629 |
| *Lithobates catesbeianus* | -123.948 | 46.15025 |
| *Lithobates catesbeianus* | -122.666 | 37.95762 |
| *Lithobates catesbeianus* | -76.4222 | 3.70641 |
| *Lithobates catesbeianus* | -82.5561 | 35.63291 |
| *Lithobates catesbeianus* | 5.13025 | 51.14472 |
| *Lithobates catesbeianus* | 5.12858 | 51.14352 |
| *Lithobates catesbeianus* | -97.6452 | 30.68496 |
| *Lithobates catesbeianus* | -98.397 | 21.067 |
| *Lithobates catesbeianus* | -82.3075 | 29.6667 |
| *Lithobates catesbeianus* | -85.2798 | 29.77702 |
| *Lithobates catesbeianus* | -82.017 | 29.56441 |
| *Lithobates catesbeianus* | -84.6419 | 34.23313 |
| *Lithobates catesbeianus* | -82.3434 | 29.88067 |
| *Lithobates catesbeianus* | -97.4361 | 39.35746 |
| *Lithobates catesbeianus* | -96.3964 | 38.73341 |
| *Lithobates catesbeianus* | -94.8534 | 38.67248 |
| *Lithobates catesbeianus* | -81.8115 | 36.1112 |
| *Lithobates catesbeianus* | -95.0695 | 37.79194 |
| *Lithobates catesbeianus* | -95.1924 | 38.69757 |
| *Lithobates catesbeianus* | -96.5626 | 38.15805 |
| *Lithobates catesbeianus* | -95.3373 | 39.04656 |
| *Lithobates catesbeianus* | -99.7813 | 39.38645 |
| *Lithobates catesbeianus* | -83.0012 | 39.97931 |
| *Lithobates catesbeianus* | -90.1124 | 31.55147 |
| *Lithobates catesbeianus* | -76.9554 | 38.91176 |
| *Lithobates catesbeianus* | -83.9439 | 31.9386 |
| *Lithobates catesbeianus* | -70.4272 | 18.97291 |
| *Lithobates catesbeianus* | -77.2583 | 37.82958 |
| *Lithobates catesbeianus* | -83.0134 | 39.97031 |
| *Lithobates catesbeianus* | -82.43 | 23.0767 |
| *Lithobates catesbeianus* | -111.673 | 33.56357 |
| *Lithobates catesbeianus* | -76.2086 | 36.6322 |
| *Lithobates catesbeianus* | -76.3861 | 36.5853 |
| *Lithobates catesbeianus* | -77.4971 | 37.30892 |
| *Lithobates catesbeianus* | -80.2911 | 36.857 |
| *Lithobates catesbeianus* | -81.4919 | 26.9844 |
| *Lithobates catesbeianus* | -77.2342 | 35.6731 |
| *Lithobates catesbeianus* | -99.6998 | 35.70015 |
| *Lithobates catesbeianus* | -94.7081 | 35.49459 |
| *Lithobates catesbeianus* | -98.4407 | 36.40723 |
| *Lithobates catesbeianus* | -95.336 | 35.1008 |
| *Lithobates catesbeianus* | -94.7011 | 34.9323 |
| *Lithobates catesbeianus* | -96.968 | 34.74688 |
| *Lithobates catesbeianus* | -97.0679 | 36.46374 |
| *Lithobates catesbeianus* | -94.7581 | 33.94706 |
| *Lithobates catesbeianus* | -95.5397 | 36.77176 |
| *Lithobates catesbeianus* | -95.3529 | 34.99554 |
| *Lithobates catesbeianus* | -83.1617 | 35.0446 |
| *Lithobates catesbeianus* | -78.565 | 34.8128 |
| *Lithobates catesbeianus* | -78.0726 | 33.9181 |
| *Lithobates catesbeianus* | -78.4441 | 35.3836 |
| *Lithobates catesbeianus* | -78.4038 | 34.1186 |
| *Lithobates catesbeianus* | -89.902 | 31.9141 |
| *Lithobates catesbeianus* | -76.9816 | 34.7176 |
| *Lithobates catesbeianus* | -83.2569 | 35.0316 |
| *Lithobates catesbeianus* | -79.1805 | 34.8859 |
| *Lithobates catesbeianus* | -75.5016 | 39.4631 |
| *Lithobates catesbeianus* | -75.0634 | 39.5556 |
| *Lithobates catesbeianus* | -75.2671 | 39.682 |
| *Lithobates catesbeianus* | -79.472 | 35.1102 |
| *Lithobates catesbeianus* | -76.4356 | 36.8986 |
| *Lithobates catesbeianus* | -78.6314 | 36.6619 |
| *Lithobates catesbeianus* | -76.9072 | 37.3213 |
| *Lithobates catesbeianus* | -77.9277 | 38.8012 |
| *Lithobates catesbeianus* | -75.2374 | 38.7913 |
| *Lithobates catesbeianus* | -75.6946 | 4.81428 |
| *Lithobates catesbeianus* | -88.4568 | 41.61543 |
| *Lithobates catesbeianus* | -121.906 | 47.77692 |
| *Lithobates catesbeianus* | -117.043 | 32.84093 |
| *Lithobates catesbeianus* | -72.25 | 45.283 |
| *Lithobates catesbeianus* | -82.5 | 41.95 |
| *Lithobates catesbeianus* | -76.067 | 46.167 |
| *Lithobates catesbeianus* | -75.317 | 45.25 |
| *Lithobates catesbeianus* | -75.8 | 45.617 |
| *Lithobates catesbeianus* | -75.938 | 45.118 |
| *Lithobates catesbeianus* | -74.59 | 45.148 |
| *Lithobates catesbeianus* | -81.167 | 44.865 |
| *Lithobates catesbeianus* | -75.643 | 45.648 |
| *Lithobates catesbeianus* | -76.599 | 45.017 |
| *Lithobates catesbeianus* | -75.647 | 44.878 |
| *Lithobates catesbeianus* | -82.5 | 41.933 |
| *Lithobates catesbeianus* | -73.933 | 45.367 |
| *Lithobates catesbeianus* | -75.85 | 45.483 |
| *Lithobates catesbeianus* | -79.88 | 40.51 |
| *Lithobates catesbeianus* | -79.22 | 41.84 |
| *Lithobates catesbeianus* | -78.86 | 39.96 |
| *Lithobates catesbeianus* | -109.83 | 34.07 |
| *Lithobates catesbeianus* | -111.7 | 34.07 |
| *Lithobates catesbeianus* | -95.8484 | 38.39018 |
| *Lithobates catesbeianus* | -79.27 | 40.14 |
| *Lithobates catesbeianus* | -81.41 | 31.96 |
| *Lithobates catesbeianus* | -82.12 | 37.34 |
| *Lithobates catesbeianus* | -80 | 36.61 |
| *Lithobates catesbeianus* | -78.17 | 37.73 |
| *Lithobates catesbeianus* | -76.88 | 37.41 |
| *Lithobates catesbeianus* | -119.041 | 46.2001 |
| *Lithobates catesbeianus* | -79.8671 | 41.32596 |
| *Lithobates catesbeianus* | -117.458 | 33.48909 |
| *Lithobates catesbeianus* | -121.873 | 37.19302 |
| *Lithobates catesbeianus* | -121.923 | 37.29988 |
| *Lithobates catesbeianus* | -120.809 | 39.90989 |
| *Lithobates catesbeianus* | -122.438 | 37.47528 |
| *Lithobates catesbeianus* | -99.6132 | 38.93496 |
| *Lithobates catesbeianus* | -114.635 | 32.82676 |
| *Lithobates catesbeianus* | -118.055 | 33.85102 |
| *Lithobates catesbeianus* | -119.319 | 34.36075 |
| *Lithobates catesbeianus* | -118.361 | 33.35301 |
| *Lithobates catesbeianus* | -117.817 | 33.85569 |
| *Lithobates catesbeianus* | -120.585 | 34.68901 |
| *Lithobates catesbeianus* | -116.383 | 38.2 |
| *Lithobates catesbeianus* | -88.2252 | 45.2812 |
| *Lithobates catesbeianus* | -75.6429 | 45.01687 |
| *Lithobates catesbeianus* | -110.119 | 31.48022 |
| *Lithobates catesbeianus* | -109.141 | 31.9137 |
| *Lithobates catesbeianus* | -119.617 | 35.7458 |
| *Lithobates catesbeianus* | -122.711 | 47.0825 |
| *Lithobates catesbeianus* | -114.443 | 32.8492 |
| *Lithobates catesbeianus* | -117.063 | 32.90462 |
| *Lithobates catesbeianus* | -93.3624 | 44.97133 |
| *Lithobates catesbeianus* | -95.4675 | 29.03389 |
| *Lithobates catesbeianus* | -97.6625 | 31.15744 |
| *Lithobates catesbeianus* | -96.3408 | 30.63306 |
| *Lithobates catesbeianus* | -96.1318 | 32.41421 |
| *Lithobates catesbeianus* | -98.3154 | 33.57515 |
| *Lithobates catesbeianus* | -94.3944 | 33.32611 |
| *Lithobates catesbeianus* | -98.0548 | 32.81086 |
| *Lithobates catesbeianus* | -98.4536 | 29.3275 |
| *Lithobates catesbeianus* | -72.7498 | 44.0003 |
| *Lithobates catesbeianus* | -120.569 | 34.67921 |
| *Lithobates catesbeianus* | -84.1391 | 39.95708 |
| *Lithobates catesbeianus* | -82.7866 | 39.3966 |
| *Lithobates catesbeianus* | -111.09 | 31.4009 |
| *Lithobates catesbeianus* | -83.709 | 39.98278 |
| *Lithobates catesbeianus* | -94.8825 | 36.9422 |
| *Lithobates catesbeianus* | -98.4836 | 37.47095 |
| *Lithobates catesbeianus* | -89.3141 | 36.67268 |
| *Lithobates catesbeianus* | -108.729 | 39.15879 |
| *Lithobates catesbeianus* | -121.932 | 39.7283 |
| *Lithobates catesbeianus* | -120.939 | 37.05675 |
| *Lithobates catesbeianus* | -95.6181 | 29.37397 |
| *Lithobates catesbeianus* | -81.5859 | 41.31891 |
| *Lithobates catesbeianus* | -65.12 | 43.71923 |
| *Lithobates catesbeianus* | -75.9336 | 38.95283 |
| *Lithobates catesbeianus* | -108.88 | 33.32099 |
| *Lithobates catesbeianus* | -81.8686 | 40.46313 |
| *Lithobates catesbeianus* | -118.427 | 33.96653 |
| *Lithobates catesbeianus* | -75.4105 | 39.08632 |
| *Lithobates catesbeianus* | -120.66 | 35.29968 |
| *Lithobates catesbeianus* | -105.779 | 36.29367 |
| *Lithobates catesbeianus* | -83.7001 | 37.71377 |
| *Lithobates catesbeianus* | -89.8164 | 39.91945 |
| *Lithobates catesbeianus* | -121.779 | 37.34484 |
| *Lithobates catesbeianus* | -107.758 | 32.26861 |
| *Lithobates catesbeianus* | -71.2385 | 42.50109 |
| *Lithobates catesbeianus* | -88.1436 | 41.82556 |
| *Lithobates catesbeianus* | -124.093 | 49.24254 |
| *Lithobates catesbeianus* | -95.6655 | 29.58822 |
| *Lithobates catesbeianus* | -96.7613 | 28.64991 |
| *Lithobates catesbeianus* | -93.7514 | 30.14248 |
| *Lithobates catesbeianus* | -116.901 | 33.33972 |
| *Lithobates catesbeianus* | -65.8442 | 18.2026 |
| *Lithobates catesbeianus* | -97.6769 | 37.53692 |
| *Lithobates catesbeianus* | -95.215 | 29.92948 |
| *Lithobates catesbeianus* | -72.2262 | 41.7794 |
| *Lithobates catesbeianus* | -71.8166 | 44.79155 |
| *Lithobates catesbeianus* | -97.7118 | 29.7401 |
| *Lithobates catesbeianus* | -123.84 | 46.14603 |
| *Lithobates catesbeianus* | -92.323 | 39.13546 |
| *Lithobates catesbeianus* | -71.1844 | 42.40475 |
| *Lithobates catesbeianus* | -122.654 | 38.45844 |
| *Lithobates catesbeianus* | -88.0691 | 41.63902 |
| *Lithobates catesbeianus* | -77.1039 | 38.75508 |
| *Lithobates catesbeianus* | -94.3905 | 29.57466 |
| *Lithobates catesbeianus* | -123.721 | 39.87097 |
| *Lithobates catesbeianus* | -76.3381 | 36.63271 |
| *Lithobates catesbeianus* | -72.7031 | 41.8986 |
| *Lithobates catesbeianus* | -94.5555 | 29.60208 |
| *Lithobates catesbeianus* | -118.828 | 34.12809 |
| *Lithobates catesbeianus* | -122.694 | 49.29337 |
| *Lithobates catesbeianus* | -84.6373 | 39.37131 |
| *Lithobates catesbeianus* | -115.267 | 36.32416 |
| *Lithobates catesbeianus* | -84.0104 | 39.7395 |
| *Lithobates catesbeianus* | -99.6519 | 28.99947 |
| *Lithobates catesbeianus* | -74.991 | 39.67479 |
| *Lithobates catesbeianus* | -121.695 | 37.22159 |
| *Lithobates catesbeianus* | -73.1779 | 44.01906 |
| *Lithobates catesbeianus* | -75.4884 | 45.5021 |
| *Lithobates catesbeianus* | -120.053 | 38.93543 |
| *Lithobates catesbeianus* | -72.5029 | 44.32923 |
| *Lithobates catesbeianus* | -97.182 | 30.11024 |
| *Lithobates catesbeianus* | -117.034 | 32.8413 |
| *Lithobates catesbeianus* | -76.4756 | 42.42621 |
| *Lithobates catesbeianus* | -81.712 | 38.3555 |
| *Lithobates catesbeianus* | -51.225 | -27.4017 |
| *Lithobates catesbeianus* | -51.9386 | -23.4253 |
| *Lithobates catesbeianus* | -49.0769 | -25.3656 |
| *Lithobates catesbeianus* | -45.07 | -23.43 |
| *Lithobates catesbeianus* | -51.74 | -27.0067 |
| *Lithobates catesbeianus* | -49.0667 | -26.4861 |
| *Lithobates catesbeianus* | -51.1794 | -29.1681 |
| *Lithobates catesbeianus* | -51.4956 | -29.3769 |
| *Lithobates catesbeianus* | -53.8608 | -27.2647 |
| *Lithobates catesbeianus* | -51.23 | -30.0331 |
| *Lithobates catesbeianus* | -50.9919 | -29.9444 |
| *Lithobates catesbeianus* | -53.7583 | -27.3711 |
| *Lithobates catesbeianus* | -51.1144 | -29.3764 |
| *Lithobates catesbeianus* | -51.7181 | -29.9433 |
| *Lithobates catesbeianus* | -53.3944 | -27.3592 |
| *Lithobates catesbeianus* | -51.9614 | -29.4669 |
| *Lithobates catesbeianus* | -48.8236 | -27.6939 |
| *Lithobates catesbeianus* | -52.7714 | -27.3617 |
| *Lithobates catesbeianus* | -51.8675 | -29.2842 |
| *Lithobates catesbeianus* | -50.6953 | -26.9289 |
| *Lithobates catesbeianus* | -52.5347 | -26.9617 |
| *Lithobates catesbeianus* | -51.5494 | -28.9361 |
| *Lithobates catesbeianus* | -51.5333 | -28.8628 |
| *Lithobates catesbeianus* | -50.3261 | -27.8161 |
| *Lithobates catesbeianus* | -49.0661 | -26.9194 |
| *Lithobates catesbeianus* | -53.4447 | -29.5747 |
| *Lithobates catesbeianus* | -53.4689 | -29.4717 |
| *Lithobates catesbeianus* | -52.9919 | -26.8481 |
| *Lithobates catesbeianus* | -53.5806 | -29.5203 |
| *Lithobates catesbeianus* | -53.3572 | -29.6217 |
| *Lithobates catesbeianus* | -44.1983 | -19.9678 |
| *Lithobates catesbeianus* | -45.4303 | -21.5514 |
| *Lithobates catesbeianus* | -51.4581 | -25.3953 |
| *Lithobates catesbeianus* | -48.59 | -24.58 |
| *Lithobates catesbeianus* | -47.41 | -22.75 |
| *Lithobates catesbeianus* | -49.1769 | -26.7406 |
| *Lithobates catesbeianus* | -47.13 | -23.52 |
| *Lithobates catesbeianus* | -45.66 | -23.38 |
| *Lithobates catesbeianus* | -48.36 | -21.6 |
| *Lithobates catesbeianus* | -48.8236 | -27.6939 |
| *Lithobates catesbeianus* | -53.2499 | -29.6416 |
| *Lithobates catesbeianus* | -53.2798 | -29.4988 |
| *Lithobates catesbeianus* | -53.2792 | -29.4821 |
| *Lithobates catesbeianus* | -41.5331 | -20.7636 |
| *Lithobates catesbeianus* | -48.9528 | -16.3267 |
| *Lithobates catesbeianus* | -49.2439 | -16.8233 |
| *Lithobates catesbeianus* | -53.0961 | -29.3387 |
| *Lithobates catesbeianus* | -51.5346 | -29.3793 |
| *Lithobates catesbeianus* | -51.4544 | -27.677 |
| *Lithobates catesbeianus* | -48.5044 | -1.4558 |
| *Lithobates catesbeianus* | -49.0654 | -26.9203 |
| *Lithobates catesbeianus* | -49.119 | -27.003 |
| *Lithobates catesbeianus* | -49.1117 | -27.0368 |
| *Lithobates catesbeianus* | -49.1125 | -27.0373 |
| *Lithobates catesbeianus* | -49.0937 | -27.0304 |
| *Lithobates catesbeianus* | -49.0863 | -27.0532 |
| *Lithobates catesbeianus* | -49.0866 | -27.0538 |
| *Lithobates catesbeianus* | -48.0106 | -16.0353 |
| *Lithobates catesbeianus* | -51.2021 | -26.7534 |
| *Lithobates catesbeianus* | -46.1444 | -22.7556 |
| *Lithobates catesbeianus* | -47.0638 | -22.8115 |
| *Lithobates catesbeianus* | -45.5801 | -22.7305 |
| *Lithobates catesbeianus* | -51.2486 | -27.384 |
| *Lithobates catesbeianus* | -53.6831 | -31.482 |
| *Lithobates catesbeianus* | -50.8019 | -29.3599 |
| *Lithobates catesbeianus* | -42.14 | -19.79 |
| *Lithobates catesbeianus* | -51.7 | -29.0167 |
| *Lithobates catesbeianus* | -51.1675 | -29.1784 |
| *Lithobates catesbeianus* | -52.6194 | -27.1857 |
| *Lithobates catesbeianus* | -52.6647 | -27.1449 |
| *Lithobates catesbeianus* | -52.6529 | -27.1434 |
| *Lithobates catesbeianus* | -52.6558 | -27.1417 |
| *Lithobates catesbeianus* | -41.9833 | -4.53333 |
| *Lithobates catesbeianus* | -42.8 | -20.8667 |
| *Lithobates catesbeianus* | -54.0728 | -28.3814 |
| *Lithobates catesbeianus* | -49.2818 | -26.4327 |
| *Lithobates catesbeianus* | -46.9525 | -23.7441 |
| *Lithobates catesbeianus* | -49.4333 | -25.7167 |
| *Lithobates catesbeianus* | -53.8553 | -27.2663 |
| *Lithobates catesbeianus* | -51.85 | -28.9833 |
| *Lithobates catesbeianus* | -52.1107 | -30.6979 |
| *Lithobates catesbeianus* | -53.359 | -29.6263 |
| *Lithobates catesbeianus* | -53.3534 | -29.612 |
| *Lithobates catesbeianus* | -53.3535 | -29.6119 |
| *Lithobates catesbeianus* | -53.377 | -29.5937 |
| *Lithobates catesbeianus* | -53.3364 | -29.6122 |
| *Lithobates catesbeianus* | -53.3386 | -29.6075 |
| *Lithobates catesbeianus* | -53.3444 | -29.5706 |
| *Lithobates catesbeianus* | -43.3045 | -22.5824 |
| *Lithobates catesbeianus* | -46.8319 | -23.2364 |
| *Lithobates catesbeianus* | -51.8799 | -29.2398 |
| *Lithobates catesbeianus* | -52.733 | -27.5286 |
| *Lithobates catesbeianus* | -52.2689 | -27.6296 |
| *Lithobates catesbeianus* | -50.98 | -28.1 |
| *Lithobates catesbeianus* | -53.1537 | -29.1723 |
| *Lithobates catesbeianus* | -53.4268 | -29.573 |
| *Lithobates catesbeianus* | -53.4267 | -29.5735 |
| *Lithobates catesbeianus* | -53.4234 | -29.5664 |
| *Lithobates catesbeianus* | -53.4231 | -29.58 |
| *Lithobates catesbeianus* | -53.3988 | -29.5305 |
| *Lithobates catesbeianus* | -53.3984 | -29.5305 |
| *Lithobates catesbeianus* | -49.25 | -25.6167 |
| *Lithobates catesbeianus* | -38.5 | -3.7167 |
| *Lithobates catesbeianus* | -53.0991 | -26.065 |
| *Lithobates catesbeianus* | -53.4 | -27.3667 |
| *Lithobates catesbeianus* | -49.2667 | -16.6667 |
| *Lithobates catesbeianus* | -50.99 | -29.95 |
| *Lithobates catesbeianus* | -51.32 | -30.11 |
| *Lithobates catesbeianus* | -51.45 | -25.3833 |
| *Lithobates catesbeianus* | -52.7798 | -27.0889 |
| *Lithobates catesbeianus* | -52.7523 | -27.14 |
| *Lithobates catesbeianus* | -52.7519 | -27.163 |
| *Lithobates catesbeianus* | -49.05 | -17.2667 |
| *Lithobates catesbeianus* | -53.1269 | -29.4211 |
| *Lithobates catesbeianus* | -50.8 | -29.58 |
| *Lithobates catesbeianus* | -49.2559 | -26.9318 |
| *Lithobates catesbeianus* | -49.2662 | -26.9469 |
| *Lithobates catesbeianus* | -49.2657 | -26.9468 |
| *Lithobates catesbeianus* | -49.2652 | -26.9478 |
| *Lithobates catesbeianus* | -48.5833 | -24.5833 |
| *Lithobates catesbeianus* | -52.4081 | -26.5707 |
| *Lithobates catesbeianus* | -40.8753 | -19.8739 |
| *Lithobates catesbeianus* | -43.5833 | -20.5 |
| *Lithobates catesbeianus* | -53.5273 | -29.4824 |
| *Lithobates catesbeianus* | -53.5305 | -29.4985 |
| *Lithobates catesbeianus* | -53.5305 | -29.492 |
| *Lithobates catesbeianus* | -49.1 | -26.48 |
| *Lithobates catesbeianus* | -51.5243 | -27.1589 |
| *Lithobates catesbeianus* | -51.5889 | -27.1609 |
| *Lithobates catesbeianus* | -50.3167 | -27.8 |
| *Lithobates catesbeianus* | -51.1269 | -29.4211 |
| *Lithobates catesbeianus* | -50.6667 | -26.8536 |
| *Lithobates catesbeianus* | -35.35 | -5.85 |
| *Lithobates catesbeianus* | -35.7451 | -9.62554 |
| *Lithobates catesbeianus* | -42.1375 | -20.2105 |
| *Lithobates catesbeianus* | -52.2039 | -28.4467 |
| *Lithobates catesbeianus* | -49.9458 | -22.2139 |
| *Lithobates catesbeianus* | -51.9372 | -23.4277 |
| *Lithobates catesbeianus* | -52.1874 | -28.2787 |
| *Lithobates catesbeianus* | -51.8693 | -29.165 |
| *Lithobates catesbeianus* | -51.1833 | -28.3167 |
| *Lithobates catesbeianus* | -52.7724 | -27.3632 |
| *Lithobates catesbeianus* | -52.8998 | -26.8697 |
| *Lithobates catesbeianus* | -52.9214 | -26.8866 |
| *Lithobates catesbeianus* | -52.9253 | -26.8815 |
| *Lithobates catesbeianus* | -53.4722 | -29.4715 |
| *Lithobates catesbeianus* | -53.48 | -29.4751 |
| *Lithobates catesbeianus* | -53.4864 | -29.4742 |
| *Lithobates catesbeianus* | -51.14 | -29.36 |
| *Lithobates catesbeianus* | -51.4067 | -28.9997 |
| *Lithobates catesbeianus* | -52 | -26.5 |
| *Lithobates catesbeianus* | -47.8833 | -24.7167 |
| *Lithobates catesbeianus* | -51.1958 | -19.6717 |
| *Lithobates catesbeianus* | -35.2603 | -5.90619 |
| *Lithobates catesbeianus* | -52.41 | -28.26 |
| *Lithobates catesbeianus* | -51.9 | -26.7833 |
| *Lithobates catesbeianus* | -34.8833 | -7.95 |
| *Lithobates catesbeianus* | -47.2314 | -24.2873 |
| *Lithobates catesbeianus* | -46.9944 | -24.3163 |
| *Lithobates catesbeianus* | -52.9702 | -26.8321 |
| *Lithobates catesbeianus* | -52.9693 | -26.8317 |
| *Lithobates catesbeianus* | -52.9526 | -26.8364 |
| *Lithobates catesbeianus* | -52.9525 | -25.8394 |
| *Lithobates catesbeianus* | -52.9426 | -26.8154 |
| *Lithobates catesbeianus* | -52.9383 | -26.8129 |
| *Lithobates catesbeianus* | -47.64 | -22.71 |
| *Lithobates catesbeianus* | -49.1757 | -26.7406 |
| *Lithobates catesbeianus* | -42.925 | -20.3711 |
| *Lithobates catesbeianus* | -52.0205 | -26.8702 |
| *Lithobates catesbeianus* | -51.22 | -30.04 |
| *Lithobates catesbeianus* | -49.0101 | -25.3101 |
| *Lithobates catesbeianus* | -49.0015 | -25.2871 |
| *Lithobates catesbeianus* | -47.5712 | -22.3547 |
| *Lithobates catesbeianus* | -49.642 | -27.2153 |
| *Lithobates catesbeianus* | -52.3717 | -29.9846 |
| *Lithobates catesbeianus* | -51.8714 | -29.2897 |
| *Lithobates catesbeianus* | -38.5093 | -12.9717 |
| *Lithobates catesbeianus* | -52.4337 | -29.7158 |
| *Lithobates catesbeianus* | -53.8285 | -29.6952 |
| *Lithobates catesbeianus* | -53.7 | -29.7 |
| *Lithobates catesbeianus* | -51.7459 | -29.1688 |
| *Lithobates catesbeianus* | -48.2603 | -15.9697 |
| *Lithobates catesbeianus* | -53.443 | -29.6216 |
| *Lithobates catesbeianus* | -49.3794 | -20.8197 |
| *Lithobates catesbeianus* | -49.1957 | -25.5308 |
| *Lithobates catesbeianus* | -45.133 | -23.346 |
| *Lithobates catesbeianus* | -46.6429 | -23.7761 |
| *Lithobates catesbeianus* | -49.0833 | -16.7167 |
| *Lithobates catesbeianus* | -51.9322 | -28.7124 |
| *Lithobates catesbeianus* | -42.3935 | -22.6556 |
| *Lithobates catesbeianus* | -48.6103 | -16.6651 |
| *Lithobates catesbeianus* | -46.2999 | -23.5698 |
| *Lithobates catesbeianus* | -51.3966 | -30.6743 |
| *Lithobates catesbeianus* | -51.8603 | -29.8011 |
| *Lithobates catesbeianus* | -50.5833 | -24.2833 |
| *Lithobates catesbeianus* | -53.7602 | -27.3759 |
| *Lithobates catesbeianus* | -42.9664 | -22.4126 |
| *Lithobates catesbeianus* | -49.7556 | -29.3482 |
| *Lithobates catesbeianus* | -51.7181 | -29.9427 |
| *Lithobates catesbeianus* | -52.9913 | -29.1268 |
| *Lithobates catesbeianus* | -45.1325 | -23.4973 |
| *Lithobates catesbeianus* | -48.28 | -18.9 |
| *Lithobates catesbeianus* | -45.1 | -22.3333 |
| *Lithobates catesbeianus* | -51.56 | -28.94 |
| *Lithobates catesbeianus* | -50.98 | -30.09 |
| *Lithobates catesbeianus* | -42.8822 | -20.7542 |
| *Lithobates catesbeianus* | -42.2947 | -20.9617 |
| *Lithobates catesbeianus* | -51.5519 | -28.8689 |
| *Lithobates catesbeianus* | -40.4127 | -20.3311 |
| *Lithobates catesbeianus* | -52.5341 | -26.9615 |
