# Supplementary material for: Perspectives on invasive amphibians in Brazil
Source: PLoS One. 2017 Sep 22;12(9):e0184703. doi: 10.1371/journal.pone.0184703 (PMC5609743; doi:10.1371/journal.pone.0184703)
Supplement: S1 Fig — (A) Eleutherodactylus johnstonei; (B) Lithobates catesbeianus; (C) Scinax x-signatus; (D) Rhinella jimi; (E) Leptodactylus labyrinthicus; (F) Phyllodytes luteolus. (DOCX) [file pone.0184703.s003.docx]

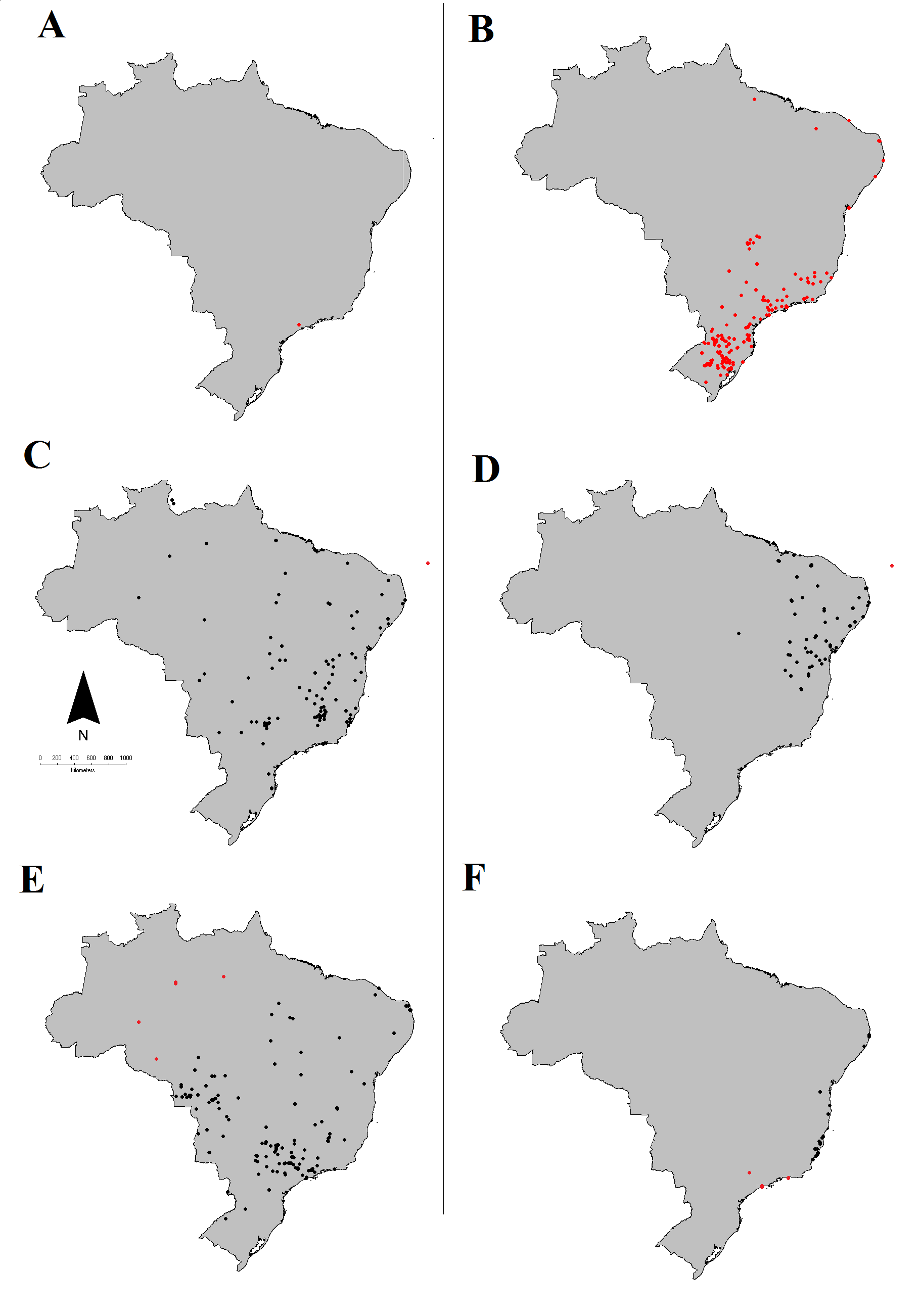


Figure S1. Maps with the invasive species distribution in Brazil; black dots as natural populations and red dots as invasive populations. (A) *Eleutherodactylus johnstonei*; (B) *Lithobates catesbeianus*; (C) *Scinax x-signatus*; (D) *Rhinella jimi*; (E) *Leptodactylus labyrinthicus*; (F) *Phyllodytes luteolus*.
